# Diversity and evolution of cytochrome P450 monooxygenases in Oomycetes

**DOI:** 10.1038/srep11572

**Published:** 2015-07-01

**Authors:** Mopeli Marshal Sello, Norventia Jafta, David R Nelson, Wanping Chen, Jae-Hyuk Yu, Mohammad Parvez, Ipeleng Kopano Rosinah Kgosiemang, Richie Monyaki, Seiso Caiphus Raselemane, Lehlohonolo Benedict Qhanya, Ntsane Trevor Mthakathi, Samson Sitheni Mashele, Khajamohiddin Syed

**Affiliations:** 1 Unit for Drug Discovery Research, Department of Health Sciences, Faculty of Health and Environmental Sciences, Central University of Technology, Bloemfontein 9300, Free State, South Africa; 2 Department of Microbiology, Immunology and Biochemistry, University of Tennessee Health Science Center, Memphis, TN 38163, USA; 3 College of Food Science and Technology, Huazhong Agricultural University, Wuhan, Hubei Province, China; 4 Department of Bacteriology, University of Wisconsin-Madison, 3155 MSB, 1550 Linden Drive, Madison WI 53706, USA

## Abstract

Cytochrome P450 monooxygenases (P450s) are heme-thiolate proteins whose role as drug targets against pathogens, as well as in valuable chemical production and bioremediation, has been explored. In this study we performed comprehensive comparative analysis of P450s in 13 newly explored oomycete pathogens. Three hundred and fifty-six P450s were found in oomycetes. These P450s were grouped into 15 P450 families and 84 P450 subfamilies. Among these, nine P450 families and 31 P450 subfamilies were newly found in oomycetes. Research revealed that oomycetes belonging to different orders contain distinct P450 families and subfamilies in their genomes. Evolutionary analysis and sequence homology data revealed P450 family blooms in oomycetes. Tandem arrangement of a large number of P450s belonging to the same family indicated that P450 family blooming is possibly due to its members’ duplications. A unique combination of amino acid patterns was observed at EXXR and CXG motifs for the P450 families CYP5014, CYP5015 and CYP5017. A novel P450 fusion protein (CYP5619 family) with an N-terminal P450 domain fused to a heme peroxidase/dioxygenase domain was discovered in *Saprolegnia declina*. Oomycete P450 patterns suggested host influence in shaping their P450 content. This manuscript serves as reference for future P450 annotations in newly explored oomycetes.

Ongoing genome sequencing momentum has resulted in genome sequencing of a large number of species from different biological kingdoms. Lower eukaryotic kingdoms occupy a special place among biological kingdoms because of the presence of a large number of species and their adaptation to diverse ecological niches. Genome sequencing of lower eukaryotes such as fungi revealed high diversity in their genomes compared to other biological kingdoms. For example, not only the presence of a large number of cytochrome P450 monooxygenases (P450s) was detected in many of their genomes, but also high diversity in terms of the number of P450 families^1^.

P450s are heme-thiolate proteins and ubiquitously present in species across the biological kingdoms^2^. These proteins are well known to perform enzymatic reactions in a stereo- and regio-specific manner^3^,^4^. Because of this characteristic these enzymes become critical in organisms’ primary and secondary metabolism, drug development, generation of human valuables and xenobiotic compound degradation^2^,^5^. Progress has been made in understanding P450s from lower eukaryotic organisms, such as their genome-wide annotation and comparative analysis^6^,^7^,^8^,^9^,^10^,^11^, heterologous expression and characterization^12^,^13^,^14^, that eventually resulted in identification of catalytically versatile P450s^15^,^16^, their engineering^17^ and further unraveling of their potential as anti-fungal drug targets^5^,^18^,^19^.

However, lower eukaryotes belonging to the kingdom Stramenopile, especially phylum Oomycota species P450s, have been underexplored. Oomycetes live as saprophytes or parasites^20^,^21^. These are organisms considered “hard-wired parasites”^22^. Oomycetes cause diseases in both plants and animals^20^,^21^,^23^. Oomycetes are counted among the most widespread and deadliest disease-causing agents of plants and crops worldwide. Their destructive behaviour lies in their ability to breach the host surface and break it down, promptly resulting in extensive destruction that hinders agricultural growth^24^. There has been a huge impairment of aquaculture owing to oomycetes and as for plants, serious diseases are caused not only in agriculturally and ornamentally important plants, but also other plants in the environment. A summary of diseases caused by oomycetes is listed in Table S1. To date, oomycetes remain a serious problem in agriculture and aquaculture^20^,^21^,^23^. Oomycete diseases are not commonly easy to control. Moreover, some oomycete species, particularly Phytophthora species, have the ability to build up resistance against chemicals by producing new genetically tougher strains. Plants are also very sensitive to oomycete attacks owing to their weak disease resistance.

The impact of oomycete species on the economy triggered various investigations on pathogenesis and control methods for these pathogens. In the quest to find a remedy, genome sequencing of oomycetes was carried out^25^,^26^,^27^,^28^,^29^,^30^,^31^. Genome sequencing analysis of oomycetes revealed the presence of a moderate number of P450s^27^. However, the genome sequencing studies were limited to mentioning the count of P450s in oomycetes^27^. A comparative P450 genomics study was limited to P450 analysis in a few species^7^. The study showed that the CYP51 of oomycetes can be a good drug target against these pathogens^32^,^33^. Despite this great importance, analysis of P450 enzymes in oomycetes has been underexplored. The recent public availability of quite a number of oomycete genomes^25^,^26^,^27^,^28^,^29^,^30^,^31^ gives us an opportunity to perform comprehensive comparative analysis of P450s in these species. In this study we performed systematic analysis of P450s across 13 oomycete species. Furthermore, considering that the lower eukaryote fungi P450s are well annotated and the poor availability or unavailability of other lower eukaryotic P450s, in this study we compared Oomycota P450s with different fungal phyla P450s.

## Methods

### Oomycete species for P450 analysis

Thirteen oomycete species belonging to two different classes and three different orders were used in this study. The oomycete species used in this study, their taxonomic group and general information such as their host and diseases caused by these organisms were listed in Table S1. As listed in Table S1, 11 species (*Phytophthora sojae*, *P. ramorum*, *P. infestans*, *P. parasitica*, *P. capsici*, *Hyaloperonospora arabidopsidis* (formerly *Hyaloperonospora parasitica*), *Pythium aphanidermatum*, *P. irregular*, *P. awayamai*, *P. ultimum* and *P. vexan*) belonging to class Peronosporomycetidae and two species (*Saprolegnia parasitica* and *S. declina*) belonging to Class Saprolegniomycetidae are used for comparative analysis of P450s. It is noteworthy that Peronosporomycetidae contain plant pathogens whereas Saprolegniomycetidae contain animal pathogens.

### Genome data-mining and identification of P450s

Oomycete species genomes whose details have been published (Table S1) and are publicly available (Table S2) were used in this study. The whole proteomes of oomycete species were downloaded from the databases listed in Table S2. Identification of P450 proteins in whole proteome is carried out using the procedure described elsewhere^11^,^16^. Briefly, the downloaded protein sequences were grouped into different protein families using the National Centre for Biotechnology and Information (NCBI) Conserved Domain Database: NCBI Batch Web CD-search tool^34^. The proteins grouped under the cytochrome P450 monooxygenases superfamily were selected for further study.

### Assigning a family and subfamily to orphan P450s

The above selected P450s were subjected to BLAST analysis against all named protist sequences on the Cytochrome P450 Webpage^35^. Based on percentage identity, i.e., family members share more than 40% amino acid identity and members of subfamilies share more than 55% amino acid homology, families and subfamilies were assigned to oomycete P450s. P450s that showed less than 40% identity were assigned to a new family. In addition, evolutionary analysis of P450s was performed in order to authenticate the annotation. P450s that showed less than 40% identity were assessed for their position on the phylogenetic tree and based on their location/alignment with other P450s they were assigned to different P450 families. The annotated and publicly available *P. sojae* and *P. ramorum* P450s were retrieved from the database^35^ and used in this study.

### Phylogenetic analysis of oomycete P450s

The phylogenetic tree was constructed for evolutionary analysis of oomycete P450s. Firstly, the protein sequences were aligned by adjusting them to the hidden Markov model of P450s in the Pfam protein families database (http://pfam.xfam.org/family/PF00067) with HMMER package 3.1 (http://hmmer.janelia.org/)^36^,^37^. Then, the phylogenetic tree from the alignment of protein sequences was inferred by FastTree version 2.1.4 using the maximum-likelihood method (http://www.microbesonline.org/fasttree/)^38^. The generated tree data were submitted to iTOL (http://itol.embl.de/upload.cgi) for viewing phylogenetic trees and making figures^39^.

### Analysis of homology

Percentage identity between P450s was determined using ClustalW2 multiple sequence analysis^40^. The ClustalW2 result file designated as percentage identity matrix was downloaded and checked for the percentage identity between P450s.

### P450 diversity percentage

The percentage contribution of the number of P450 families in the total number of P450s in an organism is considered as P450 diversity percentage. P450 count and P450 families in fungal species were retrieved from published literature^9^,^10^,^11^,^35^.

### Analysis of tandem arrangement of P450s

P450s localized in proximity on the genome were identified by scanning manually in the respective genome databases for each oomycete (Table S2). P450s localized on the same scaffold/contig/supercontig were noted. P450s that were tandemly localized and belonged to the same family were expressed as percentage in the total number of P450s in an organism. Tandem arrangement of P450s was not carried out for *P. irregulare* and *P. iwayamai* because of the shorter length of scaffold/contig/supercontig.

### Analysis of EXXR and CXG motifs

Identification of P450 family-specific amino acid patterns at EXXR and CXG motifs was carried out using the procedure described elsewhere^41^. Briefly, P450 members were subjected to ClustalW multiple alignment using Molecular Evolutionary Genetics Analysis (MEGA 5.2.2)^42^. After ClustalW alignment of P450s, amino acids in the EXXR and CXG motifs were selected and used for generation of WebLogos and calculation of percentage contribution by an amino acid at each position in the motifs. Only four amino acids were selected for EXXR motif analysis, whereas for CXG motifs upstream seven amino acids were included in the analysis, exactly as previously described^41^.

### Generation of sequence logos

Sequence logos for EXXR and CXG motifs were generated using the published method^41^. Briefly, WebLogo, a sequence logo generator programme (http://weblogo.threeplusone.com/create.cgi)^43^,^44^, was used to create sequence logos at EXXR and CXG motifs. After ClustalW alignment of member P450s, the EXXR and CXG (FXXGXRXCXG) motifs’ amino acids were selected and pasted in the WebLogo program. As a selection parameter, image format was selected as PDF and 32 symbols per line were selected. The generated EXXR and CXG sequence logos were used for the analysis.

### Genome data mining, annotation and phylogenetic analysis of P450 fused proteins

Publicly available lower eukaryote genomes, especially basidiomycetes (http://genome.jgi-psf.org/programs/fungi/index.jsf)^45^, were mined for P450 fused proteins (heme dioxygenase/peroxidase domain fused to P450 domain proteins). BLAST was performed using P450 fused protein CYP6001A1 and CYP6002A1 from *Aspergillus nidulans*^46^ against lower eukaryote genomes. The whole protein with both heme dioxygenase/peroxidase and P450 domain and the protein sequence with only heme dioxygenase/peroxidase domain were used for BLAST. The resulting hit proteins were subjected to NCBI Batch Web CD-search^34^. Proteins with both domains were selected as P450 fused proteins. The selected P450 fused proteins were assigned to different P450 families or subfamilies following the above described criteria. The domain organization in the P450 fused protein is recorded using the NCBI Batch Web CD-search^34^. The ascomycete P450 fused proteins were retrieved from recently published literature^8^. Phylogenetic analysis of P450 fused protein was carried out using the minimum evolution method^47^. The phylogenetic tree was constructed using MEGA 5.2.2^42^.

## Results and Discussion

### Oomycetes P450omes

Genome-wide identification and annotation of P450s in 13 oomycetes belonging to two different classes and three different orders (Table S1) revealed the presence of a moderate number of P450s in their genomes (Table 1). Three hundred and fifty-six P450s were found in 13 oomycetes genomes (Table S3). The P450 count in oomycete genomes ranged from 7-41. Among the oomycetes selected for the study, *H. arabidopsidis* showed the lowest number of P450s (7) and *P. iwayamai* showed the highest number of P450s (41) in their genome. Except *H. arabidopsidis*, all oomycete genomes had 19 or more P450s (Table 1). On average, Peronosporales showed a lower number of P450s (27), excluding *H. arabidopsis*, compared to Pythiales that showed 31 P450s. Comparison of oomycete P450omes with other lower eukaryotes such as fungi revealed that the number of P450s observed in oomycetes is most similar to fungal species belonging to the subphylum saccharomycotina and least similar among species belonging to the rest of the fungal kingdom, with a few exceptions, as shown in Table S4.

### P450 families and subfamilies in oomycetes

**Figure 1:**
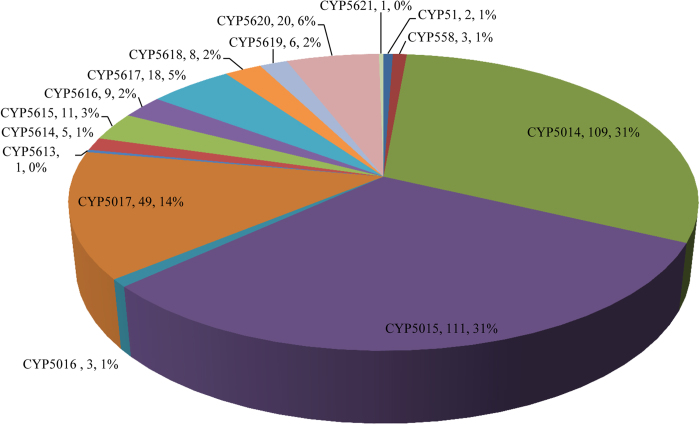
Comparative analysis of P450s in 13 oomycete animal and plant pathogens. Three hundred and fifty-six P450s were grouped under 15 P450 families. The P450 family name, number of member P450s and their percentage in the total number of P450s are shown in the figure.

Annotation of P450 families and subfamilies in 13 oomycete genomes revealed the presence of 15 P450 families (Fig. 1) and 84 P450 subfamilies (Table S5). Nine new P450 families and 31 new P450 subfamilies were found in oomycetes. The nine new P450 families are CYP5613, CYP5614, CYP5615, CYP5616, CYP5617, CYP5618, CYP5619, CYP5620 and CYP5621. New subfamilies were confined to four P450 families: CYP5014 showed 15 new subfamilies, followed by the CYP5015 and CYP5017 families each with seven new subfamilies, and CYP558 with two new subfamilies. A detailed analysis of P450 families and subfamilies and their member P450s was listed in Table S5.

Comparative analysis of member P450s across 13 P450 families revealed that the CYP5014, CYP5015 and CYP5017 P450 families are the dominant P450 families in oomycetes with 109, 111 and 49 members comprising 76% of the total P450s (Fig. 1). This suggests a high level of P450 blooming^48^ of these families. A detailed analysis of P450 bloom in oomycetes is discussed in the next section of this manuscript. A single member was found in CYP5613 and CYP5621 families (Fig. 1). Analysis of P450 families, particularly their member P450s and their contribution to the total number of P450s, is shown in Fig. 1.

### P450 family and subfamily dynamics in oomycetes

After annotation of families and subfamilies, further study was carried out to assess the dynamics of P450 families and subfamilies (loss or gain of P450 families/subfamilies) in these organisms. Among oomycetes, Saprolegniales showed the highest number of P450 families compared to Peronosporales and Pythiales (Table 1). The number of P450 families in oomycetes ranged from two to nine. Peronosporales showed a minimum of two and a maximum of four families in their genomes. Pythiales showed three to four P450 families in their genomes. Species belonging to Saprolegniales showed six (*S. parasitica*) and nine (*S. declina*) P450 families in their genomes (Table 1).

**Figure 2:**
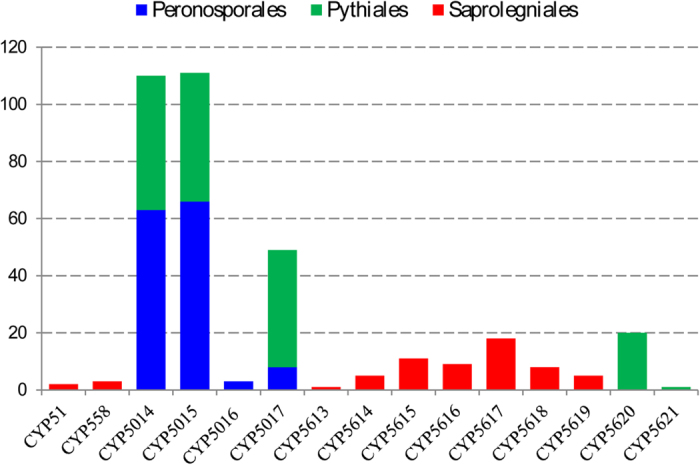
P450 family-level comparative analysis between three oomycete orders: Peronosporales, Pythiales and Saprolegniales. The Y-axis represents number of P450s.

Comparative analysis of P450 families revealed no common P450 family across the oomycetes used in this study (Fig. 2). The CYP5016 family is present only in Peronosporales and the CYP5014, CYP5015 and CYP5017 families are present in both Peronosporales and Pythiales. Saprolegniales has eleven P450 families (CYP51, CYP558 and CYP5613-CYP5621). Nine of them (CYP5613-CYP5621) are new P450 families only found in Saprolegniales. The answer to the presence of the highest number of P450 families and particularly the presence of new P450 families in Saprolegniales compared to Peronosporales and Pythiales can be obtained from a recently published genome sequencing study^31^. Genome sequencing analysis of *S. parasitica* revealed that loss of heterozygosity is an efficient mechanism for new variant genes to adapt to a distinct animal pathogenic lifestyle^31^. The presence of distinct P450 families (new P450 families) in Saprolegniales compared to Peronosporales and Pythiales (Fig. 2) suggested that P450s in these organisms play a key role in their adaptation to a pathogenic lifestyle (animal host). One interesting observation is that the CYP51 family, involved in membrane sterols biosynthesis^5^, is only found in Saprolegniales (Fig. 1). The loss of CYP51 in other oomycetes implied dependence on the host sterols.

**Figure 3:**
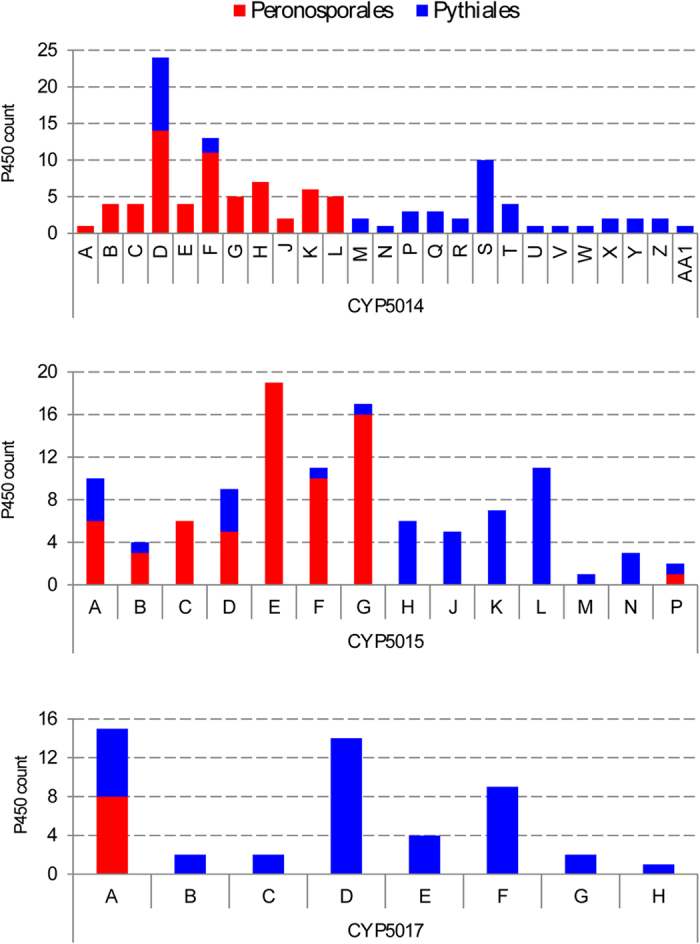
Comparative analysis of CYP5014, CYP5015 and CYP5017 families between Peronosporales and Pythiales.

The distinct pattern observed in P450 families among oomycete species was also reflected in P450 subfamilies (Fig. 3 and Table S5). Comparative analysis of subfamilies revealed that only nine subfamilies were shared between Peronosporales and Pythiales. Analysis of P450 subfamilies revealed that all the subfamilies shared between Peronosporales and Pythiales were found in CYP5014 (2), CYP5015 (6) and CYP5017 (1) (Fig. 3). This suggests that distinct pathogenic lifestyles (host and site of infection) of Peronosporales and Pythiales (Table S1) influenced the P450 content in their genomes, as species belonging to these orders show distinct P450 subfamilies (Fig. 3 and Table S5).

The above results revealed that oomycetes belonging to different orders show distinct P450 families and P450 subfamilies in their genomes. This strongly suggests that oomycetes belonging to different orders retained or evolved distinct P450 families in their genomes possibly to adapt to a pathogenic lifestyle in different hosts. In other ways, as recently suggested by researchers^31^, host cellular environment has driven distinct patterns of gene expansion and loss in the genomes of plant and animal pathogens. From the above results it is clear that the host environment played a key role in the development of distinct/new variants of P450 families in oomycetes.

### Evolutionary analysis of oomycete P450s

The presence of distinct P450 families, particularly new families and subfamilies, in oomycetes necessitated the performance of evolutionary analysis to allow grouping of P450s into different clades, a higher level P450 classification^49^. Furthermore, evolutionary analysis of oomycetes P450s played a key role in the annotation of oomycete P450s into different families.

**Figure 4:**
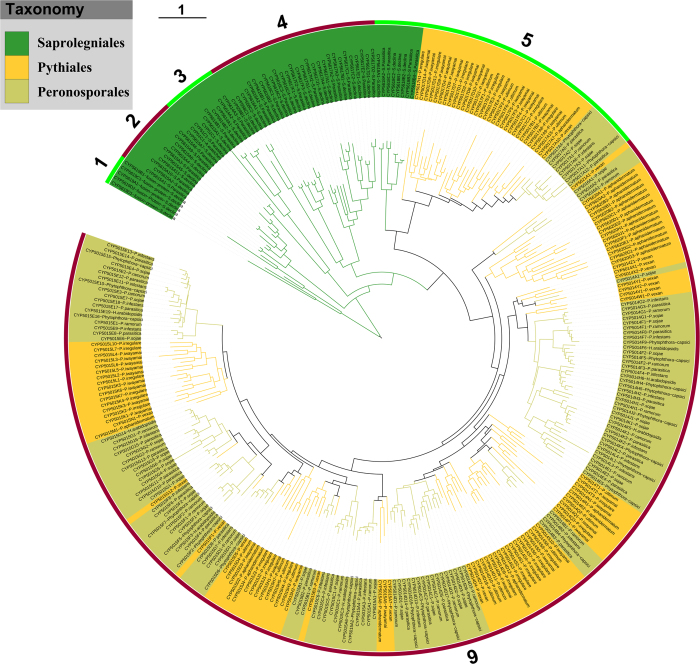
Phylogenetic tree of P450s in 13 oomycete species. The inner circle is the phylogenetic tree of annotated oomycete P450s. The branches with different colors show their taxonomic groups, as indicated in the legend. Ancestral branches with children that had identical colors were assigned the same color as the children. The middle circle is the taxon represented as P450 family, followed by the corresponding oomycete species name, which is covered by different colors to show its taxonomic group, as the legend indicates. Each taxon links the branch with a dotted line. The outer numbers indicate the six clades derived in this study and their ranges are marked by alternating reddish brown and green. A high-resolution phylogenetic tree is provided in the Supplementary data (Fig. S1).

Hence in this study a phylogenetic tree of oomycete P450s was constructed for their evolutionary analysis (Fig. 4). The results showed that the phylogenetic relationship of oomycete P450s was related with their family and species taxonomy. On the whole, the P450s of the order Saprolegniales showed a very distant phylogenetic relationship to those of the order Pythiales and Peronosporales; they were clearly separated in the tree, while the P450s from the order Pythiales and Peronosporales were phylogenetically close. This is in agreement with the taxonomy relationship between the orders Saprolegniales, Pythiales and Peronosporales, which suggested that the evolution of oomycete P450s was closely related with their species’ evolution.

Based on phylogenetic relationships, oomycete P450s are classified into six clades (Fig. 4), and the distribution of CYP families and oomycete taxonomy is investigated in these clades (Table S6). Only clade 5 has members from all three orders. Clade 6 is a very large branch, suggesting it is blooming in the order Pythiales and Peronosporales. Especially CYP5014 and CYP5015 members are not only very frequently presented in the order Pythiales and Peronosporales, but also maintain a high gene number in their genomes (Figs 1,3 and Table S5). This suggests that CYP5014 and CYP5015 family members may play a pivotal role in the physiological function of order Pythiales and Peronosporales.

### P450 blooming in oomycetes

Comparative analysis of P450s in arthropods, mainly insects, revealed the presence of P450 families with the highest number of members in their genomes and authors termed this nature of the highest number of members for a P450 family “P450 family blooming”^48^. A recent study on fungal P450s also revealed the blooming nature of a large number of P450 families in fungi^16^. Blooming of P450 families might play a key role in an organism’s metabolism or its adaptation to diverse ecological niches, for example fungal colonization of wood substrates^16^.

In order to analyse P450 bloom or its direct opposite P450 diversity in oomycetes, we performed a comprehensive comparison of P450 count and P450 families between Oomycota and different fungal phyla (Table S4). Another reason for using fungal organisms for comparison, apart from what is mentioned in the introduction, is that for a long time oomycetes were regarded as true fungi; it was only recently that these organisms were grouped under the biological kingdom “Stramenopile”. Furthermore, analysis of P450 diversity/blooming between these organisms will provide insights in evolution of P450 families, considering the primitive nature of oomycetes.

**Figure 5:**
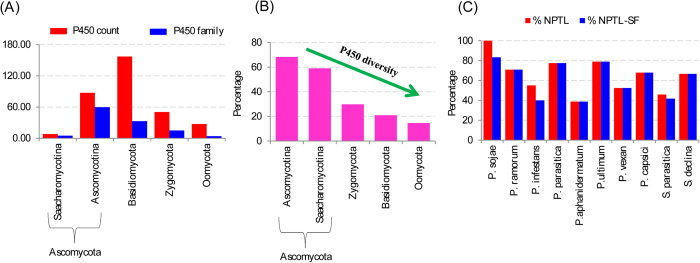
Comparative P450 diversity analysis between Oomycota and other lower eukaryote phyla (**A,B**) and P450 bloom analysis in oomycetes (**C**). A comparative analysis of the average number of P450s and P450 families (**A**) and P450 diversity percentage (**B**) between different phyla is shown in the figure. Detailed analysis of P450 count, families and measured P450 diversity percentage is represented in Table S4. As shown in Panel B, Oomycota showed the lowest diversity compared to different fungal phyla, indicating P450 blooming in these organisms. P450 family blooming in oomycetes was measured (i) percentage of number of P450s tandemly localized on the same scaffold (%NPTL) and (ii) percentage of NPTL belonging to the same family (%NPTL-SF) in the total number of P450s in a species. Detailed analysis of P450s that are tandemly localized on scaffolds in each species and %NPTL and %NPTL-SF is presented in Table S8.

Comparative analysis of P450 families across Oomycota and other fungal phyla revealed that a number of P450 families present in oomycetes are to some extent matched with species belonging to Ascomycota, particularly the subphylum Saccharomycotina (Table S4). In order to identify the P450 diversity/blooming in oomycetes we measured an average number of P450s and an average number of P450 families across different phyla and measured the average P450 diversity percentage (Fig. 5 and Table S4). As shown in Fig. 5A, the average P450 count and average P450 families in Oomycota were found to be lowest (excluding Saccharomycotina species in Ascomycota) compared to different fungal phyla. This indicates the low diversity of P450s in oomycetes. On the other hand, this implies the highest blooming of P450 families in oomycetes. To identify the blooming nature of Oomycota P450ome we measured the average P450 diversity percentage between Oomycota and other fungal phyla (Fig. 5B). As shown in Fig. 5B, Oomycota showed the lowest P450 diversity percentage (15%) compared to other fungal phyla indicating the highest P450 blooming in oomycetes or the lowest diversity.

A contribution of 76% of P450s by three P450 families CYP5014, CYP5015 and CYP5017 (Fig. 1) resulted in the lowest diversity in oomycetes. This suggests the blooming of CYP5014, CYP5015 and CYP5017 families in oomycetes. The blooming nature of P450 families is attributed by tandem duplication of their members^16^,^48^. The duplicating nature of member P450s is easily identified either by the highest identity at protein level or analysis of the gene structure (analysis of introns and exons) between members. Since the oomycetes, genes show the lowest number of introns in their structure^20^, it is not ideal to perform gene structure analysis to identify genome-duplicated P450s. Hence, in this study, we used protein percentage identity criteria to identify P450s that were possibly duplicated in oomycetes.

To assess the duplicate nature of P450s, oomycete P450omes were subjected to ClustalW2 analysis^40^. The percentage identity between oomycete P450s was analysed and the proteins showing more than 70% identity were selected and presented in Table S7. As shown in Table S7, a large number of P450s (115) showed more than 70% identity and 82 P450s showed more than 80% identity. This indicates that the majority of the oomycete P450s are highly conserved in their primary structure. Analysis of P450s with respect to the P450 families revealed that all of the P450s that showed more than 70% identity belong to three P450 families, i.e. CYP5014, CYP5015 and CYP5017, except three P450s belonging to the CYP5016 family and two P450s belong to CYP5619 family (Table S7). This suggests that member P450s in these P450 families possibly increased their number through duplication, which resulted in blooming of these P450 families.

### Tandem localization of oomycete P450s

Tandem localization of P450s, particularly P450s belonging to the same P450 family, is a good indication of P450 duplications. In order to analyse P450 duplications in oomycetes we proceeded to analyse the localization of P450s. As shown in Fig. 5C and Table S8, a large number of P450s were found to be tandemly arranged in oomycetes. Tandem arrangement of P450s in oomycetes ranged from 39% to 100% in the total number of P450s (Fig. 5C). The highest number of tandemly arranged P450s were found in *P. sojae*, where all the P450s (100%) were tandemly arranged. *P. aphanidermatum* showed the lowest number of tandemly arranged P450s (30%) in its genome. *H. arabidopsidis* showed no tandemly arranged P450s possibly due to low copy of P450s in its genome. Analysis of tandemly arranged P450s revealed that all the P450s that were tandemly arranged belonged to the same P450 family in all analyzed organisms except in *P. infestans,* where only 40% of P450s belonged to the same family (Fig. 5C and Table S8).

Family level analysis of tandemly localized P450s revealed that all of the tandemly localized P450s belonged to the P450 families that showed blooming in the respective species (as discussed above). For example, CYP5014-CYP5017, CYP5615-CYP5620 and CYP558 family members were found tandemly arranged in oomycetes. It is interesting to note that the P450 family CYP558 in *S. declina* its two members were found tandemly localized (Table S8). Based on evolutionary analysis, sequence identity data and tandem arrangement, we conclude that many P450 families in oomycetes are bloomed, owing to tandem duplication of their members.

### Oomycete P450 family characteristic amino acid patterns at EXXR and CXG motifs

**Figure 6:**
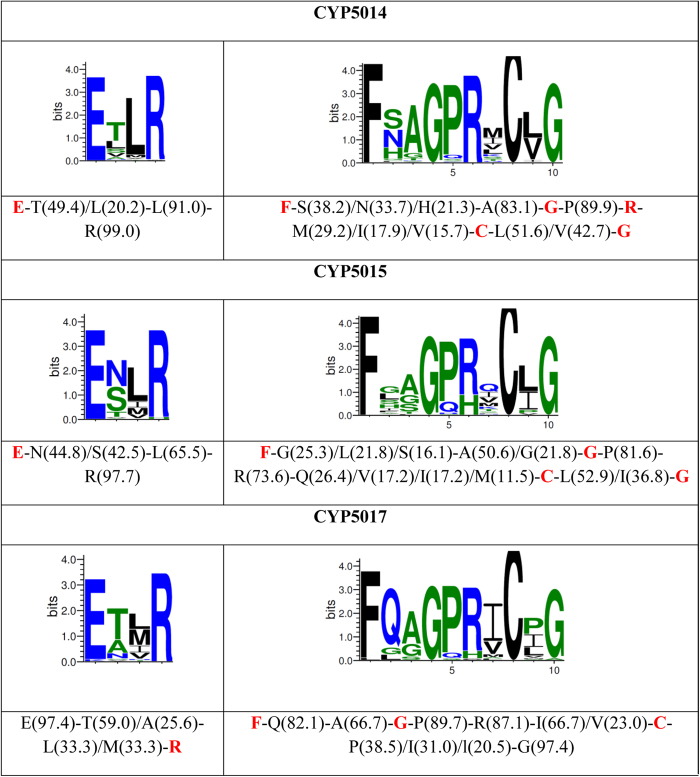
Analysis of amino acid combinations at EXXR and CXG motifs in CYP5014, CYP5015 and CYP5017 families. The P450s used for deducing amino acid combinations are shown in Table S9. Sequence logos were constructed as described in Methods. The amino acids and their percent occurrence at each of this domain are also presented. The invariant residues at these motifs were shown in bold with red font.

A recent study revealed that a certain combination of amino acid patterns at EXXR and CXG motifs are characteristic of a P450 family^41^. Authors have suggested that these amino acid patterns are evolved during the P450 family divergence from a common ancestor and are retained in family members as a characteristic of the family^41^. Considering the large number of member P450s, in this study, we analysed amino acid combinations for P450 families such as CYP5014, CYP5015 and CYP5017 (Fig. 6 and Table S9). Analysis of the EXXR motif revealed that the first and fourth amino acids of this motif “E” and “R” is conserved in all P450 families CYP5014, CYP5015 and CYP5017 with rare exceptions. CYP5017F8 showed “K” instead of “E” and CYP5014N1 and CYP5015L showed “W” and “H” instead of “R” (Fig. 6). Non-conservation of “E” and “R” amino acids at the EXXR motif are reported rarely^50^. Leucine is the major amino acid appearing at the third position in this motif in all three oomycete P450 families. Threonine is the predominant amino acid at the second position in P450 families CYP5014 and CYP5017, whereas serine and aspargine are the predominant amino acids at this position in the CYP5015 family (Fig. 6). Compared to P450 families across the biological kingdoms^41^, oomycete P450 families CYP5014 and CYP5017 also showed ETLR as predominant amino pattern. However, the E-S/N-L-R amino acid pattern where “S/N” is the predominant amino acid at the second position is unique to the CYP5015 family and this pattern was not found in P450 families published in the literature^41^. Analysis of the CXG motif (FXXGXRXCXG) across the three P450 families revealed conservation of amino acids such as “F”, “G” and “C” at the first, fourth and eighth positions. These amino acids at these positions are well known to be conserved in the P450s across the biological kingdoms^41^,^51^,^52^, with some P450s showing variant amino acids at these positions^50^. The canonical amino acids “R” and “G” at the sixth and tenth positions are conserved in the CYP5014 family and are predominant in CYP5015 and CYP5017 (Fig. 6). The amino acid pattern at the CXG motif of the CYP5017 family is to some extent matched with the CYP94 and CYP704 families^41^ where “Q” is dominant at the second position in all these P450 families. However, differences were found at the seventh and ninth position amino acids among the three P450 families CYP5017, CYP94 and CYP704. Comparison of CXG motif amino acid patterns for CYP5014 and CYP5015 with published P450 families CXG motif amino acid patterns^41^ suggested that these families have unique amino acid patterns. This strongly supports the hypothesis previously proposed^41^ that the amino acid pattern at these motifs is unique for a P450 family. Overall, amino acid patterns at the EXXR and CXG motifs of the three oomycete P450 families CYP5014, CYP5015 and CYP5017 are unique and these amino acid patterns (Fig. 6) can be considered characteristics of these P450 families.

### Novel P450 fused proteins in oomycetes

P450s fused to redox partners and also to different proteins are well documented in the literature^53^,^54^. Two different types of P450 fused proteins were reported in lower eukaryotes. These two different types were: (i) P450 fused to CPR at the C-terminal end, which is well-known as P450foxy (CYP505 family)^55^ and (ii) P450 fused to heme peroxidase/dioxygenase at the N-terminal end (CYP6001 family)^46^.

**Figure 7:**
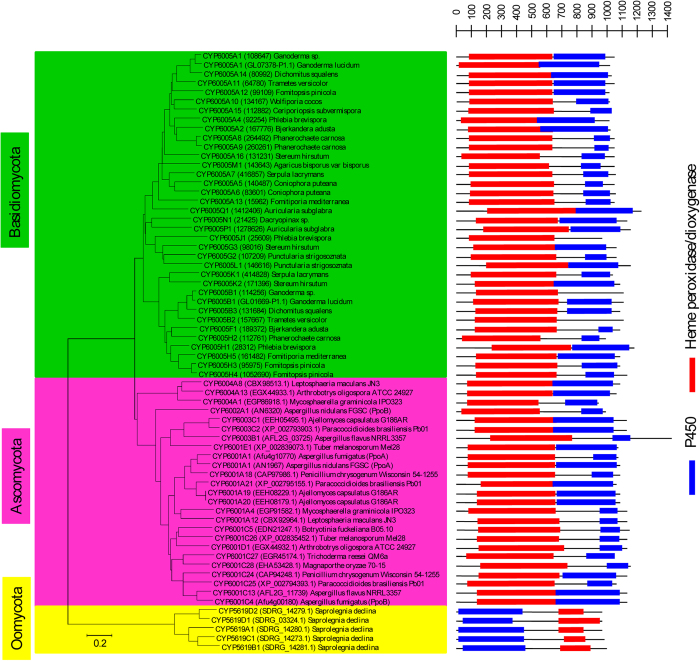
Phylogenetic and structural analysis of P450 fused proteins (heme peroxidase/dioxygenase fused to P450) between Oomycota and different fungal phyla. Sixty-six P450 fused proteins were used for the construction of a phylogenetic tree. Structural analysis of P450 fused proteins were carried out as described in ‘Methods’. The heme peroxidase/dioxygenase and P450 domain boxes is indicative of the domain length. For three P450 fused proteins (CYP6005J1, CYP6005B1 and CYP6005B2) NCBI CDD^34^ did not identify the P450 domain length, suggesting the presence of non-variant amino acids at P450 signature motifs in these proteins. A detailed analysis of the P450 fused proteins, species, size of the proteins and size of each of the domains is presented in Table S8.

Analysis of P450s in oomycetes revealed the presence of P450 fused proteins. The new P450 family CYP5619 with six members found in *S. declina* is fused to heme peroxidase/dioxygenase protein. However, the combination of fusion is different compared to the reported combination of P450 fused proteins in lower eukaryotes^53^,^54^. In oomycetes, the heme peroxidase/dioxygenase protein is fused at the C-terminal end to P450 (Fig. 7). This combination, i.e. N-terminal P450 domain fused to heme peroxidase/dioxygenase at its C-terminal, is a novel combination and not reported in the literature^53^,^54^. To confirm the novelty of this P450 fused protein, we performed comprehensive genome data mining to identify fused P450s, particularly heme peroxidase/dioxygenase protein fused to P450 in the publicly available lower eukaryote genomes^45^. A total number of 61 P450 fused proteins were identified (Fig. 7 and Table S10). The identified P450 fused proteins were grouped under five different P450 families namely CYP6001-CYP6005. Interestingly, the CYP6005 family is found only in Basidiomycota, whereas Ascomycota showed four different P450 fused families, CYP6001-CYP6004 (Fig. 7 and Table S10). The identified P450 fused proteins were subjected to heme peroxidase/dioxygenase and P450 domain analysis. As shown in Fig. 7, all the P450 fused proteins (CYP6001-CYP6005 family) identified in fungi showed an N-terminal heme peroxidase/dioxygenase domain and a P450 domain at the C-terminal end. This clearly confirms that the combination identified in CYP5619 family members is novel. Considering the nature of P450 fused protein, it can be concluded that CYP5619 family members are possibly involved in the oxidation of fatty acids, like CYP6001A1^46^. However, experimental analysis is needed to unravel the difference between the two different domains’ combinations.

### Functional analysis of oomycete P450s

All the oomycete P450s (except CYP51) were orphans, as functional data on oomycete P450s have not been reported. However, based on homology to characterized P450 proteins possible functional role(s) for oomycete P450s can be predicted. The CYP51 family identified in Saprolegniales plays a key role in synthesis of membrane sterols^32^,^33^. A recent study showed that CYP51 in these organisms can serve as a novel drug target^32^,^33^. Based on functional analysis of CYP6001A1 of *A. nidulans*^46^ it is tempting to speculate that CYP5619 family members play a role in fatty acid hydroxylation. Based on homology to fatty acid hydroxylases CYP5014-CYP5017, family members may play a role in fatty acid metabolism^7^. The above predictions were based on homology data, as mentioned in this article (for CYP51 and CYP5169) and one published prediction (for CYP5014-CYP5017)^7^. Future study will involve unravelling the P450s’ role, if any, in successful adaption to the parasitic and saprophytic lifestyle of oomycetes.

In conclusion, oomycetes are very important organisms in terms of their hard-wired parasitism leading to the loss of billions of dollars in agriculture and aquaculture. Analysis of P450s in these organisms provided insights into the evolutionary pattern of P450s. Genome-wide analysis of P450s revealed the presence of moderate number of P450s in these organisms. Despite presence of a large number of new P450 families and subfamilies, P450 family blooming resulted in a low P450 diversity in oomycetes. CYP51 and novel P450 fusion proteins with different combination of heme peroxidase/dioxygenase and P450 domain were common between oomycetes and with lower eukaryote fungi. The speciation and adaption to diverse ecological niches or lifestyle of oomycetes and fungi resulted in generation of distinct P450 families in both groups. Furthermore, at order level, oomycetes showed distinct P450 families and subfamilies. This confirms that the host influence is a major factor in shaping the oomycetes genomic content and thus also reflected in terms of P450s. Presence of unique combination of amino acid patterns at EXXR and CXG motifs in oomycete P450 families strongly supported previously proposed hypothesis that the amino acid patterns at these motifs are characteristic of a P450 family. This study serves as a reference and opened new vistas for future genome-wide annotation of P450s in oomycetes. Future genome sequencing of more oomycetes provides a broader picture of P450 evolution in these organisms and also possibly results in discovery of novel P450 families.

## Additional Information

**How to cite this article**: Sello, M. M. *et al.* Diversity and Evolution of Cytochrome P450 Monooxygenases in Oomycetes. *Sci. Rep.* **5**, 11572; doi: 10.1038/srep11572 (2015).

## Supplementary Material

Supplementary Information

Supplementary Information

Supplementary Figure S1

## Figures and Tables

**Table 1:** Comparative P450 analysis in 13 oomycete species.

Species name	No. of P450s	No. of P450 families	No. of P450 subfamilies
*Phytophthora sojae*	30	4	18
*Phytophthora parasitica*	31	4	18
*Phytophthora ramorum*	24	4	17
*Phytophthora infestans*	20	3	14
*Phytophthora capsici*	28	3	17
*Hyaloperonospora arabidopsidis*	7	2	7
*Pythium irregulare*	41	3	17
*Pythium aphanidermatum*	31	4	18
*Pythium ultimum*	19	3	12
*Pythium iwayamai*	42	3	19
*Pythium vexan*	20	4	15
*Saprolegnia parasitica*	24	6	16
*Saprolegnia declina*	39	9	26
