# Supplementary material for: Diversity and evolution of cytochrome P450 monooxygenases in Oomycetes
Source: Sci Rep. 2015 Jul 1;5:11572. doi: 10.1038/srep11572 (PMC4486971; doi:10.1038/srep11572)
Supplement: Supplementary Information [file srep11572-s1.pdf]

Diversity and evolution of cytochrome P450 monooxygenases in Oomycetes

Mopeli Marshal Sello[1], Norventia Jafta[1], David R Nelson[2], Wanping Chen[3], Jae-Hyuk Yu[4], Mohammad Parvez[1], Ipeleng Kopano Rosinah Kgosiemang[1], Richie Monyaki[1], Seiso Caiphus Raselemane[1], Lehlohonolo Benedict Qhanya[1], Ntsane Trevor Mthakathi[1], Samson Sitheni Mashele[1*], Khajamohiddin Syed[1*]

* Corresponding authors email: khajamohiddinsyed@gmail.com & smashele@cut.ac.za

Table S1. List of oomycete species selected for the study and the general information such as their host and diseases caused by these pathogenic species are shown in the table.

Taxonomic Group	Species name	Life style	Host	General information	References
**Class:** Peronosporomycetidae **Order:** Peronosporales	*Phytophthora sojae* and *Phytophthora ramorum*	Saprophytic and parasitic	Plants	These species are considered as model species for the Phytophthora genus owing to well-developed genetic and genomics resources including genetic maps, BAC libraries and EST sequences. *P. sojae* cause soybean root and stem rot and leads to substantial yield losses annually. *P. ramorum* causes sudden oak death, tanoak and ramorum blight on woody ornamental forest under canopy plants. It causes stem cankers on trees and leaf blight or stem dieback on ornamentals and under storey forest species	25
**Class:** Peronosporomycetidae **Order:** Peronosporales	*Phytophthora infestans*	Saprophytic and parasitic	Plants	It is the causative agent of late blight disease in potato and tomato plants. Responsible for the famous Irish potato famine in the mid-nineteenth century	26
**Class:** Peronosporomycetidae **Order:** Peronosporales	*Phytophthora parasitica*	Saprophytic and parasitic	Plants	A model species of oomycete pathogens. Causes destructive diseases to a wide variety of crops including tomato, eggplant, pepper, tobacco, potato, walnuts, fruits and wide range of nursery and ornamental plants and forest ecosystems	56
**Class:** Peronosporomycetidae **Order:** Peronosporales	*Phytophthora capsici*	Saprophytic and parasitic	Plants	Attacks the roots, stems, leaves, and fruits of pepper, resulting in damping-off, seedling blight, foliar blight and death. Other plants that are affected include tomato, eggplant, cucumber, watermelon, pumpkin, squash, and cocoa	29
**Class:** Peronosporomycetidae **Order:** Peronosporales	*Hyaloperonospora arabidopsidis* (formerly *Hyaloperonospora parasitica*)	Obligate parasite	plant	The causal agent of the downy mildew of the plant model organism *Arabidopsis thaliana*	28
**Class:** Peronosporomycetidae **Order:** Pythiales	*Pythium aphanidermatum*	Saprophytic and parasitic	Plants	Causes damping off, root and stem rots, and blights of grasses and fruits, papaya, beets, pepper and cotton	30
**Class:** Peronosporomycetidae **Order:** Pythiales	*Pythium irregulare*	Saprophytic and parasitic	Plants	Highly pathogenic on wide range of cereal and leguminous plants	30
**Class:** Peronosporomycetidae **Order:** Pythiales	*Pythium awayamai*	Saprophytic and parasitic	Plants	Isolated mainly from monocotyledon plants. Causes snow-rot disease of cereal plants including wheat and barley	30
**Class:** Peronosporomycetidae	*Pythium ultimum*	Saprophytic	Plants	Causes damping-off and root rot to diverse plant hosts including	27

**Order:** Pythiales		and parasitic		crops and forests	
**Class:** Peronosporomycetidae<br>**Order:** Pythiales	*Pythium vexan*	Saprophytic and parasitic	Plants	Causes canker, damping-off and rot disease in many economically important crops including rubber trees, potato and sugar cane	30
**Class:** Saprolegniomycetidae<br>**Order:** Saprolegniales	*Saprolegnia parasitica*	Saprophytic and parasitic	Animals	One of the most important fish pathogens causing millions of dollars of losses to the aquatic culture business worldwide. It attacks a wide variety of fish, amphibians and crustaceans. Members of this genus *Saprolegnia* causes "Saprolegniosis", a disease that is characterized by visible white or grey patches of filamentous mycelium on body of fins of freshwater fish	31
**Class:** Saprolegniomycetidae<br>**Order:** Saprolegniales	*Saprolegnia declina*	Saprophytic and parasitic	Animals	Pathogen of amphibians, fish and insects. Plays a role in decline of natural populations of amphibians. Outbreaks leading to severe reductions and even extinction of amphibians have been attributed to this species. It is also a large problem in fish hatcheries where it infects eggs of salmon and trout	56

Reference:

56. Broad Institute of MIT and Harvard (https://www.broadinstitute.org/).

Table S2. List of databases used to download the whole proteomes of oomycete species for P450 analysis.

**Species**	**Database**
Phytophthora sojae	http://genome.jgi-psf.org/Physo3/Physo3.home.html
Phytophthora parasitica	http://www.broadinstitute.org/annotation/genome/Phytophthoraparasitica/MultiHome.html
Phytophthora ramorum	http://genome.jgi-psf.org/Phyra1_1/Phyra1_1.home.html
Pythium irregular	http://pythium.plantbiology.msu.edu/download.shtml
Pythium iwayamai	http://pythium.plantbiology.msu.edu/download.shtml
Pythium aphanidermatum	http://pythium.plantbiology.msu.edu/
Pythium ultimum	http://pythium.plantbiology.msu.edu/
Pythium vexan	http://pythium.plantbiology.msu.edu/
Phytophthora infestans	http://www.broadinstitute.org/annotation/genome/Saprolegnia_parasitica/GenomesIndex.html
Phytophthora capsici	http://p450.riceblast.snu.ac.kr/species.php?a=intro&spe_id=2785&ref_id=3424
Saprolegnia parasitica CBS 223.65	http://www.broadinstitute.org/annotation/genome/Saprolegnia_parasitica/GenomesIndex.html
Saprolegnia declina VS20	http://www.broadinstitute.org/annotation/genome/Saprolegnia_parasitica/GenomesIndex.html
Hyaloperonospora parasitica	http://www.broadinstitute.org/annotation/genome/Saprolegnia_parasitica/GenomesIndex.html

Table S4. P450 diversity percentage analysis between Oomycota and different fungal phyla.

Fungi			
**Ascomycota**			
**Saccharomycotina**			
**Species name**	**P450 count**	**No. of P450 families**	**P450 diversity percentage**
Saccharomyces cerevisiae	3	3	100
Candida glabrata	3	3	100
Klyveromyces lactis	5	5	100
Kluyveromyces waltii	3	3	100
Pichia anomala	6	6	100
Ashbya gossypii	3	3	100
Dekkera bruxellensis	4	4	100
Pichia pastoris	4	4	100
Candida lusitaniae	8	6	75
Kluyveromyces polysporus	4	3	75
Candida albicans	10	6	60
Candida guillermondii	10	6	60
Candida dublinensis	10	6	60
Pichia stipites	10	6	60
Debaryomyces hansenii	9	5	55.56
Lodderomyces elongisporus	10	5	50
Candida parpsilosis	14	6	42.86
Yarrowia lipolytica	17	6	35.29
Candida tropicalis	21	5	23.81

**Pezizomycotina**			
Neurospora crassa	41	39	95.12
Neurospora crassa	41	39	95.12
Neurospora discreta	43	39	90.7
Coccidioides immitis	40	31	77.5
Uncinocarpus reesii	38	29	76.32
Aspergillus fumigatus	74	56	75.68
Mycosphaerella fijiensis	89	66	74.16
Aspergillus clavatus	92	66	71.74
Thielavia terrestris	70	50	71.43
Fusarium oxysporum	140	100	71.43
Histoplasma capsulatum	47	32	68.09
Fusarium graminearum	109	72	66.06
Aspergillus terreus	124	80	64.52
Myceliophthora thermophila	79	49	62.03
Aspergillus oryzae	142	85	59.86
Aspergillus flavus	162	95	58.64
Aspergillus niger	154	87	56.49
**Basidiomycota**			
Tremella mesenterica	8	7	87.5
Cryptococcus neoformans	8	5	62.5
Serpula lacrymans	159	47	29.56
Phlebiopsis gigantea	127	34	26.77
Agaricus bisporus	115	27	23.48
Phanerochate chrysosporium	149	33	22.15
Postia placenta	190	42	22.11

Ganoderma lucidium	197	42	21.32
Phlebia brevispora	209	42	20.1
Ganoderma sp.	209	41	19.62
Bjerkandera adusta	199	39	19.6
Ceriporiopsis subvermispora	205	32	15.61
Phanerochaete carnosa	266	36	13.53
**Zygomycota**			
Mucor circinelloides	43	16	37.21
Phycomyces blakesleeanus	55	15	27.27
Rhizopus oryzae	53	14	26.42
**Chytridiomycota**			
Batrachochytrium dendrobatidis	9	7	77.78
**Stramenopiles**			
**Oomycota**			
Phytophthora sojae	30	4	13.33
Phytophthora parasitica	31	4	12.90
Phytophthora ramorum	24	4	16.67
Phytophthora infestans	20	3	15.00
Phytophthora capsici	28	3	10.71
Hyaloperonospora arabidopsidis	7	2	28.57
Pythium irregulare	41	3	7.32
Pythium aphanidermatum	31	4	12.90
Pythium ultimum	19	3	15.79
Pythium iwayamai	42	3	7.14
Pythium vexan	21	4	19.05

Saprolegnia parasitica	24	6	25.00
Saprolegnia declina	38	9	23.68
**Measuring average P450 diversity between Oomycota and different fungal phyla**			
	**Average number of P450s**	**Average number of P450 families**	**Average P450 diversity percentage**
Saacharomycotina	8.11	4.79	59
Pezizomycotina	87.35	59.71	68
Basidiomycota	157.00	32.85	21
Zygomycota	50.33	15.00	30
Oomycota	27.38	4.00	15

Table S5. Comparative P450 analysis at family and subfamily level in the 13 oomycete species

		**Peronosporales**						**Pythiales**					**Saprolegniales**		
**Family**	**Subfamily**	***Psoj***	***Ppar***	***Pram***	***Pinf***	***Pcap***	***Hara***	***Pirr***	***Paph***	***Pult***	***Piwa***	***Pvex***	***Spar***	***Sdec***	**Total**
**CYP51**	C												1	1	2
**CYP558**	B													1	3
	C													2	
**CYP5014**	A	1													110
	B		1	1	1	1									
	C	1	1	1		1									
	D	3	4	3	1	3		1	2	3	2	2			
	E	1	1	1		1									
	F	2	2	2	2	2	1		1	1					
	G	1	1	1	1	1									
	H	1	1	1	1	2	1								
	J	1				1									
	K	1	1	1	1	1	1								

	L		2		2	1									
	M							1			1				
	N										1				
	P							2			1				
	Q							1		1	1				
	R								2						
	S							7		1	2				
	T							2		1	1				
	U								1						
	V											1			
	W											1			
	X											2			
	Y											2			
	Z											1			
	AA1											1			
**CYP5015**	A	1	1	1	1	2			1	1	1	1			111
	B	1	1	1								1			

	C	1	1	1	2		1								
	D	1	1	1	1	1			1	1	1	1			
	E	3	4	3	4	4	1								
	F	2	2	2	1	2	1			1					
	G	6	4	2	1	2	1					1			
	H							1		3	2				
	J							4	1						
	K							3			4				
	L							4			7				
	M								1						
	N											3			
	P					1						1			
**CYP5016**	A	1	1	1											3
**CYP5017**	A	2	2	1	1	2		2	1	1	2	1			49
	B									1	1				
	C							1			1				
	D							6			8				

	E							3			1				
	F							1		4	4				
	G							1			1				
	H							1							
**CYP5613**	A													1	1
**CYP5614**	A												2	1	5
	B												1	1	
**CYP5615**	A												5	6	11
**CYP5616**	A												1	1	9
	B												1	2	
	C												2	2	
**CYP5617**	A												2	2	18
	B													1	
	C												1	1	
	D												1	1	
	E												1	1	
	F												1	2	

	G													1	
	H												1	1	
	J													1	
**CYP5618**	A												1	1	8
	B												2	2	
	C												1	1	
**CYP5619**	A													1	6
	B													2	
	C													1	
	D													2	
**CYP5620**	A								**2**						20
	B								**3**						
	C								**1**						
	D								**3**						
	E								**2**						
	F								**1**						
	G								**4**						

	H								**2**						
	J								**2**						
**CYP5621**	A											**1**			**1**
**15**	**84**	30	31	24	20	28	7	41	31	19	42	20	24	39	**356**

Abbreviations: *Psoj, Phytophthora sojae*; *Ppar, Phytophthora parasitica*; *Pram, Phytophthora ramorum*; *Pinf, Phytophthora infestans*; *Pcap, Phytophthora capsici*; *Hara, Hyaloperonospora arabidopsidis*; *Pirr, Pythium irregular*; *Paph, Pythium aphanidermatum*; *Pult, Pythium ultimum*; *Piwa, Pythium iwayamai*; *Pvex, Pythium vexan*; *Spar, Saprolegnia parasitica*; *Sdec, Saprolegnia declina*.

Table S6. Distribution of P450 families in the six oomycete P450 Clades.

Clade	CYP family	Taxonomy
1	CYP5619	Saprolegniales
2	CYP5615	Saprolegniales
3	CYP51, CYP5613, CYP5614	Saprolegniales
4	CYP558, CYP5616, CYP5617	Saprolegniales
5	CYP5618, CYP5017	Saprolegniales, Pythiales and Peronosporales
6	CYP5621, CYP5016, CYP5620, CYP5014, CYP5015	Pythiales and Peronosporales

Table S7. Analysis of sequence identity between oomycete P450s.

CYP name	Species Name	% identity	CYP name	Species name
CYP5015E8	*P. parasitica*	84	CYP5015E6	*P. sojae*
CYP5015D8	*P. parasitica*	81	CYP5015D1	*P. sojae*
CYP5015C5	*P. parasitica*	78	CYP5015C1	*P. ramorum*
CYP5015F4	*P. parasitica*	89	CYP5015F1	*P. sojae*
CYP5016A2	*P. parasitica*	71	CYP5016A1	*P. ramorum*
CYP5017A5	*P. parasitica*	82	CYP5017A2	*P. sojae*
CYP5015A4	*P. parasitica*	87	CYP5015A1	*P. sojae*
CYP5014B3	*P. parasitica*	73	CYP5014B1	*P. ramorum*
CYP5014K3	*P. parasitica*	91	CYP5014K1	*P. ramorum*
CYP5014D14	*P. parasitica*	83	CYP5014D1	*P. ramorum*
CYP5014G3	*P. parasitica*	85	CYP5014G1	*P. ramorum*
CYP5015G15	*P. parasitica*	93	CYP5015G2	*P. sojae*
CYP5015E12	*P. parasitica*	77	CYP5015E7	*P. sojae*
CYP5015G10	*P. parasitica*	83	CYP5015G1	*P. sojae*
CYP5015B2	*P. parasitica*	82	CYP5015B1	*P. sojae*
CYP5014C2	*P. parasitica*	79	CYP5014C1	*P. ramorum*
CYP5015E17	*P. parasitica*	86	CYP5015E1	*P. ramorum*
CYP5017A12	*P. parasitica*	72	CYP5017A2	*P. sojae*
CYP5014H3	*P. parasitica*	86	CYP5014H1	*P. ramorum*
CYP5014D16	*P. parasitica*	80	CYP5014D3	*P. sojae*
CYP5015G11	*P. parasitica*	82	CYP5015G1	*P. sojae*
CYP5015E14	*P. parasitica*	89	CYP5015E2	*P. ramorum*
CYP5014D5	*P. parasitica*	72	CYP5014D2	*P. sojae*
CYP5015G13	*P. parasitica*	89	CYP5015G2	*P. ramorum*
CYP5014F8	*P. parasitica*	92	CYP5014F1	*P. sojae*
CYP5015F6	*P. parasitica*	83	CYP5015F2	*P. ramorum*
CYP5014F3	*P. parasitica*	86	CYP5014F2	*P. ramorum*
CYP5014G2	*P. infestans*	84	CYP5014G1	*P. ramorum*
CYP5015E9	*P. infestans*	84	CYP5015E6	*P. sojae*
CYP5015A3	*P. infestans*	89	CYP5015A1	*P. ramorum*
CYP5017A6	*P. infestans*	79	CYP5017A2	*P. sojae*
CYP5015F8	*P. infestans*	79	CYP5015F2	*P. ramorum*
CYP5014B2	*P. infestans*	70	CYP5014B1	*P. ramorum*
CYP5014D13	*P. infestans*	80	CYP5014D1	*P. ramorum*
CYP5014H2	*P. infestans*	85	CYP5014H1	*P. ramorum*
CYP5014K2	*P. infestans*	91	CYP5014K1	*P. ramorum*
CYP5015D7	*P. infestans*	81	CYP5015D1	*P. sojae*
CYP5015C4	*P. infestans*	81	CYP5015C1	*P. ramorum*
CYP5014F7	*P. infestans*	90	CYP5014F1	*P. sojae*
CYP5014F4	*P. infestans*	87	CYP5014F2	*P. ramorum*
CYP5015D7	*P. infestans*	81	CYP5015D1	*P. sojae*
CYP5015C6	*P. infestans*	80	CYP5015C1	*P. ramorum*
CYP5015E11	*P. infestans*	87	CYP5015E2	*P. ramorum*
CYP5015E18	*P. infestans*	86	CYP5015E1	*P. ramorum*
CYP5015G16	*P. infestans*	92	CYP5015G1	*P. ramorum*
CYP5014F5	*P. capsici*	82	CYP5014F2	*P. ramorum*
CYP5014F9	*P. capsici*	87	CYP5014F1	*P. ramorum*
CYP5015F7	*P. capsici*	79	CYP5015F2	*P. ramorum*
CYP5015G17	*P. capsici*	87	CYP5015G1	*P. ramorum*
CYP5015A6	*P. capsici*	74	CYP5015A1	*P. ramorum*
CYP5015A2	*P. capsici*	89	CYP5015A1	*P. sojae*
CYP5017A4	*P. capsici*	72	CYP5017A2	*P. sojae*
CYP5017A11	*P. capsici*	78	CYP5017A2	*P. sojae*
CYP5015G18	*P. capsici*	81	CYP5015G1	*P. sojae*

CYP5015E15	*P. capsici*	86	CYP5015E4_	*P. sojae*
CYP5015E16	*P. capsici*	83	CYP5015E1	*P. ramorum*
CYP5014C3	*P. capsici*	84	CYP5014C1	*P. ramorum*
CYP5014H5	*P. capsici*	88	CYP5014H1	*P.sojae*
CYP5014K4	*P. capsici*	86	CYP5014K1	*P. ramorum*
CYP5014G4	*P. capsici*	85	CYP5014G1	*P. ramorum*
CYP5015E10	*P. capsici*	77	CYP5015E7	*P.sojae*
CYP5015E20	*P. capsici*	90	CYP5015E4	*P.sojae*
CYP5014D17	*P. capsici*	73	CYP5014D2	*P. ramorum*
CYP5014D15	*P. capsici*	82	CYP5014D1	*P. ramorum*
CYP5014D6	*P. capsici*	76	CYP5014D3	*P. ramorum*
CYP5014B4	*P. capsici*	70	CYP5014B1	*P. ramorum*
CYP5015F5	*P. capsici*	87	CYP5015F1	*P.sojae*
CYP5015E19	*H. parasitica*	84	CYP5015E1	*P. ramorum*
CYP5015F3	*H. parasitica*	86	CYP5015F1	*P. ramorum*
CYP5014F6	*H. parasitica*	74	CYP5014F2	*P. ramorum*
CYP5015C3	*H. parasitica*	72	CYP5015C2P	*P.sojae*
CYP5014H6	*H. parasitica*	74	CYP5014H1	*P. ramorum*
CYP5014K5	*H. parasitica*	83	CYP5014K1	*P. ramorum*
CYP5015G14	*H. parasitica*	88	CYP5015G1	*P. ramorum*
CYP5015F3	*H. parasitica*	86	CYP5015F1	*P.sojae*
CYP5014C1	*P.sojae*	79	CYP5014C1	*P. ramorum*
CYP5014D1	*P.sojae*	81	CYP5014D1	*P. ramorum*
CYP5014D2	*P.sojae*	72	CYP5014D2	*P. ramorum*
CYP5014D3	*P.sojae*	75	CYP5014D3	*P. ramorum*
CYP5014F1	*P.sojae*	89	CYP5014F1	*P. ramorum*
CYP5014G1	*P.sojae*	84	CYP5014G1	*P. ramorum*
CYP5014H1	*P.sojae*	89	CYP5014H1	*P. ramorum*
CYP5014K1	*P.sojae*	86	CYP5014K1	*P. ramorum*
CYP5015A1	*P.sojae*	90	CYP5015A1	*P. ramorum*
CYP5015B1	*P.sojae*	82	CYP5015B1	*P. ramorum*
CYP5015C1	*P.sojae*	82	CYP5015C1	*P. ramorum*
CYP5015D1	*P.sojae*	82	CYP5015D1	*P. ramorum*
CYP5015E4	*P.sojae*	88	CYP5015E2	*P. ramorum*
CYP5015E7	*P.sojae*	77	CYP5015E1	*P. ramorum*
CYP5015E7	*P.sojae*	76	CYP5015E2	*P. ramorum*
CYP5015F1	*P.sojae*	89	CYP5015F1	*P. ramorum*
CYP5015F2	*P.sojae*	84	CYP5015F2	*P. ramorum*
CYP5015G2	*P.sojae*	89	CYP5015G1	*P. ramorum*
CYP5015G3	*P.sojae*	87	CYP5015G2	*P. ramorum*
CYP5016A1	*P.sojae*	74	CYP5016A1	*P. ramorum*
CYP5017A2	*P.sojae*	82	CYP5017A1	*P. ramorum*
CYP5014C1	*P. ramorum*	79	CYP5014C1	*P.sojae*
CYP5014D1	*P. ramorum*	81	CYP5014D1	*P.sojae*
CYP5014D2	*P. ramorum*	72	CYP5014D2	*P.sojae*
CYP5014D3	*P. ramorum*	75	CYP5014D3	*P.sojae*
CYP5014F1	*P. ramorum*	89	CYP5014F1	*P.sojae*
CYP5014F2	*P. ramorum*	86	CYP5014F2	*P.sojae*
CYP5014G1	*P. ramorum*	84	CYP5014G1	*P.sojae*
CYP5014H1	*P. ramorum*	89	CYP5014H1	*P.sojae*
CYP5014K1	*P. ramorum*	86	CYP5014K1	*P.sojae*
CYP5015A1	*P. ramorum*	90	CYP5015A1	*P.sojae*
CYP5015B1	*P. ramorum*	82	CYP5015B1	*P.sojae*
CYP5015C1	*P. ramorum*	82	CYP5015C1	*P.sojae*
CYP5015D1	*P. ramorum*	82	CYP5015D1	*P.sojae*
CYP5015E1	*P. ramorum*	81	CYP5015E4	*P.sojae*
CYP5015E3	*P. ramorum*	75	CYP5015E7	*P.sojae*
CYP5015F1	*P. ramorum*	89	CYP5015F1	*P.sojae*

CYP5015F2	*P. ramorum*	84	CYP5015F2	*P.sojae*
CYP5017A1	*P. ramorum*	78	CYP5017A3	*P.sojae*
CYP5619D1	*S. declina*	76	CYP5619D2	*S. declina*

Table S8. Tandem localization of P450s in oomycetes. Abbreviations: NPTL, number of P450s tandemly localized on the same scaffold; NPTL-SF, NPTL and belongs to the same P450 family.

P. sojae			
**P450 name**	**Scaffold**	**NPTL**	**NPTL-SF**
CYP5015C1	1:4692948-4694507	30	25
CYP5015B1	1:4694621-4696276		
CYP5015D1	1:4696577-4698232		
CYP5016A1	14:1122147-1123784		
CYP5014A1	14:445774-448154		
CYP5015G5	2:2294781-2296295		
CYP5015G6	2:2294781-2296295		
CYP5014H1	3:10425244-10426914		
CYP5014K1	3:10436487-10438012		
CYP5014J1	3:748780-750381		
CYP5014F2	3:752921-754234		
CYP5014F1	3:755754-757340		
CYP5015F2	3:8028502-8029848		
CYP5014C1	4:3153518-3155140		
CYP5014D2	4:3158086-3159726		
CYP5014D1	4:3182143-3183675		
CYP5014D3	4:3187712-3189298		
CYP5014E2	4:5695842-5698363		
CYP5015F1	5:4408528-4410078		
CYP5014G1	5:465521-466667		
CYP5015G1	6:1365977-1367784		
CYP5015G2	6:1373214-1374788		
CYP5015G3	6:1375336-1377079		
CYP5015G4	6:1377256-1378824		
CYP5017A2	6:3343502-3345194		
CYP5017A3	6:3357870-3359688		
CYP5015E7	9:2244328-2245944		
CYP5015E4	9:2372009-2373643		
CYP5015A1	9:370937-372642		
CYP5015E6	9:583115-584585		
P. ramorum			
**P450 name**	**Scaffold**	**NPTL**	**NPTL-SF**
CYP5015G1	11:344098-345675	17	17
CYP5015G2	11:346204-347784		
CYP5014B1	20:181911-183491		
CYP5014D3	20:473485-475095		
CYP5014D1	20:475271-476830		
CYP5014D2	20:482094-483344		
CYP5014C1	20:484076-484498		
CYP5015B1	27:365533-365883		
CYP5015D1	27:386496-388172		
CYP5014F1	36:333016-334587		

CYP5014F2	36:334989-336623		
CYP5014E1	36:393966-395543		
CYP5015E3	41:111359-112975		
CYP5015E2	41:128803-130419		
CYP5015E1	41:135039-135858		
CYP5014H1	6:590429-592072		
CYP5014K1	6:592794-594401		
P. infestans			
**P450 name**	**Supercontig**	**NPTL**	**NPTL-SF**
CYP5014L4	8: 2262001-2263972	11	8
CYP5015A3	8: 2802697-2806822		
CYP5015E18	8: 2811799-2815690		
CYP5014G2	11: 621945-623639		
CYP5015E9	11: 3191768-3193330		
CYP5014D13	30: 1958109-1959675		
CYP5014B2	30: 966101-967645		
CYP5014F7	44: 665896-667467		
CYP5014F4	44: 674710-676035		
CYP5014K2	395: 7452-9416		
CYP5014H2	395: 11045-12942		
P. parasitica			
**P450 name**	**Supercontig**	**NPTL**	**NPTL-SF**
CYP5015E8	18: 245717-247498	24	24
CYP5015A4	18: 325700-327449		
CYP5017A12	3: 2403114-2405127		
CYP5017A5	3: 2488209-2490070		
CYP5014D14	3: 467827-469543		
CYP5014D5	3: 474355-476168		
CYP5014D19	3: 481982-482988		
CYP5014B3	3: 48202-50243		
CYP5014D16	3: 483346-484501		
CYP5014C2	3: 487372-488985		
CYP5015D8	32: 37878-39181		
CYP5015C5	32: 65415-67158		
CYP5014H3	35: 325524-327290		
CYP5014K3	35: 328298-330157		
CYP5015G11	37: 539130-540772		
CYP5015E12	37: 55526-57326		
CYP5015G13	37: 559163-560810		
CYP5015G15	37: 561176-562953		
CYP5015G10	37: 562982-564837		
CYP5015E14	37: 57808-59820		
CYP5015E17	37: 65526-67416		
CYP5014F8	8: 536377-537948		
CYP5014F3	8: 538235-540173		
CYP5014L2	8: 548190-549779		
P.aphanidermatum			
**P450 name**	**Scaffold**	**NPTL**	**NPTL-SF**

CYP5620F1	138:10660 -12225	12	12
CYP5620B2	138:27734 -29498		
CYP5620B3	138:31354 -32908		
CYP5620C1	138:33338 -34882		
CYP5620B1	138:34985 -36583		
CYP5015J4	144:12585 -14159		
CYP5015A8	144:14252-15814		
CYP5620G3	177:3808-5875		
CYP5620G2	177:6038-7554		
CYP5620G1	177:890-1862		
CYP5620A1	864:2604-3659		
CYP5620A2	864:5089-6748		
P.ultimum			
**P450 name**	**Scaffold**	**NPTL**	**NPTL-SF**
CYP5015H3	1117875582023:818634-820244	15	15
CYP5015H7	1117875582023:821985-822842		
CYP5015D4	1117875582023:823909-825393		
CYP5014-fragment1	1117875582028:106159-107640		
CYP5014T1	1117875582028:108463-109667		
CYP5014D11	1117875582028:115894-117111		
CYP5014D10	1117875582028:126097-127350		
CYP5014D18	1117875582028:208033-208944		
CYP5014Q2	1117875582028:343435-344763		
CYP5017A15	1117875582033:20208-21107		
CYP5017F1	1117875582033:29594-31038		
CYP5017B1	1117875582040:330182-331012		
CYP5017F7	1117875582040:351574-353698		
CYP5017F6	1117875582040:372970-373647		
CYP5017F2	1117875582040:376104-377849		
P. vexan			
**P450 name**	**Scaffold**	**NPTL**	**NPTL-SF**
CYP5015N1	1115:1758-3381	11	11
CYP5015N-fragment1	1115:179-1757		
CYP5015N-fragment2	1115:6014-6808		
CYP5015D5	211:25683-27287		
CYP5015B3	211:28383-29954		
CYP5017A10	259:8663-10355		
CYP5014W1	357:8439-10055		
CYP5014AA1	372:7692-9299		
CYP5014D4	40:217-2073		
CYP5014D7	40:3018-4307		
CYP5014Y2	583:15736-17339		
CYP5014Y1	583:259-1835		
CYP5014X1	583:7438-9235		
CYP5014X2	583:9985-11071		
P. capsici			
**P450 name**	**Scaffold**	**NPTL**	**NPTL-SF**
CYP5015A2	10:558729-560373	19	19

CYP5015A6	10:603866-605413		
CYP5015P2	100:54458-56114		
CYP5015G17	100:56638-58218		
CYP5015G18	100:58445-60028		
CYP5014F9	12:307421-309004		
CYP5014F5	12:309235-310566		
CYP5014L1	12:320975-322546		
CYP5014H4	15:665195-666990		
CYP5014K4	15:667766-669567		
CYP5015E10	24:401441-402907		
CYP5015E15	24:411842-413470		
CYP5015E16	24:448260-450227		
CYP5017A4	5:781530-783170		
CYP5017A11	5:794369-795550		
CYP5014C3	55:106837-108386		
CYP5014D17	55:113826-115400		
CYP5014D15	55:130493-132055		
CYP5014D6	55:132132-133691		
S. parasitica			
**P450 name**	**Contig**	**NPTL**	**NPTL-SF**
CYP5618B3	18: 172697-174184	11	10
CYP5615A6	18: 225246-226760		
CYP5615A1	18: 238646-240184		
CYP5615A10	18: 246228-247715		
CYP5618B4	18: 375093-376574		
CYP5616C3	35: 144918-146460		
CYP5616C1	35: 146781-148300		
CYP5617E2	8: 303172-305005		
CYP5617D2	8: 305455-306983		
CYP5617C1	8: 312501-314611		
CYP5616A2	8: 509567-511081		
S. declina			
**P450 name**	**Supercontig**	**NPTL**	**NPTL-SF**
CYP5618B1	14: 453794-455275	26	26
CYP5615A11	14: 587491-589118		
CYP5615A2	14: 591523-593236		
CYP5615A7	14: 604570-606323		
CYP5618B2	14: 657390-658877		
CYP5617H1	18: 35461-37977		
CYP5617G1	18: 549369-551291		
CYP558C1	3: 1038264-1039523		
CYP558B1	3: 1235049-1236631		
CYP5616C4	30: 149571-151248		
CYP5616C2	30: 151524-153132		
CYP5617A4	32: 525178-527253		
CYP5617A1	32: 527419-529373		
CYP5616B3	67: 111142-112094		
CYP5616A1	67: 113069-114553		

CYP5619C1	69: 202902-205979		
CYP5619B2	69: 220501-222939		
CYP5619D2	69: 227112-230164		
CYP5619A1	69: 230636-233888		
CYP5619B1	69: 234432-237510		
CYP5617C2	7: 166635-168753		
CYP5617B1	7: 169094-171218		
CYP5617D1	7: 172665-174636		
CYP5617E1	7: 174992-177017		
CYP5617F3	7: 890360-892347		
CYP5617F1	7: 904925-906951		
**Species name**	**P450 count**	**% NPTL**	**% NPTL-SF**
P. sojae	30	100	83
P. ramorum	24	71	71
P. infestans	20	55	40
P. parasitica	31	77	77
P.aphanidermatum	31	39	39
P.ultimum	19	79	79
P. vexan	21	52	52
P. capsici	28	68	68
S. parasitica	24	46	42
S. declina	39	67	67

Table S9. List of P450s used to deduce amino acid combinations at EXXR and CXG motifs in P450 families, CYP5014, CYP5015 and CYP5017. In the list, the P450s from the same family and subfamily belong to *P. sojae* and *P. ramorum*. The number in the parenthesis indicates number of P450s used to deduce the amino acid combinations for a family.

**CYP5014 (89)**	**CYP5015 (84)**	**CYP5017 (38)**
CYP5014	CYP5015A1	CYP5015E4
CYP5014AA1	CYP5015A1	CYP5015E6
CYP5014B1	CYP5015A2	CYP5015E7
CYP5014B2	CYP5015A3	CYP5017A1
CYP5014B3	CYP5015A4	CYP5017A10
CYP5014B4	CYP5015A5	CYP5017A11
CYP5014C1	CYP5015A6	CYP5017A12
CYP5014C1	CYP5015A7	CYP5017A13
CYP5014C2	CYP5015A8	CYP5017A14
CYP5014C3	CYP5015B1	CYP5017A15
CYP5014D1	CYP5015B2	CYP5017A3
CYP5014D1	CYP5015B3	CYP5017A4
CYP5014D10	CYP5015C1	CYP5017A5
CYP5014D11	CYP5015C3	CYP5017A6
CYP5014D12	CYP5015C4	CYP5017A8
CYP5014D13	CYP5015C5	CYP5017B1
CYP5014D14	CYP5015D1	CYP5017B2
CYP5014D15	CYP5015D1	CYP5017C1
CYP5014D16	CYP5015D2	CYP5017C2
CYP5014D17	CYP5015D3	CYP5017D10
CYP5014D18	CYP5015D4	CYP5017D11
CYP5014D2	CYP5015D5	CYP5017D14
CYP5014D2	CYP5015D7	CYP5017D2
CYP5014D3	CYP5015E1	CYP5017D3
CYP5014D3	CYP5015E10	CYP5017D4
CYP5014D4	CYP5015E11	CYP5017D5
CYP5014D5	CYP5015E12	CYP5017D6
CYP5014D6	CYP5015E13	CYP5017D9
CYP5014D7	CYP5015E14	CYP5017E4
CYP5014D8	CYP5015E15	CYP5017F1
CYP5014D9	CYP5015E17	CYP5017F2
CYP5014E1	CYP5015E18	CYP5017F4
CYP5014E4	CYP5015E19	CYP5017F5
CYP5014E5	CYP5015E2	CYP5017F6
CYP5014F1	CYP5015E20	CYP5017F7
CYP5014F1	CYP5015E3	CYP5017F8
CYP5014F2	CYP5015E8	CYP5017F9
CYP5014F2	CYP5015E9	CYP5017G1
CYP5014F3	CYP5015F1	
CYP5014F4	CYP5015F1	
CYP5014F5	CYP5015F2	
CYP5014F6	CYP5015F2	

CYP5014F7	CYP5015F3	
CYP5014F8	CYP5015F4	
CYP5014F9	CYP5015F5	
CYP5014G1	CYP5015F6	
CYP5014G2	CYP5015F7	
CYP5014G3	CYP5015F8	
CYP5014H1	CYP5015G1	
CYP5014H1	CYP5015G1	
CYP5014H2	CYP5015G10	
CYP5014H3	CYP5015G11	
CYP5014H4	CYP5015G12	
CYP5014H6	CYP5015G13	
CYP5014J1	CYP5015G14	
CYP5014J2	CYP5015G15	
CYP5014K1	CYP5015G16	
CYP5014K1	CYP5015G2	
CYP5014K2	CYP5015H1	
CYP5014K3	CYP5015H3	
CYP5014K4	CYP5015H4	
CYP5014K5	CYP5015H5	
CYP5014L1	CYP5015J1	
CYP5014L2	CYP5015J2	
CYP5014L3	CYP5015J3	
CYP5014L5	CYP5015J4	
CYP5014M1	CYP5015K1	
CYP5014M2	CYP5015K2	
CYP5014N1	CYP5015K4	
CYP5014P	CYP5015K5	
CYP5014P1	CYP5015K6	
CYP5014Q1	CYP5015K7	
CYP5014R	CYP5015L	
CYP5014R2	CYP5015L1	
CYP5014S1	CYP5015L2	
CYP5014S10	CYP5015L4	
CYP5014S2	CYP5015L5	
CYP5014S3	CYP5015L6	
CYP5014S4	CYP5015L7	
CYP5014S5	CYP5015M1	
CYP5014S6	CYP5015N	
CYP5014S7	CYP5015N1	
CYP5014S9	CYP5015P1	
CYP5014T2	CYP5015P2	
CYP5014T3		
CYP5014U1		
CYP5014V1		
CYP5014W1		
CYP5014Z1		

Table S10. Comparative analysis and structural analysis of P450 fused proteins in Oomycota and different fungal phyla. Heme peroxidase/dioxygenase and P450 domains in the P450 fused proteins were identified as described in 'Methods'. The size of each of the domain is presented in the table.

CYP NAME	PROTEIN ID	SPECIES NAME	Protein size (AA)	N-terminal to C-terminal	
				Heme peroxidase/dioxygenase	P450
CYP6001A4	EGP91582.1	*Mycosphaerella graminicola*	1113	96-664	941-1058
CYP6004A1	EGP86918.1	*Mycosphaerella graminicola*	939	88-536	727-916
CYP6004A8	CBX98513.1	*Leptosphaeria maculans* JN3	1099	88-638	635-1040
CYP6001A12	CBX92964.1	*Leptosphaeria maculans* JN3	1134	135-696	958-1088
CYP6001C13	AFL2G_11739	*Aspergillus flavus* NRRL3357	1139	144-694	661-1092
CYP6003B1	AFL2G_03725	*Aspergillus flavus* NRRL3357	1419	228-784	1032-1173
CYP6001A1	Afu4g10770(PpoA)	*Aspergillus fumigatus*	1079	98-647	909-1050
CYP6001C4	Afu4g00180(ppoB)	*Aspergillus fumigatus*	1136	141-691	686-1089
CYP6001A1	AN1967 (ppoA)	*Aspergillu nidulans* FGSC	1081	98-647	654-1050
CYP6002A1	AN6320 (ppoB)	*Aspergillu nidulans* FGSC	997	22-546	828-971
CYP6001A18	CAP97986.1	*Penicillium chrysogenum* Wisconsin 54-1255	1074	93-642	900-1031
CYP6001C24	CAP94248.1	*Penicillium chrysogenum* Wisconsin 54-1255	1118	145-695	704-1068
CYP6001C5	EDN21247.1	*Botryotinia fuckeliana* B05.10	1128	131-685	957-1085
CYP6001A19	EEH08229.1	*Ajellomyces capsulatus* G186AR	1084	134-652	659-1055
CYP6001A20	EEH08179.1	*Ajellomyces capsulatus* G186AR	1084	134-652	659-1055
CYP6003C1	EEH05495.1	*Ajellomyces capsulatus* G186AR	1138	109-674	649-1049
CYP6001A21	XP_002795155.1	*Paracoccidioides brasiliensis* Pb01	1066	160-634	624-1035
CYP6001C25	XP_002794393.1	*Paracoccidioides brasiliensis* Pb01	1059	88-641	890-1028
CYP6003C2	XP_002793903.1	*Paracoccidioides brasiliensis* Pb01	1121	109-651	649-1054
CYP6004A13	EGX44933.1	*Arthrobotrys oligospora* ATCC24927	1062	88-638	641-1015
CYP6001D1	EGX44932.1	*Arthrobotrys oligospora* ATCC24927	1138	155-706	967-1100

CYP6001E1	XP_002839073.1	*Tuber melanosporum* Mel28	1079	86-646	655-1052
CYP6001C26	XP_002835452.1	*Tuber melanosporum* Mel28	1119	128-691	979-1065
CYP6001C27	EGR45174.1	*Trichoderma reesei* QM6a	1046	79-633	886-1045
CYP6001C28	EHA53428.1	*Magnaporthe oryzae* 70-15	1153	170-726	1000-1128
CYP6005J1	140487	*Coniophora puteana*	1051	100-650	866-959
CYP6005A6	83601	*Coniophora puteana*	1067	99-649	847-1010
CYP6005A7	416857	*Serpula lacrymans*	1061	97-637	858-1018
CYP6005K1	414828	*Serpula lacrymans*	1035	100-646	815-1012
CYP6005A1	108647	*Ganoderma* sp	1054	88-636	644-988
CYP6005B1	114256	*Ganoderma* sp.	1102	116-689	Not detected
CYP6005A2	167776	*Bjerkandera adusta*	1061	89-643	599-1024
CYP6005F1	189372	*Bjerkandera adusta*	1088	113-669	936-1015
CYP6005A4	92254	*Phlebia brevispora*	996	21-568	524-939
CYP6005J1	25609	*Phlebia brevispora*	967	86-650	Not detected
CYP6005H1	28312	*Phlebia brevispora*	1192	232-761	769-1153
CYP6005A8	264492	*Phanerochaete carnosa*	1050	84-634	912-1013
CYP6005H2	112761	*Phanerochaete carnosa*	990	29-546	828-946
CYP6005A9	260261	*Phanerochaete carnosa*	1050	84-634	912-1013
CYP6005A10	134167	*Wolfiporia cocos*	1061	90-643	791-1024
CYP6005A11	64780	*Trametes versicolor*	1052	82-632	640-986
CYP6005B2	157667	*Trametes versicolor*	1102	110-691	Not detected
CYP6005G2	107209	*Punctularia strigosoznata*	1070	101-670	875-998
CYP6005L1	146616	*Punctularia strigosoznata*	1155	200-742	734-1084
CYP6005A12	99109	*Fomitopsis pinicola*	1055	86-639	648-993
CYP6005H3	95975	*Fomitopsis pinicola*	1093	119-671	817-1054
CYP6005H4	1052690	*Fomitopsis pinicola*	1111	119-671	864-1071
CYP6005H5	161482	*Fomitiporia mediterranea*	1094	114-662	676-1032
CYP6005A13	15962	*Fomitiporia mediterranea*	1057	90-646	866-1019
CYP6005M1	143643	*Agaricus bisporus var bisporus*	1057	92-609	821-957
CYP6005N1	21425	*Dacryopinax* sp.	1129	132-688	696-1075
CYP6005A14	80992	*Dichomitus squalens*	1060	91-643	616-994

CYP6005B3	131684	*Dichomitus squalens*	1099	120-687	737-1035
CYP6005A15	112882	*Ceriporiopsis subvermispora*	1058	84-639	910-1056
CYP6005A1	GL07378-P1.1	*Ganoderma lucidum*	1006	38-585	558-940
CYP6005B1	GL01669-P1.1	*Ganoderma lucidum*	1101	116-688	738-1022
CYP6005A16	131231	*Stereum hirsutum*	1037	42-584	835-992
CYP6005G3	98016	*Stereum hirsutum*	1089	107-671	664-999
CYP6005K2	171396	*Stereum hirsutum*	1092	112-658	654-1043
CYP6005P1	1278626	*Auricularia subglabra*	1166	197-730	747-1094
CYP6005Q1	1412406	*Auricularia subglabra*	1215	200-795	795-1177
CYP5619A1	SDRG_14280.1	*Saprolegnia declina*	970	693-831	15-432
CYP5619D2	SDRG_14279.1	*Saprolegnia declina*	968	667-863	32-370
CYP5619C1	SDRG_14273.1	*Saprolegnia declina*	988	710-883	87-435
CYP5619D1	SDRG_03324.1	*Saprolegnia declina*	971	693-962	44-371
CYP5619B1	SDRG_14281.1	*Saprolegnia declina*	997	696-892	30-453
CYP5619B2	SDRG_14277.1	*Saprolegnia declina*	795	685-784	35-784
