# Supplementary material for: Diversity and evolution of cytochrome P450 monooxygenases in Oomycetes
Source: Sci Rep. 2015 Jul 1;5:11572. doi: 10.1038/srep11572 (PMC4486971; doi:10.1038/srep11572)
Supplement: Supplementary Information [file srep11572-s2.pdf]

Diversity and evolution of cytochrome P450 monooxygenases in Oomycetes

Mopeli Marshal Sello[1], Norventia Jafta[1], David R Nelson[2], Wanping Chen[3], Jae-Hyuk Yu[4], Mohammad Parvez[1], Ipeleng Kopano Rosinah Kgosiemang[1], Richie Monyaki[1], Seiso Caiphus Raselemane[1], Lehlohonolo Benedict Qhanya[1], Ntsane Trevor Mthakathi[1], Samson Sitheni Mashele[1*], Khajamohiddin Syed[1*]

* Corresponding authors email: khajamohiddinsyed@gmail.com & smashele@cut.ac.za

Table S3. Annotation of Oomycete P450s. P450s belong to *P. sojae* and *P. ramorum* were resourced form public available database[35].

>CYP5014A1 P. sojae

MLAFLPTLAASDSRQNVVTSALIALLLGASTYATLSHIERSRAKHQRHKEGLPVPRPSTTLPIMGNTLDFVKNN
DVFHDCVSSLVQEFNGEPFLLSAPGRPDILVVSTPEAFEGVTKRQFDTFVKGDLHEMFYDLLGNALTNSDGDVWQFQR
KIFAKLFSARALRESMTSTIQKHGRTMHTLFENGAASGASFDLFRLLSRFAMESFAEIGFGIQMGSLAIGEDHPFEKAFDI
AEEATAKRFSVPAWFWKLQRLLSVGSEGQLQRAIQVIDSTVLKFIYESIAGRARDEKRTGGAQNIVSLALDSCDLEGEAD
PQLLRSIAIAAIIAGRDTTSETLSWFFYTLSQHPEVERNIRTEMLERIPRLVLETGYFPAMDEVQSLTYLEAAIKETLRLYPPA
SFNIKHCSADIFLSDGTFIPEGTTIGLPSYAMGRMTSTWGPDCNEYKPERFLDPDTGKLLSVSPFQFPAFFAGLRICVGM
NLAMLEMKIVLTGLLSRFLSRLQTGFDIVASTFQLVHDLGLRCPPVLQVRRVGGVHVNLHAPQFFHNRSVKHPSANAFV
VKETLSGFSRSPSLITHVNTFNSAAPSELVFVFLQTLALPV

>CYP5014AA1 P.vexan

MKVLALPEVAPARAALVASSVLLSLVLLRALTASKTAEPSKPKTKDGAPLFEPPSAGRVLGSMLEIARNAGRFH
DWIGDVTALAGGKPWLLRALGRPDIVVVSTPELFEDVQKTQFDSFGKGPYVHELTRDLAGNAIVLVDGPLWAFQRKVS
VNLFSARLLRESMASVIHKNTLTLDRILDAHAQSGGELFDLSRLMYQFTMEVFTEIGFGLQLGALGNSKSHAFESALDEA
TVAVSKRSRLPTPVWKLMRALNVGSERELRECIKIIDENVMGIIAKTMEAHQRGDAPGRADLVSLFLEHSASEKGDYTV
DAKLLKDIALNMLVAGRDTSAEAMCWFLYCLSTRPDVEAKVRAEILEKLPELASGATPFVPVDALQGLVYLEAALKESLR
LFPLFAFVRRVAFEDVELSDGTFVPQGTYVAMAPFTMARRTDVWGEDAAEYNPERFLDPATGKLRQISIFKFNSFWAG
PRSCVGMKLAMLEMKTVLAKVLARFRFEIDPANQPDGRVTYRMSLTLPIKDGLRVRVARLPTDATVA*

>CYP5014B1 P. ramorum

MSSVDPTSALLYAASCSLALLVGAKVLFPEPKRAARHPDSLPLLGETWAALKYADEYYDWEAAMTEKMEGRP
WLFDVVGRPSEFVIGKPEIIEDVLRTHAESFGKGEYVHEVLSGLLGDGIIAVDGHKWARQRKTASNLFSLRELRESMATV
VQDNVLTLNGIFQHAMDRGESLDLFQLLNRFTFEVIAEIAFGIKFGGLATGSKHPLEAAFNCAQQRMFMRFLEPTWW
WKLQRWLNVGPEGAFKKQVQIIDETCYSIISRSMKERKAKRSSHDADTLEGSASTQRQKSNIISLFLDGVSDDEAKAGD
GLDPKFLRDIVVTFMTAGRDTTASALSWFFYTLSQHPQTEEKIRQEMASKLPELANGAVSSPSMLQANELVYLEAALKE
TLRLYPAVPTNIREALEDVVLCDGTVVKAGETVSWSTYALGRMPHVWGEDAKEFKPERWVDADGKLIAVSPFKYPLFS
AGPRVCLGGKLALMEMKITAASVLSKYHFTIVPGQNVTYRIGLTFGMKNGLHVKVSEAAPAI*

>CYP5014B2 P.infestans

SNVDKNQLVLYAASCSLAIWVGSKILFSEPQRAARHRESLPLIGETWAAIKHAEHYYDWEAEMTEKMEGRPW
MFNVVGRPTEFAIGKPEIIEDVLSTQFKSFGKGEYVREVLSDLIGDGVFAVDGHKWMQQRKTASNLFSTRELRGSMTTC
ALEKGETVDLFRLLNRFTFEVISEIAFGIKLGGLRLESEHPVETAFNNAQQRLCERFLEPTWLWKLQRWLNERELKENIQII
DSTCYDIISRSMKKRQVSGSAATGGKRNIISLFLDGVSDDAKSDQGLDPKYLRDIVVSFMTAGRDSTASALSWFFYTVSQ
HPEVEENIREEIFSKVPELANGTISAPSAAQAKELVYLDAAVKEVLRLYPAVPSNIREALEDVVLCDGAVVKAGETVSWSS
YAMGRMPQVWGPDAKEFKPERWIDASTGKLAAVSPFKYPIFNAGPRSCLGSKLVMMEIKITAASVLSKYNLTVAPQQT
VAYKIGLSLAMKNGLQVKVKKVAHASY*

>CYP5014B3 P.parasitica

MSNVDVNLVVLYAASCSLALWVATRILSPERKRAARHRDSLPLLGETWAAIKHAEHYYDWEAEMTEKMEGR
PWMFDVVGRPTEFVVGRPEIIEDILLTQFKSFGKGEYVHEVLNDLIGDGIFSVDGHKWMRQRKTASNLFSMRELRESM

ATIVQENANTLNGILECTMSKSESLDLFHLLNRFTFEVISEIAFGIKFGGLLSESEHPVETAFNCAQQRMFERFLEPTWLW
KLQRWLDIGSERKLKKNVQIIDNTCYNIISRSIEERHTMGSTASGSKRNIISLFLDGVSDGAKIEQDLDPKFLRDIVVSFMT
AGRDSTTAALSWVFYTVSQYPEVEKKIREEIISKVPELASGAIASPSATQANELVYLEAVVKEVLRLNPAVPSNIREALEDV
VLCDGTIVKAGEAVSWSSYSLGRMPHIWGLDAKEFKPERWIDATRGKLVTVSPCKYPLFNAGPRSCLGTKLAMMEIKIT
AASVLSKYHLTVVPGQTITYRIGLSLAMKNGLNIKVEKVNRPSC*

>CYP5014B4 Phytophthora capsici

MDTTSTMLYAVSCSLALWVGSKLLIPEPKRAARHRESLPLLGETYAAIKHADHYYDWEAQWTEKMEGRPWIF
HVVGRPVEFVIGRPEIIEDVLVTQFKSFGKGEYVHQVLSDLLGDGIFAVDGHKWMQQRKTTSNLFSMRELRESMATVI
QENVHTLNEILQRAMETRESVELFRLFNRFTFETISEIAFGVKFGGLATEKEHPVETAFNYAQQRLFERFLEPTWLWKLQ
RWLHVGGERELKAHLEIIDKTCYDIISRSINERHLGDIRDGHKRNIISLVLDGASDDEAKEPDPKYLRDIVVSFMTAGRDST
TAALTWFFYAVSQHPKVESKIRQEITAKIPELMSGDIPSPSLAQVNELVYLEATVKESLRLYPAVPSNIREALEDIVLCDGTV
VKAGEAVSWSSYSMGRMPHVWGPDAKEFKPERWIDPDTGKLVAVSPYKFTLFNAGPRSCLGTKLAMMEVKITAASV
LSKYHLTVVSGQDISYRLGLSLAMKNGLNINVEAISAVSS*

>CYP5014C1 P. ramorum

MLSIASLKLEHPLYHALTVTSIVLLPIVFQLSRGSSSSSTDDEERADSELERRDADRPPWTLPVLHNTLNWILGG
DGIHEWITRNCERFKGRPFTVKALGLPEMLVVSTPEAFEDVLKNQFMNFPKGPHVKENLQDLLGDGIFAADGVKWAH
QRDVARGLFRMRELRDCMTEAITRHTKALHDVLGKVCARNRSVDLHKLLSCFSTEAFAEISFGMKMGCLRANKELPFQ
AAFDSAQRLTAQRFVRPRWFWKMQRRLGLGAEDQLQLDIKEIDAAVLNIVQRVLSQRALVPDDGAPKSTNMLSLFLD
TIAKSPKAEEQLYDPAYLRDVVVNFLVAGRDTTAQALSWFFYNVSQNPHVEAKLRREIYKKLPELVNSEVCVPTLQQVN
RLVYLEAVMKETLRLYPPVPMSPKYAVRDAMLSDGTFVAAGSMVCLPMYAMGRMPHVWGPDAAEFNPERWIDPA
TKKIVSVSAFKFVAFNAGPRMCLGTTLAGLELKLVAASLLSRFHIHVENPEDVTHEFSLTLPVKGPMNVRLARVQAAVA*

>CYP5014C1 P. sojae

MLSVSALKLETPLHHALAVTSFLLLPLVIQLSRRIGSSSAETPEAFKERADSEPERREAGRPPWTLPVLHNTLGFL
LAGNNLHEWITRTCERFEGNPFTVKVLGLPRMLVVSTPEAFEDVLKYQFMNFPKGPQYSENMKDLLGDGLFAADGVK
WAHQRDIAHGLFRTKELRECMVKAITRHTMALHDVLKQICARNRSVDLYKLLSCFSTEAFADISFGLKMDCLRANKELP
FQAAFDRAQRLTALRFVRPRWFWKMQRRLGLGAEDQLQLDIKEIDATVLSIVQRVLAQRAMAPEDKDSNMLSLYLDA
IARSSGTDEQLYDPVHLRDVVVNFLVAGRDTTAQALSWFFFCVSQNPRVESKLRREIYKKLPELMTAESCVPTLEQVNKL
VYLEAVIKETLRLYPSMPIAPKYAVRDTVLSDGTFVAAGSMVCLPLYAMGRMPHAWGPDAAEFKPERWVDPVTKKITS
VSAFKFVAFNGGPRMCLGSSLAGLELKLVAAALLSRFHIYVENPEDVGFGFSLTLPVKGPMNARLARVSASFG*

>CYP5014C2 P.parasitica

MLSVSALKLEHPLFNALAVSSLLLLPVVLQLTRRSNSSSLDSKERTGSERREAARPPWTLPVLHNTLQLLLARNG
IHEWITTNCAQFGGRPFTVKALGLPEMLVVSTAQALEDVLKHQFMNFPKGPHVKENMKDLLGDGIFAVDGVKWAHQ
RDVARELFRTQELRECMTEAITRHTMALHDVLKKVCARNRSVNLYKLLSCFSTEVFTDISFGVTLNCLRANKELPFQAAF
DRAQRLTAQRFVRPRWFWKMQQRLGLGSEDQLQLDIKEIDATVMSIVQQVLVNRALTPEDGKAKNLSMLSLFLDILAK
SPKTKEQQYDPVYLRDVVVNFLVAGRDTTAQALSWFFYNVSQNPRVENKLRREIYKKLPELITAECCVPTLQQVNRLVYL
EAVIKETLRLYPSMPISPKYAVRDTVLSDGTFVSAGTMVCLPLFAMGRMTHVWGPDVAEFKPERWINPSTKTIISVSAFK
FVAFNAGPRMCIGSSLASLELKLVAASLLSRFHIHVENPDTVSYDFSLTLPVKGPMNVRLARVSAGFA*

>CYP5014C3 Phytophthora capsici

MLVVSTPQAFEDVLKHQFMNFPKGPHLKENMQDLLGDGIFAVDGVKWAHERDVARGLFRMQELRECMT
QAITRHTMALHDVLKQVCARNRSVDLYKLLSCFSTEVFTDISFGVSMNCLRANQELPFQAAFDRAQRLTALRFVRPRWF
WKMQKRLGLGAEDQLQLDIKEIDATVLSIVQQVLVNRALTPEDGRGKNLSMLSLFLDTVDRSPKAEEKLYDPVYLRDVV
VNFLVSGRDTTAQALSWFFFNVSQNPQVETKLRREIYKKLPELMTSECCVPTLQQVNRLVYLEAVIKETLRLYPSMPISSK
YAVRDTVLSDGTFVRAGTMVCLPLYAMGRMPHVWGPDAAEFKPERWIDPSTKKIISISAFKFVAFNAGPRMCLGTSLA
GLELKLVAAALLSRFHIHVEKPEAVDYDFSLTLPVKGPMNVRLARVSAGFA

>CYP5014D1 P. sojae

MTDKLSSSVAVAALSGLVVLPLARLLHVDKDKSQLSTRKVVRPATTLPVLGNTLDVIKNLPIRCDWLTSLCQDA
QGEPVLLQSLGTPDTTLLSTPQAFEDVFKNQFDNFPKGPKKSEYLCELLREGIFAVDNEKWYRQRKTASNLFTMRALRD
SMTSTIQRHLVVLDRIFNRAAETDDTLDLFRLLNRFTMEAFTEIGFGVHMNWLDSDKEHPFQTAFDQSQQLLVLRFVRP
SWFWKAQRMMGVGAEGQLQRELHVIHSTIFDIVAQNLQNRAKGENDKAGMDIVSLFLDDLNRSGDADESCFDPTYL
RDIVVNFIIAGRDTTAQALSWFFYCLSHNPQVETKIRKELRAKLPRLFSGDCSPSMDEVSELTYVEAALRETLRLYPSVPIV
NKEAVHDTVLSDGTFIAAGTVAALPMYALGRMTHFWGPDAAEFKPERWIDAQAGKLISALAFKFVAFNAGPRLCLGK
NLAMLEMKLIVASLLSKYRVELERPEDVTYAISKDLLVGESP*

>CYP5014D1 P. ramorum

MTDKLSSSVAVAALSGLVTLPLAWYLLSTAHGEKQLGTRKVVRPSTTKPLIGNTLDILYNLPIRHDWITSLCEEA
KGEPVLLQSLGTPDMTLLSTPGAFEDVFKNQFDNFPKGPKKSEYLRELLGEGIFAVDNEKWYRQRKTASNLFTMRALRD
SMTSTIQRHLVVLERIFRRAAETNASIDMFRLLNRFTMEAFTEIGFGVEMNCLDSDQEHPFQTAFDRSQQSLALRFVRP
SWFWKTQRMLGLGPEGQLQQDMKVINSTICDIVAKTLQNSARGAPKPDDKAAMDIVSLFLDDLNKSSDVDANCFDP
TYLRDIVVNFIIAGRDTTAQALSWFFYCLSQNKQVETKIREELLAKLPDLFNGQCSPSMDAVGELTYVEAALRETLRLYPS
VPIVSKQAVQDAVLSDGTFIAAGAMAGLPMYALGRMPHVWGPDAADFKPERWVDAQTGKLISVSAYQFVAFNAGP
RLCLGKNLAMLEMKLIVASLLSKYHVELETPKTVTYAISFTLPVKGQLNAKISAV*

>CYP5014D10 P.ultimum

MLSVGSLLELNATSVLLGILSLQVLAFAYHALSTAKAFEDVFKHQFACFPKGPFFIDNLKDLLGEGIFAVDDAKW
VHQRKTASHLFTMRTLRDSMTTTVQTHAKVLHTILQRAVDSNTPVDLFKLFNRFTIEAFAEIGFGIHLGCLDAEEEHPFQ
TAFDSAQREVILRFTRPGWFWKTQRRLGIGAEGMLKNNLKTIDDTVLDIVAKSLERRQASRGLAKGETGKDIVSLFLDN
VSQNPDFEAKEFDPKYLRDIVVNFLIAGRDTTAQALSWFFYNLSSHPEVVAKIRAEITAKLPELASGKIVTPTMGQVQDL
VYLEAALRETLRLYPSVPFETKHVTHDIVLSDGTFIRGDTTVGLPFYGMGRMTSVWGSDAEVFNPDRWIDAQTEKIINV
SAYKFVAFSAGPRMCLGMNLAMLEMKLVVSGILTKFDVELLPGQEITYDFSLTLPLLPLLAFDLRMCFRLVDELKSFK*

>CYP5014D11 P.ultimum

MLSIGQLIFAGAALSFVAVVASISILVLVLVLVYGKMVTAKSKAPRFETLDNNSRKVWRLDTAIPFFEDTLELAKHV
DDFQDYTASHVKQFNGEPYIVKVLGRPDSTGVRELLGEGIFGVDDAKWVRQRKTASNLFTQRALRDSMTTIVQTHAKV
LHSILQRSADNKTSIDLFRLSNRFSIEVFSQIVFGIRLGCLDADEEHPFQTTFDSAQRQIVLRFARPSWFWKAQWWLGVG
AEGILKENIKRIDNMVLEIIATSLQHRHNNDALTTQQQNREKDIVSLFLDNVSQNLDFEAKDFDPRYLRDIVVNFLIAGRD
TTAQSLSWLFYNLSSRPDVVAKVRAEIATRLPAFVHGEVTEISMEQSKELVYLEACLKESLRLHPTLSFGTKHVKHDVVLS
DGTFIKTNTTVGLAFYGMARLERVWGPDAESFSPDRWIDPDTDKLISVSSYKFTSFSAGPRMCLGMNLAMLEMKLVM
SGLLSKFDVELVPGQKITHDFSLTLPVRGAMMANVTCAW*

>CYP5014D12 P.aphanidermatum

VLQSFFQGGANVLTGDSTVLGLAVLAAAVLPVVSWWAFRSFVGEKTTLQGTRTIHRLKTTLPLLGNTLDMLG
NVGRFQEWIYDICKEYRGEPVLITSLGRPDLLVMCTTEAFEECLKTKFENFPKGPFQCENLEGILGNGIFAVDHAKWVHQ
RKTASNLFTARTIRDGMTKVVRKHTVTLRRMLQRASATGEQVDLFKLFNRLTIEAFAEIGFGIEMNCLEAKEEHPFQTAF
DSAQRALVLRFVRPSWFWKFQRMLGIGVEGTLQRDTRAIDKTVLDIIAKSFEKRQSGAASQRHEGVGTDIVSLFLDNFE
NSADSKDNAFDPKYLRDIVVNFLIAGRDTTAQALSWFFLSISRNPRVVTKIREEIAQVLPGLMDGTIETPSMEEVQQLTYL
EAALKETLRLYPSVPFTTKAVENDIVLSDGTFIRKGSYIGLPSYSLGRMTHVWGEDATEFKPERWIDEQGKIHQFPPSKFV
AFNAGPRLCLGINLAMMEMKIVVASLLSQLNVTVLPDLNVTHDFSLTLPVHGELLATVTKA*

>CYP5014D13 P.infestans

LTIDRMTDKLSSSIAIAALSGLVVLPLAWRLLVAYDNKTQRGSRKVVRPSTTKSLLGNTLDVIGNVPIRHDWITG
LCLEAKGEPVLLQSLGTPDMTLLSTPQAFEDVLKNQFDNFPKGPKKAEYLRELLGEGIFAVDHEKWYRQRKTASNLFTM
RSLRDSMTSTIQRHLVVLEQIFHRAAETNDTVDMFRLLNRFTMEAFTEIGFGVHMNCLDAEKEHPFQTAFDRSQQLFIL
RFVRPSWFWKLQRFLGVGAEGQVKKDMEVINSTIFDIVAQTLEHRAKGTQDDKGGKDIVSLFLDDLNRSGDADESSFD
PTYLRDIVINFIIAGRDTTAQALSWFFYCLSKNPEAETKIREEVAAKLPKLLNGQCSPSMDELGELVYVEAALRETLRLYPSV
PIVSKEAVHDTVLSDGTFIGAGTLAGLPMYALGRMPHVWGPDAAEFKPERWIEAGKLISVSAYQFVAFNAGPRLCLGK
NLAMLEMKLIVADVTYAISFTLPVKGQLNAKISAV*

>CYP5014D14 P.parasitica

MLTIDRMTDKLSSSIAIAALSGLVVLPLAWRLLVASNTNSKSQHGTRKVLRPSTTKPLLGNTLDVIGNVPIRHD
WIANLCLEAKGEPVLLQSLGTPDMTLLSTPQAFEDVFKNQFDNFPKGPKKAEYLRELLGEGIFAVDYEKWHRQRKTASN
LFTMRALRDSMTSTIQRHLVVLERIFHRAAETNKTVDMFRLLNRFTMEAFTEIGFGVHMNCLDAAEEHPFQTAFDRSQ
QLFVLRFVRPGWFWKLQRFLGIGAEGQVKKDMDVINSTIFDIVTQTLEHRAKGTQDDEGGGKDIVSLFLDDLNKSGDV
DEGCFDPTYLRDIVLNFIIAGRDTTAQALSWFFYCLSKNPDVETKIREEVAAKLPKLVSGQCSPSMDEVGELIYVEAALRET
LRLYPSVPIVSKQAVHDTVLSDGTFIAAGTMAGLPMYALGRMPHVWGPDAAEFKPERWIDSGKLISVSAYQFVAFNAG
PRLCLGKNLAMLEMKLIVASLLSKYHVELENPENVTYAISFTLPVKGQLNAKISAV*

>CYP5014D15 Phytophthora capsici

MTDKLSSSVAVAVVTGLVVFPLAWRLLSTNDGNKSQHGTRKVVRPSTTKLFLGNTLDVIGNIPIRHDWIASLC
EEAKGEPVLLQSLGTPDMTLLSTPEAFEDVFKNQFDNFPKGAKKGEYLRELLGEGIFAVDHEKWYRQRKTASNLFSMRA
LRDSMTSIIQRHLVVLERIFHRAAETKGTIDMFRLLNRFTMEAFTEIGFGVQLNCLDADKEHPFQTAFDRSQQSLALRFL
RPSWFWKTQRLLSIGAEGQVKRDMNVINSTIFDIVTQTLDHRAMDTKEHEKRDKDIVSLFLDDLKKTGNADESHFDPTY
LRDIVVNFIIAGRDTTAQALSWLFYCLSQNLDVETKIRAEIHAKLPKLSNGQCSPSMDDVSELIYVEAALRETLRLYPSVPIV
SKQAVHDAVLSDGTFIKAGAMAGLPMYALGRMPHVWGPDAAQFKPERWIDSQSGKLISVSAYKFVAFNAGPRLCLG
KNLAMLEMKLIVASLLSKYHVQLENPENVTYAISVTLPIKGQLNAKITNFH

>CYP5014D16 P.parasitica

MGCLDSDEEHPFQKAFDRAQRALRFRFTRPGWFWKTQRWLGLGVEGQLRRDIEVIDKTVLEIVEKALAQRA
NHGGEVKNGGDIVSLFLDRVSNTPGAKDREFNPNYLRNIVVNFLIAGRDTTAQALSWFFFNISKNPRVEASIRKEIADKL
PTYKVDGFPTMQDVSQLAYLEAALKETLRLHPSVPVEPKQTLEDTTLSDGTFVPAGSAIGLATYAMARMPKVWGPDAE
EFKPERWIDSTSGKLLAVSAYKFASFNAGPRMCLGMNLAMLEMKLVIVGLLSRFHIEVLNPEDVTYDLSLTLPVKGALNV
KVSRHRWPAHA*

>CYP5014D17 Phytophthora capsici

MGLIVPKTSDGRVLFFLPWENGTLAGKTDSQSEITMLPSPTKEEVDFIIDEANRYLAKDVTRNDIKSAWSGIRP
LVKDPRHADGSTAKISREHVVEVSDSNMVTIAGGKWTTYRRMAQDAIDKLVETGKDIVSLFLDSLDGSPNADTQQFDP
MYLCDIVVNFLIAGRDTTAQTLSWFFLNLTKNPQVETTIRNEIAEKLSNEGDITNATMKDVAQLTYLEAALKETLRLHPPV
PMSPKFVIQDTTLADGTSVKGNSMIVLATYAMARMQNVWGEDAAEFKPERWIDAASGKLINFSPFKFVSFNAGPRLC
LGMNLAMLEMKLVVAGLLKRFHVEVLSPEEITYDISLTLPKER

>CYP5014D18 P.ultimum

MLAIGSLLELNAASVLLGILSLQVLVFAYHALVAMKASRSDKSETRKVWRPDSTLPFVENTLDLLRHVPDFQD
WIFSICKQFKGEPFLIKAFGRPDMTVLYTPQVIEDVFKHQFACFPKGPFFIDNLKDLLGEGIFAVDDANAQREVILRFFRP
GWFWKTQRWLGIGAEGRLKQNLKVIDDTVLDIVTKSLERRRINDEPAKGDTEKDIVSLFLDNAIRDPDFEANEFDPKYLR
DIVVNFLIAGRDTTAQALSWFFYNLSSHPEVVAKIRAEIAAKLPELASGKIATPTMEQVQDLVYLEAALKETLRLHPSVPLE
TKHVVRDIVLSDGTFIRGGTTVALPFYGIGRMAHIWGPDAEVFSPDRWIDARTGKLINVSAYKFVAFNAGPRLCLGMNL
AMLEMKLVVSGILSRFDVELLLGQTITYDFSLTLPVHGAMMASVRKTPRQ*

>CYP5014D19 P.parasitica

MGSLDSDEEHPIQKAFNHAQRALLLRFVRPGWFWKIQRWLGVGVEGEFKHDLKVINTTVMDIVEKALARRS
SNNSDNANDILSLILDNVGSSPNADKQQFDPVFLRDIAVMFLVAGRDTVAQTLSWLYLMLSKNTYWEVAIRSEMLQKT
VILKPDATDITIEDVDQLVALEAVLMESLRLHPPVPMIPKYVEKDTTLADGTFVEANSLIVLATYAMARMEQVWGPDAA
EFKPLRWIDSSSGWAEIAPSKFRLYLGMDLVMSEMKLVMAGLLTKFHIEVQDLEKVAHDVSLTLPVKGSLDAKIIRCTYS
KLPKHS*

>CYP5014D2 P. sojae

MLSVSSLRNKLPFNPVKLGIGTLVFASVVVLLAKPPYEPTKKEDPSKKSHRKIHRPEATLPVLENTLTVIEAARAG
DIHDRTLLSCRESNAEPVLVRSIGVPDQLIVCTPEAFEDVLKLEFSNFPKGSYQCENLRDLLGDGIFAVDGEQWVHQRKT
ASNLFTMRALRDSMAFVIQRHAVVLYDILRQTSESNETLDLFKLLNRFTIEAFTEIGFGVHMGCLDSEEEHPFQKAFDHA
QRALLLRFVRPGWFWELQKWLGVGAEGQLKNDIEVINKTVLDIVEKALAKRSSIGSGIEIDGSASQGKDIVSLFLGDADS
DTQQLDPMFLRNIVVNFLIAGRDTTAQTLSWFFLNLAKNPDVETAIRNEIAKKLPNIEGSEVNVSHATMQDVSQLVYLE
AALKETLRLHPPVPMIPKYVVEDTTLSDGTFVKAGSLIVLATYVMARLPQVWGPDAEEFKPERWIDPSTGKLIVVSAYKF
ASFNAGPRMCLGMNLAMLEMKLVVAGLLSKFHVEVLNPEDVTYDLSLTLPLKGALNVKVSQAALPSNPDFA*

>CYP5014D2 P. ramorum

MLKWISLFDHSGVAPVHPLLIADRFDVDLVSIDLVRIQRKVHRPDSTLPLLENTLDILQAARDGDIHDRTVRAC
RGFKGEPVLIRSIGLPDKLVVSTPEAFEDVLKTQFNNFPKGSYMCENLRDLLGDGIFVADGDQWVHQRKTASNLFTMR
ALRDSMTVVIQRHAVVLYDILQRASDNKETLDLFRLFNRFTIEAFAEIGFGVHMGCLDSDEEHPFQKAFDRAQRALLLRF
VRPGWFWKTQQWLSVGAEGRLKQDIEVINATVLEIVEKALAKRSTVSNDEIGDEDKNIVTLFLDSVGGFASADSQQPD
PMHLRDIVVNFLIAGRDTTAQTLSWFFLNLTKNSDVEAAIRDEIAEKLPKGGRNATTSATATMQDVSQLVYLEAALKETL
RLHPPVPMAPKYVVEDTTLSDGTFVKGKSMIVLATYVMARMQEVWGPDAEVFKPERWIDAATGKLVNVSAYKFASF
NAGPRLCLGVNLAMLEMKLVVAGLLSKFHVEVLNPEDVTYELSLTLPVKGPLNVKVSPAKLPTSPDFA

>CYP5014D3 P. sojae

MLPILQLVEKSPVAGLALTGLLVLPLVITLHSRHKKSEEGIGKIHRPASTLPFLGNTWDLVIHGVRGDMHDFMV
QIGKQFNAEPVLLQALGIPLNLILYTPEGFEDVLKTQFSNFGKGPFMRENLRDLMGDGIFAVDGEQWVHQRKTASNLF

TMRALRDSMTVVIQRHAVVLYDILRRASESKETLDLFKLLNRFTIEAFTEIGFGVHMGCLDSEEEHPFATAFDRAQRALRF
RFTRPGWFWKTQRWLGLGVEGQLQRDIQVIDKTVLEIVEKALARRSSRVENPEKKAGGDIVSLFLDSAGSSNEKQFDPK
YLRDIVVNFLIAGRDTTAQALSWFFFNISKNPRVEAAIRNELAQRLPKVKAEAATPSMQDVSQLVYLEAALKETLRLHPS
VPVEPKQTLKDTTLSDGTFVPAGSAIALANYAMGRMPQVWGPDAEEFKPERWIDPSTWKLIAVSAYKFASFNAGPRM
CLGMNLAMLEMKLVVAGLLSKFHIEVLNPENVTYDVSLTLPVKGALTVKVSQIAEPAGA*

>CYP5014D3 P. ramorum

MLPIAELVENSSVTIGGLALLALALSFWHKMRVDEKSKVHRPDSTLPFLENTLDLIIHGGGKGDLHDFTTQMA
KQFDAEPVFLRALGIPLNIMLFTPEAFEDVLKTQFHNFEKGPFVCENLKDLLGEGIFAVDGDQWVHQRKTASNLFTMRA
LRDSMTVVIQRHAVVLYDILQRASDNKETLDLFRLFNRFTIEAFAEIGFGVHMGCLDSDEEHPFQKAFDRAQRALLLRFT
RPGWFWKTQRWLGVGVEGQLRRDIQVIDKTVLDIVEKALAQRAQRVTHGDGGYDNGDNKVKKVGNIVSLFMDNLA
NSQQFDPKYLRDIVVNFLVAGRDTTAQALSWFFVDLSKNPHVEVAIRKEMAEKLQPAEVNRPGAVSSTMEDVSHRVYL
EAALKEALRLHPSVPVVPKQAVQDTMLSDGTFVPAGSAIGLANYAMARMPQVWGPDAEEFKPERWIDPTTGKLIPVS
AYKFASFNAGPRMCLGMNLAMLEMKLVVAGLLSKFHVEVLNPEDVTYDLSLTLPVKGALNVKVSSIGRSASPAYA*

>CYP5014D4 P.vexan

MESFPLSATGAVLGALMLANLVVTVMASRKPSEPPASTRKVHRIPSTLPVLGNTWDLVRMLPRIHDFTADNC
LLFRGEPFVTRALGQPDMVTVSTPTAVEDVMKTHFDKFPKGSYFCDNVHDLLGDGIFGVDGDKWVHQRKTASHLFT
MRALRGSMTSSIRSHTVELRRIFDRAASDGAPLDLFKLFNRFTMEAFAEIGFGVRLGALAANVEHPFQTAFDGAQRVLA
LRFTRPMWFWKTQRWFNVGAEGELRRHIHVIDSTVFGIINKSLARRSATHGGVDADVEQRQRDIVSLFLDNMHSVPG
IDAEQQRFDPTYLRDIVVNFLIAGRDTTAQALSWFFLCLSANPAVEATIVSEIRSKTPRVASGEDTTPTMEQASELVYLEA
ALKETLRLYPSVPFVNKKATEDVVLSDGTLIAAQSIVGVPMYAMGRMPYVWGPDAAEFKPERWLDPATGKLVNASPF
KFVAFNAGPRTCLGMNLALLEMKLVVSSLLARFRMTLEHPAAVRYDFSLTLPVKGVMSAGL

>CYP5014D5 P.parasitica

MLSISSLRDKIPFSAVNVGVGGLVLASVVVLLVKTRNDSTKPQLRKIHRPDTTLPLLENTLDFIRAARDGDMHD
RAVELCSQFNGEPILFRSIGMPDELVISTPEAFEDVLKTQFNNFPKGSYMCQNLKDLLGDGIFAADGDLWVHQRKTASN
LFTMRALRDSMAATIQRHAAVLFDILQRASDSGETLDLFRLLNRFTFEAFAEIGFGVRMGCLDSDEEHPFQKAFDHAQR
ALLLRFVRPGWFWKTQRWLGVGVEGELKSDIEVINTTVLEIVEKALARRSSNCSDSGVDTGRDIVSLFLDSVGSSPNANT
QQFDPMYLRDIVVNFLIAGRDTTAQTLSWFFLNLTKNPHVETTIRNEIAEKIQKSGVEEAETTGATMQDVSQLVTLEAAL
RETLRLHPPVPLIPKHVAEDTTLADGTFVKGNSLVVLATYAMARMQHVWGPDAAEFKPERWIDSTTGKLISVSPYKFAS
FNAGPRLCLGMNLAMLEMKLVVAGLLSKFHIEVQDPDKVTYDVSLTLPVKGSLDVKISRTVSPTGPDFA*

>CYP5014D6 Phytophthora capsici

MLALSNQSNLDIVTFATVSGLALFLAVSRHKKRQDDAVRKIHSPDSTLPLLGNTWDLIIHGIKGDMHDFTTQIG
KQFNAEPIVIRALGIPLNLIFFTPEAFEDVLKNQFQNFDKGPFMCENLRDLLGDGIFAVDGEPWVHQRKTASNLFTMRA
LRDSMTVIIQRHTATLNDVFQRAKETKETLDLFKLFNRFTIEAFAEIAFGVHMGCLDSDEEHPFQKAFDRAQRALLLRFTR
PGWFWKTQRWLGKGVEGQLQRDIQIIDKTVLEIVEKALAQRASNSDKGVENKGGDVVSIFLDKVDVLEKQLDPKYLRD
IVVNFLIAGRDTTAQALSWFFYNVSKNPQVEIEIRKEIEEKLPKFGTATMEDVSQLVYLEASLKETLRLHPSVPFEPKQTVE
DTTLSDGTFVPAGSAIGLATYAMGRMTQVWGADAEEFKPERWINAATGKLIAVSAYKFASFNAGPRMCLGMNLAML
EMKLVVVGLMRNFHIEVVNPEAVTYDLSLTLPVKGALNVKVSQPWAAHA*

>CYP5014D7 P.vexan

LVVYTPEAVEDVLKTHFASFPKGDYVADVARDLVGDGIFAADGDKWSHQRKTVSALFTRKALRDSMTRVIQQ RAGALNAVLARAQASGTAVDLNKLFSRFTMETFAEIGFGVHMNCLDADEEHPFQASFDRAQRAIGARFLRPRWLWKL ERWLQLGREARLKDDISVINSTVVSIIERACERHVNPSASERASEPKGDDIVSLFLDSFQHLQQAGDAPVDPMYLRDIVV TFVMAGRDSIAQATSWFFCCLARHPAVEAKIRAEIDAKLGSDVNAPLTMDDVHELVYLESALKETLRLYPSVPFFRRRAT SDVVLSDGTFVPANTTVALATFAMARMPFVWGRDAAEFKPERWIDAATGKPQVVSPFKFSAFNAGPRTCVGQNLA MLEMKLLVAGLLRRFRVEVLRPEAVTYDCSMNLSIKGSLDARIHAA*

>CYP5014D8 P.iwayamai

LSVAVLLELSPLTVLAGLVALPMALFICSSLLATGAKCEPLTKGTRKVWRPLGTLPLLQNTLALVMHVPDFHDW FASLCKHFDGEPYLIKSWGRPDLMILSTPQASEDMFKTEFANFPKGDFFNDNLRDLLGEGIFAVDGAKWVHQRKTASH LFTMRALRDSMTATVQSHALVLHQVLARSVDQQSSIDLFKLFNQFTIEVFAEIGFGIHMGCLDSETEHPFQTAFDSAQRI IALRFLRPGALWRVQRFLGIGAEGELKKNMKAIDDTVLDIVAKSLKRRNDTTSTSDGEKDTDIVSLFLDNASQNPDFSGR EFDPVYLRDIVVNFLIAGRDTTAQALSWFFFNLSSHPKVEKKIRDEIAAAIPELVSGAISTPSMGQVQHLVYLEAALKETLR LHPSVPFMTKAVAHDIVLSDGTFIRGNTTVGVPAYGMGRMTSVWGPDAEQFNPDRWIDAATDKVKTESPYKFVAFN AGPRICLGMNLAMLEMKLVVAGLLSKFHVELLLNQHITYDFSLTLPVHGAMMAT*

>CYP5014D9 P.irregulare

HLSPLGVFLGFLAVPIAALLLYVKLSAKGPSFEAQTKGSRKVWRPESTLPFLENTLEMVLRVPDLHHWITSLCQH FQGEPFLLKAFGIPDMTVVYSPQAFEDVFKNEFACFPKGTFMINNMRDLLGEGIFAVDGAKWVHQRKTASHLFTMRSL RDSMTATVQSHACVLHDVLQRTCDAKKPVDLFKLFNQFTIEAFAEIGFGIHMECLSSEDEHPFQTAFDSAQRVIALRFLR PGAFWRLQRLLGVGAEGRLKKNLKVINDTVLDIVAQSLKRRNSMQSSNAERGPGAAGGKDIVSLFLDNVSQNPDFEQK QFDPQYLRDIVVNFLIAGRDTTAQALSWFFFNLSSHPEVERKIRAEIAVALPELANGTIQTPSMDQVQDLVYLEAALKETL RLHPSVPFSTKEVAHDVVLSDGTFLRGNTTVGLPFYGMGRMTSVWGPDAETFNPDRWINTATGKIKAESTYKFIAFNA GPRLCLGMNLAMLEMKLVVAGLLSNFYLQLVPGQTVTYDHSLTLP*

>CYP5014D-fragment1 P.aphanidermatum

MMTESMRTFAVQLQKLLQQKSDRGEHIDVFKLMNAFTMQAFAKMGFGADIDCLGAPEEHPFQSAFDSSQ RLLMRRALRPTWLWHLERWLNIGAEAQLKRDIAVIDSTVMTIISQSLVRRQEKQQRTSEDDTGFSDIVSIFLDRFDSTST EKGEFDPTYLRDIVVNFLIAGRDTTAQAMSWCLYNLHKNPEVVRKIREEIARELPAFFADRNAPAPTMEQVQQLTYLEA TLKETLLIATVVSRFNIQFDDIDSITYTPSLTLPVRGAMMARVTSSAP*

>CYP5014D-fragment2 P.iwayamai

LGFLHTWVLSQKFTGLQLLAGLVVVTGSLQLLTWALARLSPRSAAKQQPYNNVCEPATTLPVLKNTLDFMKH QWRFHDWITDECRLSNGQPWKFTAVGRPEVVVINSPALVEDVLKTHFANFVKGPYLNGILSDLLGDGIFAVDDANXXX XXXXXXXXXXXXXXXXXXXXVQTHAQILSRIFDDAAASRKLFDLFKLLNQFTIEAFAEIGFGIKMGCLDAETEHPFQRAFD TAQYLTTKRFMPPTWVWKFMRALGLGWEAPLKKSFKDIDDTVLDIISRSLELRTASDSQKRDANDIVSLFLDSVSHTIEG SQEPFDPXXXXXXXXXXXXXXXXXXXALSWLFLNLSQHPEVEKKIRDEIMTAIPQLASGAISTPSMDQVQHLPYLEAALKE TLRLHPSVPFNTKRAVKDTMLSDETFVKAGTTIGLPTFAMGRMTHVWGPDAAEFKPERWIDQATGKL*

>CYP5014E1 P. ramorum

MEPWSHGAKAVATVCRPADIRYRANLVADPPDLKALSRTFRPGHKSIRVVMRTADVVACIQILWPTFPPWA SVLVECLVISDRNEGGGGGGINTISYPNSTSLGQVGFKLSRLISLTMLLPLQLMGDARAPVGLLACGLLVSAVAVVRVRS

HTQQKPHDTATRVPYLPTLVPLLGNTLELATNVGRFHEWVTGHSQQRDGKPFALRLLGKNDLVFIARPEHFEEVLKTQS
RNFCKSDTTREVFDDFLGEDIVLLNGKRWQFHRKILASLITTRALREYMTPIIQENILRLQRTLKQWSETKGSVDFHKLVR
HFTIDTFAEIGFGCKLDVLASGEEHPFEVAFNDANRISSERITSPTWIWKLKRWLNIGDERRLREAIEEMNELMMKLIFTA
FGLLQAGADDNQQSAHQNLISIIVSSEREITPTEVRDIALSGLEAGRNTTADTLSWLFHALSHNPRVETTLRAEIAAKLPKL
EESDSYVPSFEEIQEVPYLEATIREVLRLYPTVPTVPYHCVNDTVLADNTFIPAGTDVFLTLYAAGRLASVWGHNATEFSP
ERFIDEKTGKLCQDSPYSAFSDGPRVCLGRNLAMLQLKIVAATIISRFHLSEDPEQDVQPILDLTIGMKDPLMMRVETTQ
QEEAASFS*

>CYP5014E2 P. sojae

SNLITTRALREYMAPVIQEKTLLLQSILADKSETKEPFDMYKLMRQFTLDTFAEIGFGCHLEILTSGKEHPFEVAF
DEANRISSERFTKPTWLWKFQRFLNIGNERRLREAISVMNEFSVDLIMEAMEQMKNSKPDEADVESPAHKNIMAILLSK
KEAVTPTQVRDIVLTSLEARRNTTSDTLAWFFHSLSHHPQVERKLRAEIRSKLPKFGEIHIYVPSYEAVQDLPYLEATLREA
LRLHPTGPSIPYHYQRDTVLQDETFISAGTDVFLHLYSAGRLTSAWGSDAASFNSQRFNDLTTGEVLPSKYSPFSSGPRVC
IGRNLALLEMKIAIAAVVGRFRLCEEPSLTQRRPAFQNFQISELFIPVCIDLTRRRSTS*

>CYP5014E4 P.parasitica

MDSPLQVDSNLPVNIGTLACVLVLISAFVMLGFSTKSASKNASSSLYLPSRIPIFGNTLSFAMNVGRYHEWVTG
HSLERDGQPFALNLVGKSDVVYVSRPEHFEEILKTQNKNFIKSDSVRDTFDDFLGENIVLLNGEQWKFHRKVMVNLLTP
RALREYITPVVQEKVLVLRNVLNEASRAKQTFDMNKLIRQFTLDTFVEIGLGCKLNLLTSGEEHPFEIAFDEANHLTSKRFS
TPTWIWKPMRWLNIGSERRLRDAIGEMNEFIVNLIMDAMEQVSSLKESATENHRTCKNIITILLNNDQDIAPTLVRDIVL
TGLEAGRNTTADTLVWLFHALSLNPRVEKKLRDEILTKMPKFKESDTYVPTYEEIQDLPYMEATIQETFRLFPSVPVIPYHC
ACDTVLKDGTFIPAGVDVFLYLYGAGRLASVWGLHVLSFMPERFLDEETGKLLQDPPAKNWAFGTGPRLCVGRNLAM
VELKMVIATVVSQFTLAEEPGQDVQPIVDLTLTMKNPLMMRVEAAN*

>CYP5014E5 Phytophthora capsici

MLSPLEYTGVVTFGVVAVSVALFFASKSSKTLKGISTLPYLPSRLPLIGNMVALATNMDRYNEWLTEITVARDG
KPFALHLFGRADVVYLSRPEHFEEVFKTQSSNFIKGDSVREMFDDLLGEDAVLINGERWQFHRKTLVNLISTRALRDHM
TPIIQDKVTELLDVLRQLVNSEHSFDLYKLLQKLTLDIFAEIGFGCRLNLLTSKEEHSFGVAFDYANKLSSERVAKPTWLWK
CQRFFNVGNERRLRENIHEMDKFIVDLITEAMEKIQSGKDKTAKNILTILLNTKKDITPKEVRDISLTGLEAGRNTTADTLA
WLFHWLSQNPRVEDKLRGEIHKKLPGFRTSDRYTPSYEEIQDLPYLEATIREVLRLSPTVPAIPYHCAVDTVLDGIFIPAGT
DIFLHLYATGRLTSVWGEDANCFIPERFLDKKTGKLLQDPPVNYSPFSAGPRICIGRNLAMLEMKMAVAAIVSQFKLTEE
PGQEVRPILDLTLTMKNPLLVRATAM

>CYP5014F1 P. sojae

MLQSMFSKSPVVPGLVTAALLVTLYWTKSAKGSAKLKGDKVKNAVILPGTLPVVGNAVELAANAARMHDWL
ADQFAATNGEAFIVRLPGKDDMMFIAKPEHLEAVLKTQFDVFPKSEYIHDVFYDMLGDGIVVTNGETWKRQRNVVVG
LFSARALREHMTPLVQKYTVQLGDILADAAATNTPVDVFDLLHRYTFDVFGEIGFGAKMGSMDGAFQPFAEAMDEAQ
FLAGKRFKQPMWYWKLRRWLNVGDEKKLKENVRVIDEHLMGIIADAIERRRHRVEEMKAGRPAALADKDIVSIVLDS
MEASGQPVNPVEVRNIAVASIIAGRDTTADCMGWLFHLLSENPRVEAKLRDEVLAKIPQLATDKSYVPSVEDINKVPYL
EACIRELLRLYPPGPLITTHCIKDTVFPDGTFVPANTDIGIALFSAGRLTSVWGEDALEYKPERFIDSESGEIIPMTATKFCAF
SAGPRICVGQNLAFVETKIVIASIVGRFHMIPEPGQNVAYTQGISLGMMDPLMMRLEAVNASA*

>CYP5014F1 P. ramorum

MLQSMFKSPVVPSLVTAAVLVTLYWTKSAKVSAKIKGDKVKSAVILPSTLPVLGNAVELASNAARMHDWLAD
QFATTDGEAFIVRLPGKDDMMFIAKPEHLEAVLKTQFDVFPKSEYIHDVFCDMLGDGIVVTNGETWKRQRNVVVGLFS
ARALREHMTPLVQKYTVQLVDILADAAASNAPLDVFDLLHRYTFDVFGEIGFGARMGSMKGAFQPFAEAMDEAQFLA
GKRFKQPMWYWKLRRWLNVGDEKKLRENVRVIDEHLMGIIADAIERRRIRVEQKKEGRAAALADKDIVSIVLDTMDTK
GLPVNPVEVRNIALASIIAGRDTTADCMGWLFHLLSQNPRAEAKLREEVLAKIPQLSADKHYTPTVEDINKVPYLEACIRE
LLRLYPPAPLITTHCIKDTVFPDGTFVPAKTDIGIALFSAGRLTSVWGEDALEFKPERFLDTETGEVISMTATKFSAFSAGPR
ICVGQNLAFIETKIVIASIVSRFRMTPEPGQNVAYTEGISLGMMDPLMMRLESVN*

>CYP5014F2 P. sojae

MLQSFFEDKLSYLHPVVPGLVAAAVAVAIYCTTDTAEPPTLEGEDGKAVAKRVRYLPSKIPVLGNAIDLLSNSER
MHDWIADQIVPFDGEPFTLRLPGKSDMMFIAKPEHIEEVLKTQFENFPKSQHIHDVFFDLLGDGIVTTNGETWKRQRR
VLVNLFSARALREHMTPISQKYVVQLRKIFEDAVASKEPMDAFGLMHRYTLDVFAVIGFGTEMKLLEGRYQPFAEAIEES
QYIVSARFKQPDAQWKLMRWLNIGSEKKLRHAIQVIDEHVMGIISGAIQRRQERDQAIKAGEAAKPADRDIVSIILDSM
ESNNQVVDPVEVRNIATAALIAGRDTTADALGWLFHVLSQNPSVEAKLRSELLTHMPRLTTDPEYVPTAEELNQVPYLE
ATIRELLRLLPAGPVIATHCVRDTVFPDGTFVPKNTDIGLAFYTTGRLTSVWGEDALEFKPERFLDADTGEVVKVSSSKFC
AFSAGPRICVGRNLAFLEMKIVIANILSRFHLVPEPGQQPTYTQGITLGMQTPLMMRVEAVTAHAAA*

>CYP5014F2 P. ramorum

MLQSFFEDKLSFLGPVVPGLIAAAVVVAVYYSTSSTHVSPIGDNEVCDGKVKPKRVRYLPSKIPVLGNAIELLSN
AERMHDWIADQIVPFDGEPFTLSLPGKDDMMFIAKPEHIEQVLKTQFDNFPKSQHIHDLFFDLLGDGVVITNGETWKR
QRRVLVNLFSARALREHMTPISQKYVVQLRKIFEEAAASKEPMDAFGLMHRYTLDVFAEIGFGTEMKLLEGRYQPFAEA
IEESQYIVSARFKQPDALWKIKRWLNIGSEKKLRHAVQVIDEHVMGIISGAIQRRQQRVEAARSGKEAKPVDRDIVSIILD
SMESNNQVVDPVEVRNIAAAALIAGRDTTADALGWLIHVLSENPRVEAKLRSELLAHLPKLATDIDYVPSAEELSQVHYL
EATIRELLRILPAGPAIATHCVRDTVFPDGTFVPKNTDIGISFYTTGRLSGVWGEDVSEFKPERFLDADTGEVVKVSSSKFC
AFSAGPRICVGRNLAFLEMKIVIANILGRFHLVPEPGQKPAYTQGITLGMQTPLMMRVEAVSSQDSSVAA*

>CYP5014F3 P.parasitica

MLQSFFGDKLSLLNPVVPGLIAAAVVVAVYYTTETTELPIVEDAEEQDGKAVPKRVRYLPSKIPVLGNAMELLS
NSERMHDWIADQIVPRNGEPFTLSLPGKDDMMFIAKPEHIEQVLKTQFDNFPKSQHIHDVFFDLLGDGIVITNGETWK
RQRRVLVNLFSARTLREHMTPISQKYVVKLRSIFEEAAASNKPMDAFGLMHRYTLDVFAEIGFGTEMNLLEGRYQPFAE
AIEESQYIVSARFKQPDALWKIKRWLNVGSEKKLRHAIDVIDEHIMRIVSEAIQRRQERKEAIKAGKDVKPADKDIVSIILD
SMETNNQVVDPVEVRNIAAAALIAGRDTTADALGWLLHVLSENPNVEAKLRSELLKHMPKLATDSKYVPTVEELSEVPY
LEATIRELLRVMPAGPLIATHCVRDTVFPDGTFVPKNTDIGIAFYTTGRLTSVWGEDALEFKPERFLDAETGEIIKVSSSKFC
AFSAGPRICVGRNLAYLEMKIVIANIVSRFHLVPEPNQPTPAYTQGITLGMQTPLMMRVETVASQDSGVAA*

>CYP5014F4 P.infestans

MFIAKPEHIEQVLKTQFDNFPKSQHIHDVFFDLLGDGIVITNGETWKRQRRVLVNLFSARTLREHMTPISQKYV
VKLRSIFEEAAASNKPMDAFGLMHRYTLDVFAEIGFGTEMRILEGQYEQFAEAIEESQYIVSARFKQPDALWKTKRWLN
VGSEKKLRHAIQVIDEHIMSIVSGAIQRRQERNEAIKAGKDVKPADKDIVSIILDSMESYNQVVDPVEVRNIAAAALIAGR
DTTADALGWLLHVLSENPRVEAKLRSELLKHMPKLSTDATYVPAVEELNEVPYLEATIRELLRILPAGPLIATHCARDTVFP
DGTFVPKNTDIGIAFYTTGRLTSVWGEDALEFKPERFLDAQTGEVAKVSSSKFCAFSAGPRICVGRNLAFLEMKIVIANIV
SRFHLVPEPDQPKPTYTQGITLGMQTPLMMRVEAMASQQRGVVA*

>CYP5014F5 Phytophthora capsici

MLQSFFSDKLSEVNPVLPGLVAAVVIVAFYCVSGVPTVEGEEERDGKVVSKHVRYLPSKIPVLGNAIELLGNSER
MYDWITDQMAPFDGEPFRLSLPGKEDMMFIAKPEHIEQVLKTQFDNFPKSQHIHDVFFDLLGDGIVITNGDTWKRQR
RVLVNLFSARALRENMTPISQKYAVQLRKIFEKAVESKEALDVFGLMHCYTLDVFGEIGFGTEMKLLDGRYQPLAEAIEES
QYIVSARFKQPDALWKIKRWLDVGSEKKLRHAIEVIDEHVMGIISGAIQRRQERKEVGKKANHADKDIVSIILDSMESNN
QVVDPVEVRNIATAALIAGRDTTADALGWLFHVLSENPSVEAKLRSELLKHLPKFATDAEYAPTVEELNEVHYLEATIREL
LRILPAGPLIATQCVRDTVFPDGTFVPKNTDIVVAFYTTGRLKSVWGEDVLEFKPERFLDPETGEVIKVSSSKFCAFSAGPR
ICVGRNLAFLEMKVVIANIVSRFHLVPEPGRPKPAYTQGITLGMQAPLMMRVEAIASLDPSTITTANHA*

>CYP5014F6 H.arabidopsidis

MSCLYLAVPGLLAAAAAVFAAVYFTSTASNLSTVSVEEKFNNKVVAKRVRYLPSKIPLLGNSIDLLLNSERMHD
WIAGHLLSSHGEPFTLRLLGKDDMMFIAKPEHIEQVLKTQFSNFPKSRHIHDVFFDLMGDGIVVTNGENWKRQRRVLV
NLFSARALREHMTPLSQKYVVQLRRVFEDAAVSRTPIDAFGLMHCYTLDVFAEIGFGTETKLLQGCDQTLATAIEESLYIV
SARFKQPDALWKLKRWLNVGSERKLRRAIQVIDEHVMGIISCALQHHQEQKKAMKKGEAVQPVYGKDIVSIILDSMES
TGEVVDPVEVRNIATAALIAGRDTTADALGWLLHILSENPSVEAKLRSELLKYLPKLATDADYVPSLEDISEVHYLEATIREV
LRLYPSGPLIATHCIHDTVFPDGTFIPKNMDIGIALYTTGRLPSVWGEDALQFKPERFLDVKSGEVLKMSSCKFCAFSAGP
RICVGRNLAYVEIKIVIANIISRFHLKPEPGQKPAYSQGITLGMQTPLMMRVETIGVQDSRYKSYSLKSAMAA*

>CYP5014F7 P.infestans

LQSMFKSPVVPGLVTAALFVTLYWTKANKSSAKSKGDKVKYAVILPGTLPVLGNAVELAANAPRMHDWLAD
QFAATNGEAFIVRLPGKDDMMFIAKPEHLEAVLKTQFDIFPKSEYIHDVFCDILGDGIVVTNGETWKRQRNVVVGLFSA
RALREHMTPLVQKYTVQLVDILTDAAATNTPLDVFDLLHRYTFDVFGEIGFGAKMGSMDGAFQPFAEAMDEAQFLAG
KRFKQPMWYWKLRRWLNVGDEKKLKENVRVIDEHLIGIIADAIERRRLRVEEKKAGRPAALADKDIVSIVLDNMESRG
MPVDPVEVRNIAVASIIAGRDTTADCMGWLFHMLSQNPNAETKLRAELLSKIPKLGTDRHYTPTVEDINKVPYLEACIRE
LLRLYPPGPLITTHCIKDTVFPDGTFVPANTDIGIALFSCGRLTSVWGEDALEFKPDRFIDSETSDIIPMTATKFAAFSAGPR
ICVGQTLAFIETKIVIASIVARFEMTPEPGQNVAYTQGISLGMMDPLMMRLKSV*

>CYP5014F8 P.parasitica

MLQSMFKSPVVPGLVTAALLVTLYWTKASKCSAKAKGDNVKHAVILPGTLPVLGNAVELAANAPRMHDWL
ADQFAATNGEAFIVRLPGKDDMMFIAKPEHLEAVLKTQFDIFPKSEYIHDVFCDMLGDGIVVTNGETWKRQRNVVVGL
FSARALREHMTPLVQKYTVQLVDILANAAATDTPLDVFDLLHRYTFDVFGEIGFGAKMGSMDGAFQPFAEAMDEAQF
LAGKRFKQPMWYWKLRRWLNVGDEKKLKENVRVIDEHLMGIIADAIERRRRRVEEKKAGRPAALADKDIVSIVLDNM
ESRGMPVDPVEVRNIAVASIIAGRDTTADCMGWLFHLLSQNPNAEAKLRAEVLAKIPKLSTDRHYTPTVEDINKVPYLEA
CIRELLRLYPPGPLITTHCIKDTVFPDGTFVPANTDIGIALFSCGRLTSVWGEDALEFKPERFIDSETGEIIPMTATKFAAFSA
GPRICVGQNLAFVETKIVIASIVARFEMVPEPGQNVAYTQGISLGMMDPLMMRLKSV*

>CYP5014F9 Phytophthora capsici

MLQSMFKSPVVPSLVTAALLVTLYWTKAVKVADKSKGDKVKHAVILPGTLPVLGNALELAANAPRMHDWLA
DQFASTDGEAFIVRLPGKDDMMFIAKPEHLEAVLKTQFDIFPKSEYIHDVFCDMLGDGIVVTNGETWKRQRNVVVGLF
SARALRDHMTPLVQKYTVQLCDILADAAVKNTPLDVFDLLHRYTFDVFGEIGFGAKMGSMGGAFQPFAEAMDEAQFL
AGKRFKQPVWYWKLRRWLNVGDEKKLKENVRVIDEHLMGIIADAIERRRQRVQEKKAGRPAALADKDIVSIVLDTMES
RGLPVDPVEVRNIAVASIIAGRDTTADCMGWLLHMLSQNPSAEVKLRAELLTKIPQLITDQHYTPTMEDVSKVPYLEACI

RELLRLYPPGPLITTHCIKDTVLPDGTFVPANTDMGIALFSCGRLTSVWGDDALEFKPERFIDSETGEVIPMTTTKFAAFSA
GPRICVGQNLAFVETKIVIASLVARFEMIPEPNQNVAYTQGISLGMMDPLMMRIKAVGEVIATLG*

>CYP5014-fragment1 P.ultimum

MFRHEPRVHDWIADQFVASKGQPVLTKAPGLPNMVMIASPELVEDVVKTQFDHFDKGAHQRGNLVDLLG
GGILATDGHSWMHQRKTSSNLFSMRALRDSMASIIRKHTXXXXXXXXXXXXXXXXXXXXXXXXXXXXXXXIGFGVCMN
CLAADSETQEHPFQTAFDSVQCLLTQRFRVPVWLWKLMKSLNCGWEARLKTDFAVIDDTVLEIITESLEXXXXXXXXXXX
XXXXXRLDIISLFLDHAEASNDNTSIDPKLLRDIVVNFLMAGRDTTAQALSWLFFSLSTRPKVEKKLRDELRLKFPALFERG
AAVDSVEQLSVDELQSLVYLEACLKETLRLYPSIPXXXXXXXXXXXXXNGTFVPKGSNLGISTYSMGRMTYIWGEDALEFK
PERWIDEVTGKLRIVSPYQFSAFHGGPRMCLGMKLSMLEMKIVVAGVVSRFNIRAVPGQHVTYASSLTLGMKGAFMV
DVTRVEDDNESFNAAASKCAH*

>CYP5014-fragment2 P.aphanidermatum

TLLVLALVRRRASLKPPGLSRKIHAPASTLPVLGNLFDAIRHEPYFHTWISDQCIKVQGEPWKLAIPGQPDMIVF
STSDAIEAITITPFDKFPKGQYQIDLTGTYFGLGLAASDGTRWLHQRRTATKFFSAKLLRAFMLQTLQKNVECASQFIEKN
MQSGLPVDLTRLFHDFTVDTFAEIGLGVQLQCIGSRVPHPFQSALEQAAIVISLRFRRPEWFWKLQQKLNVGYEAVLTE
SMDTVKAWLRDVIRTSVKQSLEKMAAGVSKQSDVKSVVELFIEASEENKEGLKEEDIVSTIKEAVRLYPAGPFTFREAAE
DTTVCELRILTANLLNCFVFSVDPINDVCSME*

>CYP5014G1 P. sojae

SSRALREHMAPVIQKHVRVLQRVLTDVAAAKMPIDMFNYSGRFTLDAFGEIAFGFNMSTLTLQRDHPFERAF
VDAQHIAASRLVVPTWYWKLKRSLNVGSERRLREALTTVDQFVMDVISKTVDKRNAPISDAEDKVHTRGRDIVSLILAN
ETVDGTPVDPILVRNVVLMALIAGRDTAADALAWLFHLLTLNPRVEEKLRAYLLASLPKLGSDFDYVPDMQEVQSLPYLE
ATINEALRLYSPVGLAQKLCVRDTVFPDGTFVPKGSNIALVYHAMARMPGVWGPDAAAFNPERFIDPQTGELIKVSSG
KFSAFNTGPRVCVGRKLAMMEMKMVVACVVSRFRFDEVPGQDVACGGGLTIGMKNPLMMRVQQLAPKEDGDEVV
VGVAA*

>CYP5014G1 P. ramorum

MLLWFLDSDDPLKKFGLLMLGVLIGAAVASQCLPSDTTDKKLIKQQQSVTPGAHGPVRVPFLPSMIPVVGHA
VLMAYHAGRFLDWVTDVFVSRGGAPFTLRLPFQRDMILTANPEHYEHVMKIQYDNFLKGDHIYDLLVDLLGDSILIVEG
DEWKFHRRVFVNLFSSRALREHMAPIIQKHVRVLQGVLSNAAQSKQAIDFFTYSGRFTLDAFGEIAFGFNMSTLTLQHE
HPFERAFVDAQHITAARLVVPTWYWKLKRWLNVGSEKNLREALTTVDQFVMDVISKTMDKRNAPPNDAEDKTRNRD
IVSLILANETVDGKPVDPILVRNVVLMALIAGRDTAADAIAWLFHLLTLNPRVEAKLRADLLARLPKLGTDLDYVPDSQDV
QGLAYLEATICEALRLFSPVGLAQKLCVHDTVFPDGTFVPKGANIALVYHAMARMPSVWGPDAASFVPERFLDPETGD
LLKVSSGKFSAFNTGPRVCVGRKLAMLEMKMVVACVVARFHLDEVPGQDVACSGGITIGMKNPLLMRVNELLPAAKS
DEVAVGVAA*

>CYP5014G2 P.infestans

MYVHNQTQLEANKLPTFTMLTWFLEVDDPFKKFGLLMLGVFVGAAVASQCLPNDTIDKKLSKQQQTVTPGA
RGPTRAPFLPSAIPVVGHAVFMAYHATRFLDWVTDVFLSRNGAPFTLRLPFQRNMILTANPEHYEHVMKTQYDNFLK
GDHIYDLLVDLLGDSILIVEGEEWKFHRRVFVNLFSSRALREHMAPIIQKHVRALQHVLTDAAKTQDPVDMFTYSGRFTL
DAFGEIAFGFNMNTLTLQHEHPFERAFVDAQHIVAARLVVPTWYWKLKRWLNVGTEKRLRDALTTVDQFVMDVISKT
MDTYGANESEGKAHRKDIVSLILANETVDGKPVDPILVRNVVLMALIAGRDTAADAMAWLFHLLTLNPRVEIKLRAELL

DKLPKLATYFDYVPDMQEVQNLTYLEATICEALRLFSPVGLAQKLCIRDTVFPDGTFVPKGSNIALVYHAMARMPGVW
GADAASFVPERFIDSQTGEMLKVSSGKFSAFNTGPRVCVGRKLAMMEMKMVVACVVCRFHLDEVPGQDVACSGGLT
IGMKNPLMMHVRKLTATDAVGVGEAA*

>CYP5014G3 P.parasitica

MHVHNQTQLEADKLPTTFIMLTWLLDVDDPFKKFGLLMLGVFVGAAVASQCMPSDNIDKKLSKQQQTVTP
GARGPTRVPFLPSAIPVVGHAVFMAYHANRFLDWVTDVFLSRNGAPFTLSLPFQRNMILTANPEHYEHVMKTQYDNF
LKGDHIYDLLVDLLGDSILIVEGEEWKFHRRVFVNLFSSRALREHMAPIIQKHVRVLQHVLAEATRTDEPIDMFTYSGRFT
LDAFGEIAFGFNMSTLTLQHEHPFERAFVDAQHIVAARLVVPTWYWKLKRWLNVGTEKRLREALGTVDQFVMDVISK
TMDMRSANESEDKPHRRDIVSLVLQNETVDGKPVDPILVRNVVLMALIAGRDTAADAMAWLFHLLTLNPHVEEKLRA
ELLSKLPKLATDFDYVPDIQEVQSLPYLEATICEALRLFSPVGLAQKLCVRDTVFPDGTFVPKGSNIALVYHAMARMPGV
WGADAASFVPERFIDPQTGEMLKVSSGKFSAFNTGPRVCVGRKLAMMEMKMVVACVVSRFHLDEVPGQDVACSGG
LTIGMKHPLMMHVQKLTATGAVAVGEAA*

>CYP5014G4 Phytophthora capsici

MILTANPEHYEHVMKTQYDNFLKGDHIYDLLVDLLGDSILIVEGEEWKFHRRVFVKLFSSRALREHMAPIIQKH
VRVLQHVLMGATKTMQAVDMFTYSGRFTLDAFGEIAFGFNMSTLTLENEHPFERAXVDSQHIVAARLVVPTWYWKLK
RWLNVGSEKRLREALTTVDEFVMDVISKTMEKRNAPNVNKADIKPRDIVSLVLDDETVDGKEADPTLVRNVVLMALIA
GRDTAADALAWLFHLLTLN

>CYP5014H1 P. sojae

MLRKWLTKHRALSPLGPAGLALLAGAAVVAAYVATRSSGDAVSVLDCKEEKTKEKWENTPQDKPKVVPYLPS
KVPWIGNMLQLAGNAHRFHSWMAEQCIAHNGVFKLHLPGQSDMLVTAVPEHYEHVVKTQFEHFSKGHQQYDMFV
DLMGHSVLIIEGERWKYHRRLLVRLFSARALRDHMTPVIQRHTLLLQNVFLKAAVAKKPVDVYMFMHRFTFKAFAEMV
FNNSLDSIDSEHEHPFEQAFDEAQSIVAGRLQQPVWFWKLMRWLNVGLERKLREDVALIDEFIMEIISTAIEARRQRQE
DLKAGRPVKDADKDIVSIVLECMEQDGDMVSPTDVRNIAVAALGAGRDTSADAMSWLLHTLTQNPHVEDKLRAELLE
NLPKLATSPSYVPSMDEVHGLVYLEATIRELLRLQTPVPFTLRECIHDTVFSDGTFVPKGTNVGMCHFGAARRPEVWGP
DAAEFNPERFIDQETGKLVQTPMAKFNAFSGGQRMCVGKALAMLEMKLVIATLVGRFHFREVPGQNVQYAMGITIG
MRNSLMMHIEPVRTGASAAAA*

>CYP5014H1 P. ramorum

MLRQWLVKHRVLSPLGPAGLVLLAGAAAVAAYISTRSSGVEVKEFDLKEEESTDKPRVVPYLPSKVPLLGNML
ELAGNAHRFHSWMAEQCVIHNGVFKLYLPGQSDMLVTAVPEHYEHVVKTQFEHFSKGKQQYDMFVDLMGHSVLIIE
GERWKYHRRLLVRLFSARALREHMTPVIQRHTRMLQTVFLKAAVAKKPVDVYMLMHRFTFKAFAEMVFNNTLDSIDS
EHEHPFEQAFDEAQSIVAGRLQQPVWFWKLKRWLNVGLEHKLREDVALIDEFIMSIISTAIETRRRRQEDLKAGRPVKP
ADKDIVSIVLECMEQDGDMVSPTDVRNIAVAALGAGRDTSADAMSWLLHTLTQHPEVENKLRAELLEKLPKLAVSATY
VPSMDEVHGLVYLEATIRELLRLQTPVPFTLRECIHDTVFPDGTFVPKGTNVGMCHFGAARRTEVWGPDAAEFKPERF
VDPVTGKLLHTPMAKFNAFSGGQRVCVGKALAMLEMKLVIATLVGRFHFSEVPGQDVQYAMGITIGMRSSLMMNV
QPVIRGAAGAAA*

>CYP5014H2 P.infestans

MLRRWLVKHRALSPLGPAGLVLLAGAAAVVAYVATRSSQNEVKADTSEEESVDKPKIVPYLPSKVPVIGNML
QLATNAHRFHDWMAEQCIAHNGVFKLYLPGQSDMLVTAVPEHYEHVVKTQFDHFSKGHQQYDMFVDLMGHSVLII

EGERWKYHRRLLVRLFSARALREHMTPVIQRHTLLLQKVLLKAAIAKKPVDLYMLMHRFTFKAFAEMVFNNTLDSIDSE
HEHPFEQAFDEAQSIVAGRLQQPVWFWKLKRWMNVGQERKLREDVELIDNFIMKIISTAIETRRQRQEDLKAGKPDG
DMVSPTDVRNIAVAALGAGRDTSADAMTWLFHTLTQHPQVENKLRAEIFDKLPKIRTSSTYVPSMDEVQGLVYLEATIR
ELLRLQTPVPFTMRECIHDTVFSDGTFVPKGTNVGMCHFGAARSTEVWGPDAAEFKPERFIDPETGKLLRTPTAKCNAF
SGGPRVCVGKALAMLEMKLVIATLVGRFHFTEVPGQNVHYAMGITIGMRSSLMMNVQPVTGGAPGAAA*

>CYP5014H3 P.parasitica

LRRWLAKHRALSPLGPAGLVLLAGAAAVAAYVATRSSENDVKVDSSEEECTIDNPKVVPYLPSKVPIIGNMLQL
ASNAHRFHDWMAEQCIANNGVFKLYLPGQSDMLVTAVPEHYEHVVKTQFDHFSKGHQQYDMFVDLMGHSVLIIEG
ERWKYHRRLLVRLFSARALREHMTPVIQRHTLLLQKVLVKAAVAKKPVDLYMLMHRFTFKAFAEMVFNNTLDSIDSEH
EHPFEQAFDEAQSIVAGRLQQPVWFWKLKRWMNVGQERKLRQDVELIDNFIMKIISTAIETRRQRQGDLKAGKPVKK
ADTDIVSIVLECMEQDGDMVSPMDVRNIAVAALGAGRDTSADAMSWLFHTLTQHPQVENKLRTELFEKIPKLTMSST
YVPSMDEVQGLVYLEATIRELLRLQTPVPFTMRECVHDTVFSDGTFVPKGTNVGMCHFGAARRTEVWGSDATEFKPE
RFIDPETGKLLQTPAAKNNAFSGGQRVCVGKALAMLEMKLVIATLVSRFHFTEIPGQNVQYAMGITIGMRNSLMMNI
QTVTSGGPGAAA*

>CYP5014H4 Phytophthora capsici

MMRQWFAKHRALSPLGPAGLVLLAGAAVVATYIATRSSESETLEPEEEEEELQVKAKVVPYLQSKVPVIGNML
ELFKNSHRFHDWMAEQCIAHNGVFKLYLPGQSDLLVTAVPEHYEHVVKTQFEHFSKGHQQYDMFVDLMGHSVLIIEG
ERWKYHRRLLVRLFSARALREHMTPVIQKHTLMLQRVLLKAAVSKKPVDLYMLMHRFTFKAFAEMVFNNSLDSIDSEH
EXGTPSHDRLLFVRMESYERGAGRPVPKADKDIVSIVLECMEQDGDMVSPVDVRNIAVAALGAGRDTSADAMSWLM
HTLTLHPLVEKKLRAEILENLPKFTTNPTYVPSMDEVQGLVYLEATIRELLRLQTPVPFTMRECIHDTVFADGTFVPKGTN
VGMCHYGAARRTEVWGSDALEFKPERFIDAKTGKLVQTPSAKFNAFSGGQRVCVGKALAMLEMKLVIATLIGRFHFVE
VPNQDVHYAMGITIGMKNSLMMTVEPVARGTVGAAA

>CYP5014H5 Phytophthora capsici

MWSRNALAIWSKSSGRAENSQSFLPEVPVIGNMLELFKNSHRFHDWMAEQCIAHNGVFKLYLPGQSDLLVT
AVPEHYEHVVKTQFEHFSKGHQQYDMFVDLMGHSVLIIEGERWKYHRRLLVRLFSARALREHMTPVIQKHTLMLQRV
LLKAAVSKKPVDLYMLMHRFTFKAFAEMVFNNSLDSIDSEHEHPFEQAFDEAQSIVAGRLQQPVWLWKLKRWMNVG
LERKLREDVALIDEFIMKIISTAIETRRQRQEDIKAGRPVPKADKDIVSIVLECMEQDGDMVSPVDVRNIAVAALGAGRD
TSADAMSWLMHTLTLHPLVEKKLRAEILENLPKFTTNPTYVPSMDEVQGLVYLEATIRELLRLQTPVPFTMRECIHDTVF
ADGTFVPKGTNVGMCHYGAARRTEVWGSDALEFKPERFIDAKTGKLVQTPSANCCPWGNFSLMRTASTSSSCFSAPL
ER

>CYP5014H6 H.arabidopsidis

MHLRQWYLNYRAGAPLRSAGLVLLAGAAVVAVCVSARSTPIKLPCNPKENEDKDEEDKDPVIIQPKEVPYLPS
KMPLLGNMLDLASNAQCFHDWMAAQCIAHNGAFRLYLPGQSDMLVTAVPEHYEHVVKTQFHHFSKGQQQYDMFV
DLMGHSVLLIEGERWRYHRRLLLRLLSARALRDHMTPLIQRHTLLLQQVLMKAVSTNQPVDLYMLLHRFTFKAFAEMV
FNNSLEGIDSELEHPFEQAFDAAQSIVAGRLQQPVWLWKLRRWLNIGQERKLREHLKLIDAFIMDIISVAIEKQRERQQD
LQAGNPIEKTDQDLVSIVLECIAQDGDMVSPIDVRNIAVAALGAGRDTSADTMSWLLHTLTQHPQVETQLRAEVLDKV
PTLALDASYVPTMDDVQGLVYLEATIQELLRLHTPVPFTMRECIRDTVFPDGTFVSKGTTVGMCHFGTARRTEVWGPD
AKEFKPERFIDPETGKLRHLPAAKLNAFSGGQRACVGKALAMLEMKIVIATLVARFHLVPVPGQNVQYAMGITIGMRTS
LMMNIEAVEANQTGQEAAAA*

>CYP5014J1 P. sojae

MPPPSLLKGGGLSSPALLGLLVATAAAMLFAAKPSRGKPLPANVTPVPFLPSTPLLGNTLELAANAARLQDWV
ADRSRECDGQPFVVQLLGKRNLVYLSRPEHFEQVLKLQSSNFNKGLAIHDIYSDFMGESILLVNGDRWKYHRRVLVNLF
SARALRDFMTPIIQKNILVLMDILARARERNEALDIHKLMNKFTFETFAKIGFGQKLGNLVSPEDHPFERAFDEAHHITGH
RMTTPTWLWKLKRWLNVGSERKLRECVEVMDSLVMGIISDAIAKRQQRGQEEEAGEHDHEKDIVSIILEMHADGRPV
EPSEVRSIALLSLIAGRDTTANAVSWILHMLHEHPRVEEKLRAELYEKLPKLATSRDYMPSLEELQDLPYLEAVINENLRLL
PIFPYTSRQCIRDTVFPDGTFIQAGEVLGLPHYVMARLTSVWGENAAEFVPERFLDAKSGEVLDLPVATSSAFGAGPRIC
VGRRLASMEMKLLLACIVGRYHLVELPGQTVRYKLALSLTMKDPLMVNVQHVNQALAKSA*

>CYP5014J2 Phytophthora capsici

MLLLSSHQPSLLVSSILAAAVVATIYASNAKHLGDKKELPGGEAPIPVSYIPNGLPILGHTLDMIHNADRFLDWL
LELSESQDGKPFVLHLLGRSDLLIDAGPEHHEEILKTQFDNFIKGDQFYDMLVDVAGPNVVIIEGERWKYQRKVLVSLFSA
RVLREHMMPIVQKHTQVLLGIFEDAAVQQRPFEINKLLHRFTLDSFAEIGFGSKLGSLQSGQEHPFGKALDDALHIAAAR
RMVPKWLWKIKRWLNVGSERKLRESVEVMDALVMGVISTAVKHHEYEEERTGRDIVSIILDRMRADGQAVELSEVRSI
ALLALIAGRDTTANAVCWVLHMLHEHPHVEEKLRAELLQKLPKLLTFEYVPSLEELQDIPYLDAVINENLRLLPIFPYTSRQ
CIRDTVFGDGTFIPAGSVLGLPHYVMARLKSVWGENAAEFVPERFLDPHTGDVLELPVAASAAFGAGPRICVGRRLAT
MEMKLLIACIVGRYHLEELPGQTVRYKLALSLTMKDPLMVRVQRVRAKPA*

>CYP5014K1 P. sojae

MLSLSELRTHPLVVGFVAVAAAVTLYSVVANAGSDALDDEDDEGKTDRKAKPIPYLPGGHPVLGHTLLMARN
LDRFQDWLVETSVARGGAPFVLRQPGKNDWLFSARPEDFEQILKVHFDTFIKGPQVRELLDDFMGENIVIINGHRWKF
QRKALVNLFTARALKEHMTPVVQKCALALQRVFAKAAESGDVLDVHHIMGRFTLETFAEIEFGSQLGLLEKGEENAFET
AIDDANHISLERFAVPMWVWKLKRWLNVGSERRLKEDMAVISSFVMSCISDAIERRKQRLEAAARGEPVGPVAKDIVSI
LLDSEDATGEPVLPKDVFNISLAGVLAGKDTTGDATSWLMHLLHENPRVENKLRAELLAKVPKLAEDESYVPPMEELDAI
TYLEATIRESLRLKPPAPCVTQHCTQDTVFPDGTFVPKGMDTTLLYHASALLPSVWGPDAAEFNPERFLDDNGKLLVLPP
LKFIAFSAGPRKCVGRKLAMIEMKVVTACLVSRFHLVEVTGQDIRGTMGISLGMKNGMKVSVQATPGVAKRA*

>CYP5014K1 P. ramorum

MTPPPPHHHHGGHQTSFAFVHRPVTMLSPSAVLTHPLVVGLLTAAAVAALSSVATSYAADADSGDEDTKPV
KPVPYLPGAHPVLGHTLLMAKNLDRFQDWLVETSVARNGEPFVLRQPGKNDWLFSARPEDFEQILRVHFDTFIKGPQV
RELLDDFMGENIVIINGHRWKFQRKALVNLFTARALRDHMTPIVQKCALALQRVFAQAATSGEAIDVHHVMGKFTLET
FAEIEFGSQLGLLESGQEHAFETAIDDANHISLERFAVPMWVWKLKRWLNVGSERRLREDMAVISSFVMSCISGAMER
RKQRQEAAARGEPLGPVAKDIVSILLDSEDTIGEPVLPKDVFNISLAGVLAGKDTTGDATSWLMHMLHENPRVENKLRT
ILLAEVPKLAVDESYVPSMEELDGVTYLEATIRESLRLKPPAPCVTQHCTQDTVFPDGTFVSKGTDTTLLYHASALLPSVW
GPDAAEFKPERFLDENDKLLVLPPLKFIAFSAGPRKCVGRKLAMIEMKVVTACLLSRFHLVQVPGQDIRGTMGISLGMK
YGMKVNVQRTPGVAIRA*

>CYP5014K2 P.infestans

MLSLSNILSHPVVKVKPVPYLPGGHPILGHTLLMARNLDRFQDWLVEASVARNGAPFVLRQPGKNDWLFSA
RPEDFEQILKVHFDTFIKGPQVRELLDDFMGENIVIINGHRWKFQRKALVNLFTARALRDHMTPIVRKCASALQRVFAK
AAKEDGVLDVHHIMGRFTLETFAEIEFGAQLGLLESGEDHAFETAIDDANHISLERFAVPMWVWKLKRWLNVGSERRL
REDMDVISTFVMSCISGAIERRKKRLEAAARGEPLGPVAKDIVSILLDSEDTIGEPVLPQDVFNISLAGVLAGKDTTGDAT
SWLMHLLHENPRVENKLRTELLAQIPKLAIDESYVPSMEELDAVTYLEAVIRESLRLKPPAPCVTQHCTQDTVFPDGTFV

AKGTDTTLLYHASALLPSVWGPDAVEFNPERFLDENEKLIVLPPLKFIAFSAGPRKCVGRKLAMIEMKVVTACLVSRFHLV
QVPGQDIRGTMGISLGMKYGMKVTVEPTPGVAAA*

>CYP5014K3 P.parasitica

MARNLDRFQDWLVETSVARNGEPFVLRQPGKNDWLFSARPEDFEQILKVHFDTFIKGPQVRELLDDFMGEN
IVIINGHRWKFQRKALVNLFTAKALRDHMTPIVQKCALALQRVFAKAAKEDGVLDVHHIMGRFTLETFAEIEFGAQLGLL
ESGKDHAFETAIDDANHISLERFAVPMWVWKLKRWLNVGSERRLRQDMEAISTFVMGCISGAIERRKQRQEAAARGE
PLGPVAKDIVSILLDSEDTTGEPVLPQDVYNISLAGVLAGKDTTGDATSWLMHLLHENPRVENKLRAELLAKIPKLAVDES
YVPSMEELDAVAYLEATIRESLRLKPPAPCVTQHCTQDTVFPDGTFVAKGTDTTLLYHASALLPSVWGPDAAEFNPERFL
DENDKLIVLPPLKFIAFSAGPRKCVGRQLAMIEMKVVTACLVSRFHLVEVPGQDIRGTMGISLGMKYGMKVTVEPTPG
VAAA*

>CYP5014K4 Phytophthora capsici

MLSPSSLFTHPIVVGIVVAAAGAALVSYSNSYASDPEDDEEKAKPIPYLPGGLPILGHTLLMARNLDRFQDWLV
ETSVARNGEPFVLRQPGKNDWLFSARPEDFDQILRVHFDTFIKGPQVRELLDDFMGENIVIINGHRWKFQRKALVNLFT
ARALRDHMTPIVQKCALALQRVFAKAAEEDDVIDVHHIMGRFTLETFAEIEFGAQLGLLESGEDHAFETAIDDANHISLE
RFAVPMWVWKLKRWLNVGSERRLRENMAVISSFVMNCISGAIERRKERLEAAARGEPLGPVAKDIVSILLDSEDQTGE
PVLPQDVFNISLAGVLAGKDTTGDATSWLMHLLHENPRVENKLRAELLARIPKLAMEESYVPSMEELDAIAYLEATIRES
LRLKPPAPCVTQHCTQDTVFPDGTFVPKGMDTTLLYHASALLPSVWGPDATEFKPERFLDENDSNKLLVLPANKFIAFS
AGPRKCVGRKLAMIEMKVVTACLVSRFHLAELPGQDIRGTMGISLGMKYGMKVMAEPTPGVVPRAA

>CYP5014K5 H.arabidopsidis

MASRRYASDPDSGHDHETKEEPTCSVPYLPGGHPLLGHTLIMARNLDRFQDWLVETSVARNGQPFVLRQPG
KNDWLFSARPEDFEQILSVHFDTFIKGPQVRDLLHDFMGENIVIINGERWRLQRKALVNLFTARALRDHMTPVVRKCAL
ALQQVFASAAKTQGVVDVHHLMGRFTLETFAEIEFGAQLGLLASGRDHAFETAIDEANHVSLERFAVPTWVWKLKRW
LNIGSERRLRQAMAVISTFVMTCISGAIKRRSERRAALARGEILGPVANDIVSILLETEDKTGEPVLPTDVFNISLAGVLAG
KDTTGDATSWLLHLLHENPRVENKLRTKLLAHIPKLALDETYVPTIDELEGITYLEATIRESLRLKPPAPCVTQHCTQDTVF
PDGTFVAKGTDTTLLYHASALLPSVWGPDAAAFNPERFLDDDDKLVVLPPLKFIAFSAGPRKCVGQKLAMIEMKIVTAC
LVSRFHLVQVPGQDIRGTMGISLGMKNGMKVTVHPTFGRATSA*

>CYP5014L1 Phytophthora capsici

MLSVSQLQNVKSSSVLLGLLTAAAVSTTVYSIWKSSNNAQDRKILRVSFLPSIPLLGNTLEVAANGSRMLDWEL
ERIKERDGKPFAVSIVGKNDLIFTANPQHFEQVLKVQSSNFNRGASSHDMYADFMGESILIVNGDRWKYHRKVIVNLFS
ARALRDVMTPIIQKNIGVLVGILTKASENGEALDFHKLMNKFTLETFAEIGFGRKLGNLASPDDHPFEKAFDEAHHISAM
RLMSPTWLWRLKRWLNVGTERRLRESMVVINKFLMETITSMMENRASNDQSNKDILSIIFDTMESSGQTITPTDIRDIV
FTGMIAGRDTTADALAWLMHMLHLNPRVVKKLREEILTKLPKFAESETYVPSMEDVQGLVYLEATIRELLRLFPTAPHV
GYYCLRDTVFPDGTFIPADTTVMISLYVSARLESVWGADAASFVPERFIDKDTGGVLQISSLKFASFSAGPRVCVGRNLA
MLELKLVTACLVSRFKLEEVSGQHVIYTRGVTIGMKNPLMMKAEKVPAAESH*

>CYP5014L2 P.parasitica

MLPPSLLQGDDKLTFSSSFLLGLLTVTAAATTLYTITSRKSNNLKGNAEHITQVPYLPSTLFLGNTLEVVANTARF
QDWISDHSQERNGQPFAVRLLGKNDIIYMAKPEHFEQVLKEQSSNFNKGPTMHEVYSDFMGEGILLTNGDRWKYHR
RVLVNLFSARALRDFMAPIIQKNVQVLTEVLSRACDTKELVDLYKLMNKFTFETFTEIGFGRKLGNLASPDAHPFELAFDE

AHRISGYRFTTPSWLWKLKRWLNVGTERRLRDAMVVVDEFLMGTIIGTMERRQLDSTVNRDVVSIILDTMETSGQTM
TPGDIRDIVFAGMIAGRDTTADALSWLMHALHNNPRVVKKLREEILAKLPKFAESECYVPTMDEVQELPYLEATIRELLR
LSPAAPTIPYQCVRDTVFPDGTFIRAGCGVMLSLYSAARLESVWGPNAAEFAPERFIDAETGDLVQISSTPFAAFSAGPR
VCVGRTLAMLELKLVTTCLVGRFRLEEAEGQNVTYSRGVSLGMKNPLLMKVARVPIEMS*

>CYP5014L3 P.infestans

LLPSVLGADDKFGPIRLGLATATAVVTFFSVCKKSPPPDGVIPVSFLPGIPLLGNTLEMVANIPRIQDWISDQFQ
ERNGNPIGARLVGKNKLIYLAKPEHFEQVLKTQSSNFSKGIDVHVVFSDFMGNGILLVNGDRWKYHRKVLVNLFSARA
MREFMTPVVQKNVHVLMEILSNASDTGKEIDIYRLMNKFTFETFTEIGFGQQLGNLKSIEDHPFEVAFDEAHYICTERFS
YPVWLWKLKRWLNVGSERLLRDSMVVINEYLMETISTAMKRREQGHSRSNYVPSMDEVQDLPYLEATIREVLRLVPSV
PIIPYHCYHDTVFPDGTFIPADSAVLLSTYSTARLECVWGPDASSFVPERFLDPKTGELLKMSSTEFVAFSAGSRVCVGRN
LAMLELKLVLSCLVSYFRLVEVNGQDVTYGNGVTIRMKNPLMMKVETVSNTSA*

>CYP5014L4 P.infestans

MGEGVLLTNGDRWKYHRRVLTNLFSARALREHMAPVIQRNVQVLTEALYRVSDANELVDFYKLMHKFTFET
FTEIGFGRKLGSLASPDTHPFEVAFDEAHRISGHRFTAPVWLWKLKRVLNVGTERRLRDAMAVIDKFLMGTIVGAMER
HQHGDRSAKRDIVSIILDTMETSGQRITPGDIRDIVFAGMIAGRDTTADALSWLMHTLHNNPRVARKLRKEILAALPQF
AESESYVPSIFHFTSVKLMEVSGKPLMLKDVSSMIAGMRRNSYTSPEDNVRVTEVLQDFSEEPGIVVNVFRDADTKLTSC
ITFQTAHMRRMARQFPDVMCVDATHGTNISSGAFAQYALIDGEPQENMKSSITALKRNNGK*

>CYP5014L5 P.parasitica

DDHPFEVAFDQAHQICSERFSYPVWLWKLKRWLNVGSERLLRDYMVVINQYLMETISSAMNRRSTHEGDN
QPVNKDIVSIILDSMEKSGQTITPSDIRDIAFAGMIAGRDTTADALSWLMHELHHHPRVLENLRQELTTKLPKFGDSESY
VPSLDEVQDLPYLEATIREVLRLVPPVPTIPYHCYRDTVFPDGTFIPADSAVLLSTYSTARLGSVWGPDASSFVPERFLDAN
SGELLKMSTTEFVAFSAGSRVCVGRNLAMLELKLVVSCLVSYFRLEEVSGQDVTYGSGVTIRMKNPLMMKVEKVSSSGC
*

>CYP5014M1 P.irregulare

TAAEDLGRIDWQLVLRVVLGAALLALVLYLLHWLGQKQYPAPLIDLKTSSKRLEEPESTLPFFKNTLDFSRHEAH
IHDWIASQCERFQGRPWKFKILGSPPAIVFSDPRAFEDILKTQFWRFEKGADLHEVLHDFIGDGIFSVDGEQWLRQRKT
ASHLFTLRELRDTMVAVIQQHTTRLCKLLDKYADEDTQFDLFQLLNRFTMSAFCEIGFGVDMDCFEPKEGSRHAFPVAF
DRVQQVTQLRLVRPRWFWKLQRLLNVGAERMLRSDMQVVNDMVLRIVNDSLQQQQNSSKDQKTIVSLFLDKCVEFS
GQSEVDSKLLRDVVLNFLLAGRDTTAQTMSWLLLCLSEHPHVETKLRQELVDNLSPESTKVVKLDQVQSLVYLDAVIHET
LRLYPPVSFNQKMAVHDSVLSDGTLVPKGTNAAFPSYALGRMTSVWGDDAKQFKPERWIDPETGTIRKESSFKFIAFH
AGPRQCLGKNLAMMEIKIVLATLLLHFQFQVEKRAKGEVKYGTSLTLPMKTPLCTRVTKSFTL*

>CYP5014M2 P.iwayamai

ASDDNLQRIDWQILLRGLLGAAIIVLVLFVLHWLRQRNTDERDEEAKLGTARRVLEEPASTLPIFKNTLDFVRHE
DHLHDWICEQCERFDGRPWTFKVLGQPRTIVFSDPQAFEEILKTHFWSFEKGEGLHEILFDMMGDGIFAVDGETWTM
QRKTASHLFTHRELRDTMAAVIQGHTATLCQVLDRFADDKKQFDLFALLNKFTMGAFCEIGFGVDVNYLEDENATHPF
QVAFDRAQQVTRLRMNRPRWLGKLQRLLNLGAERTLRADMPVIDDMVYKIIRESLQAQKNPSLATQKKTIVSLFLDDP
VKLPSETEAHPVDAKFLRDIVLGFLLAGRDTTAQTMSWLFLCLSENPDVEAKLRHELEQKLPETVGAGTEVAALDQLQSL

VYLDAVLHETLRLNPPVAFNWKIAVRDCVLSDGTLVPNGTYAAFPSYALGRMTRVWGDDAKQFKPERWIDPETGSVRVESSFKFIAFHAGPRQCLGKNLAMMEMKIVVATLLSRFQIQVAKRKAGEVKYGMSLTFPMNTPLLTRVKRK*

>CYP5014N1 P.iwayamai

VHVDFAVVLGLAVAAKAFFALYEWLTTSSGLKRSLALPRPKGTLPIVGNTLQTAQHFHRIHDWFLEQSELHGGNSFYLSLIGAQHLLVVAKPKFFEDINKIHLERFDKGPLYADLMRDVLGEGIFAVDGDKWRQQRKTVSNLTVSVHDNLVLFRKLLQRAAKSHERVNLVKYFFRFSTETFVNFGLGAKIGVLDANEDHPFQVAFDKVKRDIVSLFIESFNHNNPDETFEPAVLRDIIVNILVAGRDSTAVTMNWFFYLLSEHPHVEAKLIEELLEHAPELMRGDVDALGSEDLQKLVYLEAALREITWLFPAIPINIKTDSKNVVLHNGTFVGAGWGIVYASYTMGRMTSVWGPDALEFKPERWINPSTGKLITFSPYKFTPFHSGPRVCLGMTMAIMKMRIVIASLLSQFRVRVVPEQRVTYDFALILQMKDPLLVTVEPAAHRLAPAANTIAPAATECVIRIR*

>CYP5014P1 P.iwayamai

ATAFVVAMLLIYKTRGSQPHKAIAHSPDSLPILYDMLSLIKHRDDLLDWVALQNELLGGRTWLLQMVLGDSPTVVINTPELIEDVLVTHFENFGKGEYVSENFRDLLGDGIFAVDGLKWIHQRKTASNLFSHRDLKETMDQTVSHQVQALTRILEHHATDGTPVDIFRLMNRFTLDVFAEIGFGVKMGTLEGKDTIDSQCSEGVSFESAFDEAQKLVVMRFIKPCWLWKTQRYLNIGSEKQLKACVKVIDDTVLGIIEQSLKNRQDMLTSQRVKGSLSDPGRPGGRKDIVPLFLDYVSNDTKPQSDTKFDPKFLRDVVVNFLIAGRDTTAQALSWFFYCLSQNPHAEQKIREELAAKLPGLANASPSMEQLHQFVYLEAAIKETLRLYPSVPDSTQQCLKDTLLCDGSFVKAGMCIAYPGYSLGRMTHVWGPDAKEFKPERWIDPSTGKLIAISAFKFNAFHAGPRMCLGMNMALVEMKTVAAVLLRQFRFSLVPGHEVTYMLSLTLPMKNGLSMHVSRSTPSAH*

>CYP5014P-fragment1 P.irregulare

MHFENFGKGELVTDSFRDLLGEAIFTVDGLKWLHQRKTVSNLFSNRELRDFMEATVRQQVHELTKILQRHTTDNGSKQRGQPIGMFRLFNRFTFEVFAEIGFGVKMGALEEDEDNSGVPSDQSFQSAFGNAQRLITNCFMVP

>CYP5014P-fragment2 P.irregulare

FNLFNQFTFEVFTEIGFGIKIGDSKDEAVTPVQAAFNAAQPLLIWRFLEPSWVSKTKRYINVNTERKLKSCIQLIGDTVYGIISQSMENRLKPESSTTDGRKDIISLFLDEVSYDIKSEPGSKTAGNASLDPKFLSDVVXXXXXXXXXXXXXTLSWFLYCLSQNPTTGQRVREELASRLPEVGGAAGREEAFAPSMDQLHQLVYLEATIKETLRLYPSVPDNRKTALRNTVLCDGTFVKVGMNVGYPGYVLGWMTRIWGPDAKKFRPEHWIDSETSKLTPVFTSKFNVFHVGPRMCLGMNLVMMEMKTVAAVLLSKFRFELVPGQEI*

>CYP5014Q1 P.iwayamai

MASEWLAAAMATSDWQQVGVTLVALYFSSRVVAQLYARATMSDAERNAPHVPRPKGTLPFLRNTLQALAHNADFHDFWAGLSDEYDGATVQVKMLGRPEIISVATPELFEDVLKTHFESFAKGPYLNSVTSDLLGNGIFAVDGPQWVRQRKMVSNLFSLRMLRESMAETVHKHVQVLNRVLQRHGDSKTPADIANLMSRFTIETFGEIGFGVNLESLETVEEHPFAIAFDSVQRLLVERFLRPPWFWKTQRWLNVGVEGELKQNIRVIHDTVLEIIDKSLKNRKSRQLKEPENKKDIISLFLDNASYDGADENDKSNPELLRDIVVGFLIAGRDTTSQALSWFLYNITHHPDVEKKIREELTKELPGLMRGEIESPSMEQSHKLMYMEAALNESLRLHPPVPGNLKQAMKDVVLSDGTFVKAGWSVSIATYTLGRMKGVWGPDVREYKPERWIDSATGKLIPVPAYKFSTFNAGPRMCLGMNLAMMEVKIVVASLLAKFRLELVPNQTVVYDFSVSLPVKGALLMNILPVDAKKTA*

>CYP5014Q2 P.ultimum

MFTSVWHEITMAGDWRNAATALLAVLFGAKVAANAYARAVMSDAERKAPRVQRPKGSLPFLHETLQAMAHSADFHDYWTTLSEDAEGRSVHVKILGHPDTISVATPELFEDILKTQFESFEKGPYFKSIVSDLLGDGIFAADGQVWVHQRKTASNLFSLRMLRESMAESVQKHITVLYRVLLRQGVTEKQPVDLAELFNRFAIETFAEIGFGVDLKCLDTPEEHPFAQAF

ARVQKILIVRFLRPPSFWKLQRWLNVGMEHELTRNIRVIHDMVLDIIEKSLENRSLPDYKFDETKKDIISLFLNHTSLDGSR
SAPGDEGSVDPRFLRDIVVNFLIAGRDTTAQALVWLMYNVMRHPEVGSKIRQELEEQLPELVRGKIESPSMEQAQKLV
YLEAAVRESLRLHPPVPGNMKQATKDVVLCDGTFVKKGWRVSISPYTLARMES*

>CYP5014Q3 P.irregulare
MTSEWRFPAVITSDWMQIVSMLAGLFLASQVVTQLYGRLAISSLEKNMPRVSRPQGTLPILRNTFQFLKYSG
DFHDFICELSEEAGSRTVQVKILGRPEVLVLATSELFEDVLKTHFESFMKGPYLVSVFSGLLGNGITVVDGHKWEHQRKTT
SNVFSMRALRESMAGSVYKRIKVLDRVLQRHADAQTPVDVANLVNRFTIETFAEIGFGIDLNNIKSVENHPFAATFDRT
HIRVIHDTMLDIIVQSLENRRPRDCRNKDDAKKAPFAENAKKDIAFLFFDSVSYDGPNAGDKFGPDLLQDIVMNFLLAG
RDTASQGLVWFLYNLNQHPEVEKKIREELARELLDLMSGGIEAPPMEQS*

>CYP5014R1 P.aphanidermatum
CDNLHDVLGNGIFAVDGVKWMHQRKTASNLFTMRTLRDSMAHTVQDLMPAIDRILQRVVDSGEPIDLVRLL
NRFTMEGFSKIGFGIDLNLLESEHEHPFMAAFDSAQRITTLRFVRPRFVWKILRWLNVGGERELKKNIETINNTVLGIISQ
SLQRRQESTCNHPDTHSKGKDIVSLFLDDVSHNEKHEQEPFDPAFLRDIVVNFLIAGRDTTAQALSWFFYELSENPEVES
RLRVELKAVLPRLFQRDAAPPSMEEVQKLTYLEAVLKETLRLHPSVPTNFKRANRDTVLVDGTFIAKGWSVAVSSYCLGR
AEHVWGPDAKKFRPERWIDPPTNPNKLIAVSAFQFNSFHAGPRMCLGMNLAMMEMKIFVS*

>CYP5014R2 P.aphanidermatum
MEMALDLSPATPSAVATVLVLVLVVSLAWAALTTAARKKPVGAVPQLAPLAPTGIPILHHTFEVIKNAPRAHD
WLNDFCQQFDGKAFRIKVGGRVNITMMTTPELFEDVLKTQFSCFDKGELMCDNLYDLLGNGIFAVDGVKWMHQRKT
ASNLFSQRTLRDSMAHTVQELVPVLHKIFLRAEGNDESIDLVKLLNRFTMEAFSKIGFGVDLNCLDAEDDHPFMAAFDS
AQRCITYRFVIPRPLWKIKRWLNIGTERERKKQVKVINDTVLTIISQSLEMRQRRGSTPATPPTKDIVSLFLESVASHDSSE
HDAFDPVFLRDIVVNFLIAGRDTTALSLSWFFYELSQNSHVEKRIREELEEILPDIFKVGSQPPGMEQVQRLTYLEASLKET
LRLHPAVPTNFKSANHDTVLVDGTFIPAKSVVCISPYCLGRAKHVWGPDASDFRPERWIDPADPKKIIPVSAFQFPAFHA
GPRICLGMNLAMMEMKILVASVMSKFRVEIVPDQSIIYDLSLTLPIKGQLLARVHSL*

>CYP5014S1 P.irregulare
WSLPTFQSDWDRHAGAGLLLLLAGGLAGRWAFEKLGPSKKPATQLRLRPQGTLPIFYNTLHAMKHGPRWA
DWITEQVESFKGQAFWLRILGTPDMLVLTTAEQFEDVLKTHFDVFEKGPYIIDIFEGLFGAGIFAADGKQWTHQRKTAS
NLFSLRALRESMTESIQKHALVLNGVLDRTAALKSSVDVFKLLNRFTMDTFAEIGFGIELGTLESTEDHPFQVAFDSAQHI
LVLRILRPSWFWKTQRYLNMGVEKEFRQHLRVIDDTVFTIIARTLENRAASQSQSQSSQQHGTRKDIVSLFLDNVSADA
DEDKTQAAEAFDPVFLRDIVLNFLIAGRDTTAQMLSWFFYSLSQHPHVEQKIRDELAHKLSNLNQGKGKVATTPPSME
QTGELVYLEAALREAMRLHPAVPRNRRQANRDVTLRDGTFIRKGEAVSISSYVLGRMTHVWGDDAKEYNPERWIDHQ
SGKLIQVSPFQFPAFHAGPRMCLGMSLAMVEMKIVAAMVLSKFHLELAPGQTITAGLSLTLPIKGALMMNVLPVVG*

>CYP5014S10 P.iwayamai
MLDALSLSTHPLVSIALLLAAVGFAQWLYEVATRPTVAKSARPREWPEWTLPVFRNTFQTIIHAPHMYDFLT
MHAERFKGQPYWVSLIGGPAAAVLSTPEQFEEVLKTHFETFDKGDMMTENLRDLLGNGIFAVDGDKWVRQRKTASN
LFSLRSLRESMTESIHKHALVLTAILQRSSQQQPIDLFNLMNRFTIETFAEIGFGVELGSLDSEKDHPFQAAFDSAQRVLVY
RFMRPAWFWKTQRYLNLGSEAGIKRNIQVIDDMVLGIIAQSLENRRSQSHISLSTLSGKKDIVSLFLDSVENSNGGDDV
GHEEAFDPVFLRDIVVNFLIAGRDTTAQALSWFFYCLSQHPEVERKIREELMAKTPEIFSGEIATPSMELSQQLVYLEAAL

RETLRLYPSVPVNIKQANKDVMLSDGTFIRKKEDAVISSYVLGRMSHVWGEDAKEFKPERWLDPVTGKLVAVSAFKFSA
FHAGPRMCLGMNLAMMEMKIVAAMVVSKFRLALVPGQKVTYDLSLTHPIKGELLMTVVPVTSCS*

>CYP5014S2 P.irregulare

MWSLFSFQSFWEHAGVGLLLLLAAVFAGHWAFEQLVSSSSSKKQIVQPQLRPKGTLPVFYNTFHAVKHGHR
WAEWITEQVEAFDGKPFLLRILSNPDMLVLSTPEQFEDVLKTHFDAFEKGPFKISVFHGLLGAGIFAADGKQWAHQRKT
ASNLFSLRALRESMTESIQKHALVLNNIFERTAEATQPIDVFKLLNRFTMDTFAEIGFGVQLNTLEATEDHPFTVAFDSAQ
RILVLRMLRPSWFWRTQRLLNIGVEREFKQHIRVIDDLVLAIITRALENRASQSQQQQQGAHKKKDIVSLFLDNVSSDE
KSQADAFDPVYLRDIVVNFLIAGRDTTAQMLSWFFYLLSQHPQVETKIRDELASKLPELYESKISTPSMEQTGELVYLEAA
LRETMRLHPAVPRNSRSANRDVTLRDGTFVRKGQTVSVSSYVLGRMTRVWGDDAREYKPERWLDPQSGKLVHVSPF
KFPAFHAGPRMCLGMNLAMVEMKIVVAMVLSKFYLTLVPGQTITDGMALTHPIKDALMMNVLPVAK*

>CYP5014S3 P.irregulare

WSLLTFRSYWEEYVGAGLLLLFAAGLAGQWAFEQLVLFKSATQHRRRPNGTLPIFYNTFHAMKHGHRWAD
WITEQVEYFGGKSFWVRILGNPDELVLTTPQQFEDVLKTHFXXXXVKGPVTIDIFKGLLGAGIFAADGKHWAHQRKTAS
NLFSLRSLRESMTESIQKHALKLNDILDRTAKVSQPIDVFKLLNRFTMDTFAEIGFGIGLGNLEAVKDHPFTVAFDSAQHIL
VLRILRPSWFWKTQRALNLGIEREFKQHIRVIDDTVLAIIAQVLENRASQSQQLESPKKDIVSLFLDNVSADEKSQADAFD
PVFLRDIVVNFLIAGRDTTAQMLSWFFYLLSQHPHVEQKIRDELASKLPDLCQGKVSTISMEQTGELVYVEAALRETMRL
HPAVPRNSRYANRDVTLRDGTFIQKGQVVLISSYVLGRMPHVWGDDAKEYKPERWIDPQSGKLAQASPFKFPVFHAG
PRTCLGMNLAMVEMKIVAAVVLSKFHLELAPGQTITDGLSLTHPIKGALMMNVLPGGV*

>CYP5014S4 P.irregulare

WSLLEFHSYWQQAGVGLLLVLGAALVGQWTFERLTHPKNSQHRLRPKGTLPFVYNTFQAATLSHRWLDWL
VEQCELFEGKGFWTRIVGNPDTLTITSPELFEDVLKTNFEVFDKGPRVNDIFRDLLGAGIFGVDGVQWIHQRKTASNLFS
LRSLRESMTETIQKHALLLTGIFDRLATSAQPIDVFKLLNRFTMETFAEIGFGVELGLLGSTEEHPFQASFDGAQRILMLRF
MRPWWFWKTQRALGIGVEREMKQHIQAINDTVFGIIARALENRDSQPQSGKKDIVSLFLDNVSADSSNGLTQGGAFD
PVLLRDIVVNFLIAGRDTTAQALSWFFYMLSQNPAVEKKICDELARKVPALYHDKATAPSMEQTSDLVYLEGALRETLRL
HPSVPRNSKFANRDITLCDGTFVRKGESISISTYLLGRMTNVWGQDAGEFRPERWIDSESGKLIQVSAYKFPAFHAGPR
MCLGMNLAMVEMKIVASMVLSKFHLELVPGQEITYDFSLTHPIKGELMMNVRPVVK*

>CYP5014S5 P.iwayamai

MWPVADFARSLWQQAGVQHLLLLGAVAVAAHCAYEALVTLKDASRPRKLRPKWTLPLLYNSLHVALHKHHY
ADWVVAQLELFKQPFWLRMLGGPDILIVNTPEHFEDVLKTHFEAFAKGERMNGNLRDLLGAGIFGVDGHRWVHQRK
TASNLFSLRALRESMAESIQKHALVLNGIFERTAGGAEDQSIDLFRLLNRFTMETFAEIGFGVELGALTAEAEDHPFQAAF
DGAQRILMLRFVRPAFVWKTLRWLDVGAERELRDHMRMIDDMVLGIISQALTNRASQTHASYKSGKKDIVADAKEQ
NDAFDPVFLRDIVVNFLIAGRDTTAQALSWFFFLLSENPSVEAKIRQELAEKLPDLAANKAARVSMEQSGELVYLEAAM
RETLRLHPSVPLNSRQAQRDVVLSDGTLVKKGNAVSISSYVLARMTHVWGEDAKEFHPERWLDPQSGKLITVSAFKFTA
FHAGARTCLGMNLAMMEMKIVAAMVLSRFHLELVPGQGVTYDLSLTHPIKGELLMKCVPVKQE*

>CYP5014S6 P.irregulare

MWSLLNFTSYWQQVSAALLLLLAGALVGQWVLELLSSQKGTQPRLRPKGTLPIFYNTFQIIKHGHRWVDFIVE
QCEAVGGKPIWFKIIGGPEILTVTSPEQFEDVMKTHFEVFDKGWRMNDILQDLLGSGIFGVDGVPWVHQRKTASNLFS
LRSLRESMTDSVRKHALVLNGIFDRTSKSSQSIDLFKILNRFTIETFAEIGFGVELGSLDAPEEHPFQAAFDSAQRILMLRF

MRPRFVWKAQRALGVGPEKDLKTHIRAVDDTVLGIIGQALEKRASQPHVLSSKKDIVSLFLDNISVNGVGETSSEFDPVF
LRDIVVNFLIAGRDTTAQALSWFFYMLSQNPHVEKKLRDEIAAKLPELYAGKVSSPSMEETGELVYLEAALRETLRLHPSV
PLNVKQANRDVTLSDGTFIRKGQSVSISSYVLARMTHVWGEDAKEFVPERWIDSQTDKIIQVSTFKAPFFHAGPRMCL
GMNLAMMEMKIVAAMVLSKFHLELVPGQNVTYDLSLTHPIKGELMMNVVPVVKRI*

>CYP5014S7 P.irregulare
WILAAFVNFWQHATVNLLLLFVALAVTGQFAYDWLTLPKGAAPKLRPKGTLPVLYNTLHVALHKHHYGDWV
VEQLERIGQPFWVRIVGGPDILILNRPEQFEDVLKAHFEAFEKGDRMNVALKDLIGDGIFGVDGHKWVHQRKTSSNLFS
LRSLRESMAESIQKHALVLNEIFQRSATSSFSSSQQRSIDLFKLLNRFTMETFAEIGFGVELGSLDAVEDHPFQAAFDGAQ
RILMMRFVRPKFIWKALRYLNVGYERELTQHIRTIDDMVLGIISQALENRKTQTFAESASSTGKKDIVSLFLDNISVNGVG
ETSSEFDPVFLRDIVVNFLIAGRDTTAQALSWFFYMLSQNPHVEKKLRDEIAAKLPELYAGKVSSPSMEETGELVYLEAAL
RETLRLHPSVPLNSRQANRDVVLSDGTLIKKGNCCSVSSYVLARMTHVWGEDAKEFVPERWIDSQTDKIIQVSTFKAPF
FHAGPRMCLGMNLAMMEMKIVAAMVLSKFHLELVPGQNVTYDLSLTHPIKGELMMNVVPVVA*

>CYP5014S8 P.ultimum
MLEFAPVSLANAHHIYDWLVTQCERFDGKPYWLQLVGGPSVVVLSTPEHFEEVLKTHFETFDKGPIMNENLR
DLLGDGIFAVDSTKWVFDRAARSQQPLDLFKLLNRFTMETFAEIGFGVELGALASDHDHAFQAAFDSAQRVLGYRFMR
PQWTWKLQRYLNIGAEKGLKRSIQVINDTVFGIIEQSLENRRMNTSRSTLPQGKKDIVSLFLDHMSDDHASRSDPFDPT
FLRDIVVNFLIAGRDTTAQALSWFFYCLSQHPDVEHKIRAELAAKLPELMRSAIAHPSMEQVNQLSQNEGAVISSYVLAR
MTHVWGHDAKEYHAKSGRLVSESAFKFNTFHAGPRMCLGMNLAMMEMKIVVAMVVARFHLALVPGQHITYDFSLT
HPIKGALMMNVSSSSAV*

>CYP5014S9 P.irregulare
KHARLRLWPKWTLPVLLNTFQSAVHAHHIYDWIVTQCEYFQGQPFWLSLVGGPGVVVLSTPELFEEILKTHFE
TFDKGAIMTDNLRDLLGNGIFAVDGMKWVRQRKTASNLFSLRTLRESMAESVQKHALVLNEILQCTARSPDSTSLDLFN
LLNRFTIETFAEIGFGVQLGCLDSETDHPFQKAFDSAQRVLVYRFMRPAWFWKTQRYLSLGAEGGLKKSIQVIDDTVLGI
IAQSLENRKAPVDQRANQVGGGSGKKDIVSLFLDSVSSINETGKGETFDPVFLRDIVVNFLIAGRDTTAQALSWFFYCLS
QHPHAERKIRDELATKLPELFDGTIAAPSLEQTQQLVYLEAALRETLRLYPSVPANIKQANKDVMLSDGTFIRQGEGALIS
SYVLGRMTDVWGKDAKEFKPERWIDSSTGQLNIASAFKFNAFHAGPRMCLGMNLAMMEMKIVAAMVLAKFHLELV
PGQTVTYDFSLTHPIKGELRMK*

>CYP5014T1 P.ultimum
MRFHDWITEVCQAANGRPWLMTTVGRPSVIVISTPEAYEDVTKTQFDNFIKGEYVRDVLKDVFGLGIFSFDG
AQWAYHRKLTSNVFTKRSMRDSMTATIQKHCHSLCSAFQRAADEQRELDLFRVLSQFTTEAFCEIAFGIDMDYLNSDR
EHPLQQALDGAQRAITFRLFSPTWLWKLQRRFGLGLEGHLQECLKTLDAIVLEIINKSITHHAQRDSADKPAKTDLISLFLS
HANDVDAEDKEIFTPDALKYMAMQFLLAGRETTAQTLSWFFWVLHQHPEDMSPSIAIVQQFTYLEAALKETLRLYPVA
PFNARDAAEDTTLSDGTFVPAGYRVGLPNYAIGRLTSIWGPDAEDFNPSRWIDPGTGKLAAIAVAQIFSRFHVDVLPGQ
NITYATSITLPMNAPLKVHIVAL*

>CYP5014T2 P.iwayamai
MTRMIVKHSQSLCRVLGVASETHKALDLSKVLTQFTTEAFCELAFGLEMDYLTSEREHPLQQALDGAQRAVTF
RLFAPVWLWKVQRALGLGLEGQLRKHLNTLDGIVLELITHSIEYHAALDKSASNTRTDLISLFLSQAAKDATVDGAEQLD
DMLTPESLKDMAMQFLLAGRETTAQTLSWFFLLLSQHPEVEAKLRDELSQRLTSTDDSEEISIADLQQCVYLEAALKETV

RLYPSAPITARDAAEDTTLSDGTFIPKGVRVGLPGYAMARLTSVWGDDAAQFNPGRWIDATTGKMVPVSSFKFVSFHA
GPRMCIGMNLAMLEMKIASAHILRRFRVQVVPGQTISYDMSITLPMNAPLMVQLAKI*

>CYP5014T3 P.irregulare

LSILRDVSASLAHAGVSTSAQVALSLGLLLLLGSLRSSLQSTTGSSTPHSRKLALTPASTLPILGNTVDLVKNGLRF
HDWVTEQCVAAQGRPWIMKALGRPDVIVVSTLEAYEDVTKTQFENFIKGDYVRDVLKDVFGVGIFTLDGAKWAHSRR
LSSQLFSMRQLRDSMTKSIEKHVQSLCSVFKDASAADTVASNKTLDLCKVLTQFTTEAFCEIAFGIEMDYLTSDKEHPLQV
ALDGATRALDIGLEGQLKQHVKALDAIVLDLIAQSIEHHAKHDDEGGNANPKSDLISLFLSQAAPKKDKDAAALAADGG
GDGKELDQPDEKVTPESLKYMAMQFLLAGRETTAQTLSWFFLLLSQHPEVETKIRNELKQKSLTASSDTDGDGSASTAQ
RLVYLEAALKETVRLYPSAPITARDADKDTTLSDGTFIPAGTRVGLPAYAMARLPSVWGKDAAEFKPERWIDQDTGKLV
HVSSFKFVPFLAGPRSCIGVNLAMLEMKIVLAHILSRFHVDVLPNQSVSYDMSITLPMNAPLLVRVRKA*

>CYP5014T-fragment1 P.irregulare

PTTTLLVAVLILAGFLSLTLLRKTAASREDKVPWLPGTLPIFYNTFQIFNHKDNLLDMIALGKMIGRPATIYIARPE
LIEDVLATQSENFDKGGYFKDNTKDLLGNGIFSVDGLKWAQQRKTANNLFSRRALRDSMAVTVNEQ*

>CYP5014U1 P.aphanidermatum

MHVASIINALLQHLQQWPWVSAVTLVAAVTIVRRWADNHRKRAAIAALGAVEATELPGKLPVIGHTHFFAK
NEHVLNDWIAAQCLAYAGKPWRLSVLGQPELLVLSTPQAMEDICKSQFDNFPKGEFTCDALHDLFGDGMIAVDGDK
WYRQRKIARHLVTMRALRDSMAHVVQKHVDTLLQILDEYAEDHRSVDLVKLLREFTIETFAEIAFGIHMGSLGSKSEHPF
HTAMDAISPRLLLRFRLPSWFWKTQRWLGVGAEGEIARNLTIVNKILLDVVAKSLDSRLDGEKATPSKRATDIISLFLDHS
SNGDGESKLSPQMLRDVVMVFMVAGRDTSADSLSWFVYMIQQHPHVEAKIRQELRDKLPTGVRRVELTMEHIAELVY
LEASIRETLRLYPPAAFNTREAKQDTVLSDGTFVRAGTRVGLSAYGLGRLPSVWGEDAAVFRPERWIDEATGGIISVSPFE
FMAFHAGPRTCMGASIAMLEMKLVAAKVLSTFHLDVLPNQDMTQEIGLTLAIKHGLQTQVRRV*

>CYP5014V1 P.vexan

MMRPSQHSLWLSGLADELPIGAGALLVALPAFIYTLFRSIYTHRTRILPACVDIDVVKTAARQPLPPSTLPVLGN
LVDIARQGERLHDWFAELSRHFLGQAFRLCIPGKPDLVVLSSPADFQIVQQTQADHFIKGANFHDLLADLLGDSLFIVNG
EQWKRQRRILARLFSSRALRCHMTPIIQAHTRSLLLQAAAHAAETGEELDVSALTHRFTLATFTEIGFGIDMASLAEGEAS
RSFEQAFDEAVHLATDRFRQPVSVWKALRWLNIGTERRLRDAIAVVNTTATDLITQSMARRRAMVVEDSTSATASDIT
SLLLADDSEEGLDAAIIRSLVVTALVAGRDTTADTLNWFLLALSGQPWVQTKLRDELLAAIPDLATSPTYVPSMDEIQHLP
YLEASIREVLRLYPAGPFTVKQCVQDTVLSDGSLIPSGADVGFAVYAMGRLESVWGRDACEFKPERFLDETTGELKPMS
NFQFSAFSAGERQCVGRRLAMLELKLALAALVARFRFSGSSPRTVQEQTYVPGVSLPMKTSLLMRVHRL*

>CYP5014W1 P.vexan

MLQLLVSTEATPSTVLVVAVAVAGVAALLVRAQPLFLDPRAQVSPVPSSFDTLHKDRASAPVVRPSTTLPVVG
DLLHLARHANRGHDWFLELQRESGGKPVLLKLPGKPDLLLIATPELFEAMQKDQLESFAKGENFHDLLVDLLGDSIFIVN
GVKWKRQRQFLARLFTARTLRYHVAPIVQKHTHTLLDLLGSAAEARQVVDVTEIMHRFALEAFVELAMGVELGQLSAR
AQHPFQAAFDTAQLVIARRFSVPTFVWKLKRWLNVGSERRLRESLEVVNTMALDLINQSMEQRKHQSDDRAKHLVD
QILEMAETDQLEVHPTEVRDIMVATMVAGRDTTADTMSWFFHVLSHEPQAVAAIRAELLENVPQLATDPTFVPAMD
DIQHLPILEATIREVMRLFPAGAFTVKHCIQDAWLPDGTFVRAGTDVGVAVYALHRLESVWGDDAAQFKPERFLDDST
GKLRSFSPFQLATFSAGPRSCVGKALAMLQLKMVLATVLFHFDLVEEPGQEVVYTAGITLTMKNPLLMRPRRTVAIGA*

>CYP5014X1 P.vexan

MPRGPPPLHLGFPVAAGLVGGRVSTYSQPAKVQDPMSGLHCKASCNCRVEGDRILRSTEGPTDRAHFSFSAF
ELKPTAPATAMLEFLANLRGRVSVEVQLASAVAVSLAVLYTVSSSRRGQKQLNVAEPPTRKPLIGDTLELLRNNDNFHD
WLLDLCLRFDGRPFSLSAPGRPQVLIINSPEAFEDVVKTQFDNFGKGPYLYDIMHDLVGDSIAIRKIFASLFSARALRESM
ATVVRKHAQSLNKVFERSAETGDAIDLFKLMNRFTMVTFAEIGCSIEIDSLVTGNEHPFERAFEEAEHIIGSRMSLPVWF
WKLQRFLNVGAEKELKHHVDVIDKIVYGFVLESINARARGEKVGGRDIISLVLDNSEVNEGVTPSLLRDIVMSALVAGRD
TTAETLSWFFHMIAGHPEVERKVRAEVRTVLPKLFEDPYYIPTMEDVQNLIYMEAAFKETLRLYPPAPFNYKHAFNDTFL
SDGTFVPGGTDIGIPNYAMGRLTQIWGPDAREYRPERFLDDEGKLLTISNFKFSAFHAGPRLCVPGQDITYCRSVTMPM
KNPMMVNVHPVASPNAA*

>CYP5014X2 P.vexan

MLGFLFNVRDRVSVEVQLASAVALGLTALYTVSSLRPSRKKQLNVAQPPTRKPLXXXXNDNFHDWLLELCLEF
DGRPFSLQAPGRPPVLVINSPEALEDVAKTQYDNFGKGEYLYDIMHDLVGESLAVLDGERWRFQRKIFASLFSARTLRES
MTVVVQKHAQTLNEIFARSAQSKEPLDLFKLMNRFTMVTFAEIGCGIQMDTLVTGKEHPFEHAXXXXWKLQRFLNVG
SEKLLKEHVAVIDEIVYGFVLESINARARGEKVGGRDIISLVLDNSEVNESVTPSLLRDIVVSALVAGRDTTGETLSWFFHEL
TSQPGVEAKLRAEIRAKIPKLFEDTNYIPTMEDVADLTPRSTRRCD*

>CYP5014Y1 P.vexan

MLQFLPKLQNLFVGPDGDAPTSLQLASGVLLVAATAAVLARSLRSKETPGKPLRQLKSTLPLLGNTLDLALNND
TFHDWIAGLVEEFDGEPFVVKAPGRPDIVFITTPEMFEDVSKTQYDNFGKGDYQFELFHDLLGTALTIVDGERWRFQRK
IFANLFSARQLREVMQPIIRKHTQTLHSIFTDAVSTGEPLDLFRLFNRFTMETFAEIGYGIQMGSLGKKEAHPFETAXXXIA
MGRFSLPVWFWKLQRLLGVGKEGELVRAVEIIKETGLKFINESIEKRNRGEKSEAKNIVSLVLDSVDSDIVDPELLRSIVVA
SVAAGRDTSSNTLSWFVHVLTQHPDVEDKIRAEMFAKIPKLSTDIGYLPTMDDVQELTCAFQPPTLRQRHVPVGRHVX
XXIPADTDIGIPYWAMGRSAKIWGDDCTEYKPERFIDPKTGKLVQLSPFKFNVFHAGPRSCLGMNLALMEMKIVIAGLL
SRYHVVALPGQTITYARSLTLPMRNPFMVRVQPVGGR*

>CYP5014Y2 P.vexan

MLAFLQNLQNAVASGDDASPSGLELASGVLVALVAAAMLARGLRGKKTPGKPLLQPKSTLPLLGNTLDFVKN
NDIFHDWIASLVEEFENRPFVLTAPGRPDMVVISTPETFEDVSKTQYDNFGKGDYQFELFHDLLGTALTIVDGERWRFQ
RKIFANLFSARQLREVMQPIIRKHTQTLHSIFTDAVSTGEPLDLFRLFNRFTMETFAEIGFGIQMGSLAKKEAHPFETAFD
DAQELCTRRFSLPAWFWKLQRLLRVGGEAELIRAIEVIKTTALGFINESIEKRNRGEKSEAKNIVSLVLDSVDSDVVDPELL
RSIVIASLAAGRDTSSQTLSWFVHVLTQHPDVEDKIRAEMFAKIPKLSTDIGYLPTMDDVQELTYLEASIKEVMRFYPAVS
FNLRHSSNDTYLSDGTFIPAGTDLGIPCLGMNLALMEMKIVVACLFSRHRVVPLPGQTVTYTRSITLPMRDPFMVHVES
VKEAAAPTNS*

>CYP5014Z1 P.vexan

TKTLEEEALEAAKAAYASLPVPPTTLPVLGNVLDLLRNNDVLHDWVLANCERVHPSPFVIRAPGRPDMLVVST
PEAFEDVVKIQFDVFGKGPGIHMRISDLLGDSLTLVDGDKWRVQRKILASLFSARTLREAITRIIQKNARVLLSILEQQRDG
NTPFDLCNLLGRFTIEVFAEIGFGLELGGLAAGEASQFEQAFDRAQYIVTTRFTQPTALWKLKRLASVGLEGELKRCVQVI
DATVLTLIDRSLNRTEGEKQRASDEGRHDIVSMAIDAMTADHEAFDAKLLRALSVSALVAGRDTTSQTFCWFIFMLTQH
PEVELKLRDELLAKLPKLRESTDWVPAMEDVHDLPFLEACLKETLRLYPAAALNSRVANRDTVLSDGTFVPEGATVLFPS
YTMGRLEALWGHDAREFKPERWLDPDKPGKLVQVSPFKFNAFLAGPRMCVGMNLAMLELKVLAATTLGRFHFAAEP
HQHVTYQRSISLPMRDPFMVRAVGVPHTAPIA*

>CYP5015A1 P. sojae

MGGSSASSSSSLALWVALPTAAAAAVLAYLLIPDERQRAIRRLPAPASTLPVLGNTLDMMSLEQPRLHDWIAE
QCKAFGGRTWRLQVVGAPPLVVVSSVEGFEDVLKTQFEVFDKGDRMNTIFRDIAGGGIVAVDGPQWVAQRKMLSRL
FTMRAFRDTISQCVHDYTLVLGRMLGDAARTGVPIDFADVMHRFSFDVFTDIAFGLQGNSLEGGEHTQFMEAMGKIV
HNIEMRFHSPDWLWKLKRALKLGSEKELAQEVAILDKMVFTIINKNMERKFNPDAAAAEWPPRPQRSTKDVVSLFLDA
HDEQKAAGEDGGDTPLDANFLRDIAVVVLLAGKDTTAWSMSWLIIMLNRNPKVETKLRQELREKLPKLFSDPSYVPTM
DDVEGLVYLEAVLRENLRLNPLVPLNAKEANRDTTLVDGTFVKKGTRVYIPSYTLGRMKSVWGRDASKFKPERWLMQD
PWTGEQTIRPVSAFQFVSFHAGPRTCLGMRFAMLEMKTVLAYMLSKYHFTTRENPKSYTYDVASLLQVKGPLICKVQR
AG*

>CYP5015A1 P. ramorum

MGESKSSLWVALPAAAAAAVLVYLLVPDERQRAIRRLPAPASTLPLLGNTLDLMSLELPRLHDWLAEQCKAFG
GRTWRLQVLGAPPLVVVSSVECFEDVLKTQFDVFDKGARMNEIFRDVAGGGIVAVDGPQWLAQRKMLSRLFTMRAF
RETISQCVHDYTLVLGRKLNDAVRTGAPLDFADLMHRFSFDVFTDISFGLQANALEGGEHSQFMEAMGKIVHHIEMRF
HSPDWLWKLKRALKLGGEKELAQEIAILDKMVFTIINKNMERKFNPDAAANEWPPRPPRSTKDVVSLFLDAHDEQKEA
GGSPLDASFLRDIAVVVLLAGKDTTAWSLSWLIIMLNRNPKVETKLRQELREKLPRLFSDPTYVPTMDDVENLVYLDAVL
RENLRLNPLVPLNAKEANRDTTLVDGTFVKKGTRVYIPSYTLGRMKSVWGRDASKFKPERWLMEDPWTGELTIRPVSA
FQFVSFHAGPRTCLGMRFAMLEMKTVTAYMLSKYHFTTKENPKSYTYDVASLLQVKGPLVVKVQRAG*

>CYP5015A2 Phytophthora capsici

MVASSSSSSSSSLWVVLPTAAAAAALIYLLVPDEKQRAISRLPAPTSTLPVLGNTLDLMTLEQPRLHDWIAEQC
KAFGGRTWRLQVVGAPPLVVVSSVECFEDILKTQFEVFDKGDRMNTIFRDVAGGGIVAVDGPKWVAQRKMLSRLFT
MRAFRETITECVHDYTLVLGRFLNDSARTGAPIDFSDVMHRFSFDVFTDISFGLQANSLEGGEHTEFMEAMGRIVHNIE
MRFHSPDWLWKLKRALKFGSEKELAEEIAILDKMVFTIINKNMERKFNPDAAAQRSPRTTKDVVSLFLDAHDEQKASDE
AGDSPLDASFLRDIAVVVLLAGMDTTAWSLSWLIVMLNRNPKVETKLRQELREKLPRLFSDPNYVPTMDDVENLVYLD
AVLRENLRLNPVVPLNAKEANRDTTLVDGTFVKKGTRVYIPSYTLGRMKSVWGRDASKFKPERWLMQDPWTGELTIR
PVSAFQFVSFHAGPRTCLGMRFAMLEMKTVTAYMLSKYHFTTKENPKSYTYDVASVLAIKGPLIVKVQRAG*

>CYP5015A3 P.infestans

MGKSSSLWVVLPTAAAAAVLIYLLVPDERQRVIRRLPAPASTLPVLGNTLDLMSLELPRLHDWIAEQCKAFGG
RTWRLQVLGSPPLVVVSSVECFEDVLKTQFEVFDKGHRMNDIFRDVAGGGIVAVDGAKWVTQRKMLSRLFTMRAFR
ETISQCVHDYTLVLGRSLNESARTGVPIDFADVMHRFAFDVFTDISFGLQANSLQGGEHTEFMEAMGKIVHNLEMRFH
SPDWLWKLKRALKFGSEKELAEEVAVLDKMVFTMINRNMERKFNPDAAANEWPPRPPRTTKDVVSLFLDAHDEQKTS
AEADDTQLDANFLRDIAVVVLLAGMDTTAWSLSWLIVMLNRNPKVGTKLRQELRDKLPRLFSDPSYVPTMDDVEGLV
YLDAVLRENLRLNPVVPLNAKEANRDATLVDGTFVKKGTRVYIPSYALGRMKSVWGRDAAKFKPERWLMQDPWTGE
LTIRPVSAFQFVSFHAGPRTCLGMRFAMLEMKTVTAYMLSKYHFTTKENPKSYTYDVASVLQIKGPLIVKVQRAG*

>CYP5015A4 P.parasitica

MGGSSTSSSLWVALPTAAAAAVLIYLLVPDERQRAIRRLPAPTSTLPVLGNTLDVMGVELPRLHDWIAEQCKA
FGGRTWRLQVLGAPPLVVVSSVECFEDVLKTQFEVFDKGQRMTNIFRDVTGGGIFAVDGAKWVTQRKMLSRLFTMR
AFRETISQCVHDYTLVLGRSLNESARTGVPIDFSVVMHRFAFDVFTDISFGLQANSLEGGEHTQFMEAMSKIGHNLEMR
FHSPDWLWKLKRALKLGSEKEMAEEIAILDKMVFTMINKNMERKFNPDAANEWPRRPQRTTKDVVSLFLDAQDEQK
TSSEAYDTQLDANFLRDIAVSVLLAGMDTTAWSLSWLIVMLNRNPKVETKLRQELRDKLPRLFSDPNYVPTMDDVEGL

VYLDAVLRENLRLNPVVPLNAKEANRDTTLVDGTFVKKGTRVYIPSYSLGRMKSVWGRDASKFKPERWLMQDPWTGE
LTIRPVSAYQFVSFHAGPRTCLGMRFAMLEMKTVTAYMLSKYHFTTKENPKSYTYDMASILQIKGPLIVKVQRAG*

>CYP5015A5 P.iwayamai

MDKLKLDGVGASSWAVASAVVVGGALAWAVLPDAKQHALRHLPAPASTLPVLGNSLDLALYQTHRAHDWI
AEQCALHGWKPWRLQILGSAPMVVLSSHEHFEDVLKTQFEVWDKGPEMKFVFNDVFGDSIIAVDGEQWKFQRKKLS
HLFTMRSYRETITTSIHKYIRVLGTVLDDAMLHPATPFNLGEACHQMSFDIFAEVGFGLQTNALATRDNNPFVVSLGTLS
ETLESRFQQPNFIWRFKRFFNIGSERRMKECLAVMHGAMNDIILRNLTRRNDPTAATRPTKDVISLFMDEMDLDNATE
DDFKLLRDIAFSIIGAGKDSTATTLMWTIIMLNRSPDVEHKLRDELREALPKLFSDKSYVPSMDDVDQLPFVEAVVKETLR
LCPIVPLNAKEANRDTTLSDGTFIKKGTRAYVPTYTLGRNPNVWGQDATKFKPERWIEVDELTGKQKLIVVPATKFASFH
AGPRICPGMRVGMFELKAALAYIVSKYHLQTLRRAEDFTYAVSAVLSVKGPLMVSVEHARARSY*

>CYP5015A6 Phytophthora capsici

MTLELPRLHDWLAEQCKAFGGRTWRLQVVGAPPIVVVSSVECFEDILKTQFEIFDKGERMNTIFRDVAGEGIG
AVDGPKWAAQRKMLSRLFTMRAFRETIIINKNMEQKFNPDAAAQRAPRTTKDVVSLFLDAHDEQKSPLGASFLRDIAV
VVLLAGMDTTAWSLSWLIVMLNQNPKVETKLRQELRKKLPRLFSDPNYVPTMDDVENLVYLDAVLRENLRLNPIVPLN
AKEANRDTTLVDGTFVKKGTRVYIPSYTLGRMKSVWGQDAPKCKPERWLMQDPWTGELTIRPVSAFQFVSFHAGPRT
CLGMRFAMLEMKTVTAYMLSKYHFTTKENPKSYTYDVASVLAIKGPLIVKVQRAG*

>CYP5015A7 P.vexan

MMSLSSSNASSHHSMPGADQLDGTEKRQNVLVYYVGGVTVAELTAYRWLNQKQTAFSFFVAATSLPRLHD
WIAEQCEMFGGRTWRLQALGAPPLVVVSSPALFEDVLKTQFEVFDKGERMRTIFNDMAGESIVAAD*GDKWYQQRK
IMSRLFTMRAFRDTISARFHDHTLVLGRVXXXXXXXXXXXXXXXXXXXXXXXXXXXXXXXXXXXXXXXXSLERGGRNDYFDAI
VSTNRALEARFHQPDWLWKLKRLLRVGQERTLANNIALINKISYGIIEDTLRRRSGAASDATAGEAEQRSTKDVISLFVDH
QQDFHHASDGEDHFELTPKALRDVTTTFLAAGKDTTALTMTWLVVMLNRNPAVELAIRQELREKLPELFKDHEFVPTM
HDIDQLVYLEAAIRESLRLNPVVPLNAKEANRDVTMCDGTFLRKGTRVYIPSYALGRMKNVWGDDAAEYKPERWIELD
PTSGKPQLRHFSAFQFPAFHAGPRICLGMRFALTEIKIALAFLLSKFHFETERDPSSFSYDIALTLAVKEPLMVTVSSV*

>CYP5015A8 P.aphanidermatum

LDDAVTPALAVGGAALLWLLLRPDPRLRSVQALPCPASTLPVLGNTLDLASYHLEHLHDWILEQCLLHEGRPW
RLQALGSPPLIVVSTPDLFEDVLKTQFEIFDKGPRMNMIFESAMGDGIFAVDGDKWKQQRKALSHLFTMRKFRDTIAH
AIEKHLQVFAQVLDKAVTDQDAGRTALFEMSSAFHRYAFDTFTEIAFGLDEDTLGRMGTDGEDALFLTACTDVTKHLE
MRFHQPDFVWQLKGKLNVGTERLLKEAIDIVDKKVYAVVHKTIERSQKGEASQGQDVLSMMMEHMHEFKDEHATD
PQRLGKFLRDVGFSMIAAGKDSTGTTLTWVLIMIARMPHVLTEIRQELRSVLPALFTDRSFVPSMDEVENGLPYLEAVV
RETLRLCPLAPLNAKEANHDTTLCDGTFVPRGSRVYIPAYALARMPSVWGADAADFKPQRWLDGGKLRSFSSFQFNAF
HAGPRICLGMRFALVQLKTTLAYLLSKYDFELEKDPHAYKYEMAITFALHGDLRARVTRL*

>CYP5015A-fragment1 P.ultimum

MDKVELPWLLLMSIVAGRAIALILDAKNRTIRKLPKFASTLPIVGNLLDLIKYQNHRVHDWITEQCRAWRLLIM
GLTQKINRKFLCGVNFTILGHGIDSTASMLTWYVIILRHHAEVEKDYDYVPSLEDLGARVLGRALSESLRLNPTVPLNAKE
ANCNTTLPNSSFLKKSTRKCISLCAHDARVGTRCAPSARLKLTTQVENVAYTATNFPSFHTGPCICLVIAEMHSNYSGWP
PQADNVPAQVNAQRTSPKTPRDDQIDKVNEATNYLKSIMSTTVAIHLTSRSFAMAFWKLLRKS*

>CYP5015B1 P. sojae

MSNMVLPFAIAASLVAAAVAYFTSPTEQDRAVCELPTPRSTLPVLKNTLDLTIRQRARIYDWILEQCREHGGRP
WRVRVLGRPPAVILSSPEAMEGVLKTQFDVFVKGSAVAEISHDLLGEGIFTVDGSKWRHQRKAASHFFSMNMIKHAM
EHVVRDHSALLAVKLRAAADNGETLNIKRVFDFFTMDIFTKIGFGVELKGLETGGNCDFMEAFERASRRIMARFQQPM
CVWKLARWLNVGAERQMAEDMKLINGVVYDVIHRSLEGNDKRSSCSGRKDLVSLFLEKASVEYAADDHTEMTPTML
RDMSMVFIFAGRDSTSLTMTWFIIEMNRHPEVLANVRRELADKLPKLGMDDTETPSVEDIDQLVYLEAAIRECIRLNPV
APAMQRTAAQDTTLYNGTVIKAGTRVILPHYAMGHLETVWGPDAEEFKPERWIDADTGKLLHVSPFRFTAFLAGPRM
CLGMRFALAEMKITLATILSKFDLQTVENPDGFTYIPSVTLQVKGPVDVAITRAHA*

>CYP5015B1 P. ramorum

MLPPFAIAASLVAAAVAYLTSPNDQDRAVCELPTPRSTLPVIKNTLDLAVRQRARIYDWILEQCREHGGRPWR
VRVLGRPPAVIVSSPEAMEDILKTQFEVFVKGSAVATISKDLLGDGIFAVDGSQWKHQRKAASHFFSMNMIRDAMEHV
VRDHSVRLTKKLSEAAVSGEVVNIKRVLDFYTMDVFTKIGFGVELRGLETGGNSDFMEAFERATRRIMARFQQPMCIW
KLARWLSVGAERQMASDMKLINGVVYDVIHRNLEGKKQRKQSGGTFNSGREDLISLFLEKANVEYSDDDHVKMTPTM
LRDMSMVFLFAGRDSTSLTMTWYIIEMNRHPEALANVRRELTDKLPRLGLNDAETPSMEDIDELVYLEAAIRECIRLNPV
APVLQRTAAQDTTLYDGTFVKAGTRVILPHYAMGRLETVWGPDAEQYKPERWIDPDTGKLVHASPYKFAAFLAGPRM
CLGMRFALAEMKLTLATVLSKFHIETVEDPFEFTYVPSVTLQVKGPVDVRVTRPSSM*

>CYP5015B2 P.parasitica

VLPFAIAASFIAAAVAYFTSPNDQDRAVCELPTPRSTLPLVKNTLDLVFIQRARIYDWILEQCREHRGQPWRVR
VLGRPPAVILSSPEAMEDVLKTQFDVFIKGPTISDISHGLLGDGIFVVDGIKWKHQRKTASNFFSMNMIRDAMENVVRD
HSKLLTSTLNEAVENGETLNIKRVLDLFTMDIFTKIGFGVELHGLETGGNSGFMEAFERASSRIMARFQQPMCMWKLA
RWLNVGAERQMAKDVKLINDVVYDVIHRNLEEKASRKRSGENSNSYRKDLISLFLEKASVNYSDDDQTEITPTMLRDM
SMVFIFAGRDSTSLTMTWFIIEMNRLPEVLAKVRRELADKLPKLGRDGDTPSLDDIDDLVYLEASIREAIRLNPVAPVMQ
RIAAQDTTLYNGTFIKAGTRIILPHYAMGRLETVWGSDAEEFKPERWIDQDTGKLIHVSPYKFTAFLAGPRMCLGMRFA
LAEMKITLATIFSKFDIQTVNNPLDFTYIPSVTLQVKGPVNVTLSKTTRAT*

>CYP5015B3 P.vexan

MPVWIPVAIGGSCVAVFVCYLATPNEQDRAVLHLPTPRSSLPLLHNTLDLMVKQRTRIYDWLFEQCREHQGG
PWRVRVLGRPPAIVVSCPEAFKDVLKTQFDLFVKGPMITAIVSDVLGHGIFGVDGELWSHQRKTSSHLFTAHMIRDAM
QDVVRDHSMVLGIKLRGAAANGDTVDVKRLLDFFTMDVFVKIGFGVDLGGLESDGESEFMDAFERASRRVVQRFQQ
PMVVWKLLRWLNVGAERQHAKDMELLNEFVYTVIARSVQEKTKATADVSTCPVYSKKNLISLFLDKADVVYSDGHQA
PTDPRLIRDMTLNFLFAGRDTTSLSMSWFIIMMNRHPAVLQRVRREIAEKLPDLMRDEVFAPTMDDVEHLVYLEAAIR
ESMRLNPVVAVTTRIATQDTTLFDGTFIRAGTRIILPHYAMARMQSVWGDDAEFYNPDRWIDPSTGKLIQVSPYKFSVF
LAGPRMCLGMKFALIEMKIALSMLLSKFELKTVRDPATISYRPSMTMQIDGPLDVVVSPYKFVL*

>CYP5015C1 P. sojae

MKLEAITALISPASVAASCVALLLVYVATPSAHDRAVKHLPTPEGDIPVLRSTLEIVRAQKSGKFHDWALAYCRK
FQGRPWCLRILGKTPSVVVCCPEAFEDIQKTQFDAFDKSPFVSAAMYDVLGHGIFAVSGPLWQHQRKTASHLFTTQML
QYAMEVVVPEKGEALVKRLDEISKANQVVNMKRLLDLYTMDVFAKVGFDVDLHGVESDQNAELLDAFDRMSVRMLE
RIQQPVWYWKLLRWLNVGPEKQLAEDIKMTDDLIYSVMSRSIEEKTKGSRKDLISLFIEKSAVEYTKGVHTKKDLKLMRD
FVISFLAAGRETTATTMSWVILMLNRYPKVLDQVRQELKAKLPGLASGETRAPTLENIQQLVYLEAVIKETLRLFPVVAIT

GRSATRDVRLYEGTVIKADTRVVMPHYAMGRMETVWGPDANEFKPERWIDPATGKVNVVSPFKFSVFLGGPRVCLG
MKFAMAEVKISLAKLLSQFDFKTVKDPFDFTYRSSITLQIKGPLDVVVSRLKA*

>CYP5015C1 P. ramorum
MKLEAMTAWVSPASVAATCVAALLVYLATPSAHDRAVKHLPGPDGDLPILRNTLEIIAAQKSGTFHDWALKY
CRKYQGKPWRLRVVGKEPTVVLCCPEAFEDIQKTQFEAFDKSRFVSAAMYDVLGQGIFAISGPLWQHQRKTASHLFTT
QMLQYAMEVVVPEKGDELIKRLDGVCLKEKTADRVVSMKRLLDLYTMDIFAKVGFDVDLHGVQSNQNAELLDSFDR
MSVRILERIQQPMWYWKLLRWLHVGPEKQLAEDVKKLDDLVYGVMARSIEDKNRQDASQSARKDLISLFIDKSDVEYT
KGVHTKKDLKLMRDFVISFLAAGRETTATAMSWVILMMNRYPDVLKRVRQELNEKLPGLASGKLRAPSMEDAQKLVF
LEAVVRETLRLFPVVAITGRSATRDVYLYEGTLIKAGTRVVMPHYAMGRMSTVWGPDVDEFKPDRWIDPITGKGKVVS
PFKFSVFLGGPRICLGKKFALAEIKISLAKLLSQFDFKTVRDPFDFTYGSSITLQIKGPLDVVVSRLH*

>CYP5015C3 H.arabidopsidis
MYASYVGEQYNWTTVSAIVCLALIFGYLAIPSAQTRAVLRLPRPSGCLPILGNTLLIARVQLSGRFHDWVSDICR
KNQGRPWCMHVLGKAPTVFVCTPEDFEDIQKTQYEAFGKNPLFVEAATDVLGQGVFAISGPLWYHQRKVASRLFSTQ
MIQYNMDVVVSEKCKQLMRSLDLASRRENSTNGVVSLKGMLDLFTMDVFCKVGFGIEMQGMERQPIIAMLEALQRS
SARIVGRILEPSWSWKLRRYLSFGAEKQFASDMKRVNDMLYKFIARSVKEKASRDAGGHEDSDSRMDLISLHLEQEAA
DNGKHGEILPKNLRDFLVSFLAAGQDTTATAMSWFVVMMNRYPKVLERVREEIRDKLPDLANGKQRVPSLHETHHLV
YLDAAVRETLRLFPVAPISGRTATRDVTMSNGLFLVKGTSVHMPHYAMGRMGSVWGPDADEFKPERWIDSSSGKVSA
VSSFKFSAFYGGPHACLGMKFAMSEIKITLAALLSRYDFETLRDPFDYTYRMALSLRIDGGLNVAVSQLTEQKQQEVSEIS
KSSAS*

>CYP5015C4 P.infestans
MKLEAMTTWVTPASVALSCVAVLLVYLATPSAHDRALKHLPTPEGDYPILRHTLEIIKAQRSGNFIDWALKYCR
KYEGKPWSLRVVGKYPAIVLCCPEAFEDIQKTQFDAFDKSPLVSEALYDVLGQGIFAVSGPLWQHQRKTASHLFTAQM
MQYAMEVVVPEKGEQLVKRLDGICLQEKESERVVNIKRLLDLYTMDIFAKVGFDVDLHGVGSNQNAELLDSFGRMSA
QLLKRIQQPMWTWKLLRWLNVGAEKQLAEDVKAVDDLVYGVISRSIEEKNRQGAKETARKDLITLFIEKSEVEYTKGVH
TKKDHKLMRDFVISFLAAGRETTATTMAWVILMLNRYSKVLKHVRQEIKDKLPDLASGKIHSPKLEDIQQLVYLEAVVRE
TLRLFPVVAVSGRSATRDVRLYEGTFIKAGTRIVLPHYCLGRMTTVWGPDADEFKPERWIDSVSGKIKVVSPFKFSVFFG
GPRICLGMKFALAEVKITLAKLLSQFDFKTVKDPFDFTYRPSITLQIKGPLDVVVSRLNV*

>CYP5015C5 P.parasitica
KVEAITWMSLTSIAVSSVTVLLVYLAIPSAHDRAVKHLPTPEGDYPILRHTLEIIKAQKSGKSLDWVLKYCHKYKG
KPWCLRSVGKYPSIVVCSSEAFEDIQKTQFDAFDKSPFVSEALYDVLGQGIFAISGPLWQHQRKTASHLFTAQMMQYA
MEVVVPDKGEELAKRLNGICLTEKESERVVNMKRLLDLYTMDIFAKVGFDVDLNGVESDENAELLDAFERMSLRMLERI
QQPMWVWKLLRWLNVGAEKQLAEDVKVVNDLVYGVISRSIEEKNRQGVKENSRKDLIALFIEKSEIEYTKGVHTTKDLK
LMRDFVISFLVAGRETTATTMSWVILLLNRYPEVLERIRQEIRDKLPNFASGKMRSPKMEDIQQLVYLEAVVRETLRLFPV
VAVSGRSATRDVRLYEGTLIKAGTRMIMPHYCMGRISTVWGPDVDEFKPERWIDPISGKIKMVSPFKFSVFLGGPRICL
GMKFALAEVKITLAKLLSQFNFKTLKDPFDFTYRSSLTLQIKGPLDVAVSRVNI*

>CYP5015C6 P.infestans
MKLEAMTTWVTPASVALSCVAVLLVYLATPSAHDRALKHLPTPEGDYPILRHTLEIIKAQRSGNFIDWALKYCR
KYEGKPWSLRVVGKYPAIVLCCPEAFEDIQKTQFDAFDKSPLVSEALYDVLGQGIFAVSGPLWQHQRKTASHLFTAQM

MQYAMEVVVPEKGEQLVKRLDGICLQEKESERVVNIKRLLDLYTMDIFAKVGFDVDLHGVGSNQNAELLDSFGRMSA
QLLKRIQQPMWTWKLLRWLNVGAEKQLAEDVKAVDDLVYGVISRSIEEKNRQGAKETARKDLITLFIEKSEVEYTKGVH
TKKDHKLMRDFVISFLAAGRETTATTMAWTNFQTSRVAKFTLQNWRTFSSSST*

>CYP5015D1 P. sojae

MWGPLELALNLSLTSWGVLVCSLLLGWHFLSSRKQARALSKFTRPASTLPVLGNTLDLMFKHRHDIHDWML
DECRRCEGRPWVLAAVGRPTTVVLSDVDAFEDVLHRKFDSFGKCSAWLVSDVFGDGIFAADGVSWIHQRKTASHLFSL
HMMRESMEQVVREQATVLCETLRAHCTDNQTSTSPQRGVPVNLKYTMDWYATNVFTRVGFGVDLDSLSSQEHNEF
FCAFTRLPIGIHRRIQQPGWLWRLKRALDLGDEKQLKLDMARVDGVIYQVISQSMESKSDTAPVESKRLPDLISLFLAKET
NEYRDREAKQDNGAVATCRVETTPKLIRDMAFNFTAAGRGTTSQSLQWFIIMMNRFPGVERKIREELQAKLPQLFEED
STPPSMNDVQQLVYLEAAIKESLRLNPVAPLIGRTATQDVVFSDGMFIPSGTRVIIPTFAVARLQSIWGEDAAEFKPERW
IDPHTGKLRVISLYKFLVFLAGPRSCLGAKLAMLELKVALATVLSKFHLRVLRDPFEIGYDASISLPVKGDVLAIVEAAKVGN
SAGAA*

>CYP5015D1 P. ramorum

MQACDYLQVDARYGCYHIVGRIARGGEVEGASSWLALSHLRASAQAPTMWSSLKFAIEASTTSWGALLLCSL
LVGWHLLSMRKQARAPSKFARPQSTLPVLGNTVDLMFTHQEDIHDWMLAECRRCKGRPWVLAALGRPMSLVLSNVE
AFEDVLQRKFDQFGKRSAWLVSDVFGDGIFAADGVSWIHQRKTASHLFSLHMMRESMEQVVREQAAVLCKTLFAHL
NSNQSSSMADQHGVPVNLKYTMDWYATNVFTRVGFGVDLDSLPSQEHDEFFRAFTRLPIAIHRRIQQPGWLWRIKRA
LNLGYEKQLKLDMKRVDDVIYQVISRSMTSKSSEDPRRLPDLISLFLAKESNEYRDRDTKQEGGAAATRSVKTTPKLIRD
MAFNFTAAGRGTTSQSLQWFIIMLNRYPSVERKIREELLAKLPQLFESNSSPPTMNDVQQLVYLEAAIKESLRLNPVAPLI
GRTATQDVCFSDGTFITSGTRVVIPTYAVARLKSIWGEDAAEFNPERWIDPQTGKLLVISPYKFLVFLAGPRSCLGAKLA
MLELKVALATVLSKFHLRVLRDPFEIGYDASISLPVKGDVLAVVEPAEMVVPPSAENVERSAGAA*

>CYP5015D2 P.aphanidermatum

MVVVGTVVLGYAFLRMMKKQPQGLPPQATTALPFLGSTVELFTKQWHRIHEWFLEESLRQGGKAWQLTAL
GRPPAIVLTQESHFEDVLKTQFDIFEKGPKTRSFIKDFFGENGIFALDGAAWRHQRKTASHLFSHHMMKEIMETVIIEDT
EIVLEKLQSATVQGSKVEVKLLMDALTTDVFTRIGFGVRLGCLRGDSSNSEFDGEFHRAFSRCPKVIQARAQQPVFLWKL
KRWLGVGAEKQLMIDMKRINGAVFEVIEQCMRCKHGETPKEQDLITLFIAKGGDEANVTPQLIRDMALNFIAAGRGTT
GQSLAWFLVMMSRHQDVYERVLEEMNEKMPGFVGSRQVPSMDKVASLTFLEAAIKESLRLNPAAPMNAREAMQDA
VLSDGTFIPKGTRVIIPTYATARFSWMWGDDACEYRPERWLDDDGKLKQISPFKFLVFHAGPRMCLGVKLAMLEMKIV
LAAIVANFKVELERDAHEITYDMSLSLPIKGELFARIYPVPTKHSEKLDGSAACVA*

>CYP5015D3 P.iwayamai

WSASQFLHAAFDSPLLLLACALVAGWYLLAPSKQLRALARLPRPASTLPFLGNTLDLMFKQRERFHDWTVDE
CVRHKGQPWLLTAVGRPPSIVLSNAAVFEDVLQKQFKVFEKGPAGRLIAKDMFGGGIFGVDGVQWLHQRKTASHLFS
LQMMRDIMETVIVEETTVLCDLLDDACRSPGKQLEIKELMDRLSTEIFVRIGFGIKLGCLQQQKQAGEFLEFFKAFSRCPV
AIHARIHQPMWLWRLKRLLNVGLEKQLKQDMKLVNDKIYELIEKSMASKVAHAPQQKDLISLFIANAGSEYKDADEKTV
RVETTPQLIRDVAFNFIGAGRGTTAHSFAWFVVMMNRYPDVKQRVRDELQATLPELCSGVKRAATMDDVQKLVYLEA
ALKESLRLNPPAPTNARTANQDTTLSDGTFLRAGTRVILPTYAASRMTWIWGDDACEFKPERWIDPATGKLLVVSPYKF
LVFHAGPRLCLGIKLAMMELKIVLATMMSRYDITTLKDPFEITYATSIALPIKGSVYVTVESAATTGPVATKATDVPLAGV
GAS*

>CYP5015D4 P.ultimum

MWGAASFSAKSLLDAFASSPSSLLVLLCAIAAGWYICAPSEQLRVLSALPRPKSTLPVLGNTLDLMFRQRDRFH
DWITEECKRQKGKPWLLTAIGRPPSIVVSTVDAFEDVLSKQFKTFEKGPLGRAISEDLFGKGIFSSDGVNWMHQRKTAS
HLFSLQMTRDIMERVVLEETANVCNILDAACESHEQVEFKDLVDYFSTDVFVRIGFGVDLGCLKNEQEPEFFRAFSRCSA
VIGHRIHQPMWLWQLKRFLDVGIEKQFKKDIKLVNDKIYEVIDKSMAINAAANKNRGDGATMPPTKDLISLFLEKECNE
YKDGDETVRVETTPQLIRDIALNFIGAGRGTTAYSFSWFIVMMNRYPDVMKKVRAELQTVLPELCQGTKKIPTVEDVKK
LVYLEAALKESLRLNPPGPANARTANQDTTLNDGTFIKAGTRVLLPTYALSRMSWVWGDDACEYKPERWIDPATGKLF
VVSPYKFMAFHAGPRLCLGIKLAMMELKIVLATMLSRYELKITQNPFEISYATSIVLPIKGHLYVNVAHANSAKTSASPGP
VDSAVGAGGA*

>CYP5015D5 P.vexan

MWDAAAFSTIVTAACSLAVLWWILVPDKRVRALSRMYRPVSTLPIIGNTLDVMFKHRDDFHDWLRDECERS
GGKPWLMTLVGRPPSIVLTSVAAFEDVMSKQFERFAKLSNSLASDVFGDGIFAVDGLKWVHQRKTSSHLFSHQMMRE
VMERVVLDKAALLCKLLENKHAENKAENLKDIMDWYATDVFVEIGFGVDLGCLKQQQKCEFFRSFTRLPMAIHARVQ
QPHWLWKLKRALNIAEERQLKQDLEVVNGAIFQMIADSMNLRARTTAGEIEQTASQRNLVSLFLEKDRAEYTNEDGEA
VHVPTTPKLIRDMAFNFTAAGRGTSAQSLQWFIIMLNRYPDVEQKIRAELQSKLPHICGSGATQVAPTMLEIQQLTYLE
AAIKESLRLNPAAPLIGRTAVQDTELSDGTFLRAGTRVVIPAYASARLESIWGDDAAEYKPERWIDSTTGKLLPVSPFKFLV
FYGGPRMCLGAKLAMLELKVALASILSKFHLHTTRDPFEIQYAPSLSLPIRGSVFVTVEPVDLPHSASVAA*

>CYP5015D6 Phytophthora capsici

LEFLFNVSAASWGVLLCSLLFGWYSLSCRKQKQALSKFPRPISTLPVLGNTLDLLFTHRHDIHDWILDECRRCEG
HPWLLTALGRPSSIVLSNVEAFEDVLHRQFDKFAKCSAWLVSDVFGDGIFAADGLSWTHQRKTASHLFSLHMMRESM
EQVVREQAQVLCQTLFDSVQDGSPVNLKYTMDWYATNVFTRVGFGVDLNSLTDQEHDEFFQAFTRLPIAVHRRVQQ
PGWLWRFKRAFNLGYEKQLKLDMERVDGVIYQVISQSMKAKTDTSSDDPKRLPDLISLFLAKETNEYRTEEGGRTVKTT
PKLIRDMAFNFTAAGRGTTSQSLQWFFIMMNRFPFVEEKIRQELWTKLPQLFDINSTPPTMNDVQQLVYLEAAIKESLR
LNPVAPLIGRTATQDVVFSDNTFITAGxVSCLGAKLAMLELKVALATVLSKFHLQVLQDPFEXGYDASISLPVKGDVLAM
VE

>CYP5015D7 P.infestans

MWNALEFALNVSLTSWGALLCSLLVGWYFVSSKKQARWLVKFPRPESTLPVLGNTLDLMFKHRHDIHDWIL
DECRRYEGRPWALTALGRPTSIVLSNVEAFEDVFHRKFDKFGKCSAWLVSDVFGDGIFAADGVSWIHQRKTASHLFSLH
MMRESMEQVVREQAAVLCETLEAHSTANKPANLKYTMDWYATNVFTRVGFGVDLQSLSSQEHDEFFRAFTRLPIAVH
RRIQQPGWLWRFKRAFNLGYEKQLRLDMKRVDGVIYQVISQSMESKTNVNNSQRLPDLISLFLAKETNEYRHQQDGST
THSVKTSPKLIRDMAFNFTAAGRGTTSQSLQWFFIMMSRYPHVEKKIREELLTKLPHLFDKSSKPPTMSGVQQLIYLEAA
IKESLRLNPVAPLIGRTATQDVHFSDGTFIPTGTRVVIPTFAVARLQSIWGEDAAEFKPERWLDPHSGKLLVVSPFKFLVFL
AGPRSCLGAKLAMLELKVALATVLSKFHLRVMRNPFEIGYDASISLPVKGDVLAVVEAVEVPGPCEPAQKGFAGAA*

>CYP5015D8 P.parasitica

FKHRHDIHDWILDECKRCDGRPWVLTALGRPTSIVFSNVEAFEDVLHRKFDKFGKCSAWLVSDVFGDGIFAA
DGVSWIHQRKTACHLFSLHMMRESMEQIVREQAAVLCETLEAHSTANNPANLKYTMDWYATNVFTRVGFGVDLQSL
SSQEHDEFFRAFTRLPIAVHRRIQQPGWLWRIKRAFNLGYEKQLKLDMKRVDSVIYQVISQSMESKTDVNTPQRLPDLI
SLFLAKETNEYLDQQDGSTTHRVKTSPKLIRDMAFNFTAAGRGTTSQSLQWFFIMMNRYPHVAAKIREELLSKLPRLFH

KDSAPPTMNDVQQLTYLEAAIKESLRLNPVAPLIGRTATQDVYFSDGTFVPSGTRVVIPTFAVARLQSIWGEDAAEFKPERWIDPQSGKLLVVSPFKFLVFLAGPSNGAE*

>CYP5015E1 P. ramorum

MKAVSELLGDRNDVAVAAAAAVAVSLGLSLLLHSSKKNATIEARKLPPMPKTTLPILKNILDAGGNAERFHDWLNEQSTEFDNRPWMFTIPGRPANIVLSSPEIFEDVLKTQDDVFLRGPTGQHISYDLFGNGMVITDGDLWFYHRKTASHLFSMQMMKDVMEATVGEKLGVFLDVLDIYHKRGKPFSIKEELSHFTMDAIAKIGFGIEMDTLKNSPDREEDHEFLKAFNEGSVAFGVRIQSPLWLWELKKYLNIGWEKILMDNTRIMHEFINDVIVQSMNKKAELAAKGEKLVARDLISLFMESKLRQTEEMHIEDDDATIMRDMVMTFVFAGKDSTAHSMGWFIVNMNRYPEVLQKIREEMKDKLPGLLTGEIKVPTEQQIRDLVYLEAVVKENVRLHPSTGFIVREAMESTTLVDGTFVEKGQTMMVSSYCNARNKRTWGEDALEFKPERMIDPETGKLRVLSPYVFSAFGSGQHVCIGQKFAQMEIKLAMATLFSKFDIKTVEDPWTLTYEFSLTIPVKGPLNVEVTPLAPLATAASA*

>CYP5015E10 Phytophthora capsici

MKSVSELLGDRNDVAVAAATAVAVSLGLSLLLHSTKKNKTIEARKVPPMPKTTLPILKSLLDIAGTVDRFHDWLYEQSVEFDHRPWMYHIPGRPETILLSSPETIEDAMSTQGHIFLRGPVGQYTSYDIFGKGMIIADGDQWFYHRKTASHLFSMQMMKDAMESTVQEKLEVFLDVLDVYHKRGKPFSIKEELLHFTMDVISKIGFGVELNTLKDSPYRETDHEFFETFDSAGVGFAVRVQTPIWVWRLKRFFNVGWEKVFQNDIKNMHDFINKVIMESMQKKAELAAKGETMEAKDLISLFMESNLRESEDMHIEDDDVTIMRDMVMTFIFAGKDTTGHSMSWFIVMMNRYPEILKKIRDEMKEKLPGLFTGEIRVPTQEQVRNLVYLEAVIKENMRLTPSTGFIARECMQDTTLVDGTFIRKGQTIMVSSYCNGRNKKSWGEDALEFKPERMINPETGKLRVFSPFKFSAFGSGQHVCIGQRFAMMQLKMTLATLFSKFDIKTVEDPWQLTYEFSIGIPVKGPMEVEVTPLGPV

>CYP5015E11 P.infestans

PKSTLPILKSLLDIGGAVDTFHDWLYEQSAEFDNRPWMYHIPGRPETIVLSSPDTIEDVMSTQGHIFLRGPVGQYTSYDIFGKGMIIADGDQWYYHRKTASHLFSMQMMKEAMESSVHEKLGVFLDVLDIYHKRGKPFSLKEELLHFTMDVISKIGFGVELNTLKDSPDRETDHEFLEAFDSAGVGFAVRVQTPIWVWRIKRFLNIGWERVFQNDIATMHNFINKVIMESMQKKAELAAKGEKMVAKDLISLFMESNLRESEDMHIADDDVTIMRDMVMTFIFAGKDTTAHSMSWFIVMMNRYPDVLRKIREEMKEKLPGLFNGEIRVPTQEQVRNLVYLEAVIKENMRLTPSTGFIARECMQDTTLVDGTFIKKGQTTMVSSYCNGRNTKSWGDDALEFKPERMINPETGKLRVFSPFKFSAFGSGQHVCIGQRFAMMQLKMTLATLFSKFDIKTVEDP

>CYP5015E12 P.parasitica

MKSVSELFGDRNDVAITAATAVAVSLGLSLLLHSTKKNKAIEARRVPPMPKSTLPILKSLLDIGGTVDTFHDWLYEQSAEFDNRPWMYHIPGRPETIVLSSPDTIEDAMSTQGHIFLRGPVGQYTSYDIFGKGMIIADGDQWYYHRKTASHLFSMQMMKEAMESTVHEKLEVFLDVLDIYHKRGKPFSLKEELLHFTMDVISKIGFGVELNTLKDSPDRDTDHEFLEAFDSAGVGFAVRVQTPIWVWRIKRFLNIGWEKVFQKDIAKMHNFINKVIMESMQKKAELAAKGEKMDAKDLISLFMESNLRESEDMHIEDDDVTIMRDMVMTFIFAGKDTTAHSMSWFVVMMNRYPDVLRKIREEMKEKLPGLFNGEIRVPTQEQVRNLVYLEAVIKENMRLTPSTGFIARECMKDTTLVDGTFIKKGQTIMVSSYCNGRNKKSWGEDALKFKPERMINPETGKLRVFSPFKFSAFGSGQHVCIGQRFAMMQLKMTLAALFSKFDIKTVEDPWDITYEFSIGIPVKGPLEVEVTPLASTSV*

>CYP5015E13 P.infestans

MKSVTDLLGDKNDLVVTAAAAVAVSLGLSLLLHAKKNKSTEAQKLPPVPKTTLPLLKNILDAGGNAERFHDWLNEQSTEFGNRPWMFTIPGRPDNIVLSSPEMFEDVLKTQDDVFLRGPSGQYISYDLFGNGMVITDGDLWFYHRKTASHLFSMQMMKDVMEATVREKLAVFLDVLNIYSKRNQQFSVKQELSHFTMDVIAKIAFAIELDTLKNSPDRDEDHEFLQAFNKACVAFGVRIQSPLWLWRLKRYLNVGWEKVLKENNAIIQNFINDVIVKSMNKKAELAAKGETMVARDLITLFMESNLR

QTEDIQIEDDDATIMRDMVMSFAFAGKDSTADNMCWFIVNMNRYPNELRKVRDEMKQKLPGLLTGEIVVPTQDQV
RDLVYLEAVIKENMRLHPSTAFIMREAMQDTTLVDGTFVRKGQTLMVSSYCNARNKRTWGEDCLKFKPERMIDPETG
KLRVLSPYVFSGFGSGQHVCIGQKFAMMEIKMTLATLLSKFDIETVEDPWKLTYEFSLTTPVKGGLNVKVTPLTPAASA*

>CYP5015E14 P.parasitica
MKSVSELFGDKNDVAVTAAAAVAVSLGLTLLLRSSKKDKSIEGRKLPPVPKTTLPILKNILDAGGNAERFHDWL
NEQSTEFNNRPWMFSIPGRPDNIVLSSPEMFEDVLKTQDDVFLRGPTGQYISYDLFGNGMVITDGDLWFYHRKTASHL
FSMQMMKDVMEATVREKLQVFLDVLNVYAKREQQFSVKQELSHFTMDVIAKIAFAIELDTLKNSPDRDEDHEFLQAF
NKACVAFGVRIQSPMWLWRLKRYLNVGWEKVFKENNTIIQNFINDVIVKSMNKKAELAAKGEKMVARDLITLFMESN
LRQTEDIHIEDDDATIMRDMVMSFAFAGKDSTADNMCWFIVNMNRYPEKLRKIREEMKQKLPGLLTGEIKVPTQDQI
RDLVYLEAVIKENMRLHPSTAFIVREAMQDAILVDGTFVRKGQTLMVSSYCNARNKQTWGEDCLEFKPERMIDPESGK
LRVLSPYVFSGFGSGQHVCIGQKFAMMEIKMTLATLFSKFDIKTVEDPWKLTYEFSLTTPVKGGLNVEVTPLTPLTPAAS
A*

>CYP5015E15 Phytophthora capsici
MKNISELVGDRNDVAITAATAVAKSKSNDARKLPPMPKTTLPILKNILDAGGNAERFHDWLNEQSTEFENRP
WMFAIPGRPDNIVLSSPEMFEDVLKTQDDVFLRGPTGQYISYDLFGNGMVITDGDLWFYHRKTASHLFSMQMMKDV
MEATVREKLEVFLDVLSIYSKRGQQFSAKQELSHFTMDVIAKIAFAIELDTLKNSPDREEDHEFLKAFNKACVAFGVRIQS
PMWLWRLKRYLNVGWERVFKENNDIIQNFINDVIVKSMNKKAEMAAKGEKMVARDLITLFMESNLRQTEDIHIDDD
DATIMRDMVMSFAFAGKDSTADNMCWFIVNMNRYPHELNKIREEMKEKLPGLLTGEIQVPTQEQLRDLVYLEAVIKE
NMRLHPSTAFIVREAMQDATLVDETFVRKGQTLMVSSYCNARNKRTWGDDCLEFKPERMIDPETGKLRVISPYVFSGF
GSGQHVCIGQKFAMMEIKMTLATLFSKFDIKTVEDPWKLTYEFSLTTPVKGGLDVEVAPLTPLTPAASA

>CYP5015E16 Phytophthora capsici
MRSVSELFGDRNDVVATAAAAVAVSLGLSLLLHSSKKSKSTDARKLPPMPSTTLPILKNILDVGGHAERFHDW
LNEQSIEFENRPWMFKIPGRPATIVLSSPEMFEDVLVTQDDVFLRGPVGQYISYDIFGNGMVISDGDPWYYHRKTASHL
FSMQMMRDVMEATVCEKLEVFLDVLSIYHQRGQAFSIKEELSHFTMDAIAKIGFGLDMDTLKNSPDREEDHEFLKAFN
EGSVAFGVRIQSPLWLWELKKYLNIGWEKILMDDTKIMHDFIARDLVTLFMEKKLKQTEDMHIEDDDATIMRDMVMT
FVFAGKDSTAHSMGWFIVNMNRYPEVLKKIREEMKEKLPGLLTGEIKVPTQEQINDLVYLEAVVKENIRLHPSTGFIVRE
AMQDTTLVDGTFVEKGQTVMVSSYCNARNEFYIAWNSSASSNKLREAQSVLEVCCSILDRQLVELLHVTP

>CYP5015E17 P.parasitica
KSVSELFGDKNGMAITAAVAVSLGLTLLLRNSKKSTSAEARKLPPMPTTTLPILKNILDVGGHAERFHDWLNEQ
SIEFDNRPWMFSIPGRPATIVISSPEMFEDVLATQDDVFLRGPVGQYISYDIFGNGMVISDGDPWYYHRKTASHLFSMQ
MMRDVMETTVCEKLSVFLDVLTIYSKRGQAFSIKEELSHFTMDAIAKIGFGIEMDTLKNSPNREEDHEFLKAFNEGSVAF
GVRIQSPLWLWELKKYLNIGWEKILMDNTKIMHDFIRKVILDSMNKKAELAAKGETMEARDLITLFMEKRLKQTEDMHI
EDDDATIMRDMVMTFVFAGKDSTAHSMGWFIVNMNRYPEVLRKIREEMKQKLPGLLTGEIKVPTQEQIRDLVFLEAV
VKENIRLHPSTGFIVREAMQDTTLVDGTFVQKGQTLMVSSYCNARNKKTWGEDTLEFKPERMIDPETGKLRVLSPYVFS
GFGSGQHVCIGQKFALMEIKLAMATLFSKFDIKTVEDPWKLTYEFSLTIPVKGPLDVEVTPMTPLSPAASS*

>CYP5015E18 P.infestans
MKAVSELFGIKDDVAATAAVAVAVSLGLTLLLRNTKKSKDTEARKLPPMPKTTLPILKNILDVGGHAERFHDWL
NEQSLEFDNRPWMFSIPGRPATIVLSSPEMFEDVLVTQDDVFLRGPVGQYISYDIFGNGMVISDGDPWYYHRKTASHL

FSMQMMRDVMETTVCEKLSVFLDVLSLYSKRGQVFSIKEELSHFTMDAIAKIGFGIEMDTLKNSPNRDEDHEFLQAFNE
GSVAFGVRIQSPLWLWELKKYLNIGWEKILMDNTKIMHDFISKVIVDSMNKKAELAAKGETMEARDLITLFMEKRLKQT
EDMHIKDDDATIMRDMVMTFVFAGKDSTAHSMGWFIVNMNRYPEVLRQIREEMKEKLPGLLTGEIKVPTQEQIRDLV
YLEAVVKENIRLHPSTGFIVREAMQDTTLVDGTFVQKGQTVMASSYCNARNKKTWGEDTLELKPERMIDSETGKLRVY
SPYVFSGFGSGQHVCIGQKFAMMEIKLAMATLFSKFDIKTVEDPWKLTYEFSLTIPVKGPLEVEVTPLTPLMPAASS*

>CYP5015E19 H.arabidopsidis

MGLGLTVFTHKNAKKPASSGPRKLPPMPKTTLPLLKNILDAGGNAERFHDWLNEQSIEFDNRPWMFKIPGRP
ATIVLSSPELFKDVLVTQDNVFLRGPVGQYISYDIFGNGMVIADGDPWYFHRKTASHLFSMQMMKDVMEATVFDKLS
VLLDVLDIYAKRGRPFSIKEELSHLTMDVIAKIGFGLELDTLKNSPDREEDHEFLKAFNEGSVAFGVRIQSPLWLWKLKKYL
NIGWEKILMDNTRIMHNFINKVIVDSMNKKARLAAEGETMVARDLITLFLEKRLVQTEDMHIEDDDATIMRDMVMTF
VFAGKDSTAHSMAWFIVNMNRYPQVLHKIREEMKDKLPGLLNGTIRVPTHQQVHNLVYLEAVVKENIRLHPSTGFIVR
EAMQDTTLVDGTFVQKGQTVMVSSYCNGRNPKTWGDDTLEFKPERMIDPDTGKLRALSPYVFSGFGSGQHVCIGQK
FAMMEIKLAMATLFSKFDVKTVTDPWQLTYEFSLTIPVKGPMDVEVTPLPSTVPAALA

>CYP5015E2 P. ramorum

MKAVSELLGDRNDVAVTAAAAVAVSLGLSLLLHSSKKNATIEARKLPPMPKTTLPILKNILDAGGNAERFHDW
LNEQSTEFDNRPWMFTIPGRPANIVLSSPEIFEDVLKTQDDVFLRGPTGQHISYDLFGNGMVITDGDLWFYHRKTASHL
FSMQMMKDVMEATVCEKLSVFLDVLGVYHTRGRTFSVKQELSHFTMDVIAKIAFGIELDTLKNSPDRDDDHEFLKAFN
KACVAFGVRIQSPMWLWELKRYLNVGWERVFKENNTIIQKFINDVIVQSMNKKAELAAKGEKLVARDLISLFMESKLR
QTEEMHIEDDDATIMRDMVMSFAFAGKDSTADNMCWFIVNMNRYPEVLQKIREEMKDKLPGLLTGEIKVPTQDQVR
DLVYLEAVMKENMRLHPSTGFIVREAMESTTLVDGTFVEKGQTLMISSYCNARNKRTWGEDALEFKPERMIDPETGKL
RVLSPYVFSGFGSGQHVCIGQKFAMMEIKLTLATLFSKFDIKTIEDPWTLTYEFSLTTPVKGGLSVEVTPLTPAASA*

>CYP5015E20 Phytophthora capsici

PMWLWXLKRYLNVGWERVFKENNDIIQNFINDVIVKSMNKKAEMAAKGEKMVARDLITLFMESNLRQTEDI
HIDDDDATIMRDMVMSFAFAGKDSTADNMCWFIVNMNRYPHELNKIREEMKEKLPGLLTGEIQVPTQEQLRDLVYLE
AVIKENMRLHPSTAFIVREAMQDATLVDGTFVRKGQTLMVSSYCNARNKRTWGDDCLEFKPERMIDPETGKLRVISPY
VFSGFGSGQHVCIGQKFAMMEIKMTLATLFSKFDIKTVEDPWKLTYEFSLTTPVKGGLDVEVTPLTPLTPA

>CYP5015E3 P. ramorum

MKTVSELFGDRNDLAVTAAAAVAVSLGLSLLVHKSKKNKSTEARKLPPMPKTTLPIFKSLFDIGNNLDRFHDWL
LERSAEFDNKPWMYSIPGRPVTIVLTAPEYLKDVMVTQEDVFLRGPLTQYMSEDIFGNGMIVTDGDPWFFHRKTSSHL
FSMQMMKDVMETTVRDKLDVFLDVLDIYHKRGKPFSIKKDISHFTTDAICRIGFGIELDTLRNGPDSDEDHTLLKAFDLA
SIAFVVRVQTPWWIWEPKRFFNVGWEKVFKDNVKIVHDFIDEVILQSMNRKAELAAKGEKMEARDLITMFMESNLKE
TEHMNFQDNQATIMRDMVTTFMFAGKDTVGHSLSWFIVHINRYPETLKKIREEMKEKLPGLLTGEIRVPTHEQLRELIY
LEAAVRENVRLFPSTGFIAREAMRDTTLVDGTFVGKGQTIMVSSYCNTRNKKLWGEDALEFNPDRMIDPETGKLRVFS
PYQYSGFGSGQHVCIGQKFAMMQMKLAMATLLSKFDIKTVEDPWKLTYDFSLTIPVHGPLDVVVTPLTPLSSA*

>CYP5015E4 P. sojae

MKSVSELFGDRSDVAVTAAAAVTVGLGLSLLLHSTKKSKMSDTRKLPPMPKTTLPILKNILDAGGNAERFHDW
LNEQSIEFDNRPWMLSIPGRPATIVLSSPEMFEDVLKTQDDVFLRGPSGQYISFDLFGNGMVITDGDLWFYHRKTASHL
FSMQMMKDVMEATVREKLAVFLDVLGVYHQRGQQFSAKQELSHFTMDVIAKIAFSIELNTLKDSPDREDDHEFLKAF

NKACVAFGVRIQSPMWLWRLKRYLNVGWEKVFKENNTIIQNFINDVIVQSMNKKAEYSAKGEKMVARDLITLFMESN
LRHSEDIHIADDDATIMRDMVMSFAFAGKDSTADNMCWFIVNMNRYPEVLKKIREEMKEKLPGLLTGEIRVPTQEQLR
DLVYLEAVMKENMRLHPSTAFIMREAMDNTTLVDGTFVEKGQTLMISSYCNARNKRTWGDDCLEFKPERMIDPETGK
LRVLSPYVFSGFGAGQHVCIGQKFAMMEIKTTLATLYSKFDIKTVEDPWEITYEFSLTMPVKGGLSVEVTPLTPLKRASSA
CK*

>CYP5015E6 P. sojae

MKIVTQLPTDKRDAAVAAAAVVTLGLLVSYLSRPKDKGNKPKRKMAHVPKSTLPLLGNMLDMSTNMPRFHD
WISECAEFDNEPWTLQIPGKEPWIVLSSAELFEDVLKTQADNFLRGPVSHHQAYDVFGNGLSISDGDAWFYQRKTASH
LFSMQIMKTVMEDSVREKLDVFLDVLGKYAARGKPFGIKKWLSHFTMDVFSKIGFGVELDTLKNTFDQEGDHEFLEAF
NVASVAFGVRIQTPTWLWELKKFLNVGWEKIIMDNCKKFHDFIDSFVLKAMVERGQNKVARDLISLFLDSSIDTSELQIE
EDEATIMRDMVTTFIFAGKDSSAHSLGWFIVNMNRYPEILRKIREEIKEKLPGLLTGEIQVPTAAQLQELVYLEAVIRENIR
LHPSTGFIMRQATEATTLVDGTFVDKEVSVLLPSYANARNPRTWGEDASEFKPERFIDADTGKIRNFSPFVFSSFGSGPHI
CLGMKLALMEVKLTLATLLSKFDFKTVEDPWQMTYDFSLTIPVKRPMEVEVTPLVTPYADSA*

>CYP5015E7 P. sojae

MKSVSELFGDRNDVAVTAAAAVAVSLGLSLLLHSTKKSKKPEGVRLPPMPKTTLPILKSLFDAGGNVARFHDW
LNEQSIEFDHRPWMYSIPGRPVTIVLTSPDTIEDALSTQNDVFLRGPVGQYMSEDIFGNGMIIADGDPWYYHRKTSSHL
FSMQMMKDVMEATVREKLEVFLDVLDIYHKRGQSFSAKQELLHFTMDVIAKIGFGLELDTLKDGPHRDEDHEFQEAFD
QAAVAYAVRVQSPLWLWEIKRYFNIGWEKVFRDNTTILHNFIDEVITQSMKKKAELAAKGEKMVARDLITLFMESTLRE
NQDMHIEDDDATIMRDMVMTMMFAGRDSTAHSMCWFIVHMNRYPEILEKIRDEMKEKLPGLLTGEIKVPTQEQLR
ELVYLEAVMKENIRLIPSTGFIAREAMRDTTLVDGTFVGKGQTIMVSSYCNARNADNWGEDASEFKPERMIDPKTGKL
RVLSPFVFSPFGSGQHACMGQKFAMMQMKLTLATLYSKYDIKTVEDPWKLTYEFSLTIPVKGPLDIEVTPLSPLMA*

>CYP5015E8 P.parasitica

KLVPADKRDAAVAAATVATLGLLLSYLTRPNDKKEKRKMAHVPKSTLPVLGNMLDMSSNIPRFHDWISEQCA
EFNNEPWTLKIPGKEPWIVVSSSELFEDVLKTQADNFLRGPVSQYQSFDVLGNGLSVSDGDAWFYQRKTASHLFSMQ
MMRTVMEDTVREKLQVFLNVLNTYATRGKPFGIKKELSHFTMDVFSKIGFGIELNTLKDTFDREEDHEFLEAFNVASVA
FGVRIQTPTWLWEIKKFLNVGWEKILMDNCKKFHDFIDSFILKAIEDRREKKVARDLISLFLESRIDTSELDIKEDEAQIMR
DMATTFIFAGKDSVAHSIGWFIVNMNRHPDVLRKIREEMKQKLPGLLTGEVQVPTSAQVQELVYLEAVLRENIRLYPST
GFIMRQATEATTLVDGTFVDKEVSILLPSYANARNPRTWGEDAHEFKPERFLDPDTGKLLTFSPFVFSSFGSGPHICLGM
KFALMEIKLTLATLFSKFDIKTVEDPWEMTYDFSLTIPVKGPMDVAVTPLSMPSADSA*

>CYP5015E9 P.infestans

MAHVPKSTLPLLGNMLDMTSNMPRFHDWISEECAAFNNEPWTLQIPGKEPWIVVSSSELFEEVLKTQADNF
LRGPVSQYQSFDVLGNGLSVSDGDAWFYQRKTASHLFSMQMMRTVMEDTVREKLEVFLGVLNQYAARGSPFGIKKE
LSHFTMDVFSKIGFGVELDTLKDTFDREEDHEFLEAFNVASVAFGVRIQTPTWLWEIKKFLNVGWEKILMDSCTKFHDFI
DSFILKAIEGRRENKVARDLISLLLESRIDTSELDIKEDEAQIMRDMAITFIFAGKDSVAHSIGWFIVNMNRYPDVLRSIREE
MKQKLPGLLTGEIQVPTSAQVQELVYLEAVVRENIRLYPSTGFIMRQATEATTLVDGTFVDKEVSILLPSYANARNPRTW
GKDAHAFKPERFLDPETGKLVTFSPFVFSSFGSGPHICLGMKFALMEIKLTLATLFSKFDIKTVEDPWTMTYDFSLTIPVKG
PMDVKVTPLYIATAESA*

>CYP5015F1 P. sojae

MNPAGLPHQQLQLQHSTSSCHSPIFSQQLKPSQPTNQLTGTSMWSSASHDGAQQSVLLAFGALTALYASW
KILSMPVPLPDPGMEDLFRPASTLPILGNTLDVLLFNRYRMSDWINDQTDASEGKPWILQLLFQPPWVVLSMPSDLDD
VFRDQFDVFEKGGTLGDISFDVLGNGLLNVSGDKWKQQRRAASHLFSTQSIRDVMEPVIREKTLQLRDVLAQCADREQ
TVSMKSLLGKFTSDVFTRIGFGVELNQLGGDVLVDDMHPLDIALHAVQNRFQTPMWMWKLTRFLNVGAERRLREN
MKIVNDMVRGIMVRSIGDKTPGDGKKNLLTLLMKDDVDADPRELQDTAVNFFIAGKDTTSFSLSWLIVMMNRYPRVL
QKIREEIASVLPGLLTGEMSAPTLEDTQKLVYLDAAVKESVRLWSVSTYRCTTRDTTLTSGAFIEKGTVVVVSKYAAARRK
NVWGDDAAEYRPERWFDEKTGEPKSITPPQFITFSTGPRKCIGMRLAMLEMKTVMAVLFSRFDIETVEDSFKITYDFSF
VLPVKGPLAVRIRDRTAPSV*

>CYP5015F1 P. ramorum

MWSSTSHDGAQQSVLMALGALTVVYASWKILNLPVPMPDPGMEQLFRPASTLPVLGNTLDVLLFNRYRMS
DWINDQTDASSGQPWILQLLFQPPWVVLSMPNDLHDVFVDQFQVFEKGGTLGDISFDVLGNGLLNVSGDKWKQQR
RAASHLFSTQSIRDVMEPVIREKTLQLRDVLAQSAGRKQTVSMKSLLGKFTSDVFTRIGFGVELDQLGGDVFKDEQHPL
DIALHAVQNRFQTPAWMWKLARFLNVGAEKRLRESMKTVNDMVRDIMVRSISEKSSGDQKKNLLTLLMKDNAAADP
RELQDTAVNFFIAGKDTTSFSLSWLIVMMNRYPRVLQKIREEIRSVLPTLLTGEMDAPTLEDTQKLVYLDAAVKESVRLQ
AVSTYRCTTRDTTLTDGAFIKKGTVVVVSKYAAARRKGVWGEDAAEYKPERWFDEKTGEPKNITPPQFITFSTGPRKCIG
MRLAMLEMKTVMAVLFSRFDIETVEDPFKITYDFSFVLPVKGPLAVRVRDRAPLSA*

>CYP5015F2 P. sojae

MIDSQALSPVLATAFALLLVCWKLLSKPRPHSNGQELFRPASTLPFLGNTLDVLWFQRHRLHDWMTEQSLAS
GGKPWLLTGIGQRPKVVLTSPAAYEDVFKTQFDVFVRGPGETVLEVLGQGIFNVDGDKWRHQRRVTSHLFSMHMLK
DCMKSVVREKTVQLREVLATCAERGQTVSMKSLLNKFTADTFTRIGFGVDLNGLADPVDVDTSQPLDTALGVVQTRLQ
SPVWLWKPRRFFNVGSERVMRENMQQVQDTVQKIMAKSLADKEHQANGEEATTSSKHKDLMSLMLQSGDFTDPR
EVRDICVNFYAAGKDTTAFSLSWFIVMMNRHPRVLCKVREELRRVAPELFTGELDTPTLGHLQQLTYLEAALKESLRLNS
LAVYRLANRDTTLSDGTFVPKDARAVFSMYASARQPSVWGSDAADYNPGRWIDEETGKLSSFKFVTFSAGPRQCIGM
RLAMMEMMTVLSVVFSRFDLETVVDPLDITYDFSLVLPVKGSLAVRVHSLSAHMA*

>CYP5015F2 P. ramorum

MIEQQALPSLLATVFALLLVWWKLLNKPSTNSQKLFRPASTLPILGNTLDLLWFQKHRLHDWMTEQSLASGG
KPWLLTGIGQLPRVVVTSPAAYEEVFKTQFDVFVRGPGETVLEVLGEGIFNVDGDKWRRQRRVTSHLFSMHMLKDCM
NAVVREKTVKLRDVLAMCAERGDPVSMKSLLNKFTADAFTRIGFGVELNGLDDPADVDTSQPLDAALQVVQIRLQSPV
WLWKLRRFFDVGSERVMRESMQQVHDTIQHIMAKSLADKEEQAAASEEATRTSSHKDLMTLMLQTGDFKDTREIRD
VAVNFYAAGKDTTAFSLSWFIVMMNRHSHVLCKVRDELRCVAPELFTGELDTPTLEHLQQLTYLEAALKESLRLNSLAVY
RLANRDTTLSDGTFVPKGARVVFSMYGSARQPGVWGPDAAEYKPERWIDETTGKLKNISSFQFVTFSAGPRQCIGMRL
AMMEMMTVLAVVYSRFDLKTVEDAFDITYDFSLVLPVKGPLAVRVHSLAAHKA*

>CYP5015F3 H.arabidopsidis

MWSSARHDGTQQSVLLAVGALTAVYASWKLLSLPVPVPDPGMETLFRPSSTLPILGNTLDVLLFNRYRMSD
WINDQTDASEGKPWLLQLLFQPPWVVLSMPNDLHDVFVDKFDVFEKGGALQDISFDVLGNGLLNVNGAKWKQQRR
AASHLFSTKSIRQVMEPVIREKTRQLRDVLAQCAKRGQTVSMKSLLGKFTSDVFTRIGFGVELNQLAGDVLLDEMHPLDI
ALRAVQNRFQTPMWMWKLQRFFNVGAEKQLRANMNIVNDMVRDIMVRSMREAQPTGEEKLNVLTLLMRDNADA
NPRELQDTAVNFFIAGKDTTSFSLSWLIVMMNRHPRVLQKIREEIRAVVPGLLTGETDVPTLEETQQLVYLDAAVKESVR

LQSVSTYRCTTRDTTLSDGAFITKGTVVVVSKYAAARRKIVWGDNAAEYRPERWFDETTGAPKNITPPQFITFSTGPRKCI
GMRLAMLEMKTVMAVLFSRFEIETVEDPFEITYDFSFVLPVKGPLAVRVRERTAYCV*

>CYP5015F4 P.parasitica

MPAPVPDPCMEDLFRPASTLPILGNTLDVLLFNRYRMSDWINDQTDASEGKPWILQLLFQPPWVVLSMPND
LHDVFVDKFDVFEKGGTLGDISFDVLGYGLLNVNGDKWKQQRRAASHLFSTKSIREVMEPVIREKTLQLRDVLAQCADR
EQTVSMKSLLGKFTSDVFTRIGFGVELNQLGGDVHIDEMHPLDIALHAVQNRFQTPMWMWKLARFFNIGAEKRLRD
NMKIVNDMVRDIMVRSITEKTLDERKNLLTLLMNDNVNADPRELQDTAVNFFIAGKDTTSFSLSWLIVMMNRHPRVL
GKIREEIRTVLPGLLTGEIEAPTLEDTQKLVYLDAAVKESVRLWSVSTYRCATRDTTLTDGAFIKKGTVVVVSKYAAARRTN
VWGEDAAEYRPERWFDEKTGELKNISPPKFITFSTGPRKCIGMRLAMLEMKTVVAVLFSRFDIETVEDPFKITYDFSFVLP
VKGPLAVRIRKRIDSTV*

>CYP5015F5 Phytophthora capsici

MWSISSHDGAQQSALLALGALTAFYATWKLLNMPIPRPDPDMKLFRPASTLPILGNTLDVLLFNRYRMSDWI
NDQTDESGGKPWVLQLLFQPPWVVLTMPNDLNDVFVDKFDVFEKGGALGDISFDVLGYGLLNVNGDKWKKQRRAA
SHLFSTSSIREVMEPVIRDKTLQLRDVLAKAADEDQTVSMKSLLGKFTSDVFTRIGFGVELNQLGGDVLVDEMHPLDIAL
HAVQNRFQTPMWMWKLTRFLNIGAEKRLRDNMKIVNNMVRDIMVRSITEKTADTERKNLLTLLMKVNENADPREL
QDTAVNFFIAGKDTTXFSLSWLIVMMNRHPRVLQKIREEIRTVLPELLTGKMDVPTREDTQKLVYLDAAVKESVRLWSV
STYRCTTRDTTLTSGGFIKKGTIVVVSKYAAARRRNVWGEDAAEYRPERWFDEKTGEPKKITPPQFITFSTGPRKCIGMR
LAMLEMKTVMAVLFSRFDIETVEDPFKITYDLSFVLPVKGPLAV

>CYP5015F6 P.parasitica

MLDSQSLSPLLATIFALLLVLWKVSTKPSPHTNGQKLFRPASTLPFLGNTLDLLWFQRDRLHDWMTEQSLACG
GKPWLLTGIGQVPKVVVTAAEAYEDVFKTQFDVFVRGPGETVKEVLSEGIFNVDGDKWKHQRRVTSHLFSMHMLKDC
MNAVVREKTVKLRDVLTKCAERGDTVSMKSLLNKYTADTFTRIGFGVELNGLDDPMDVDTTQPLDAALRVVQTRLQS
PVWLWKLRRFFNIGSERVMRESMQKVHDTVEQIMTKSLEDKNQASTSVHKDLMTLMLQSGDFTDTREIRDVAVNFY
AAGKDTTAFSLSWFIVMMNRHPRVLTKIREEVHRVAPGLSTGELDTPTLEHLQQMTYLEAALKESLRLNALVVYRLANR
DTTMYDGTFVPKGARVVFSMYGSARQPTVWGSDAAEYKPERWIDKKTGKLKTISSYQFTTFSAGPRQCIGMRLAMM
EMMTVLSVVFSRFDLVTVDNAFDITYDFSLVLPVKGPLDVRVRSLAAHVA*

>CYP5015F7 Phytophthora capsici

MLNPQFLPTLLATVFAILLVVWWKLLSYKSPHVNGRQLFRPGSTLPILGNTLDVLWLQRNRLHDWMTEQSLEN
GGKPWLLSGIGQRSKIVVTAPAAYEDVFKTQFDVFVRGSGETVQEILGEGIFNVDGDKWKHQRRVISHLFSMHMVKD
CMNAVVREKSVKLRDVLAKCAERRDPVSIKSLLNKFTADVFTSIGFGVELNGLDEPVDVDTSQPLDTALGVVQIRLQSPV
WLWKLRRFLNVGSERVLRESMLKVHNTIEQIMTKSLEDKEKTSKSTSPHKDLMTLMLQSGDFTDAREIRDVAVSFYAA
GKDTTAFSLSWFIVLMNRYPHVLCRIREELRCVAPGLFTGELETPSLELLQEMTYLEAALKESIRLYSLTVYRLANRDTTLYD
GTFVPKDVRVVFSMYASVRQPTVWGPDADQYKPERWIDEKTGKIKNISNLQFVMFSAGPRQCIGMRLAMMEMMT
VLSVVFSRFDLVTVVDPFEITYDFSLVLPVKGPLAVRVRPLAAHLA*

>CYP5015F8 P.infestans

MTEQSLASGGKPWLLTGIGQLPKIVVTAAAAYEDVFKTQFDVFVRGPGETVLEVLGEGIFNVDGDKWKHQR
RVTSHLFSMHMLKDCMNAVVREKTVKLRDVLAMCAERGDSVSMKSLLNKYTADTFTRIGFGVELNGLDEPVDVDTTQ
PLDAALRVVQTRLQSPVWLWKLRRKLATSDTPHRDLMTLMLQSGDFTDAREIRDVAVNFYAAGKDTTAFSLSWFIVM

MNRHPLVLCKIREEIRRVAPGLFVGELDTPTLEQLQQMTYLEAALKESLRLHSLAVYRLANRDTTMYDGTFVPKGARVV
FSMYGSARQPTVWGADAAEYRPERWIDEKTGKMKTISSFQFVTFSAGPRQCIGMLWP*

>CYP5015-fragment1 P.ultimum
MSMLSTVAAIVLSTLVAGLLFVVVPPLPCATSTLPVLGSTLDILVTHRDRMYDWVTDQCELLEGRPWAMYIFG
RDPGVVISSPELLEDVLKTQFDQFPKEDNICTVFRDFFGRGILATNMLRGAMHEVVSEKVRTPFDVLTEYEARHEAMSL
KSVLSHFTSDVFARIGFDVEQRSMENRVNGHTINDFCRCIQDYLPRRVHAFFVADVVLAPEALFQPRRRELAESVKFIDF
LVYNIMGESIARNTQRTRTQATSMSEHSGGATRQGGYVSPTEVGGAKDLVPMFLESNSLTESSDIDAQAIRDMVMCFI
STGTDTTSPSMSFFLVMVNWYSPVLKKCERN*

>CYP5015G1 P. sojae
MWTLSQHATFDKAAATVALVTAAYVGWNVVSAVVARRAVNRVLADQGVYEPPSLPVLGHTLDLMHNKDR
FHDWFAEQCLAAGGRPWVLRIIGRPPTLVLTSPQEIEDVFKTQVDIFEKGLDIREIGHDFFGDGIVGVDGEKWQKQRRT
ASHLFSVGMLRDVMDAVVMEKTLQLRDVLAECARVNRPVSMKSLLAKLSSDVFTKIGFGVDLNGLGGDVDDDMEHP
FIKAVETYGSVFQSRLQSPMWLWRLKKRLGVGEEGELRKARVIVHDLVMEIMKKSMASKNSATGSKQQKDLITLFMKT
MDSSADVMEVRDAVMNFFLAGRDTTSFSMSWMIVNMNRYPRVLEKIRAEINANLPELLTGEIQAPSMADLQKLPYLE
AAMRESLRLYMATVHRAPNRSTTLSGGLHVPFGTHVIVPTYAMGRMPTVWGEDAAEYRPERWIGEDGRVLKVSPFKF
FSFLAGPHQCLGMRFALLEMQTVMAVLLSRFDIKTVENPFEITYDYSLVIPVKGPLMANIHDRSTSVAASS*

>CYP5015G1 P. ramorum
MWGIAQHQVNERQAVIAVGALSGLYLGYKLLSAVYSDMKITRALDSQGLHRPKSTLPILGNTLDVMFFQKDR
LQDWMADQSQISDGKPWVLSIIGRPQTLIITSPEACEDVFKTQFDNFGRGDELVDLQHDIFGEGVAGVDGEKWLKQR
RIASHMFSMKMLRDVMDEVIIEKSKKLRDVLAACAKEGRIAPMKSLLGKFSSDVFTKIGFGVDLHGLDGDINSEMDHPF
IEAVDGYAEVFGARLQSPMWFWKLKRFLNIGDERMLKRCIKVATDLLNDVMLKSMSNKTAEDWNSKTDLLTLFVDST
GNTDSSDLRDAMMNFFLAGKETTSFSMAWIIVNLNRHPRVLAKLRAQIRENLPELLTGELEVPTMEDLQKIPYVEAVLK
ESLRLNMTGVHRTPMRSTTLSEGTFVPFGSYVVMSVYAAARVKNVWGEDAAEFNPDRWIDEETGKVKFVNPFQFITF
GGGPHQCLGMRFALLEMQTVIAVLFSRFDIKTLEDPFKITYDYSVTLPVKGPLECAINEVAAPAF*

>CYP5015G10 P.parasitica
MLKLHGRDLHDRIDGISWEVLALWWSPLLGDLLPTAYSGGLIRAMWTLSHHDAAAAIAFVTAVYVGWNVA
SSVAARRAVNKVLADQEIYEPPSLPVLGHTLELASNKDRFHDWFTEKSLAAGGRPWVLRIIGRPPTLVLTSPKEIEEVFKT
HVNIFEKGPDIREIGYDFFGEGIVGVDGEKWRQQRRTASHLFSMNMLKDKMDAVVIEKSLQLRDVLAECARLNKPVS
MKSLLSKLSSDVFTKIGFGVDLNGLGGDVDVEMEHPFIKAVETFGYVFQSRLQSPMWLWRLKKRFGLAEEGELRKAKKI
VHDLVMEIMKKSITDKNAATSSKQEKDLITLFMDTMDSTADVMEVRNAVMNFFLAGKDTTTFSMSWMIVNMNRYP
RVLDKIRAEINSNLPELVSGELEAPSMSDLQKLPYLEAAMRESLRLYMATVHRAPNQSVTLEGGLHVPFGTHVIVPTYA
MGRMTNVWGEDAAEYRPERWIDDDGNVIKISPFKFFSFLAGPHQCLGMRFALLEMETVMAVLLSRFDLKTVENPFDI
TYDFSVTLPVKGPLECMIHDRSASIAPSS*

>CYP5015G11 P.parasitica
WTLSQHDAAAAIAFVIVVYVGWNVASSVVARRAVNKVLADQVIYEPPSLPVLGHTLELANNKDHLHDWFTE
KSLAAGGRPWVLRIIGRPPTLVLTSPKEIEEVFKTHVNIFEKGPDIREIGYDFFGEGIVGVDGEKWRQQRRTASHLFSMN
MLKDKMDAVVIEKSLQLRDVLAECARLNKPVSMKSLLSKLSSDVFTKIGFGVDLNGLGGDVDVEMEHPFIKAVETFGYV
FQSRLQSPMWLWRLKKRFGLAEEGELRKAKKIVHDLVMEIMKKSITDKNAATSSKQEKDLITLFMDTMDSTADVMEV

RNAVMNFFLAGKDTTTFSMSWMIVNMNRYPRVLDKIRAEINSNLPELVSGELEAPSMSDLQKLPYLEAAMRESLRLY
MATVHRAPNQSVTVEGGLHVPFGTHVIVPTYAMGRMTNVWGEDAAEYRPERWIDDDGNVIKISPFKFFSFLAGPHQ
CLGMRFALLEMETVMAVLLSRFDLKTVENPFDITYDFSVTLPVKGPLECTIHDRLVSSS*

>CYP5015G12 P.vexan

MWGHAQFAAVTEQPAATTALVSAAALAALYSSYHVVKSVLADRAIRRVLAKQGIHQPSSTLPLLGNTLDTQF
DIFGRGDELLDVLHDLFGDGIVGVDGAKWKEQRRVGSHLFSMQMLRDVMDEVVIEKTQQLCDVLSSCAKQGKVVSIK
SLLSKFTSDVFTKIGFGVDLHGLSGDVDVEYEHPFIQAVAGFGPVFQSRANSPMWFWKLKRFLSIGDEGQFKKDAKIVH
DLIRDIMVESMANKTAKARDSKTPPRRDLLTLYVESSGTTDVMAVRDIVMNFFLAGKDTTSFSMSWFFVMLNRHPRVL
HKIREEIRINLPTLATGELKVPTMEDLQKLPYLEAAIKESLRLYMSAVHRTPSRSTTLSDGTFVPFGTYVIMSAYAAGRMTS
TWGPDATEFRPERMLDPETGKVKNFSPFKLVSFIGGPRQCLGMRFAILEMKTAMATVLSRFDITTIEDPFDITYEISLVIP
VKGDLRVTVNELAPSPAAS*

>CYP5015G13 P.parasitica

WGLTQHQVNQREATFAVGALGALYVSYKWLIAVYRANAMTRAFDAQGLHRPKSTLPILGNTLDVMFYQKE
RLWDWMAEQSNLNDGKPWVLSIVGRPDALIVTSPEACEDVFKTQFDNFGRGTELRDVIYDIFGDGIAGVDGEEWLKQ
RRIASHLFSMKMLRDTMDAVIIEKVTKLKEVLADCAKQDKVVPMKSLFGKYTSEVFTKIGFGVDLDSLESDPCSDSNNAF
IKAVDVYAEVFGARIQSPAWFWKLKRLLSIGDEGRLKQSAKVAEGLTQEVLAKSLEIRRRDSGEVKRTDLLTLFVENNTNI
DPKAVHDTLMSFLLASKDTTSFSLSWVLVNLNRYPAVLAKLRNEIREKLPGLMTGEIQVPTMEDLQKLPYLEAVVKESLR
LHMAATNRMTNTSTTLSDGTFVPEGCAVMVPIYASARVKSVWGEDAAEFKPERWIDPTSGKVKPVSPFKFFTFAAGP
RQCLGMRFALLQIQTTVAVLFSHFDLKTKENPFDITYDFAITLPVKGQLNVTVREITPAAY*

>CYP5015G14 H.arabidopsidis

MVTLALDGQGLHRPKSTLPILGNTLDVMFFQKDRLQDWMADQTQLSDGKPWVLSIIGRPQTLIVTSPEACE
DVFKTQFDNFGKGEELVDLQHDIFGEGLAGVDGEKWLKQRRIASHMFSLKMLRDVMNEVIMEKSMKLRDVLAQCAK
EGRIVPMKSLLGKFSSDVFTKVGFGVDLHGLDGDVNSEMDHPFIKAIDGYAEVFGARLQSPMWFWKLKRFLNIGDER
MLKQCIKVVTELLNVVMLKSMTNKTAEDWKTKTDLLTLFVDHTGKTAPSDLRDATMNFFLAGKETTSFSIAWVIVNLN
RHPRVLAKLRTQIRENLPELMTGELQVPTMEDLHKIPYLEAVLKESLRLHMTGVHRTPVRSTTLREGTFVPVGSYVVMS
VYAAARVKKVWGEDAAEYNPERWIDANTGKIKHVNPFQFITFGGGPHQCIGMRFAMLEMQTVMAVLFSRFDIKTVK
DPFEITYDYSVTLPVKGPLECTVREVNAPAY*

>CYP5015G15 P.parasitica

MWGIAQHQVTERQAVLTAGALSGLYLGYKLLVAVYKEMKITRALDSQGLHRPKSTLPILGNTLDVMFFQKDR
LQDWMAEQSQISEGKPWVLSIIGRPQTLIITSPEACEDVFKAQFDNFGRGDELVDLQHDIFGEGVAGVDGEKWLKQRR
IASHMFSMKMLRDVMDEVIMEKSIKLRDVLAQCAKEGRVAPMKSLLGKLSSDVFTKIGFGVDLHGLDGDINSEMDHP
FIEAVDGYAEVFGARLQSPMWYWKLKRFLNIGDERMLKRCIKVATDLLNEVMLKSMANKTAEDWSTKTDLLTLFVDH
TGKTDSSDLRDAMMNFFLAGKETTSFSMAWVIVNLNRHPRVLAKLRKEIREKLPELMTGELQVPTMEDLQKVPYIEAV
LKESLRLYMTGVHRTPMRSTTLRDGTYVPFGSYVVMSVYAAARVKNVWGEDAAEYNPDRWIDEETGKIKFVNPFQFIT
FGGGPHQCVGMRFALLEMQTVIAVLFSRFDIKTLEDPFKITYDYSVTLPVKGPLECTIHEASAPAF*

>CYP5015G16 P.infestans

MWGIAQHQVTERQAVLTAGALSGLYLGYKLLVAVYKEMKITRALDSQGLHRPKSTLPILGNTLDVMFFQKDR
LQDWMAEQSQISEGKPWVLSIIGRPQTLIITSPEACEDVFKAQFDNFGRGDELVDLQHDIFGEGVAGVDGEKWLKQRR

IASHMFSMKMLRDVMDEVIMEKSIKLRDILAQCAKEGRVAPMKSLLGKFSSDVFTKIGFGVDLHGLDGDINSEMDHPFI
EAVDGYAEVFGARLQSPMWYWKLKRFLNIGDERMLKRCIKVATDLLNEVMLKSMAEKTAEDWNTKTDLLTLFVDTTG
KTDSSDLRDAMMNFFLAGKETTSFSMAWVIVNLNRHPRVLAKLRQQIRENLPELLTGELQVPTMEDLQKVPYIEAVLKE
SLRLYMTGVHRTPMRSTTLREGTYIPFGSYVVMSVYAAARVKNVWGEDAAEYNPDRWIGEETGKMKSVNPFQFITFG
GGPHQCVGMRFALLEMQTVIAVLFSRFDIKTVEDPFRITYDYSVTLPVKGPLECTIHEASAPAF*

>CYP5015G17 Phytophthora capsici

MWGIAQHQVNERQAVFAVGALSGLYLSYKLLSAVYKEMKITRALDSQGLYRPKSTLPILGNTLDVMFFQKDR
LQDWMAEQSQITEGKPWVLSIIGRPQTLIITSPEACEDVFKAQFDNFGRGDELVDLQHDIFGEGVAGVDGEKWLKQRR
IASHMFSMKMLRDVMDEVIIEKSIKLRDVLAQCAKEGRVVSMKSLLGKFSSDVFTKIGFGVDLHEVFGARLQSPMWFW
KLKRFLNIGDERMLKRCIKVATDLLNEVMLKSMANKTAEDWNTKTDLLTLFVDSTVWRGSS*

>CYP5015G18 Phytophthora capsici

MQKNYTAAHRSLVNKVIVLGVTSSPSSVSLGAISKYDPATQRLEISLLCINQAATCQYAEFDKAAAAVAFVTAV
YVGWNVASTVVERRAVNKVLADQGVYEPPSLPVLGHTLELASNKERFHDWFYEKSLAAGGRPWVLRIIGRPPTLVLTSP
KEIEEVFKTHVDIFEKGPDIRDIGHDFFGNGIVGVDGEQWRQQRRIASHLFSMNMLRDKMDAVVIEKALQLRDVLAEC
ARLHKPVSMKSLVSKLSSDVFTKIGFGVDLEGLRGDVDVEMDHPFINAVETMGYVFQSRLQSPMWLWRFKKRFGLAE
EGELRKAQKIVHNLVMEIMKKSTHK

>CYP5015G2 P. sojae

MWGISQHHERQAVLAAGTLSGLYLGYKLLVAVYKELKITRALDAQGLHRPKSTLPILGNTLDVMYFQKDRLQD
WMAEQSQVSDGKPWVLSIIGRPQTLILTSPEACEDVFKAQFDNFGRGDELVDLQHDIFGEGVAGVDGEKWLKQRRIA
SHLFSMKMLRDVMDEVICEKSLKLRDVLAQCAKEGCVVPMKSLLGKFSSDVFTKIGFGVDLHGLDGDINSEMDHPFIEA
VDGYAEVFGARLQSPMWYWKLKRFLNIGDERMLKRCIKVATELLNEVMLKSMASKTAEDWNTKTDLLTLFVDTTGKT
DSSDLRDAMMDFFLAGKETTSFSLAWVIVNLNRHPRVLAKLRAEIREKLPGLMTGELEVPTMEDLAKVPYIEAVLKESLR
LYMTGVHRTPMRSTTLREGTFVPYGSYVVMSVYAAARVKKVWGEDAAEYNPDRWIDEETGKIKFVNPFQFITFGGGP
HQCIGMRFALLEMQTVIAVLFSRFDIKTVEDPFKITYDYSVTLPIKGPLECTVHEATAPAY*

>CYP5015G2 P. ramorum

MWGLAQHQGNQREAMLAVGTVSALYVSYKVLSAMYKSNSIGRAFDAQGLHRPRSTLPLLGNTLDVMFYQK
ERLWDWMAEQSNLSDGKPWVLSIIGRPDALIVTSPEACEDVFKTQFDNFGRGADLRDVIYDIFGDGIAGVDGEEWQK
QRRVASHLFSMKMLRDVMDEVIIEKVTKLREVLAGCAKEGKVVPMKSLFGKFTSEVFTKIGFGVDLHSLESDPCCDSNN
AFIKAVDVYAEVFGARVQSPAWFWKLKRFFSIGDEGRLKESAKVAEGLTQEVLAKSLEARRRDSSEVKRTDLLTLFVETN
TNIDPKAVHDTLMSFLLASKDTTSFSLSWVLINLNRYPAVLAKLRDEIREKLPGLMTGEIKVPTMEDLQKLPYLEAVVKES
LRLYMAVTNRMAKTSTTLSDGTFVPEGCAVMVPIYASARVKNVWGDDAEEYKPERWIDLSTGKVKPVSPFKFFTFAAG
PRQCLGMRFALLQIQTTVAVLFSHFDLKTQENPFDLTYDFAITLPVKGPLNITVRDIVPAAF*

>CYP5015G3 P. sojae

MWGLAKHQVSEREAALAVSALGALYVSYKLLSAMYKSGSMARAFDAQGLYRPKSTLPILGNTLDVMFYQKE
RLWDWMAEQSILQEGKPWVLSIVGRPDALVVTSPEACEDVFKTQFDNFGRGTELRDVIYDIFGDGIAGVDGEEWQKQ
RRVASHLFSMKMLRDVMDEVIIEKVTKLKDVLAECAKQGKVVPMKSLFGKFTSEVFTKIGFGVDLRSLESDPCSDSNNA
FIRAVDVYAEVFGARVQSPAWFWKLKRFLSIDDEGRLKQSAKVAGGTQQVLAKSLEVRRQDSSDAKRTDLLTLFVEANT
SIDPKAVHDTLMSFLLASKDTSSFSLSWVLINLNRYPAVLAKLRDEIRANLPGLMTGEIKVPTMEDLQKLPYLEAVAKESL

RLHMTASNRMANTATTLSDGTFVPEGCAVMIPMYASARVKSVWGEDAAEYKPERWIDAATGKVTPVSPFKFVTFGA
GPRQCLGMRFALLQIQTTMAVLFSHFDLKTTEDPFDLTYDFAITLPVKGPLNVTVREITPAAY*

>CYP5015G4 P. sojae

MWTAQNHATTSSAVLLTAATLGSLYAGWKVATALYSQRVLDAALTKQKLHSPDSTLPVLGNTLDLLFFQRER
LWDWVTEQSAISGGKPWVLRIIDRPTSLVVTSPETLEDIFKTQFETFERGADMRELFYAFVGDGIVGADGEQWVKHRR
TASLMFTTRTLREVVDAVAKEKSLQLRDVLSECAKQGRVVSMKSLLTKFSGDAFTKIGFGVDLNSLGGNVESAMDHPF
MEAVEVYAEVLCTRLLSPTWLWKLKRFLNVGDERALKHANKIVHDLTYEVMRESMEKKTHGEGMALQQKDLLSLFM
QSGDTVDVQVVRDSVMNFLLAGHDTTSFSLSWVVINLNRYPDVLAKLRTEFRERLPGLMTGEIDVTTYEDLQNLPYLEA
VVKESLRLYVTAVNRVANQSTTLSDGTFVPLGCGIMVALYAAARMKNVWGEDADEYKPERWIDPKTGKVKNVSSFKFI
SFIAGPRQCIGMRFALLQMRVAIAVMFSRFDLKTVEDPFKLTYDIAFTLPVKGPLNVSVHELA*

>CYP5015G5 P. sojae

MLASLYDVVFSVASLAALYAAWRVGSRVYSQRIIDVALANQKLHSPPSTIPLLGNTLDALFLQKTRFWDWIAE
QSELSGGKPWVLRLVGRPTTLVCTSPEALEDIFKTHFDTFERGADLRDLLYDFFGDGIVGADGENWQKQRPLRDAMAT
VVKEKALHLRDALAKCAKEGRTVDMKSLLEKFSGDTFTKIAFGVDLNGMESDHPFNKAVDVMSETLDSRLLSPTWLWK
MKRFLNVGDERKLKEACAIVHELTHQVMTESMQQQQKKKNKNKSDVLTLLLDSSGDLDVAVVRDAVMNFLLAGKDS
TIFSLSWILVNLNRHPEVLRNEINEKLPGLVSGEMDAPTMDDLKDLTYVEAVVKESLRLHGIATTRVPKKSIILSDGTFAPA
GCAVMMPAYASARLTSVWAMDRPRVKPVSPFKFGTFIAGPRQCVGMRFALLEMRLVTAVLFSRFDLKTVKDPFEISYE
YAFTLPIKGPLLDVTVRAVSAA*

>CYP5015G6 P. sojae

MLASLYDVVFSVASLAALYAAWRVGSRVYSQRIIDVALANQKLHSPPSTIPLLGNTLDALFLQKTRFWDWIAE
QSELSGGKPWVLRLVGRPTTLVCTSPEALEDIFKTHFDTFERGADLRDLLYDFFGDGIVGADGENWQKQRRAASTTRAL
RDAMATVVKEKALHLRDALAKCAKEGRTVDMKSLLEKFSGDTFTKIAFGVDLNGMESDHPFNKAVDVMSETLDSRLLS
PTWLWKMKRFLNVGDERKLKEACAIVHELTHQVMTESMQQQQKKKNKNKSDVLTLLLDSSGDLDVAVVRDAVMNF
LLAGKDSTIFSLSWILVNLNRHPEVLRNEINEKLPGLVSGEMDAPTMDDLKDLTYVEAVVKESLRLHGIATTRVPKKSIILS
DGTFAPAGCAVMMPAYASARLTSVWGEDASDYKPERWIDPRVKPVSPFKFGTFIAGPRQCVGMRFALLEMRLVTAVL
FSRFDLKTVKDPFEISYEYAFTLPIKGPLLDVTVRAVSAA*

>CYP5015H1 P.iwayamai

LDLLFTYRVKFYNFFLNETLKQKGKLWRLKVPGQPVVVMLTTPEASEHMLKANFDDFDKGPRFWTTMEDILG
NGIFIMMHDMMEQSVIEYTTILCERFDQISANNEVVNFKRLLDTFAMEIFSKIGFGIELNGLKSDENKAFAGDAFERASR
NLFVRYQLPIWFWKLKKWLNIGSERVMAEDHDKQQSTGRDLISLFLDKEANKYANGETPKTDTPLIRDMAISSPTASRD
TTSQSMMWLILALNRHPHALKTIREELRDVQQLTYLEAAIRENLHLNSVVANNSRMSVHDRGAIGLRACPLTVGLGRQ
RGRVQSRAWIDAETGKLIQVSLTKSQAFLSGPRTCVGMNFALMEMKIAVATVLSKFDVKTVKDPFDFTYHATVTMAIK
GPVDVAVTSLALKN*

>CYP5015H2 P.iwayamai

LDIDALTTLLTAPTVATAAAVALLGYLALPDARERAVSHLPRPETTLPILGNTLDVTMRYAGRFYDLLLDECIRQK
GKPWQLKIVGKPVIVVVCTPEEFENVLKTKFDDFGKGAYLTNVLSDILGNGVFAVDGMLWMHQRKTASHLFSLQMM
RDAMERSVIEYTSKLLTRLDAIECGNEVVNMKRLLDVFTMDVFTKIGFGVDLRGLEDDTQPPFFSAFERASANLLTRSIEA
LPLWQLKKKLNIGSERDMAHDIKVIDDMVYDIIAQSMNGKREAGDGVKDLITLFLKKEETEYANGEKVTTDPKFIRDMA

LNFVAAGRDTTLRMNPVLAFTSRIALRDTTLHDGTFIKEGTRVALPHYALGRAPWVWGDDANEYKPERWIDAETGKV
QQVSSYKFSAKFEITTERDPHAIKYRQTATMSIDGPLNVRATRLAVAGGA*

>CYP5015H3 P.ultimum

MLDSAAFFTALCEALTPSALTLTCIFAIIGYLLVPDAKLRAVSHLPTPKSSLPILFNMLDLMFTYKGRLYDFLLDNS
RLNDGKPWRMKVPGRPVTIVICNPEAYEDLLKTKFDDYGKGEMQHRNMEDILGNGIFAVDGMLWMHQRKVGSHLF
SLQMMRDTMEHVVREYVAKVNNHLEQVYKNNEEVNFKRLLDLFTLDVFIKIGFGVDLKGVETNTNGPFMDAFERAS
MNMLVRSTEALAIWPLKKWLNIGSEKQNKKDMKVINDFVYRIIERSMENTRKKREATASGSALPTAAPAKDLVSLFLEK
EQTEYANGETVKTDPKFIRDMTIAFLTAGRDTTSQSMSWFVIFLNRYPHVLKKIRAEIKEKLPNLANGTTLIPSMDDVAQ
LTYLEAALRENLRLNPVLPLNIRMALRDTTLYDGTVIKEGTRFIFPHYAMGRLERNWGPDAAEFKPERWIDPDTGKIIQV
SPFKFTTFNSGPRMCLGMKFAFMELKITMASILSKFEVTTVRDPFSYEYLATATLSIKGPLNVKVKSLDA*

>CYP5015H4 P.irregulare

NDTSGAFISSLSPSPVALSSAVVVAVLLGYLLLPNTVERAVRHLPRPATTLPIFHNSFDIVLRYPGRIYDFLLDETR
KQNGKTWIVQAMGRPVTIVLSSSEAFEHVLKTKFEDFGKGEINYITLFDVLGNGIFAVDGVRWLHQRKTASHLFSLQM
MRNTMEEAVKEYTALLCGRLEEISKNDEVINIKRLLDLFTMDVFTKIGFGIDLQGLTSNKNPEFLDAFERASKNLLARFQA
PMWAWQLRRWLKIGKEKQMAKDVKVINDLIFDIIARSMDRRKKQTKDISGMAENESEGRKDLISLFLEKEATEYGASG
ERAETDPALIRDMTVSFIAAGRDTTSQSMSWFLLMLNRYPSVLKRVREEIKEKLPGLVDGSMLVPNMEDVAQLIYLEAA
IRESIRLNPVVALNARTALRDTTLHDGTFIKAGTRVALPHYAMARLTSVWGEDAEEYNPDRWIDAGTGKLIQMSPFKFT
AFIGGPRMCLGMKFAFMEMKIAMAAVLSKFDVMTVKDPFAFTYSPSITLTIKGPVDVRVQSIRDK*

>CYP5015H5 P.ultimum

MGEDSTLHALCTPAVLVVTSVAVLLGYLAFPSAAERATRKQNGQMWRIKAMGRPVTIVLTSPAAFEHVLKTR
FEDFGKGELNSVTLYDVLGKGIFAVDGMLWMHQRKTASHLFSLQMMRDTMEHAVMHYTEILCKCLAGIAETQETVNI
KRMLDLFTMDVFTKIGFGVDLHGLESNKNPEFLDAFERASLNLLSRFQVPVWSWQLRKWLNVGKEKQMAEDVKVIN
DFTLNIIKQSMEETRTKSDADAKAKDLISLFLEKESTGYSNGAYNSSINTNATLIRDMTMSFLAAGRDTTSQSMAWFLL
MLNRSPSILTRVRQEIYDKLPKLRNSTTAIVPSMKDVQQLTFLEAALRESIRLNPVVAMNARTALRDTTLHDGTFIKAGTR
VALPHYAMARLTQVWGDDAEVYNPDRWIDRATGKLIEVSPFKFTAFLGGPRMCLGMKFALLEMKIAMAAVLSKFDFT
TERDPMKYTYRPSITLTIRGPLNVTVRSVDAAAGTEQ*

>CYP5015H7 P.ultimum

MLSSASFAVLLGAADFALMTPGVLGLALAVALLGYALLPSAQERALRHIPQPATTLPILANTIDFTQSASRLYDF
FLDETQRQHGKMWRYRAIGLPVTLVLTTPEAYEDVLKTKFDDFAKGYVMWKNMEDLLGNGIFTVDGKLWVHQRKTA
SHLFSLQMMRDSMEQTVIHYTAILQSRLEDIAHNNEVVNLKRLLDLFTMDVFTKIGFGVDLNNLKSNENHEFLNAFERA
SSTLLARFTAPMWVWQVKKWLNIGSEKSMAKDIKALNTLIFDIISRSMANQTTAHVLAKIHEELKDKLPDLVNSKKLVPS
MEDVQKLVYLDAALKESLRLNPVVAVNSRIAMKIAMATVLSHFDVKTLQSAEVTVTQT*

>CYP5015J1 P.irregulare

LSSETFNQSLQLASEHYVAIAIALLVIYVCTPSAQERAVAHLPKPTSTLPLLGNTLDFVVTHREKLHSWFADECA
RQGGKPWVARVFGRAPMVMIYSEEGVKDVLKTQFDVFEKGQEFYDAMIDTMGETITVVNGAKWHHQRKVASNLFT
MNMLRDSMSHVVGYAGTQVCEIIGKAADAGNLIEFKYLMEIFTNDVFTKVGFXXXXLKHANHEFFTRVNRLSVVFKER
TMGAGGVWQLKRALNIGSEKQHKEDVAWVDEYVYNVINQSIAKKQQQQQNEQDSTTVGRDLVSLFLESNKVEDSVE
SQKAQFKSLRDMVMLFLFAGRSTTSNSMTWLLLMLAQNPHVQDKIRTELREKLPDLVSGKIQVPTKEQIGELTYLEAAI

RESMRLNPALPLMTRVAEQDTTLSDGTFMKKGTTVQLPIYAMARMKFLWGEDAAKYKPERWIDAKTGKLVVFSPYKN
LTFAAGPRICLGMNFSLMEMKMMASALLSKFEYTTEKDPYTYDYTATLILIINGPFNLRFKRIAPAAF*

>CYP5015J2 P.irregulare

MLSSETFNQSLQLASEHYVAIAIALLVIYVCTPSAQERAVAHLPKPTSTLPLLGNTLDFVVTHREKLHSWFADEC
ARQGGKPWVARVFGRAPMVMIYSEEGVKDVLKIQVDIFEMGSNEGITIAHGTKWFHQRKVASNLFTMNMMRASMS
EGIVNDGGLVCDIVGKAADAGETVELKRLMEIFTNDVFTKVGFGVDLNYLKHTNHGFFTCVDRVSELIKERTIGTGEIWK
LQRMLNLGLEKQRKEDMTWIDKYVYRIINKSIAKKKEDSVESQKAQIKSLRDMAIVFLFAGRSTATNSMDWLVIMLAQ
NLHVQDKIRAELRAKLPNLVSGTIKVLAMEQIGELTYLEAPIRESLRLNPAIPLTTRVAQQDMTLSDGAFLKKGTIVMVPIY
AIGRMQHLWGEDAAEFKSERWIDSKTGKFITVSPFKSFAFCAGPRICLGKNFSFMEMKMLTAALLSKFEFATEEDPHTC
EYHATLALNVVDPLE*

>CYP5015J3 P.irregulare

VFRFLHSLLLEATITPSSLFAGVLLLALLYALLPSAQDRAVAKLPRPSSTLPVLGNTLDAMFAQRMRLNDWFFER
CKQFQGVPWVFKVVGQAPMVVIYTEATVEDVLKTQFDVFPRGADFRDALRDLFGDGILNADGANWFHQRKTASYLF
TPQMMRDVMEGTITRHTERLCEIVAESAYKGDTLDIKHLLELYATDIFTKIGFGVDLQFLEQGNKEFFERFRRIGNIVLHR
NEQPMWLWKLMKVLGLGVERQQSEDIAWVDKFILSIIVDAISANTAKLPQQQETTAKRSSKDLIDLFINSSDNANTPLD
PTAIRDMAMSFISGGRETTAYGMSWFLIQMSKYPHIQSKIRAEIRDKLPGLMNGEIVVPTKEQTNALIYLEAAIKETLRLY
PVVPINVRIPLEDVTLVDGTFLKAGTKVIMPNYAMARMKSVWGPDAEEFNPERWIEPATGTIRTFSPFKFTTFSGGPRIC
LGRHFSLMEMKITLSSLLARFEIVTEKDPDSYAYDKSLVLSILGPVMVRATPVKGDAVAI*

>CYP5015J4 P.aphanidermatum

DDVFFSPIRLHALQPSLLIPVAAVAILLWLSVAPSRAHRYAAMLPSASSALPLLGDTLTAMFTHRRTLHTWLYH
ECQRFQGRPFVVRILTKPPMVVLSTVQAMEDVLKTHFDKFVKGYEFTEALRDLFGRGIFVTDGATWHRQRKTASHLFSL
HMMRDIMDAVMVEHSKQLCDIVGHAAKQGAVIDIKHLLELFTTDVFTKIGFGVDLRFMTSDQHEFFERFGRVNHNLL
YRQERPMALWKLQNWLGLGLEKRQREDIAWLDKFVYKIIEDSIARKAAEGMETTMTANKDLIRLFLDHPSTTNDAGEP
EYDVREIRDMAMAFISAGRDTTAYGMAWFVIMMDQFPDVQECIREELREKVPQLMTGEIKAPNKEQTANLVYLEAAI
KESLRLNPVIPLNTREASEDVVLSDGTFLAKGTQVLLPAYAMARMPSVWGPDAATFNPDRWIDTESGRLLAISPFQFLT
FSAGPRVCLGRQFSIMEMKIALAALLSKFRLAIDKDPRAFDYDASLTLGVEGPILLRPTLL*

>CYP5015J-fragment1 P.irregulare

VASETGKLVTVSPFKFFSFGAGPRVCLGKNFSFMEMKMSIAALLSKFEFTTEKDPHAFECHATLMLGVGGPLVI
RLK*

>CYP5015K1 P.iwayamai

MAVRQRERLYDWFLEQCELHKGPWRLKVLGRTPAIVIASPQLFEDVLKTNFEVFQRGESEVEILRDFFGDGIFT
SNGEAWHRHRTATAHLFGLPAMKTVMYDVLSEKVRILCDVVGEYALRGEEFSLKSVFGHFAADVFTKMALGVERNNL
ENGLRGYSDDFTASSRTISYAMQMRFHVPMWLWKLRRHYDLGLEKKFRECIKYTDFLIYPIINESLAKKSRANAAAKAKT
DASVVAAAETSRVSNSSFYSDTSSGSSGAESERDAEPPKQKDLISIFLENNPLTESLEVDAQVIRDTVINFISPGTDTTSHC
MSFFILMMNRYPKILKKIREELQAKLPQLFTDRIRTSAPKLTVEDLAKLTYLEAAIHESLRLNPQYAVTTREAIADTMLSDG
TFVRQGTRVVMAFYASMRRRSVWGEDAMRFKPERWIDLDTGRLITVSPYKFAAFFAGPRKCLGIKFALMEVKMTMA
VLLSRFQVKTSENPWFMTYQVGIALSVKGDMMVRATPFQATNSRMRA*

>CYP5015K2 P.irregulare

MLTLDQLIEALHAHPALISLATAVLGFVYLLLRGHQLNSDARLGEELRPKIRALRQLPCPPSTLPILRNTLDMAVR
QRERLYDWFLEQCELHKGPWRLQVLGRPPVIVIASPELFEDVMKTQFELFPRGENECEILRDYFGEGVFTVNGDAWHR
HRSTTAQLFALPAMKSAMYSVLCEKIRVLCHVVGVYASRDEEFSLKAVLSHFTADVFTKMALGVERNNLENGLKSYNDD
FIESSRTISYAMQLRFHQPMWLWKLQRKHDWGIEKRFKECIKFTDFLIYNLINESIAKKNLAPKQSQHSKQRAAPAPRDL
ISIFLEHNLLTDSLEVDAQVIRDTVINFISPGTDTTSHCMSFFILMMNRYPKVLRKIQGELQTKLPQLFYGNNNNGASTEP
SNLSAEDLSKLVYLDAAIHESLRLNPQYAVTAREANADTVLSDGTFVPKGTRVVMAFYAAMRQRSVWGEDAMRFKPE
RWIDLQTGRLITVSPFKFSAFFAGPRKCLGMKFALLEVKMTMAVLLSKFQIKTTENPWFLTYQVGLAVSVKGPVTVRAT
PFKKTSVAVSGI*

>CYP5015K3 P.iwayamai

GTLPILGNTVATLLYRHQFYDWHLENCERFQSPWRVHIFGRGPAVVIASPALFEDVLKTHFDSFPKDESMCEIF
RDFFGRGIFAVNGDEWRRHRHTASNLFSFQMLKRVMYEVVKDKTATLSDVLAEYAATKRTVSLKTVLNHFTCDVFVKV
GFGVEQRALESDLQGKPIDSFVSAATTISRVMYLRFLTPTWLWQLKRRWDTSDERQLRKSVAFIDAVAYKIINKSILEKH
RFRQEVRSRSFNGHDQSPHDVELPTRDLISMFMESNALASPSSDPQEQEEEEMDPKVIRDMVVSFFASGSDTTCQSLL
SFIMMMNRYPRVLKKVRTELRAKLPGLQHKDAELPSMADLSCLIYLEAVIRENLRLNPAIPVTTRAASVDTVLSDGTFVP
RGTRVVLSIYSLMRSRVVWGDDAL*

>CYP5015K4 P.irregulare

DASISWLKALWGQDAAATLFRSSSAITTVTAVVLGTLFLVSVKVLVARNDPLSKLPRPAYTLPLLGNTIEIMGW
QRNRMYDWVAEQCEIHRQPWRMHIIGRGPSIVLATPELFEDVLKTHFDQFPKDESMCTIFRDLFGHGIFAVNGDEWR
RHRHTASNLFSFXXXXXXXXXXQEKAQVLSDVFAKSESRGEQVSLKAVLNHFTCDVFAKIGFGVEQGSLEGDLRGKLVN
DFVDAAITISRVMYNRFLTPTWLWQLKRRFSLSDEQHLRKSVQLIDSLVYKIINASILRKHRLEKEAQEYDEKPPPLTQNLI
SMFMETNLLTGAEEEVDPKAIRDMVVSFIAAGSDTTCQSMLSFVMMMNRHPQVLKKVRAELKLKLPFLGHDDAKLPT
MQDLACLIYLEAAVRENLRLNPAIPVTTRSASEDMVLSDGTLVPKGTRVVMSFYATMRSKAIWGEDALEFKPERWINP
QTGGLITVSPFKFSAFLAGPRACLGKKLGKIELKMMLAVVLSRFDLTTVENPHEMTYQTGLTASVRGPMMIDVTRIGCD
AGAEATKRSTSAWNLTQKNPSEDSVK*

>CYP5015K5 P.iwayamai

LSIEHLVTLAQDQPAWVVVPVIVTALLGVYMLLNLLSASRNKKKTSSSAFPTYPHPPSTLPLLGNTLDAIYTQKD
RLYDWMLEQHELHKRPWELAVIGYPLTIVVSTPEHFEDVLKTHFDVFPKDDSQCDIFSDVFGCGIVAVNGHAWHAQRK
ATSQLFTPQKIKESMYPVMQLKAQTLCAMMTKYEARGEPISLKRVLNHFSADVFAKGAFDVDLNCLENGVESDHENEF
AYATRVASQVLQERFHEPLWLWRLKRALNIGQERVLKENIRHIDSLVYNILEAAIEKKKKSTSGEPAAEAAAKDLIAALLD
TDNSKSELGGDLDAKTIRDMVVTFFMEGTDAAAQNMAFLVVMLNRCPRALAKIRQEIHTKFPGLAKGDLPSMEELGQ
LVYLEAAIRENLRLNPTAPASMRQADKDVVLSDGTFVAKGTRVILAFYAGMRSKAVWGDDANEFKPERWIDPATGTLI
PDSPFRFASFSAGPRVCPGMRFALAEMKISMALLLSRFDLTTVEDPWKITYGIAITCGVKGPLMVNIASVAM*

>CYP5015K6 P.iwayamai

AEQHELHQQPWQLAVIGYPLMLVVSAPEFIEDCDIFRDVFGFGIIAVNGAAWHAQRKATSHLSTSQKIKESM
YPVMQLKAKTLCAMMAKYEARGEPISLKRVLNHFTTDVFAKTAFGVELHCLESGVESDRENEFAYAARVASQVMHARL
HEPLWLWRLKRALNVGQERLLKENIAHIDALVYSIIEAAIEKKTGEPAAEATKDLIATFLETGGSTSAEPGGRGLEAKAIRD
SVVNFFIEETETSAQSMAFLVVMRNRSPRVLAKIRQEIDAKFPGLAAGEGELPTMEELGQLVYMEAAIRENLRLNPTAP

VAMRQADADVVLSDGTFVAKGSRVILAFYASMRSTSVWGEDALEFKPECWIDPATGALIAESPFKFPAFIGGPRICPGL
RFALTEMKLNMALLLSRFNLTTVEDPWEVTYEVAIACGVKGPLMVKVASVAL*

>CYP5015K7 P.irregulare

MFSIAQLLGALREDSAVTSVAFTALALFLVATQLRPKRDPSSSTSKLSSYDSLPRPASTLPILGNTLDAIYTQKDRL
YDWMAEQHELHKSPWLLSVVGYPPTIVVSTPELFEDVLKTHFESFPKADSQCDIFRDVFGYGIIAVNGEDWHAQRKATS
QLFTPQKIKDAMYPIMQLKVQTLCAMLTQYEARGEPISLKRVLNHFTTDVFAKTGFGVELNCLENGVKSDRENEFAYAT
RVASQVMQQRFQEPLWLWRLKRSLNIGQEKVLRKNIDHIDSLVYSILDTAIEQRASSSSTESTRKDLISTFLEERGADADL
KAIRDSVVNFFIGGTETSAQSMAFLVVMMNRYPRVLAKIREEIQTKFPGLARGDSDLPSMEELGQLVYLEAAIRENLRLN
PTGPVTMRASIDDVVLSDGTFVAKGTRIILAFYASMRSKSVWGEDVLEFKPERWIDPATGFLIAESPFKFPVFLAGPRICL
GMRFALTEMKLNMALLLSRFDVTTVENPWEMTYEVAIACGVRGPLMVNVAKIAPSS*

>CYP5015L1 P.irregulare

LSVAQMQSLVLEAPLASITILFLSLYILVLSKKSSPVRYARPASTLPILGDTLDAAIYQVDRIYDWLAEQSEVIGGP
WALSILGDPPNLIITSPELIEDVLKTQFEIFQRGDQDTDCMKELFGKGIFASGGDAWYFHRKTASNLFTGQMMRDVMY
RAVHDNATVLCNVLRVYASREPGTMSFKSVITQFTSDVFCKIGFGVDLKCLEAGVHGEKGNEFVDAFGNAMHVTFMR
YQQPAWLWHLKRYLNIGDEKLHRHSIDVINKFIFRIIDESTARTQSGDGQAAPPKDLVSLFLHSKVNESEGIKSRAFDSEV
QMIRDMVVNFLVAGEDTTANSISYFILMMNRYPHVLAKIRDEMRKHVPGLLTGEQAVPSMDELPRLTYLEAAIRENLRL
NPVVPWVSRQAKAATVLCDGTPIPYNTRIGMLVYATGRSKTVWGEDALDFKPERWIDADTGKLIAVSPYKFPTFIAGPR
QCLGMTFALMQMKCTLAVLLSKFDLQTVQDPWEVTYKAALTMAVKGPMMVKVAPLVGGERARSA*

>CYP5015L10 P.irregulare

HTRDFLFVSRPYAETIILNLAPLVERLTLALAILGIMAGALLAYALMNLLPRTKTAASKHAQPTSTLPLLGNILDAT
IFYSDQIYDWITEQCVTFGSRPWLRSITGCMPSLFISTSELYEDVFKTRFNSFQKEENGTHYFTDLFEKSMLTSDGDAWTF
HRKTASHLFSQRMLQDLMYETVREEIQTFCGVLKVYESRDQGAVSFKNAMAHSAGDAFGKIGFGVELNCLENSMNRE
KGNESVDAFSVSTAVIFLRFTQPVWLWRLERYLNIGFEGKNKEYIKVISMFIHRVISESVARKNNEAARPRDNVIQPPKDL
ISLFFNSKNQNQNLKGKALDSEAQLIRDTVVNFIFNRKDTTSNSMAFFIVMMNRYPAVLVKVRDELRKKLPRSPTGEF*

>CYP5015L2 P.iwayamai

MLSVTEQLEALAHKAPLLAIGGYVLFRLTRTPEHRYARPASTLPVLGDTLDAALYQVDRIYDWLADQSETLGGS
WALSILGDPPNLIITSPELIEDVLKTQFELFQRGAQDADCMGELFGNGILAANGHAWAFHRKTASSLFSNQMLRDVMY
DAVSANARVICQVLRKCDDQQPVSFKRVITRFTSDVLCKIAFGVDLHCLASDGSSAAGSEFVDAFATVMRTTFMRYQQ
PAWLWHLKRYFNIGDERLHRQSMDAINSFAFRIINDSIAQKKKTTAEATINPSASAAQSPRDLISLFLSSNARQGEGEDP
SVDANLIRDMVVNFIVAGKDTTSHSLTWFIVMMNRYPQALAKIREELRAKLPQLETGDAEEMLAPSLNDLPRLTYLEAA
IRENLRLNPPVPWVSRQANAATVLCDGTPIANGMRIGMLVYGTGRSKAVWGEDALEFKPGRWLDADNKIVSVSPFKF
APFIGGPRQCLGLKFAMLEMKCALAVLLSKFELTTVEDPWTITYEAALTMAVKGSLMVKVSSR*

>CYP5015L3 P.iwayamai

MLSIEQLMATVTSSQSPAANVLTALGALVGVHVLLRFLTTSKKKKAHFVYPTSSLPFLGSVLDMAIYHRERLYD
WITDESVAADGKPWIQAVPGVPPNVMICTPELFEDVLKTQFDKFERGDMDYFQDLFGKAILASTGDDWVFHRKTASH
LFSNQMMKDAMYAAVREKVETLCKALRIYESRDEPVNFKSVIMHCTTDMFGKIGFGNGLTGTKGNKFVEAFATSTQTI
FFRFLQPKYLNIGTERAYKSREVIDKFIFRVINESIAKKKELSASSLLGEAPVPPKDLISLFLNTTIKDEENVKGTAFDSEMHLV

RDTAVSFIFAGKDTTSHSMSYFVVMMNRYPEVLKKIRAEFRRNLPRSPSGELQVPTLDDLPQLTYLEAAIRENLRLNPAA
PLIGRHALEDLMLCDDTFFGKGISRQSANLRRSPDEVGLGRRRACVQA*

>CYP5015L4 P.iwayamai

MSIDQLVVAVEAQPTAVVVSAAIGALFSAHLLLRLTQKKPKSKLVVPKSSLPILKNTLDLMFFNRHRLYDWVAD
ECEAAGGKPWVQAIVGGAPSVVVSTPELFEDILKTQYESFHKGDTSYFNDVFGNGILAADGEAWAFHRRTASTMFSSQ
MMKDVMCEAVREKAEVLCKALRTYESRGDPVSFKTVIMHFTSDVFGEIGFGIDLKCLENGVEGKQGNEFVEAFSTSTAI
LFQRLLQPRWLWKLKRYLNIGTEKTNKEKHEVIDKFIYRIINESIAKKKTESESGAPSSSLSDGASAPKDLISLFLSSLGQDDE
SLKGTGYDSELQLIRDTVLNFIFAGKDTTSHSMSFFIVMMNRYPEVLKKIRDELQAKLPRNSSGEFQIPTMEDLPQLVYLE
AAIRENLRLNPSAPGLRKLAKKDLVLCDGTFIAKDGHVGLSIYASGRMKCNWGEDALEFKPERWIDPETGKLSVVSPFKL
TAFGGGRRNCIGSKFAMMEIKCTLAVLLSQFDFKTLEDPWTMTYVAALTMGVKGPMMVNVTSIEASA*

>CYP5015L5 P.iwayamai

MMRDVMYKAVEDKMKTLCDVLRTYEARDEALSFKNVITQFTSDVFGKIGFGVDLDCLQNGVAGKQGNEFV
NAFADATQAMFLRFVQPKWLWRLQRALKIGSERVLYENVQVIDQFIFRVINVSIAKKSVNDQAIDNKTPAVAPPKDLISL
FLNSSIKEEENVKGKAFDSEMHLIRDTVVNFIFAGKDTTSHSMWWFMVMMNRYPEVLKKVRAELNEKLPGLVSGEMV
VPSMDDLPQLTYLEAAIRENLRLNPVLPIGGRQAVKDTVLCDGTPIAKHTRVAPSIYTAARRTEIWGEDALEFKPERWID
AATGKLLVVSPFKYGYFFGGPRQCIGMKFAMMELKCTLAVLVSKFDLKTIEDPWNLTYDIALTMAVKGPLLVNVTPRTD
SAAIGTALPA*

>CYP5015L6 P.iwayamai

LPIEQLVAVTLGALVGAQVLLSLRGRKKSEYAYPQPASTLLILGNTLDAMIYNRDRIYDWITDHCLAFERPWLLP
AIGSVLCVTVSTPELFEDVMKTQFENFPKGDDETDYYRDLFGKGILAADGDIWYFHRKTTSNLFSNQMMKDVMYEAV
RDNLKTLYEALCTYESRGEPVSFKNVMTQFTSDVFGKLGFGVNLRCLQNGVDGKDGNEFIGAFAIATKILDKQFMQPK
WLWQLKRALQVGSEKELRENVEVIDQFIFRIIESIAKKNALDKSAGGGEDTSKDSKGLISLFLSRNVKAEEIAKGQAFGLE
MHVIRDMVASFIFAGKDTTSHSMSWLIVMLNKYPEVLEKIRTELRQQLPGLASGEMAVPIIDELPQLVYLEAAIRENLRL
NPVLPFSARQANKDTVLCDGTPIFKGTRVGIGIYVSARKTSVWGKDALEFKPERWIDPVTGKLAVLSSFKYAYFFGGPRQ
CIGMKFVMMELKCTLAVLLSKFDLKIVESPWKVRYEAALTMVVKGPLLVDVSSVATESA*

>CYP5015L7 P.irregulare

MLSVEQLVASVEKQPAVFTAVVGALLGGYALLSSTRKPTFKHAQPASTLPIIGNMLDVTVFHSERLYDWLTEQ
SLALGGRSWRLSIPGSVPILVVSTPEVFEDVVKTQYAKFQKEEDGTHYFTDLFGKGILTSDGEEWNFHRKTANNLFSHQ
MIRNVMYAAVREKMQTFCEALRVYESRSQPLGIKKIITQYTSDVFGKIGFGVDLHCLENGVSGAKGNEFVDAFAKSTHII
FLRFTQPVWLWRLKRFLRIGVEGENREHIKVVNRFIYRIIDESIERKNRYLAEAAGSSASITNSDSNSPLPPPKDLISLFLHSP
VKDAEELKGRAFDSDMQLIRDTVVSFIFAGKDTTSHAMAFFVVMMNRYPRVLEKIREELWQKLPRSPTTGEILIPSMDD
LPQLTYLEAAIRENLRLNPAIPLSPRQAKEDLVLCDGTPMKKGTRATLAIYTAGRLASTWGDDVLEFRPERWLDPETGKL
LMLSPFKAIQFFAGPRNCIGSKFAIMEMKCTLAVLFSKFDLKMVENPWDATYESAMNMTVKGPLLVQVVSLASASLAK
FT*

>CYP5015L8 P.iwayamai

MLSVEQLVAFTQDQPASVVVPMVITALLGTFVLLSLSRNNKKSSSSYAYPQPASTLPLLGNTLDMAYFHKDRIY
DWMADQCLSLQRPWSRAILGRAPMLVVSTPELFEDVMKTQFDVFTKGRGETEFYSDLFGKGILASDGDAWYFHRKTA
SNLFSNQMMRDVMYEAVRDKVKTLCDVLRTYEAKDEAVSFKAVITQFTTDVFGQIGFGVNLNCLQNGVAGNKGNEF

VDAFVATTRIIFLRFIQPRWLWHLKRSLNVGSEKILRENVEVINSFIFRIINESIVRKRAATDKDADHSPSSPPKDLISLFLNS
TVKEDEG*

>CYP5015L9 P.iwayamai
MLSIDQLVAAVEAQPTAVLVSAAIGAFFSARLLLQLTQRPKQELVVPKSSLPIIKNTLDLMFYQRGRIYDWMAD
ECEAAGGKPWVQAVAGSAPSAVISTPELFEDILKTQFECFYKGDVSQFNDLFGKGVLASDGEPWAFHRKTAGMLFSNQ
MIKDVMYEAVHEKVEILCKALRTYESRGEPGSFKTVMMHFTSDVFGKIGFGVDLQCLENGVEGKEGNEFIEAFSTSTQII
FYRALQPRWLWKLKRYLNVGTEKQNKECCAVINSFLFRIINESIAKKSEAGAPSSNLSGEGSSQPPKDLISLFLSRTTEDDE
SLKSKAFDSEMHLIRDTVLNFIFAGKDTTSHSMS*

>CYP5015L-fragment1 P.irregulare
ENFRRIDGNSRVWFAEPIWLWQLKRFLNIGIEKKNTESREVINKSIFRIINESIEKRNSSSSARTDEAKDASPPKD
LILVTFNSKAKEAEDLKGRTFNSEMHLVRDTMTLRDELWQKLPRSPTTGEILIPSMDDLPQLTYLEAAIRENLHLNPVVPL
ASRQAKKHVVLSDGTPIAKGTHVGTAIYATARLKSIWGGKMRTSKLLIVSPFKAMPFLAGSRQCIGMKFAMMKMKCAL
AVLLSKFDVKIVENP*

>CYP5015M1 P.aphanidermatum
MVLSLQELLTRVEGKRDGWLSVAVAAAVAASAAVLLLRSRSTNSHELPRPESTLPVLGNTLDTMKKQRARLYD
WFTDESLRHGGKPWQLSVVGRPRAIIATSPLIFEDVLTNEGGTFMKGYQSCFLLRDFFGHGILAVENDEWYFQRKTASY
LFSLQMMKDVMSDVIREKVALFNHVLLTHASRNDGMTPLSFRRAITHFTSDVFAKIGFGVELDCLKRSLEGDDENEFVS
AFSYCAQVIQLRLQQPFWLWRLKRFLNVGDEKKYKQSMRVIDDLMMDIISRSIAKKNELVANRQPARDLITFFLESRVD
EAMARAWRESHGGSELSQIRDMVVNFIFAGKDTTSNSMSWFIVMMNRYPHVADKIREELRAKMPALMTGKLVVPT
MDDLKHLTYLEAAIRENLRLNPPVAVTARTATCNTTLRDGTAINPGDRVVLASYASARQPSVWGEDAAAFRPERWIDQ
ASGQLISESPFKFTSFLAGPRMCIGRKFAMLEMKIALAVLMARFDLDMVEDPWAVTYEIALTCPVAGPLMARVKPLV*

>CYP5015N1 P.vexan
MLTVKELATSNVQAIAAATAVVAALAVLQWRWSSAKPDPFDMSHLPTPDSTLPIIGNTLDAGKKHKERLHD
WFTEQSAKFEGKPWLLKVIGRPPTLVLSSPDNFEDVAKRQADIFTRGDDSKSLSKDFLGRGIVAEDGQQWYFQRKMSS
HLFSMKMMQEVMHEVVREKVAVFCDVLEKHIARGGVVDMKRELMYFTSDVFGKIGFGLELHCLESGLEGKSHEFIEAF
AAASHGTMVRYQLPKWYWKFQRFFGLGDEGRLAKGMKVINDFTYKVISDSMEVKKEQAQQGGTSAHKNLISLFLTTG
TKYREEITGGDPKIEMEFIRDMAINFIFAGKDTTSVAMGWYIIMMNRYPEVLQKVRAEIAKKIPGLIDGSIKVPTIDELASL
TYLEATIRENTRLNTPAAVSLSIYASARQPTVWGPDAAEFKPERWIDEATDTVRPISPPQSFTFGFGPRRCPGRNMAM
MEMKISLAVLLTRFEFKTIENPFEFLYEIGITHLIKGPLMVNVSPITPAPSS*

>CYP5015N-fragment1 P.vexan
MEFIRDMALNFIFAGKDTTSVAMCWFVIMMNRYPKVLQTIRAEIANKVPGLMNGAIDVPTIDDLKPLTYLEA
AIRENMRLNAPAASTGRTAAADTILSDGTPIKKGTRVNMAIYASSRLPTVWGPDAAEFKPERWIDPEKDAVRLIGPPQT
FTFGFGRRICPGRNMAMMEMKIALAVLLSRFNFKTLEDPFAITYELALGVEHRPLEPHTTLPFPVLPATTHT

>CYP5015N-fragment2 P.vexan
MFTVADLTARLMAVDRSTALAIAVAAASGVAIVLLRSSDSDGLRRPDTTLPIVGNTIDLLFKNRHRLHDWVAE
ESVKVQAKPWVVSVVGRPPTVMISDPADFEDIVNHQRDSIQRGNMTFDIAGDFLGRGILTTEGTEWYFQRKVSSHLFS
MKMMQQVMHEVVREKAALLCNVLESYAERGEPVSLTRQLMYFTSDVFAKIGFGVELQCLENGLKGKTHGFVDAYVRT
SAIVKRRFEQPVWLWKLKRWLRVGEEGELYNQLKTVN*

>CYP5015P1 P.vexan

MKRIHRPASTLPVLGNTLDAMVYQRERFVDWMSEQSQITGGKTWLMTIAGQPPWYVISSPESYEDLLKRQV
NIFERGPTMSYIFKDFLGEGIIAVDGHKWLVQRKTVAHLFSNRMMREVMDKAIIEKAIKFRDVLEFCRQRGTPVSLKSLIS
RFTSDVFTKIGFGVELNGLDSTNVDVETEHPFIEAIAAMCRLLQARAQSPMTWWRLKRWLNIGDEREAKVHMKVVHD
LVHEIMLKSFAMKHESLENLAQSKPSEELHAPTAKTGDIISFYIESGLTDPQQLQDMAVNLFFAGKDTTSFSLSWLFVM
MNRHPHVLRKIREEIHAKLPELATGEIDAPTMEQLGELTYLEAAVRENLRLNTSMTTRTPNQDTTLSDGTFVPKGSVVY
VCHYASARLKSTWGEDAAEYKPERWIDPATGKLIHFSPFKFVTFIAGPRQCPGMRFAILELMTVAAVLLARFDIKTVEDP
FDLTYELSVVFPVKGRLMVTVDKAAPLSPAA*

>CYP5015P2 Phytophthora capsici

MWGLEKHANHQKAFLAGTAFVVVVLGWRALKRSVTLKAVDGSFRQIHRPESTLPLLGNTLDAMFSQRERFV
DWLADQSALASGKPWLMTIVGQSPMFVISSPQSYEDLFKTQRHIFERGATMSYIFKDFLGEGIIAVDGHKWTQQRKSA
SHLFSNRMMRSVMSDEVREKCIKLRELLDICAKEGKTVSMKSLISKFTSDVFTKIGFGVELHGLDASKVDMENEHPFIQAI
DNMSRLLQLRCQQPIDASPLTTVEEPVLENPKTKTDNLLTFFLQSGLTNAQQLQDMAVNFFFAGKDTSSFVLSWFVVM
MNRYPEVLGKIRKEIRERLPGLLTGEIDVPSMEQLARLPYLEAAMRENLRLNTSMTTRSPNQDTTLSCGTFIPKDSIVYVC
HYASARLKSTWGEDAAEFKPERWIDPETGNVRQFSPFKFVTFIAGPRQCIGMRFAMLELRMVAAMLFSRFDIKTVDDP
FDLTYELSTVFPVKGRLLCTVQLAKSTS

>CYP5016A1 P. sojae

MMPSNLLNSRGLVAIPPGPLLGSTLALVVFWVMRHGRERRLSWTSTPLPPRGYRTLPTASASQSPVRVGMM
TDQPTEGSPAFYEWVCAMTSRFRGKPWLLHLPGRPDVLVVSSPTSFEDIQRTFALQFEKVDNDAEGLAHDAHGGAIAF
IYTGQVRPSVNMQRQLASSVLSSAALRQQASVLVKHHLQSLLRILDDTASSGDPLDVTRLMRSFAMEVFTELDFGLQLG
ALRSRRGECSDLEQAVDEVQRRVAERLKRPAVAWKLERLLDVGSEAALSRSVDVVSRITLGAVDTKRKRRRGGSPCDSP
IAGARVDMLDLLLSQKCSSKSSKDPEFLAEFVLGLVVAARDSMAHALSSCLQCLARHPEEQEKLARELKEAEEEDRDLQS
VVYLEAVVKEALRLYPAKPFIRRRARIDTVLSDGTFVAAGAKVAMDLYSMARRENVWGQNSAQFRPQRWIDSTNGKL
RPTSNYKFNAFLGGPRACLGADMAMTEMKTVLAKVIGRVHLDAVEPRMAEKDKTKAWDAACDAAVRVLVRRRGPS
PPGQYS*

>CYP5016A1 P. ramorum

MVPTNLLNTRGLVTFSPGPLLGSTVALMLLWAMRHGRERRLSWTSTPLPPRGYRVLPTASTSPVLVRVGMT
AEQPSEGSPAFYDWVFAITTRFHGKPWLLHRPGRPDVLVVSSPGSFEDIQRTFAVQFEKIEGDADGLAHDVHGGATAL
VCTGQLRPSVNMQRQLAASVLGSPALRQQASALVNQHLDSLLRVLEGAARLGTGNVSTDLDVSKMMRQFAMEVFTE
LGFGLQLGALRSPCYQASKLENAIDDVQRRMAERSQRPAVSWKLERLLAVGSEAALSRSIDVVSTITLEAVHAKRSKHRA
GSGCDSPLAGSRVDMLDLLLGQKCSSKSSKDPEFLAGFVLGLVVAARDSMAHALTKCLQCLAQNPEEQEKLLRELKEAE
EEGKDPRSIARLEAVIKEALRLHPSKPFVRRRARQDTVLSDGTFVAEGTEVAMDLYSMARRENVWGPGSAQFRPQRWI
DATNSKLRPVSKYKFNAFLGGPRACLGADIALAELKTVIAAVVSKVYLDAIEQSDGKDKALTRGVPCGEAMRIQVRRRDF
SRPGHYS*

>CYP5016A2 P.parasitica

MMSSSLLNARGLMFISPGPLLGSTLALVVFWAMRHSRQRRLSWISVPLPPRGYQHLPTASTAQTLVRVGMT
AEQPTEDSTTFYDWVLATTTRFRGKPWLLQRPGRSDVLVVSSPVAFEDIQRTYAVQFEKVNSEAEGLTHDVHGGAIALV
YSGHVRPSVNMQRQLAATVLGSSALRQQASVLVKQHINSLSRILDNNHALDMTKLMRQFSMEVFTDLGFGLQLGALR
SASMETSKFEKAVDDIHQRVIERKSRSAAVWKLQRLLDIGSEAALSRSVDIVNSVTLEAVEAKRKRRRAENPIAGSRVDM

LDLLLSQKCNSKSSKDPEFLAEFVLSLVVAARDSMVHTLTKCLQCLAQHPEEQEKLVCELKQAQEKGIDVQSVVRLEAVV
KETLRLYPAKPFIRRRARLDTVLSDGTFVAAGTEVVMDLYSMARRENVWGPDSAQFRPQRWIDTANNRLRPVSSYKFN
TFLGGPRACIGADIALVEIKTVIASVINSVQLDAIECDQSCDDKRLTLKIRRRDANLPGQYS*

>CYP5017A1 P. ramorum

MKMPWHLFFDGAIYVSSVLLQYQENLLWWSANRSLFVQITDPKDVQHILSTNVNNYVKPQGFLDAFQEIFL
NSFFALNHHPQAPDGGARWRLQRKVAAKVFTTANFRVFTEQVFARHADKTLASAQAGAIQAEAREGDDQATGGSFC
CDMQEISASYTLQSIFDVAFGLPLGEIEGTEDFAGHMGFVNEHCAQRLFVKQYYKMFRWFMPSERELRRHTRAIHAVA
ESVLLRRLQESEEKVSSRSDLLSLFICKARELAFDGKNGHDGPEVASLLGPETLRSIILTFVFAGRDTTAECLTYAFYAIARHP
RVQKRIVEELGSAKPNDDTPFTFDEVKHLRYLEAVVYETVRLYPALPYNVKNAVKDDYLPDGTFVPAGVDIVYSPWYMG
RNGPLWGDDPLEFRPERWLEMAKRPSAYEFPAFQAGPRVCLGMKMAVLEAKLFLATTLLRFDVAIAPGEKHERGYML
KSGLFMGGGLPLQMTPRPRNAPPA*

>CYP5017A10 P.vexan

MLSTALRVAVATSLVLILLRAASALYRKLRIARGLAPLSGPKGVFLLGNIPAFIRNRDRIYDFLEELLKTHGGRMK
MPWHLFFDGAIYGCTLSDFLLDMQITDPADVKHILSTNFANYVKPQGFIDAFKEVFEHSFFAMNHHGDAPDGGNKWR
LQRKVAAAVFTTKNFRDFSERVFAKYAMDAVRTIESQGNQCDMQELCAKYTLQSIFDVAFGLPLHDVADDDEFAEHM
GFVNEHCASRLFVKQHYKLLGWAMPSEYALKRHTDAIRAVADRILTRRLAEPASQLAARSDILSLFIQRARDLDAASASLL
DIPTLRSIILTFIFAGRDTTAECITYTFYAIARHPDVQQKIVEELEKAAEGDDDTSGMTYETVKRFKYLEAVVLEAVRLYPAL
PFNVKHAVCDDYLPDGTFVPAGTDVVYSPWFMGRNNPIWGPDPLEFRPERWLEMPSRPSAFEFPAFQAGPRVCLG
MNMAIIEAKLFVAVVLRRFHVRILPGEQQERGYTLKSGLFMSGGLPLQMTPRDEAALFS*

>CYP5017A11 Phytophthora capsici

TLRSIFNVAFGISLSKVAVADEFACHMDFINKHCAQRLFVKQYYKILRWFMPSEQELRWRTREVRSVADAVLL
QRLGESQEEINARSDLLSLFIRKARETEACSLLGAKTLRSIILTFVFAGRDTTAECLTYSFYAIARHPRVQERIVEELESLRPKT
GSNPTPFTFDAVKSMRYLEAVVYEVIRLYPALPYNVKNAVEDDYLPDGTFVPAGVDVVYSPWYMGRNGALWGDDPLE
FRPERWLEMTKRPSAYEFPAFQAGPRICLGMNMAVLEAKLFLATTLHRFHVAIAPGEKVERNYVLKSGLFMENGLPLQL
TSRLQYYCNMPYQVLPGLVVISVAFSLMGVGFGAVNKWQARNNMQSKLELLTDWDRMMDERDRKLAARAAAFDA
KQ*

>CYP5017A12 P.parasitica

INRAMKMPWHVFFDGAIYITDPMDVQHILATNLNNYVKPQALLDAFQEIFGDSFIAMNHHPQAPDGGAG
WRLQRKVAAKVFTTANFRVFTEQVFARHAEETLALAQAESKGESFCCDLQTLSTRFTLHSIFDVAFGLPLSEVKNADSFA
AHLDLANEHCAQRLFVKQHYKLFRWIMPSERKLRQCVRGIYAVSDAILLHRLGESEDKIQARSDLLSLFICKARELAATSQ
EERDESETSCLLGPKILRSIIVTFIVAGRDTTAACITYCFYAIARHPQVQKRIVEELESLKRSTDSSSTPFTFENVKNMRYLDA
VVHEALRLYPPVPYNLKCAVKDDYLPDDTFVPAGVEIVYSPWYMGRNSAIWGDDPLEFRPERWLELTKHPSAYEFPAF
QAGPRLCLGMNMAVLEAKFFLATTLHRYHITIAPGETQERGYVLKSTLVMKGGLPLQMTPRARHSLSA*

>CYP5017A13 P.iwayamai

LVLPPVSVTLVAQVLAAALTIALTSVLVAIGSSIRRKVRAAKLLAPVGGPKGKFLLGLLPEMTRNLPRLFDFQAEL
LTKYGGRLVVPWNLLSPNTLFIAPRSDLLSLFIKRARELEAEGANALDLPTLRSLVTSSVFAGRDTTSSSILYSFYNLAQHPE
QQDKIFAELKTVDTANLTYDDVKKLKYLDAFVWETLRLYPTTPMNLKEAAEDDHLPDGTFVPAGTDVMYSSYYMGRSN

AALWGEDQLVFRPERWLEMKTRPTAYEFPMFQAGPRICPGMNMALLETKMFIAILLQKFHVKMQEGEQVKDRPYVF
GASLVMKGGLPLQLTPRFAPSY*

>CYP5017A14 P.irregulare

PSEYQLKRDVKKLQQVADSVLFPRLAESDEEISKRNDILSLFIQKARELEGGEGKAVLDVNTLRDVFMNFVIAG
WHTTASFITWLSYVIAQYPEVQQKLYEELTKLNKTALTYDDMKQLKYLDAVAMETMRLYPTLPFNMKIAAKDDYLPDG
TFIPAGTEITYSPWYMGRHNPMWGDDPLVFRPERWLELKTRPSAFEFPVFQAGPRICPGMNLSLLETKMFVAVMLQR
FHIKIQDGEQVENRSYKMGTNLVMDNGLPLQMTPRKAAAPAS*

>CYP5017A15 P.ultimum

MALASSDGPKGAFLLGNIPNFIKHKDRIYDFLYSCLTLISDPEHVKHVLATNFDNYVKPQGFLDAFHEVFAHSFF
AMNHAHNADNGAKWRLQRKVAAKVFTTSNFRHYTEHVFAKYTQEMVKTIDVCMEKGQSCDMQALCAQYTLRSIFDI
AFGIPLEDVVDPFEFANRMDFVNAHCASRLFVKQYYKLLKWVMPSEHRLKRETDAIRGIADAILARRLDKPDDDLVARS
DILSLFIQKARELEKGESDPSLLCVDTLRSIILTFIFAGRDTTAECITYTFYGIARHPHVQRKIVEELQTVLNGTGDASLSYSDV
RQLKYLDAVVYEAIRLYPALPYNRWLEMKTRPSAFEFPAFQAGPRVCIGMNMALLEAKMCVAGVLRHIDVRIPASESQ
ER*

>CYP5017A2 P. sojae

MATSLHHKLRIARGLAPLSGPPGVWLLGNMPAYIKNRDRMYHFLEDLLKQHGGRMKMPWHLFFDGAIYIT
DPKDVQHILSTNFDNYVKPQGFLDAFQEIFENSFFAVNHHAQAPDGGAGWRLQRKVAAKVFTTANFRIFTEQVFARH
AEETLLAAQAEATESRAREDPDERFCCDMQEISAKYTLNSIFDVAFGLPLSEIEGTENFAEHMGFVNTHCAQRLFVKQYY
KLLRWVMPSERELRRRTREIRAVADTVLLRRLQESQEKINARSDLLSLFIRKARELAFESTKDQKVDAASLLGPKTLRSILLT
FVFAGRDTTAECLTYSFYAIARHPRVQKQIVEELESTKENTGSTHATFTFDQVKEMKYLEAVVYEAVRLYPALPFNVKNA
VKDDYLPDGTFVPAGVDVVYSPWFMGRNGELWGNDPLEFRPERWLEMPKRPSAYEFPAFQAGPRVCLGMGMAVL
EAKLFLATTLSRFHVAIAPGEKQERGYVLKSGLFMDGGLPLQMTPRPQSAASA*

>CYP5017A3 P. sojae

MQLPPLPLSPWSSLEPPAASAVAKILLTAAVASLLAKIGTMFHRKVRIARGLEPLSGSPGVWLLGNMPAFIKNH
NRIYEFLEDLLKQYGGRMKMPWHLFFDGAIYITDPKDVQHILSTNFNNYVKPQGFLDAFQEAFDNSLFILNHNAEAPDG
GAGWRLQRKVTLKVFTTANFRIYTEKIFARHAEETMVNAQAEAVKVRDSQSSNESFCCDMQAVSARYTFNSIFDVAFG
LPLSEIEGADEFAEQINFVNEHCAQRLFVKQYYKMLSWVMPSERELRRCTRGIRAVADNILLRRLKEPGEKISARSDLLSL
FIRKARELAAEGTKEQGADAAALLGPNTLRSIILTFVFGGRDTIAECITYSFYAIAKHPQVQQRIVEELESIKTSGGLKVTAFT
FDEVNSMKYLDAVVYEALRLYPAVPYNVKSAVKDDYLPDGTFVPAGVDVVYCPWYMGRNSALWGDNPLEFRPERWL
EMSKRPSAYEFPVFQAGPRICPGMNMAILETKFFLATTLSRFHVAIAPGEKQERGYVLKMALFMDGGLPLQMTPRAQF
TS*

>CYP5017A4 Phytophthora capsici

MQSPLLFLGSCAVAWLLAGISTALYRKLRIARGLAPIPGPPGIFLLGCMPAYAKNIHRIYYFLEDLLKENGGRMK
MPWHIFFDGAIYITDPKDVQHVLSTNFNNYVKPRGFLDAFHELFGNSLVGTNHHPQAPDGGASWRLQRKVSAKVFTT
ANFRIFTEQIFARHAEEVLTATRNKAMQSTGNQPADGSFCCDMQTTSANFTLQSIFDVAFGLPLSEVIGGEDFNEQMD
FINEHCAQRMFTKQHYKLFRWIMPSERELQQCVRSIHAVADKILLRRLEEPEEQVKARSDLLSLFICKARELASNDQEIAH
GDLAASSLLGLETLRSIILTFIVAGRDTTAACVTYSLYAIARDPRVQKRIVEELESSKPGTGSSLAPFTFEDVKNMKYLDAAV

YEALRLYPSVPYNVKVAVDDDHFPDGTFVPAGAEIVYSPWCMGRSEVFWGEDYLEFRPERWLEMDKRPSAYEFPAFQAGPRICLGMNMAVLEAKLFVATTLRRFHVAIAPGEKHERGYVLKSALFMEGGLPLQLTPRSKSPLSA*

>CYP5017A5 P.parasitica

INRAMKLLLTVTAAILLVKISTALHRKLRIARGLAPLSGPSGVWLLGNMPAYIKNRRRIYHFLEELLKQHGGRMKMPWHLFFDGAIYITDPKDVQHILSTNVSNYVKPQGFLDAFQEVFANSFFAMNHHAQAPDGGAGWRLQRKVAAKVFTTANFRVFTEKIFARHAEETLNAAQTESFKAAARDGNQSADGSFCCDIQEISARYTLQSIFDVAFGLPLSDVAIAEDFAKHMSFVNEHCAERLFVKQYYKVLRWIMPSERELRWSTREIRAVADAVLLRRLGESEAEIKARSDLLSLFICKSRELAANDQCGTDASSLLGPETLRSIILTFVFAGRDTTAECLTYSFYAIARHPKVQKRIAEELETAISSTSTPFTFEDVKNMKYLEAVVYEAVRLYPALPYNVKSAVKDDYLPDGTFVPAGVDIVYSPWFMGRNGAFWGDNPLEFRPERWLEMTKRPSAFEFPAFQAGPRICLGMNMAVLEAKLFLATTLRRFHIAIAPGEKHDRGYVLKSGLFMDGGLPLQLTPRPQRAASA*

>CYP5017A6 P.infestans

MSTALQRKLRIARGLAPISGPSGVWLLGNMPTYIENRRRIYHFLEDLLKQFGGRMKMPWHLFFDGAIYITDPKDVEHILSTNVSNYVKPQGFLDAFQEVFANSFFAMNHHPQAPDGGAGWRLQRKVAAKVFTTANFRVFTEKVFARHAEETLATAQAESIRDAVQYGNQSNDGSFCCDLQEISARYTLQSIFDAAFGLPLSDVADVEEFAKHMDFVNEHCAQRLFVKQYYKVLRWIMPSERELRWRTREIRAVADAVLLRRLEESEEEINARSDLLSLFIRKSHASSLLGPETLRSIILTFVFAGRDTTAECITYSFYAIAKYSRVQDRIAKELKTAKRSTSTPFTFEDVKNMKYLEAVVYEAVRLFPALPYNVKNAVKDDYLPDGTFVPAGVDIVYSPWYMGRNGALWGDDPLEFRPERWLEMSKRPSAYEFPAFQAGPRICLGMNMAMLEAKLFLATTVRRFQVAIAPGEKQERGYVLKSGLFMDGGLPLQLTPRQSSRLQPAHSA*

>CYP5017A7 P.aphanidermatum

MRAVTWSLYEVVLALGCVAATVVLSVPLRALRRKLRIARGLAPIPGPKGMFLLGNMPLFIKNKNRMYHLLEEMLKQYGGRMKMPWHIFFDGAVYITDPRDVEHVLATNFENYIKPQGFIDAFQEVFEHSFFAMNHAHTPDNGDKWRLQRKVASRVFTTKNFKVFSEQVFTKYAQQIAVELQEAGGRCDMQELSAQYTLRAIFDIAFGTALDNFIDPEVFAGRMNFVNEHCASRLFVKQYYKWFRWIMPSEATLRRYTSEIRAIADKILLARLQEPVEELDQRFDILSLFIKKARELDGESASLLDPLTLRSIILTFIFAGRDTTAGCITYMFYALARHPEVQHKIIAELETVNAANAMDALSYDDVKQLKYLDAVAMETFPAFQAGPRVCVGMNMALLEAKVFTAVLLRHFHVQIADGEARERGYVLKSGLFMAGGLPLQMTPRARD

>CYP5017A8 P.irregulare

PAFSWSQITRLLFCIVLSVLIAAPIQALRRKLRIARGLAPLQGPKGVFLLGNIPNFVRNKNRIYHFLEERLQEYGGIMKMPWHLFFDGAIYITDPEHVKHVLSTNFENYVKPQGFIDAFQEVFEHSFFAMNHHAHTPDKGEKWRLQRKVAAKVFTATNFKTFTEKVFDKYAQSVIAVIEAQDSQCDMQELCAQYTLQSIFDIAFGIPLQEVVDPQAFATSMDFVNEHCASRLFVKQYYKLFAWCMPSEYRLKRETDFIRGIADKILLRRLQESEQEIAERFDILSLFIKKARELDEDSASLLCPDTLRSIILTFIFAGRDTTAECITYTFYGIARHPEVQQKIVDELLSCDKDSSLNYDDMKSLRYLEAVVYEAVRLYPALPYNVKMAVNDDQLPDGTFVPAGTDVVYSPWYMGRNNPIWGSDPLAFRPERWLEMKTRPSAFEFPAFQAGPRVCIGMNMAVLEAKMFIAVMLRHFHVKIPDHEKQERGYLLKSGLFMDGGLPLEMTVRDARAFS*

>CYP5017A9 P.iwayamai

LPLFEMRVALSWTLIARALLAAALLWLIYIPIRALRRKLRIARGLAPLKGPKGAFLLGNIPNFIKHKNRIYHFLEENLRECGGIMKMPWHLFFDGAIYITDPEHVKHVLATNFDNYIKPQGFIDAFQEVFEHSFFAMNHHAQTPDKGAKWRLQRKVAAKVFTASNFRTYTEHVFDKYAQAMARMTQAQEQDDKASSRVDMQELCAQYTLQSIFDIAFGIPLQEIVDPETFAHSMNYVNKHCASRLFVKQYYKLLKWCMPSEYRLKRETDFIRGIADKILLRRLAESDLEIAERSDILSLFIKKARELDEDSASLLC

PETLRSIILTFIFAGRDTTAECITYTFYGIARHPEVQQKIIDELRVSHGTAGDAALSYDDVKSLKYLEAVVYEAVRLYPALPYN
VKMAVADDHLPDGTFVPAGTDVXXXXXXXXXXXXXXXXXXXXXXXXXXXXXXXXXXXXXXXXXXXXXXXXXXXXXXXXXXX
MFIAVMLRHFHIQIPADEAQERGYLLKSGLFMAGGLPLEMTPR

>CYP5017B1 P.ultimum

PLDGPKGVILLGILPKYIANKTRIYDFLEDLLKQFHGRMKLPWHLFFDGAIYVSHPEDVKYILSTNFDNYIKPPGFI
AAFDELFAGSLFAMNHAHCADGGAKWKLQRKVAAKVFTTTNFKLFVEQIFDKYAREMATKIDQSEDGKCDMQDIAR
QYTLHSIFDITLGVPLTEVADVHAFGESIDFVNEQSAARMFIKQHYRWLGWCMPSEYRLMREVKVMTAIADKILERRLK
ETXXXXRKVTVKILAQRVAEPEHELAKRFDILSMFVKKMRELENDEASLLDLDTLQRILHTFISAGRDTTSSSITHTIYALCR
HPEVQRKVADEISELTNNDSNDNHGGSVLSYDGIKNLRYLDAVVHEALRMYPPVPFNIKRAAEDDHLPDGTFVPAGCD
LAYSPWYMGRNSAMWMPDPLVFRPERWLEMATRPTAFEFPVFQAGPRICIGMNMAVLQAKMFIATMFQRFDGIKI
QDGQQQERGYVLRSTLTMDGGLPLQMVPHAKPSTIT*

>CYP5017B2 P.iwayamai

MEFSSAQIATTAACIALTGLLAHVARLLERKLRIARGLAPLSGPKGVLLLGLLPQFAENKDRVYDFLEGLLKQHG
GRMKMPWHIFMDGAIYQVCYTFFKSIPQVTDPEDVKHVLQANFDNYIRPPGFIAVFRELFADSLFALNHAHCPDNGAK
WRLQRKIAAKVFTTNNFRLFMEQAFGKYAEEVVHLVGHEQGGKCDMQRVAKLFTLQSIFDITMGVPLAQIADVDAFG
ESIEFANEQGAARLFVKQHYAWFGWCMPSEQRLKREVAAIHKVTGAIMKQRMLESDEQLAQRSDILSLFLKKQRELSP
EEASLFDMDALQKLLHTFLFGGRDTTASCITYAFYALCRTPKAHCKLVDEVKAYATKTSSSSLSYEGIKSLKYLDAVVHETL
RLYPPAPFVIKRAAENDHLPDGTFVPAGCDVAFSAWYLGRNSSMWAPDPLVFRPERWLEMETRPTPYEFPVFQGGPR
ICPGMNMAVLEAKIFIAVMLRSFESVEIQDGEQQERGYVLKSTLTMGDGLPLQLIPRLASA*

>CYP5017C1 P.iwayamai

MKLPWNLLYENSLYIASAEDVKHIMSTNFDNYIKPPAMVDAFRDLYEDSLFFVNHAHTRDEGEKWRVLRKLT
AKVFATSNFKLFFEQIFHKYAMEMVAEIEIQSGVVNLLDVASEFTLRSIFDIGCGVPLREIEDPMSFAKALDGVNQQCML
RLMLKRHYRYFWWCMPSEYEMKRNVKIVGDIVDAIVSQRLSESHEEITKRSDSLSLFIVKAHELDESEKSWMVDRTTM
RSVPMTFLIAGRDTTSSTISYAFYEIVRHSEVQRKIIEELQQLDKAVFDYDDMRKLTYIDAVVNETLRLHPAVPFDLKVAVE
DDHLPDGTFVPAGTEVNFCPWYMGRNNAKLWGDDPLVFRPERWLEMKTRPSAYENPVFQAGPRICIGMSMALLEA
KMFIAVLLNHFHVELQEGEKSERGYILSASLTLEGGLPVKMTSREARSF*

>CYP5017C2 P.irregulare

HHFPLVSVLSLDWGQVADTLAVLVIMWVFLSFAGALQRKLRIAKGLVAVPGPKGTLLLGILPQMAANIHRFYH
FQEDLMKQYGGRMKLPWNIFFDSALYIASPEDVQHVLSTNFGNYVKPQAMLDAFKELYEDSLFFVNHTHCRDNGER
WTMLRKVSTKVFTTSNFRVFSRQIFYKYAASDTIGTIETQGGKCNMLHVARQFTLRSIFDIGCGIPLANLEDPDTFANAL
DGVNEHGAMRLLLKRHYKLFWWCMPSEYKLKRDVSAVASVAEAIVRNRLAEPSEVIAKRSDILSLFVMKARELEDAPS
WMLEFTTLRSVLMTFVLAGRDTTSSAITYAFYEIVRHPEVQEKIVKELHQLGTSDLNYDDMKRLPYIDAVLNETLRLNPP
VPFDIKVAAEDDHLPDGTFVPAGTEIFFSPWYMGRNNSIWGNDPLMFRPERWSEMKTRPSAYENPVFQAGPRICIGM
SMALLEAKMFIAVMLRHFHVEFQDGEGDVNRERPYALNASLMLDCGLPLKMTPREARPC*

>CYP5017D1 P.irregulare

MNVDDVKKLKYLDAFVWETMRLYPAVPANLKQAAEDDVLPDGTLIPAGTEIMYSPYYMGRNNTKLWGEDQ
LVFRPERWLSMPRRPTAYEFPVFQGGPRICPGMNMALLETKIFVAILLQKFHVKIQDGEQVKDRPSATCLIVATTMVFP
PVSQLPFTQVATAAATILLTRVVILIASSIWRKMRAAKVLAPVPGPKGHFLVGMIPELIKNLHHLYDFQLDLMTKYGGRT

KSPWSIFSNHVLYLADVADVEHVLSTNVNNWVRSDRMIAALGELFGKSFLALNHAHTADGGAMWRLQRKVVNRVFT
TNNLKALTEGIFSKYAVRIADLIVAEEGKCDMRQVSSQYTLQTAFDIICGVPLESFDDELGVKLIESMDFVFSSISLRGIAKP
YLKYLWWCMPSEYRFRRESKVVTDLVDSILKRRLEESDDEITKRSDLLSLFIKKARELEAEYGVILDIVTLR*

>CYP5017D10 P.iwayamai

PVLPPLSLSLAAQVLATTLTVALTGVLVAIGSSIRRKTRAQMLLAPVPGPKGAFLLGLFPELAKNLHRIYDFQAEL
ITRYGGRMKTPWTIFNDNLIYLSDPKDVEHVLSTNMQNWIKSDRFIAAVGDIFGKTLLGINHAHTADSGAMFRIQRKVI
TRVFTANNFREFTEGVFHKYALRIVDVINSQGGKCEMHTIASQFTLQTIFDIGLGVSLEEFDKDLGVKFIDSMDYAFSSISA
RLIFKPYFRYLWWCMPSEYRLKRESKVMLDLIDGILHERLAESEEKFAPRSDILSLCIKKAREYVTEGAVMLDIETLRASVT
GVLFAGRDTTSSTILYAFYNLAQYPEQQDKVLDELKSVDVANLSYEDVKKLKYLDAFVWETLRLHPTAPLNTKQAAEDD
VLPDGTFIPAKTEVIISSYYMGRNNAKLWGEDQLVFKPERWLEMKTRPTAYEFPVFQGGPRICPGMNMALLEAKIFIAIL
VEKFHVKMQDGEQIKDRPYVLAPAMMMKGGLPLQLTPRDAAAAPAS*

>CYP5017D11 P.irregulare

MALTGVLMAIASSIRRKMRVAKLLAPVPGPKGIFLLGLLPELNQNIHCCYDFLTELAHKYDGRVHIPWDIFTGN
MLFYVLSTNLKNWVKSDHFRRSICEVFGIVLFGLNHTHTVDGGAMFRLQRKVTSKIFTTSNFEGIFQRYALRLMDIINAQ
NGKVSMYAIANQDTLQVTFDIVVKVPPESIDKELGLKFTKSMGVSEYHYQHEAKVLLDTFDGIARQRLEEPEENVATRSD
VLSLFVKKARELEAEGDRTLDIPALRSITVGTIFGGRDTTSAAITYSLYNLAQYPEQQNKTLEELKRPTPKLRHLDAFVWET
RRLYPVVPSVMKVAVEDDVFPDGTFIPAETEITASAYYMDRHNAKLWEEDQLVFRPQRWLDMKTQPTAYNHPVLLGG
PRMCPAMDLALLEVKIFIAVLLQTFHMTI*

>CYP5017D12 P.irregulare

MTTVAQVAAATAAVALTSVILSLATSIRRKIRAAKLLKPVPGPKGRFLVGLMPELIQNMHRFYDFQMDLMVKY
GGRTKIPWNIFANHFLYLADPKDVEYVLSANVNNWIKSDRFTAAMGELFGRSFLGINHAHTPDDGALWRLQRKVVTR
VFTTSNFNLLTEGIFHKSALCLVELIVSHNGKCDMRKTSSQYTMQTAFDIVCGVPLESFDKELGLQFIESMDFVFSSISQR
GIKKPYYTFLWWCMPSEYRLKRETKVLTNLVDSILEKRLAESGGEIAKRYDVLSLFIKKARELKSGEGESILDIPTLRSIVATLI
FGARDTTASTICYAFYNLA*

>CYP5017D13 P.irregulare

MRYLRNSLRAAKLLAVPGPKGHVLFGLVPELIKNAHRVWDFQSELVIKYGGRARIPGPIFGNDVLYLGDPKDV
EHVLSKNVDNWIKSDRFIASVGEVFEKSLLGINHAHTADGGALFRLQRKVVTRVFTTTNFKALTEGIFHKYTTHLADRIM
AEDGKCDMHKISSQFTMQTTFDIVCGVPLETIDKDLGLQFIESMDFVFSSISWRGLTKPYFTFLWWCMPDEYRLKREIK
VRLEESEEEIAKRYDMLSLFIKRARELEGEEGAVVLNITTLRSIIASLIFAGRDTASSSIVYAFYALA*

>CYP5017D14 P.iwayamai

LVLPPLSLAQVLTAAVTLHPAQGARRKVRGAKLLGPVPGPKGKFLLGLIPELTGNLHRIYDFQAELMNKFGGRT
LVPWNIFGDNMIYLSDPKDVEYVLSTNMQNWIKSDHFIKSMGDMFGKSFLGVNHAHTEDDGAMWRLQRKITSRVFT
TSNFRLFTEQIFHKYTTAILSHIKAQDGKVDMHTLASQYTLQSTYDISCGVPLESFDKGLGLKSVKSMDFVFSMITMTELF
NRILEKRLGESAEEIAPRSDILSLFIKKARELEAEGAAVLDVPTLRAIVAMLIFAGRDTTSSAILYAFYNLAQYPEHVDTSALT
LEDVKKLKYLDAFVWETMRLHPTLEFRPERWLEMKTRPTAYEFPVFQGGPRICPGMNMALLEAKIFIAILLKKYH

>CYP5017D2 P.irregulare

MPPVSVVIQLLAAAATIALTSVIVSIANQVRRKMRSAKLLVSVPGPEGIFLLGFLPEFAKNIDRIYDFLNELMTTY
GGRMKVPWTIFNDNTLYLTDPADVEYILATNMNNWVKSDHFIASVGEVFGRSLLGTNHAHTADGGALYRIERKFVSKV

FTTSNFKAFTEGIFHKYAVRIADLIDAQNGKIDMHIISSQYTLQTIFDITCGVPLESIDKELGLKFINSMDYVFGSISQRLAVK
PYFRYFWWCMPSEFRLKRETKVVLELASGVLKQRLDEDEAVVVKRSDVLSLLVKRARELDMEEGAVPDIPTLCSLITATIF
GGRDTTSSAILYSFYNLAQYPEEQDKILAELKTVDSTALTYEDVKKLKYLDAFVWETMRLYPTAPMNLKQAAEDDVLPD
GTFIPAGTEAYYSAWYMGRNNTKLWGEDQLVFRPERWLSMPRRPTAYEFPVFQGGPRICPGMNMALLETKIFVAILL
QKFHVKIQDGEQVKDRPYVLGPTLVMKDGLPLQLSPRQAAAN*

>CYP5017D3 P.irregulare

MTSNFKVFTEVIFHKCAMRTVGLIHAQNGKVEIHALSSQFTLQTIFEVVCGVDLQSIDEELGLKFIESMDFHPIR
TIFKPYFKYFWWCMPSEYRLKRESRKMLNLVDGILKERLRESEEQIAKRLDILSMLIKKARQLGKEGKTILDIPTLRSVVTAT
VFAGRDTSSSVILYTFYALAQYPKQQTKILDELKPLSKTALTYDDVKNLTYLDAFVWETIRLYPTTPSTVKQAAEDDVFPD
GTFVPAKTEILVSSYYMERYNAKLWGDDLLVFRPERWLEMKIRPTPYEFPLFQGGSRMCPGRNMALLEAKLLVAIMLPK
FHVAAQEGYQVKD*

>CYP5017D4 P.iwayamai

LVLPSLSATLVAQVFAAALTVALTGALVSFGNSIRFKTRAAKQLKPIPGPKGRFLLGLVPELTKNLYRIYDFQADL
LVKYGGRVHMPWNIFGANMVYISDVKDVEHILVKNVNNYIKSEHFVLSVGEVFEKSLLAINHANTPDGGAMYRLQRKV
VTRVFTTANFKVHTEGIFHKYAMRLVDLINAQNGKCNMHQVASQYTMQTIFDIGCGVPLESFDKDLGLQFVKSMDFV
FEMFLLRLVTQPYFKYFSWFMPSEYRYKREAKVMTDLIDSVLAKRLVESEEALAGRNDILSLFIKKAREESDGGLIDFETLR
SLVTAIVFGGRDTTTSTITYAFYNLAQHPEQQDKIFEELKTVDVSSMTFDDVKKLRYLDAFIYETLRLYPTTPSNFKHAAND
DYLPDGTFIPAGTDVMYSSYYMGRNNAKLWGDDQLEFRPERWLEMKTRPTACEFPIFQAGPRICPGMNMALLEAKIF
TAILVKEFHVKMQDGEQVKDRPYLLGATLVMEGGLPLQLTPRVAASN*

>CYP5017D5 P.iwayamai

MLVLPPVSANLVAQVVAAALTVALTGVFVSIGSSIHRKTRAAKQLEPIPGPSGRFLLGFMPELAKNLQRIYDFQ
SELLSQYDGRVKLPWNIFGANMVIVSDPKDVEHMLTTNMDNYIKSDHYIQSLGEIFGKTLIGTNHSHVADGGAMYRLQ
RKDGKVDMYLIASQFTLQTIFDIDCGAPLESFDKDLGLQFVKSMDFVFEMFLLRLVTQPYFKYFSWFMPSEYRFHRESKF
MMSLVDSILERRLGESEEEIAPRFDILSLFVKKARELKAEGASILDVPTLRAILTGTLFGGRDTTASTLHYCFYNLAQYPEQQ
DKILEELKSVDVNALTFEDVKTLKYLDAFVWETLRLYPTVPTNFKQAAEDNYLPDGTFIPAGTEITSGGEDQLVFRPERWL
EMKTQPTAYEFPVFQGGPCICSGMNMALLETKIFIAMLLKKFHVKMQDGEQIKGRPYTFGATLVMEDGLPLQLTPRV*

>CYP5017D6 P.iwayamai

LVLPPVSMTLVAQVVTAVLTIALTGVLVAIGSSIRSKMRGAKLLAPVAGPNGRFLLGLLPEMTENLHRFFDYEM
DLFKEYGERVVMPWNLLNPNMLYVSDPKDVEHMLSGNLKNYIKSDHMIACVGERKITTRIFTTSNFKAFTEGIFQQYAL
VLVDKIAANDGKCEMHTVASQYTLQTIFDIVCGVSLASIDEDLGLQFVESMDIVFDSIMVRAVFKPYFKYLWWCMPSEY
RIRRESKVMLDLVDGIIRKRLLETEAQIVPRLDLLSLFVKRARELEAEGANALDLPTLRSVVTSSVFAGRDTTSSSILYXXXXX
XQYPEQQDKILDELKSVDATKLTFEDVKKLKYLDAFVWETLRLYPTAPGNSKQAAEDDYLPDGTFVPAGTEVLYSSYYM
GRSNAKLWGDDLLVFRPERWLEMKTRPTAYEFPLFQGGPRICPGMNMALLQTKMFIAILLQKFHVQIQDGEQVQDRP
YVFGASLVMKDGLPLQLTPRVTPSN*

>CYP5017D7 P.iwayamai

MLVLPPMSLALVAQVLAAVTTIALTGVLVTIGNSIRRKMRAQKLLVALPGPKGRFLVGFVPVMVKNLHRVYDF
QVELHARYGERFAVPWSLFAGNLIYTSDPKDIEYMFTTNVDNWIKSDHFIASVGEVFGKSLLATNHAYTKDSGAMYRV
QRKLMSRVFTTTNFKDFTEQVFHQKALRMVGIINTQDGKVDMHEIASQYTLQTIFDIGCGVPLEEIDEDLGVQFIDAMD

FVFASITSHLMFKPYFRYLWWCMPSEYRLKRESKAMLDLADGILQKRLLETDDEIAKRFDMLSLFIKEVRELKAEGQEML
DSATIRSMFAAIIFAGRDTTTSTILLLFYNLTQYPEQQDKILEELKSVDTAKLTFEDVKKLKYLDAFVWETIRLNPGYI*

>CYP5017D8 P.iwayamai
LILSPVTLSAQVLTVALAVVLASAFVAIGGSIRRTLRADKLLTPVPVPDGAFLLGCIPDVLRNLHRIYDYQAELLLK
YGGRVRVPWSVFGGNVLYTSNPKDVEHMFSTNMDNYIKSSHFVASVGEIFDKTLFALNHAHTADGGAMYKLQRKTIT
RVFTANNFREFTEGVFHKYALRTIDVINTQGGKCEMHTIASQFTLQTIFDIGLGVSLESIDEDLGLKFVDSMDCVFLGIAA
RLMYKPYFKYFGWCMPSEYKLKRESKVLLDLIDGIIKDRLAESDEQFQARSDILTMCIKKGRELEAEGNTMVDIPTLRSSIT
GMLFGGRDTISSVILYAFYNLAQYPEQQDKLLEELETVDIANLTYDDTLRLNPTAPLNTRQVAEDDVLPDGTFVPAGTDII
ISTYFMGRNNAALWGEDLLVFRPERWFEMKKRPSAYEFPVFQAGPRICPGMNMALLEAKVFIAILLSEFHVKIQDGEQ
VKDRPYVLALAMMMKDGLPLQLTPRAAPFAS*

>CYP5017D9 P.iwayamai
LVLPPISLSLAAQVLATALTIALTGVFVAIGNSLLAPVPGPKGTFLLGLIPELKKNIHRIYAFQAELMVQYGGRM
MVPWNLIGDNTIYLSDAKDVEHVLSGNMNNYIKSDHLIQSVGHLFAKGLLGTNHAHTADGGAMYKLLRKLVSRVFTTS
NFKKFTEGIFHKYALRLVDVINTQDGKCEIHTIASQFTLQTVFDIGCGVSLESIDKELGFQFIESMNYAFGIITERILLKPYFKY
LWWCMPSEYRYKREAKVMSDLIDGILQERLNESEEIIAPRADILSLAIKKARELQSEGEQMLDISTLRSLVTSIIFAGRDTTS
STILYCFYNLAQFPDEQEKIFEELKSVNTTTLTYEDVKTLKYLDAFVWETMRLYPTSPANLKQAAEDDYFPDGTFVPAGTE
VMYTAYYMGRNNAKLWGDDQLTFRPERWLEMKTRPTAYEFPVFQAGPRICPGMNMALLEAKIFIAILVEKFHVTIQD
GEKLEGREYIAAPTLMMVDGLPLQLTPRVASAC*

>CYP5017E1 P.irregulare
MRTEDGGAIWWLQRKVVSKVLSAANFKVFSDQVFYKHAVQIAHQIKAQNGKCDISTLTSQYTLQSIFDVSCD
VPFAQVDDQLGLGFIASLSFVVQHISAQIFTKPYFKYFWWCMPDEYRYKREAQVLRKIANETLDRRLLENEDEIAPRSGI
MSLFIKKARELSADEDFKSSSSPVLDVAALRAIFLSLIWGSWDTTSSAITYTLYALALSPKVQEKLFEKISMGSGLHETGGG
GETYDSLKKFAYLDAA

>CYP5017E2 P.irregulare
MLVFIPDSDVSYALIAKSLLTILLTAFAARVVSALQHKLRIAEGMAPIPGPKGWPLVGVLPLFIKHAYRFNEFHM
CRHDEMMVKYGGRMKYPWSIFSDGMISIASPEDVKHVLATNMDNYIRSPRFFASMEELFGKGLLLLDHAHTKDNGA
MWHLQRKVASRVFTTNNFKLFSEQIFRKHALHMVQQVEEQRGKVDMSKLASQYTLQSIFDVSCGVPLAEVDDQLGLS
FVDSLSFVAHHMSTRLLSKPYFLYFWWCMPSEYRLRREAQVLTNIANMILDRRLQESAEEIEPRSDIMSLFIKKARELSLE
EGSSSTSPVLDIATLRSIFLTFNWAGWDTTSSAVIYTFYALAQYPEVQEKLFREISSSGDSENLQGNDENSDGVTYTDLKK
FAYLDAV*

>CYP5017E3 P.irregulare
NNCIRVPRFVESMQELFGKGLMLFNHAYTKDNDAMWCLQRKVVAKVFTTNNFKLFSEYIFYKHAIQIADITN
DQRGMCDMSKLASQYALQSIFDVSCGESLVKVDGRYKQSFANSMDFVAQHISTRLVTKPYFKYFWQCMPEGYRLKRE
AGVLTKIVDSIVERRLCEIKDEIAPRSDIMSLFIKKACELSAEKNEEEAPLPPSSMLDLATLREALPEINGGSGRKLPQDDAY
GDGIMYESLKRFAYLDTVVSEALRLYMTVPITIKLAFEDDYLPDGTFIPAGAEVSFAPWYMAHHNPIWGSDPLVFRPER
WLEMKARPSAYEFPIFQAGPRICPGMNMALTETKLLVVVLLRQFHIKIQEGEQVENRGYILAPVLTMSGGLPLQVVSRI
AAVSS*

>CYP5017E4 P.iwayamai

FIVSYELFAKTVLVILLSSIGARLVAALRCKRRIAKGIVGIPGPKGWPLIGVLPTMIKQGHRYYDFHDEMMVKYG
GRIKYPGNLISDGTVCVASPEDVQHILSTNFKNYIRAPRFVAAFDELFRNGLLLLDHAHTRDNGAMWTLQRKVAAKVFT
TNNFKLFSEQIFNKYALQAKKSIQEQGGKCDLSLLASQYALQSIFDVGCGVALAQVDDQLGLSFIESLRYVVGHAATRLVT
KPYFKFFWWCMPCEYRLKREVHVLISVADKILTRRLSESEAEIAPRSDIMSLFIKKARELNDEGGENASSLVLDIATLRSIFF
TFNLAGSDTTSSALTHTFYALALYPQVQEKLFQEIARSTDLVDGDDVSYDTLKRLVYLDAVVSEAMRLYTTVPMTIKLAAE
DDYLPDGTFIPAGVEVQYTPWYMARHGPLWGDDPLVFRPERWLEMKTRPSAFDFPLFQAGPRVCPGMTMALTEVKL
LVVVLLREFHVNIQEGEQIENRGF

>CYP5017F1 P.ultimum

MWSSVSFVQVAAALAVVALTRVLVPFLRDESLMVQYGGRIKLPWDLFSNGSVYITSAEDVKHILTTNFNNYIK
AEPLLATFSDLFGKSFFGLNHAHSPDNGAMWKLQRQVASKVFTTNNFKLFSEQIFHKYALKMVEIVDLQDGKCNILEIA
SQYTLQAIFDVGCGVPLTEVDERLGLSFVESLSFVASNIADRFFVKPYFRTFWWCMPSEWRLKREAKVMTNIADKILAR
RLKESDEELATKFDIMSLFIKKARELDGNECSSVLDVETLRSIFLTIIAAGWDTTSSTITYVFYCLIQYPETQDKLLQELEELNK
SQLTYDDVKKLKYLDAVVSESMRLYPTVPIDRKEAAEDDYLPDGTFIPAGTEVLYSPWYMGRHNPIWGDDPKVFRPER
WLEMKTRPSVYEFPAFQAGPRICIGMNMALLEAKMFIAVMVRNFHVQIQEGEQLKNRGYTLTPTLTMKGGLPLQMT
QREQVARPY*

>CYP5017F2 P.ultimum

MESWTERSPTLAQIVRALIAIALSSVAVARALEPIPGPRGVPLLGVLPTLIKNSKRMLEFMEELIEQHGGRLKTP
WSIFSDGMVVVSSPADVEHVLSTNHNNYIRSERLLQAVGEVFGKSFLGLNHAHTPDNGAMLRLQRKVGVKVFTTTNF
RVFAEQIFYKHAQTMVKVVQEQGGKCEMHKLSSQYTLQAIFDISCGVSLQDVDPTLGLSFINAMDYVLSYITVRLTVKPY
YRYLWWCMPSEYKMRRNEKIMIDLAEGILTQRLQESDDVLANRSDIMSLYIKKARELAGEGDAVLDVSTLRSIFLSFLFA
GKDTSSSAITYTFYALTQYPNVQQKLFDEIQSIKSTNFTYEDIKSLRYLDAVVSETMRLYPTVPTSVKRAVEDDHLPDGTFI
PGGINVMINQWYMGRHNPVFGDDLLTFRPERWLEMKTRPSVYDFPVFLGGPRICIGMNMALLETKMFVAVMVRHF
KMQIQDGHQVAKRPYAIGSTLVMKDGLPLQMMPHDFAQSS*

>CYP5017F3 P.iwayamai

MFVLSLVSSSFAALAVSTLLAVALSCVFVPIGLALLDLLLKIVRHVLRFHEFMSGYVFLLLALIFPSDCLVSHQPSYT
DRDGELTSPEDAQHVLATNHGNDVRSLRVMGAVGHVFGKSFLGLNHAHTQDDYSSCSAKWTSRTANFRVFMDQVF
HKYAAKMTGIIKAKDGKRDMQEICTQYTLQAIFDIACCIPWTPSLGLSFMKAMDYVLSYVSERLATKPYYKYFWWCMP
SEYKMKRNEQVMINLAESILSDRLAESDEEIAPRSDIMSLVIKKARELQDGEGASILDVDTLQDDCLPGGTFIPAGTEIVYT
PWYMGRHNLIFGEDREVFRPKRWLEMKKTKPSAYDFPVFRGGPRICIGMSMAVIEVRLFVAVMVHEFHVAMQEGEQ
VQDRPYVLSLTLVMDGGLPLQLTARA*

>CYP5017F4 P.iwayamai

LVLPPPSASLAALLAVVLTYLVVPVVAALRRKLRISAGLKPVAGPKGWPLLGMLPDILSHAPRVHEFTESLMVQ
YGGRVKIPWSLFGDSSLWLASAEDVQHVLATNHDNYVRAQRFIGSVGRVFDKSFLGLNHAHTADGGTMIKLQRKVGI
KVFTTTNFRVFTDQIFHKYAAKMVELVKQKGGKCDMHEISSQYTLQAIFDIGCGIPLQDVDPSLGLSFIKAMDYVFSYVT
ERLTTKPYYKYFWWCMPSEYEMRRNEQVMIDLADSLLSKRLQESDDEMAPRADIISLFIKKARELQDGEGTSILDLETLR
SIFLSFVFAGKDTSASTITYTFYALAQYPDVQQKLFQELKQYSNKGSKTPLTFDDIKKLPYLDAVVNETMRLYPTLPMNFK
VAVEDDHLPDGTFIPAGTEMVYNPWYMGRHNPIFGEDREVFRPERWLEMKTRPSAYDFPVFQGGPRICIGMSMAVI
EVRLFVAVMVREFSVAIQEGEQLVDRPYVLSPTMTMEGGLPVQMTARTKLQSALFE*

>CYP5017F5 P.iwayamai

MQIRASIFSNAMVFLASPADVQHMLATNHDNYVRAQRFIDAMGPTFQKSFLGLNHAHTSDNGAMLKLQRK VVTKVFNTSNFKTFSEHVFPKYALEMVELIRSQDGTCNMHDVSTQYALLAIFDISCGVSLHDVGEIRGLGFIKAMDFVFA HLSDRLMTKPYYKLLWWCMPSEYEMKRSEQVVMELADDILGRRLEESDEEIAKRSDVMSLFIKKAREQSEDDEGSGVL DLETLRAIFHNFIFAGKDPTSSVITYALYALCLYPDVQNKLYDEMPASIKASTSALTYENTKGFEYLDAVVSETMRLYPALP ANFKVAVEDDYLSDGTFIPAGTELAYSPWYMGRHNPIFGDDREAFRPERWLEMKNAKPSAYDFPVFQAGPRICVGMN MALIEVKVFVAVMVREFHVAIQEGEQVEDRGYIRSPTLTMEGGLPLQMTPREAVA*

>CYP5017F6 P.ultimum

MMAMLLAAKLPLAEVITAVLLVALTAVVAAIAHDIRRKVKIGRGLQAIPGPKGWPLLGILPAFMTPSPRLHDIV LASAEDVQHVLATNYKNYIRAPRFIASVGKLFGKSFLGINHAHTADNGAMLRLQRKVGIKVFTTSNFRLFSEQIFHKYAEK MVSIIAEQGGKCDMNVISGQYTLQAIFDIACGISLQEVDPQLGRSFMKSMDYVFATISERLMVKPYFKYFWWCMPSEY RMKRNEQVMIALADNILGRPRELEDGEGVSILDVEMLRSVFLSFLFAGKDSSSSAITYTFYALAQYSQVQQLLYEELQKV PTSGFSYEDIKNLRYLDAVVSETMRLYPTIPSNFKVAVEDDYLPDGTFIPAGTEVTYTAWYMGRHNPIWGHSPLVFRPER WLEMKTRPSAYEFPVFQAGPRVCIGMNMALLEIKMFVAVMIRQFRVQIQPGELVHDRGYIICPTLTMEGGLPLQITRR EIAPGF*

>CYP5017F7 P.ultimum

MIAIVESQDGKCDMHEISSQYTLQSIFDIACGISLQDVDQKLGLSFIKAMDYVFSYISVRMLAKPYYKYLWWXX XXYKMKRNEQIMIDLAESILGPRLQESDDKLAKHSDIASLFIRKARELEGDGAAVLDVSTLRSIFLTFIFAGKDTTSSAITYT MYALAQYPNVQEKLVDELQQVKNATFTYEDVKNMRYLDAVVSETLRLYPTVPSNMKLAVKDDYLPDGTFVPAGTEVV YHTWYMGRHNPIFGDDPLVFRPERWIEMKTRPSAYDFPVFQAGPRICVGMNMALAETKMFVAVMVRHFHVKIQAG EKVEGRRYILAPTLTMESGLPLQLTPRNTASSS*

>CYP5017F8 P.irregulare

MHDMSTQYALQAIFDISIGISLHDVSKDHESSFVEAMDFVFAHLADRLMAKPYYRYFWWCMPSEYEIRRSEQ VIINLVDDILRHRLQETDQEIKPRSDIMSLFIKKERKLTEEEQGGGGRNSVLSIETFRSIFMTFLSAGKGCTLYTLSLYPDVQ QKLYDEMPEQLKRSQHITYNDLKGFEYLNAVANKTMRLYPAFPSSFKVAVQDDCLPDGTFIPAGAKVAYCPWHMGRN NPVFRSSGDVLEFKPERWLEMKTWPSAYDHPAFLAGPRICIGMNMVLIEVKVFVAVMVHHFEIAIQKGEKMEDRGYIL SPTLVMDGGLPLHMAPRLTLAATAQ*

>CYP5017F9 P.iwayamai

VATKVFTTNNFRLVTETVFYKYANMMAEIINTQGGKVDLYEISQYSLQAIFDVSCGVPLKNIDASLGLSFVKSLG IMREQSIRRLLTAPYYRFFWWCMPSEYRVKRHEKVIATLADKILLKRLQESDEVIEQRFDIISLFIKKAREQGGSSASARLLE IETLRAIFLTFIFAGWESTSSVTTWTFYILALYPEHQQKIIDEFEALDDTSDLSYDDVKKLKYLDTAVNETTRLYPTIPLNMK QAAEDDLLLDGTFVPAGTEDFRSSRWFEMEVRPTAYEFPVFQAGPRICPGMNIALLETKLFIAVMLRRFHVKIQDDAQV EDREYIFSATMTMDGGLPLQMTPRTASCY*

>CYP5017G1 P.iwayamai

MLVLPPLESVTRGQVAQAALVVVLTPVVLSLFHVVRRKMRVAKALEPIPGPKGSLLFGIFPEIVANFARLYDFQ EDLMVKYGGRIKLPGNIFKDGTVMLASAEDVKHILSDNFDNYIKSDEMMDAFRTMFGNSFMCLNHAHTHDNGQMW RIQRKVASKVFTANNFKVFTEELFHKYAQDMGDLIDAQDGKCDMSKIANLYTLSTIFDISCGVVLKDVDKKFGLSFMDS MTFVIDNLIDRLIARPYYRYFWWCMPSEYRLKRDVKVIQDIADSILQPRLAESDAEISKRHDILSLFIQKARELEGGEGQA

VLDISTLRDVMLAFVIAGWHTTASFITWLSYVIAQYPEVQEKLYDEVSKLSTTALTYDDVKKLKYLDAVAMETMRLYPTL
PFNIKIAAKDDHLPDGTPLWSEDPLVFRPERWLELKTRPSAFEFPAFQAGPRVCPGMNLSLLETKMFVAVMLQRFHFK
VQDGEQVANRPYKMGTNLVMEGGLPLQMTPRVAPSS*

>CYP5017G2 P.irregulare

MLVIPPPLQNVTLGQGVKATLVVALTPVVFSLFRAVRRKMRITKGLKPIPGPKGSLLLGMFPEIVENFARLYNY
QEELMVRYGGRVKLPANIFQDGTVYVASTEDVKHILSTNFDNYVKSETMMDAFRTGFGNSFMCLNHANTADGGEM
WRLQRKVSSKVFTANNFKVFTEELFHTYAQQMGDIVDSQGGKCDMSKISTLYTLSTIFNITCGVTLKEVDAKMGLSFM
DCMAFVIDNLIDRIIKRPYYRYFW*

>CYP5017H1 P.irregulare

MTALIRSVRYKMRIARGLAPVPGPKGRFLLGILPELMKNSPRMYNYQEELMKQYGGCMHFPWHLFSNGML
FIGSSEDVQHVLSTNFRNYVKSSHFQASFGRLLANSLVTLNHVHTEDSGAMWRLQHKLAVKVFSTRNFTLLGEQLFHKY
ALQMSELIEAQGGRCNMHDISSQYTLQVIFDITRSVPLKGVDGALGLSFIHSLNTMAEAVSRRTSSTSGGRYERIMSDIV
NKILARKLSELLGEIEKRFDIISLFIQKAPEVSQVDVDRSENPRGIADSVVSAASLLNIEMLRSVLFAFTSAGWDTTSALSTY
TFYALALYPEIKILKELENKLVEKASTVDTLPLLSYDDIKTLRYLDAVVSEVLRVHPTIVFDMKVAAEDDHLPDGHR*

>CYP51C1 S.Parasitica

MLALTDLFAEHAKLLAAFGLFLLVAIYLSLPKKTNGKLPPVLNYMIPILGSFPAFAKDPIQMVLDGYKEKGSCFTA
KMFGQDLTFLIGPKAHESFYKPNDDYLSQSEVYQLMTPVFGPGLVYDATPKRRAQQMQFMANGLRTSRLKTYVEKIR
METELYFKAFPKESTVSLKKIFSEVTILTASRALLGDEVRENLFKEVSSLYCDLDGGTTPLSFFFPYLPIPMHRRRDAARKK
MSDLFSGVIKQRRESGKRADDILDTFMNSEYKDTPGVPIPNEHIVGLLIALLFAGQHTSSLTVTWAVIELVLQTPEMIPKIL
KEQDEVLGEGGSDNLNFDTVNNMPILHACIKETLRLTPPLIFLMRKVMKDITCNEYTIPAGHIVFSSPAAACRLPDYWAE
PDKYDPMRFLDGRNEEDRQPYTNVSFGGGMHGCMGQQYAYIQVKIVLSIFFRLFKVEMVDKTFPKQDYTCLVVPPVG
ETLAKLTRVD*

>CYP51C1 S. declina

MLALTDLFAEHAKLLAAFGLFLLVAIYLSLPKKTNGKLPPVLNYMIPILGSFPAFAKDPIQMVLDGYKEKGSCFTA
KMFGQDLTFLIGPKAHESFYKPNDDYLSQSEVYQLMTPVFGPGLVYDATPKRRAQQMQFMANGLRTSRLKTYVEKIR
METELYFKAFPKESTVSLKKIFSEVTILTAARALLGDEVRENLFKEVSSLYCDLDGGTTPLSFFFPYLPIPMHRRRDAARQK
MSELFAGVIKQRRESGKRADDILDTFMNSEYKDTPGVPIPNEHIVGLLIALLFAGQHTSSLTVTWAVIELVLQTPEMIPKIL
KEQEEVLGEGGSDNLNFDTVNNMPILHACIKETLRLTPPLIFLMRKVMKDITCNEYTIPAGHIVFSSPAAACRLPEYWAE
PDKYDPMRFLDGRNEEDRQPYTNVSFGGGMHGCMGQQYAYIQVKIVLSIFFRHFKVEMLDKTFPKQDYTCLVVPPVG
ETLAKLTRVD*

>CYP558B1 S. declina

MHRASNVVNSRPRMPPTTSAFPARLLGHLGDLDLFGSSRGGRIGHRLLERFGDWYAAVNAKTFCVRLGGQD
IIVTKDPRVFKPVLGQFLAHFEPSTKARAAVGSLMPSHVFVQESADDWARVRKVLSHALAQQDVERIPSIASSIVDTLTV
PSSGTMDVVPEVVMPKLAFDMVSQLLFDWDPKTMSGESLPLLASALLVSDVIGERTMLPLPWLWRLPLPRNRDANA
ARDVLRAHVQSFVAARGPRHSPTSLLDAMVAATSDGNLSATEMRDNLLALFFGAFDNTAHTLSILFNLLATHPEVQDEL
YAALLSAFPNGASEIATATSSQLRDVPYLCWTVDEVLRLKPAVPVISRQCVAPCVIHGVEFRAGDDVLIDGGSACRDADY
GPGHSDLDVFRPSRFGDTFAALDKSVLLSFGAGKRLCPGRSIALSQVRAVCAYVIATFHVSRPASSPPLLFDLKLTVTTRA
GHGTMVWTKRKE*

>CYP558C1 S. declina

MVVTKDPAIYKAALGLSHQKYFDRAPSITAVMTYFLPRSLGAVTSHDWSRIRRTLQRGMAMQSFDNFPSLVA
SCIQKLDRATLSKQPASDNKDVLTVVPEDFFPSLTFDVFHRLMYQWDPNTVAGDSMDLLRAALHLSEMFAERVMLPFP
LLWKLPLPRNYEADAALHDLHAKVVELVNARRQYLASLDELPHPTLLDYLLGALEDETLSNDEVYDNVTGFFIAAFDTTS
NALSLLFNNLALNPDVQAKLRAALATSFPGGPDAIARATSKDLDGCDYLTMTLDESMRLTPPIHSAVRKCVQACEIHGV
RFEVGDELFMHSHGAAMDPDNYAGMDDLECFRPERFQDTTFDKPVSLVFGYGQRMCLGRHLAIAEMKAICAYVVSS
YGLSRPADGPPLKLDSTLTIGTKVGYGTMHWRRL*

>CYP558C2 S. declina

MVVTKDPAVYKAALGLSHRECFGVSPSLSVVLSYFLPRSIAVVEHQDWSRIRRTLQRGMAMQSFDSFPALVA
SSIQKLDATLSKQQSPDDNVLVVVPEDVFPNLTFDVFHRLMYQWDPNTVAGDSMDLLHAALQLGEVLADRFLLPLPHL
WKLPLARNYEADAIRDNLHAKVVELVNTRRQYLASLDELPHPTLLDYLLGALEDTTLSTDEVYDNVMGMFLGAFDTTSN
ALALLFNNLAQYPDVQTTLRAALATSFPGGPAAIARATPKDLDGCDYLTMTLNESMRLTPSVRSVNRECVSACEIHGVR
FEVGDELFVHSHGVAMDPDNYAGMDDLDCFRPDRFQDPALEKSISLGFGYGQHMCLGRHIARAEVKAICAYVVSSYEL
SRPADGPLLKYNSTLTIGTKVGFGTMHWRRLAPPLAA*

>CYP5613A1 S. declina

MWALVLASLLVVWLTWHRYSHRHHNQYPYVYSYLPYLGASIHFRHGAVRCVQRWVRQYGTIFNITMLGEPL
TLIADPALFSTISKHPQLSLLPLKTRMMGVGFGAFTYATQPDETIQSIAAATRKSLTTSLGFHKPSLDEFNHASATTLLQKL
QLSSSPKRVALYPFLNEVLFETLLEVFYGKDLATPAFRADFNAWDAKFTALYAGFPAAWLGVEEPRRRMHATLRQYAAT
SRDKLSSVVTDRLAICATHGVSPSDTDGHQLSFVWAMTTNTMRACFWAICYLLENPTAWAAVQEEVELHVGVTNTT
WDRDAIEKCVRLESAVKETLRLALNSSAVRYAMEDVTLDFPDGRRLAMRKGDGLVLQSNLAYYDEAAFPDPFNFAYDR
FLNNPSLAKDFSPFGFGKYLCPGQHYATDFVKIVVTTLMLRASITNVEGRATLQHNAVGVFIPKDPLAVHVTLQARQAP
HA*

>CYP5614A1 S. declina

MSDITSALLALASLLVLVLVWSKPSVRSNIPLVSYWIPFLGSSLAFSKDPIAFLNQCQATHGSVFTVLLAGKRMT
FFTSPKHYATILKSKTLSHKPVFSEIEQVAFGQDPTFTHYRNDLRKTDANLTRFNALPTPYLANRGAVVDLIQRTFGCQRA
VLATLAPQTHIVSLLTLVEHAIFASGSAAMFGETVLAAHPCMADDFLAFDAAFPLLVAGCVPSVFLSKAMASRQRLIDAF
KAAPLTDGAPLVQARAAALPTLACGSATDRASCHLEILWAASANSIPTAFWALFHLLATPAALTAVRDEIDAHLDKTPLA
DTAAAPWTQEMLQKCVLLDSTIDECLRLAASSMLLRVAVEPTDMVLDKTTYHFEKGDRIAIFPSLGHFDPTLFPDPTRF
QYDRFVHATKAQLEAFKPFGMGANMCPGRYFAKNQIKMWVALVLQHLPSLRLVPGFAPPTFDPSRLGLGVIPPADHA
IHVEF*

>CYP5614A2 S.Parasitica

MSDVTSTLVVLASLVGLVLLWTKPRSNIPLVSYWIPFLGSSLAFSKDPIAFLNRCQATHGSVFTVLLAGRRMTFF
TSPKHYATILKSKSLSHKPIFSEIEEVAFGQDPVFTHYRNDLRKTDANLTRFNSLPTPYLANRGAVLDLIQRTFACQRAVLA
TLAPQQHGVSLLTLVEHAIFASGSAVMFGERVLAAHPRMADDFLAFDAAFPLLVAGCVPSVFLSKAMASRQRLIDAFKA
APLTDGAPLVQARASALPTLACGSATDRASCHLEILWAASANSIPTAFWTLFHLLESPAALKAIRNEIETYLDKSPLSDTSA
APWTQEMLQKCVLLDSTVDECLRLAASSMLLRVAVEPTDVVLDKTTYHFEKGDRVAIFPSLGHFDPTLFPDPTRFQYDR
FVHATKAQLEAFKPFGMGANMCPGRYFAKNQIKMWVALVLQHLPALRLVPGYTPPTFDPSRLGLGVIPPADHAIHIAF
*

>CYP5614A3 S.Parasitica

MSDVTSTTLVVLASLVGLVLLWTKPHSNIPLVSYWIPFVGSSLAFSKDPIAFLNMCQATHGSVFTVLLAGRRM TFFTSPKHYATILKSKSLSHKPIFSEIEEVAFGQDPVFTHYRNDLRKTDANLTRFNSLPTPFLANRGAVLDLIQRTFACQRA VLATLAPQQHGVSLLTLVEHAIFASGSAVMFGERVLAAHPRMADDFLAFDAAFPLLVAGCVPSVFLSKAMASRQRLIDA FKAAPLTDGAPLVQARASALPTLACGSATDRASCHLEILWAASANSIPTAFWTLFHLLESPAALKAVRDEIDTHLDKSPLS DTSAAPWTQEMLHKCVLLDSTVDECLRLAASSMLLRVAVEPTDVVLDKTTYHFEKGDRVAIFPSLGHFDPTLFSDPTRF QYDRFVHATKAQLEAFKPFGMGANMCPGRYFAKNQIKMWVALVLQHLPALRLVPGYTPPTFDPSRLGLGVIPPADHA IHIAF*

>CYP5614B1 S.Parasitica

MLQLSHGLIATAVVLTVVLAVVLATRKTSVPTVRWYLPLLGSALAFAASPVAFLRACCAEHGSVFRVLLAGRY MTFFTSPAHVTVLLQTKALSFGPVVHDVCVAAFGHDSGFPHPSQWHYRQVHSLFTQHFVNGTGLSALVEANVLQQRR ALQRRLAAPGASQLHLFSFLRQALFESSSRTLFGDAFCDLHADLLHDYVAFDDAFPVLASGYVPTWLLRTARAAASTMA QGFSDAIVEPSLMRGCSQFMLDRLQLFRRVQAPVHNCAGTQLALLWATNSNSVPTAFWTLYHLLHDVAAWDAVLAE VHEYLPSTALESKATWSKADLDKCIVLDSAIDETLRLVSASLLLREATCDVELPLEPPVVLRKGDRVAMAPFLQHMDATLF PEPEIFRFDRFVHATQAQRSALRPFGLGASMCPGRFFAKTQLKSCIALLMQHLPGLHFASLPASTVAINQTRVGMGVYP PATDTVVVVFDRA*

>CYP5614B2 S. declina

MLPLIVIVAAVVLTVVLALKLATRQTGVPTVRWYIPLVGSALDFAASPIAFLRACRTKHGPVFRVLLAGRYMTFF TSPAHVAVLLQTKALSFGPVVHDVCVAAFGHDAGFAHPSQWHYRRVHSLFTQHFVNGTGLAALIEANVLQQRRALQR HLAASGSSQLRLFSFLRQALFESSSRTLFGDAFCDTHADLLDDYVAFDDAFPVLASGYVPTWLLRTARAAASTIAQGFGD AIVEPSLMRGCSQFMLDRLQLFRRVQAPLHNCAGTQLALLWATNSNSVPTAFWTLYHLLHDVAAWNAVLAEVHLHLT SPALASEATWSKADLDKCVVLDSAIDETLRLVSASLLLREATCDVELPLEPPVVLRKGDRVAMAPFLQHMDAALFPDPEI FRFDRFVHATQAQRSALRPFGLGASMCPGRFFAKTQLKSCIALVMQHLPGLRFASLQASTVAINQTRVGMGVYPPATD DVVLAFDRAAV*

>CYP5615A1 S.Parasitica

MLRSCLRLKSTVAAAPKPVTAIPNAPETGVGPLRSSMMYFSKGGGFQVLFKHLRSLHDTIGPILRVRFVPFGPH MVSVTDLDVIAQLHRNEGPIPLRGAMPVWEYYRQVKKWPRTTITTNDYATWKKMRSSLSDHVLQPHNIGPWLPRID AIAQDTVMRFQREAAASPTADVNVTHTLKAYTLESVSSVLFGKRMGCLSPDHLTPITPAAERFINAVVGFFETTQALVNL PPSMPPVLFWLFPAFWSHVGHANVIFEIGVELMTEKLASTETTAPDLLTAFLLRPELTEREAVTQCIDLLFAGVDTTSQGI LWTLYAIASSPNAHDLQRRLHDEVAAAMGDKAHMDEDTLSKLPLVRACVKEALRLYPVFSTSMRIMRDDTTLLGYSVP KGTQVLLPIYAMSRDRSVFAEPDTFVPERWLRRDTKSAADRKKAAYATLPFGMGARSCAGRRLAETELYLLLAHMVRT MRFECSPGADHPEPIVQLALTPDKPLVLRVTPW*

>CYP5615A10 S.Parasitica

MLRHAIGRRAKSTAARAWDDIPTAPEYGVGPFKSSGLYFFRKNGLRVTYERFQTLHRELGPIFKLRFAPFTPYL VSVADPDATALVCRTEGAMPQRHLFPAWTLYRQQRQWPMATSNTNEYAAWKKSRAGMSDNLLLMHNVPTWIGRI DDVAKDVVARLAAEAQNGSPVPVTNLMKAFALEAVSSLVFGKRMGCLSPNPSTPIPAAAQAFIDAVDGFFDTTQQLHI IPPEVPTWIYPFLPAYRAHAAHCDYIFDLGYELMRDKIASTDASPEPDLLGAFLERPELDERDAISLAIDVLFAGVDTTGNT LLWTMYCLATAPNGREMQARIRDEIGSGPIDETALGRLSYLRACVKETLRLYPSATPNQRYAGEDVTLAGYHVPKGTSV

LMATYTMSRDPSIFEDPEVFAPERWLDREKKGADSRKQQAYSTLPFGIGARSCVGRRLAETEMYVLLAHILREMEIQWI
PNETHPKGILRLLIVPDKPLSFRFQPWSS*

>CYP5615A11 S. declina

MLRQSAGRRAKSTAARAWDDIPTAPEYGFGPFKSSGLYFIRNNGLRVTYERFQTLHRELGPIFKLRFAPFTPYL
VSVADPEATALVCRTEGAMPQRHLFPAWTLYRQQRQWPMATSNTNVFAEWKKSRASMSENLLQMHNVPAWIGRI
DDVAKDVVARIATEAQHGAPVAVTNIMKAFALEAVSSLVFGKRMGCLSPDPSTPIPPAAQAFIDAVDGFFDTTQQLHII
PPEVPTWIYPYVPAYRAHAAHCDYIFDLGYQLMRDKIACTDASPEPDLLSTFLQRPELDEREAISLAIDVLFAGVDTTGNT
LLWTMYCLATAPNGREMQARIRDEIGSGPMDEAALTRLSYLRACVKETLRLYPSATPNQRYAGDDVTLAGYQVPKGTS
VLMATYTMSRDPDIFEHPEVFAPERWMDREKKDADNRKQQAYSTLPFGIGARSCVGRRLAETEMYLLLAHLLREMEIQ
WIPNEAHPKGILRLLIVPDKPLSFRFQPWSS*

>CYP5615A2 S. declina

MRSVHLRISSMLRSCLRLKSTLAAPKPVTAIPNAPETGVGPLRSSMMYFTKGGGFQVLFKHLRALHDTIGPILR
VRFVPFGPRMVSVTDLDVIAQLFRNEGPVPLRGAVPVWEYYRQIKQWPRTTVTTNDYAIWKKMRSSLSDHLLQPRNI
GPWLPRIDAIAQDVTTRFEREAAASPTRDVDVTHTLKAYTLESVSSVLFGKRMGCLSADHLTPITPAAQRFINAVVGFFE
TTQALNTLPPSMPPILFWLFPAFWRHVGHANVIFEIGFELMTEKLASTEATAPDLLTAFLLRPELTEREAITQCIDLLFAGV
DTTSQGILWTLYAIATSHDALDLQRQLRDEVTAAMGDKTQLDKDALSKLPLVRACVKEALRLYPVFSTSMRVMHDDTT
LLGYSVPKGTQVLMPIYAMSRDRSVFAEPDTFVPERWLGREAKSAADRKKAAYATLPFGMGARSCAGRRLAETEHYLL
LAHMVRTMRFEVAPGVDHPEPIVQLALTPDKPLVLRMTPW*

>CYP5615A3 S.Parasitica

MLRARLKSTLAAVAAEIPAPLSAIPTAPEVGFGPFRCSLAFFGKDDGLGRHHERFDTLHKMLGPIYKFRVLPFSP
YMVSICDQLSVAHVYRKEGPFPERFSLACWRLHRREKQWPIGIVNAHNHEEWKKFRASMSEHILQPHNITTWLPRLN
DVAKDLATRLAADAASSEPVNVALPLKAFGLESVSSLVFGKRMGCLSPDPTTPVPPDAQAFIDAFYGVLHTSQDMLNFP
QETPDWVYKLLPVYKEFVQHADYIFDVGSAFVLEKMAARDNSIAEQDLLSVFLARPEFDQRDAVAQAVDLLFASVDTT
GTALLWITYCLATAPNGRAIQIKMRDEANAALAGKSDIDDAALEQLSYIRAVVKETLRLYPVANPNQRVLPNDLTLLGYSI
PKGTSVLMQTYTMSRDPTVFENPNEFKPERWLERDGKTASQRQAAAYSTLPFGMGARGCAGRRLAETEMYMLLAHL
VRKMELQWPEGQEHPDAIIKTFMMPNKEFKLRLVPWSSS*

>CYP5615A4 S. declina

MLRVRQCARLKSTLAAVAPEIPASLSAIPTAPESGFGPFRCSLAFFGKDNGLGRHHERFDTLHKMLGPIYKFRV
LPFSPYMVSICDQLSVAHVYRKEGAFPERFSLACWRLHRREKQWPIGIVNAHNHEEWKKFRASMSEHILQPHNITTWL
PRLNDVAKDFATRLAADAASSDTVNVALPLKAFGLESVSAIVFGKRMGCLSPDPSTPVPPDAQKFIDAFFGVLHTSQDM
LNFPQETPDWIYKLLPVYKAFVKHADYIFDVGSAFVLEKMAARDNSIVDQDLLSVFLARPEFSQRDAVAQAVDLLFASV
DTTGTALLWVTYCLATSPNGREIQIKMRDEANAALAGKTDIDDAALEQLSYIRAVVKETLRLYPVANPNQRVLPNDLTLL
GFTIPKGTSVLMQTYTMSRDPTVFEDPEAFKPERWLERDGKTASQRQAAAYSTLPFGLGARGCAGRRLAETEMYMLL
AHLVRKMELQWPEDQAHPNAIIKTFMMPDKEFQLRLVPWSTS*

>CYP5615A5 S. declina

MLRLKSTLAAQPPSLPVAPESGLWPLRSSFAFFAKDDGFRLRHERFDTLHKLLGPIYKIRFLPLQKYMVTIADPA
AVAQIYWHKGPMPQRRVNPTWKVHRDARQWPIGSVNTNEFAEWKRFRRSLSAHVLQAHNVAAWLPRLDDVAQDI
VARIRSQAVTDNSVPMTRVTQAYALEAASALVFGRRMGCVSRDHLTPIDAEAQAFIGAVDGFAAMTQVLGRVPPFVP

HWAYRFLPSYQAFAAHSDVIFALGETRMRQKMASTEETDPDLLSMFLARPELDERDAIAQAVDVLLASVDTTTNALLW
TLYLLASSPNGRAMQETIRDEANAALGGNASFDDAALEKVSYVRAVVKEALRLYPVANPNQRYLTSDLTLLGHKIPAGTS
VLVATYTMSRDPNVFENPTAFVPERWMNKASNRQAAAYSTLPFGMGARSCVGRRLAETEMYLLLSHLLRTMEVSWI
ATEAHPKAVIHLLMTPETELTLRFQPW*

>CYP5615A6 S.Parasitica

MLRTRLTSTLAAVAAEARPSLHDIPVAPETGFGPLRSSYASFAENDGFRLLFERFHKLHASLGPIFRLRFLPFQSY
TVSISDQDAVAEIYRHEGAMPQRQTFGFWKLYRDERKLPVGLANTNEYASWKKYRASLSAHLLQPHNVSTWVPRIDA
VANDLAARVCAQATASSSGHVTMPLLTKAYALEAVSSLVFGKRIGCLSPDHLTPIAPTAQALIDVVDGFFATSQKLMAV
PPEMPHFVYKLMPVYKEHVAYADAIFRFGHERMAEKLASTEVTAPDLLTMFLQRPELNEEEAVTQAIEVLVGGIDSTSIA
LVWSLYCIATSPNSREIQRKIRAEVDAALDGATELDAAALEKLSYIRAVVKETLRLYPAVNPNHRFLSQDMTLLGYDLPKG
TSVMMATYTMSRDPAVFDEPETFKPERWLDRDHASTAARKKKAYSTLPFGMGSRSCLGRRLAETEIYLALAHLVRRME
MQWIPEEEHPKPILSTLLVPNKPLTLRFEPWAT*

>CYP5615A7 S. declina

MLRARLKSTLAAVATEAAPCLRDIPVAPETGFGPLRSSYAYFAESDGFRLLFERFHKLHTSLGPIYRLRFMPFQP
YTVSISDPDAAAEIYRHEGAMPQRQTFDFWKLYRDERNLPVGLANTNEYAVWKKYRLSLGAHLLQPHNVGTWVPRVD
AVANDLAARVCAQASASSTGEVPVTLLTKAFALEAVSSLVFGKRMGCLSPDHLTPIAPTAQALISAVDGFFATSQQLMA
VPPKAPLFVYKLLPAYKEHVMHSDYIFRLGHEMVQEKLASTEVTAPDLLTMFLQRPELDETDAITQAIEILFGGVDTTSIAL
LWSLYCIATSPHSREIQLKMRAEVESILDGATEFDAAAHEKLSYIRAVVKETLRLYPAANPNQRILSQDMTVLGYDIPKGT
SVMMATYTMSRDPAVFDEPEIFKPERWIDRDHATPAARKKKAYSTLPFGMGSRSCIGRRLAETEIYLALAHLVRRMEM
QWIPEETHPKPILQLLLVPNKPLTLRFQPWTT*

>CYP5615A8 S. declina

MLRTRLKSTLAAVATAAEASPSLRDVPVAPEWGFGPLRCSFAHFASNNGFRFLFERIHALHASLGPILRVRFLPF
QRYTVSISDPDAVALIYRHEGAMPERQLFAFWQLYRDESRWPLGLTNTNEYAEWKRFRLGMSAHVLQPHNMSAWLP
RLDAVARDVASRVHDEARASSSGDVPMTLTTKAFALEAVSSIVFGKRMGCLSPNHLTPVSSTAEALIRAVDGFFETSQQ
LLSVPPEAPRIVYKLLPAYKEHVAHADVIFGLGHDLMREKIASHEVSEPDLLTLFLQRPELTERDAIAQALDVLFAGVDTTS
IALLWALYCLATSPNSHEIQRKMRAEVEDALDGSTEFDAVGLEKLSYIRAVIKETLRLYPVATPNQRFLSQDMTLLGYDLP
RGTNVLMATYSMSRDPTVFDEPESFVPDRWLERDRKSPMRRKHEAYSTLPFGMGARSCVGRRLAETEMYLFLAHLVR
CVEIQWVPDETHPQAVLKTMLVPDKPLTFRFQPWTS*

>CYP5615A9 S.Parasitica

MLRTRLKSTLAAVATASPSLRGIPVAPELGFGPLRCSFAHFAPKNGFRFLFERLHALHACLGPIFRVRFLPFQSYA
VSISDPDAVAQIYRHEGAMPQRQLFAFWKLYRDERQWPLGLTNTNEYAEWKKFRLGMSAHLLQPHNVQSWLPRLDA
VARDVAARIQSQARASPSGDVPMTLTTKAFTLEAVSSIVFGKRMGCLSPNPSTPVSAPAQAFIRAVDGFFETTQQLMAI
PPDAPRIVRTWASDVDFIFRLGHDMVREKIACREPRAESDLLTLFLQRPELSERDAVAQALDVLFAGVDTTSTALLWALY
CLTTSPKSREIQAKMRAEVESALDGAAEFDAMSFEKLSYIRAVVKETLRLYPVATPNQRYLSQDMTLLGHDLPRGTNVL
MATYSMSRNPSVFDEPESFVPDRWLERGRKLPQSRKQEAYSTLPFGFGARSCAGRRLAETKMYLFLAHVVRRVEIQWA
PDETHPHAILQTMLMPDKPLTFRFQPWTS*

>CYP5616A1 S. declina

MFAESAVAVTAPAIAYIYWRFGRNEPFLHGVPFPASLHQPFLGVAGLLGSINGCDRLFADAADANGMVSFRL

LTTNAVSVLKADHVRQVVCATSYREMVPILGNHLRKLLGDASLLLLSRHEWKSHRRLVAKAFHWQSLAQMVPTMVEV
AEAFVDVALQTGSGASVDVAPLLKLAALDTIGQCAFGYDFEALANGKNGVTSAFEFLLNEAKRRGFDDTLHPASVWYD
LPTPANRRYKREARILRSTLEMIVVARMSDIATTKHDLLGSLLAAAADESSVVTPATLADNLLTFLFAGFDTVATALAYAM
HLLSTHPDSIGRLPYVYAVMTEALRLFPPGPGTSRTLEADLVLDGHTIPAGTLVYLPIWEINRSRLNWGDDADAFRPERH
VEGASDEICSDRTFRMMSFSAGPRNCVGMRFAMLEAVVMLATMLRRCWLTRPSDAPPVTPKISGILQVPAHGVWVR
VTPLPPCA*

>CYP5616A2 S.Parasitica

MFTESAVAVAALAVAYVYWRFGRNAPFLPGVPFPASLHQPFLGVAGLLGSIDGCDRLFADAADANGMVSFR
LLTTNAVSVLKAAHVRQVVCATSYREMVPILGNHLRKLLGGASLLLLSRHEWKGHRRLIAKAFHWQSLAQMVLTMVA
VAEAFVDVALQTAGGASVDVAPLLKLAALDTIGRCAFDYDFEALANGTNDVTSAFEFLLNEAKRRGFDDTLHPASLWYD
LPTPANRRYKREARILRSTLESIVVARMSDHATTKHDLLGSLLAAAADESSAVTPTTLADNLLTFLFAGFDTVAIALAYAM
HLLSTHPDVQAKALAEVEAVVGRSGSITYEAIGRLPYVYAVMTEALRLFPPGPGTSRTLEADLVLDGHSIPAGTLVYLPIW
EINRSRLNWGDDADAFRPERHVEEGASDESSSDRAFRMMSFSAGPRNCVGMRFAMLEAVVVLATTLRRCTLTRSSDA
PPVTPKISGILQVPANGVWVRVAPLPPGA*

>CYP5616B1 S.Parasitica

MPDLVTTASVGVALAALAIAYLFHRYGRNEPFVAGLAFPQNLHAPFLGVAKHMGKLEASARIFVDAANADG
MVSFRLMSMRVVAVTKAEHVRAVVCSSSYRKPMPLIQKHIRELLGYNSLPVLMHDEWKVHRRIFARAFHWEYLAAM
VPAMAKVAAEAASVLLAASSADVYRVLQLSALDTIALTGFGFNVNALRDSSHPIAEAFEFLLSETTRRCFEAPLRPTSYFYS
IPTPANRRYRREVGVVRTQLGDIIAARLASPTNAHPDLLQALLDAAAAEDDPMTPDALADNVLAFFFGGYDTTSTGMA
YSLYLLATHPDVQAKAVAEIDTVVGRSGAITYELLQQLPYLNAVLTESLRLFPPTPVTSRHLETNLVLSGYTLPAGTMVYLP
IWYINRSTRNWGDDALDFKPERHLIDHMDGDVGAKDRAYRFMSFSGGPRNCVGMRFAVLEATCMLVAVLQKCVLSR
SEDAPPLQPKARGIVQKPELGVWLQMSPRSSPPPA*

>CYP5616B2 S. declina

MTDLVTTASVGIALAALAIAYLYHRYGRNEPFVPGLAFPHELHAPFIGVAKHMGKLEASARVFVDAADANGM
VSFKLMSMHVIAVTKAEHVRAVVCSSSYRKPMPLIQKHIRELLGYNSLPVLMHDEWKVHRRVFARAFHWEYLAAMVP
AMATVAAEAASVLLAASTTDVYRVLQLSALDTIALTGFGFNVHALRDSSHPIAEAFEFLLSETTRRCFEAPLRPTSYFYSIPT
PANLRYRREVGVVRAKLGDIIAARLASPTKGHPDLLQALLDAAAAEDDPMSPDALADNVLAFFFGGYDTTSTGMAYTLY
LLATHPEVQAKAVAEIDTVVGMSGAITYEVLQLLPYLNAVLTESLRLFPPTPVTSRHLETDLVLSGYTIPAGTMAYLPIWYI
NRSTLNWGNDALDFKPERHLTDHMEGDVGAKDRAYRFMSFSGGPRNCVGMRFAVLEATCMLIAVLQTCVLARAEGS
PPLQPKARGIVQKPELGVWLQLSPRLRPPPA*

>CYP5616B3 S. declina

MITLTAFGVDIYALRDNMHPIGKSFCFLLGEVSPSELPTATNRRYHREAAIVHNTLNKIITERGANPGGAHPDLL
QALLDASASETDPIAPEMLIDNVVMFFFGGYDTTSTGMGYTLCLLAAYPDVQAKAVAEIDAVVGRDGKIKYEVLQNLPY
LNAVLTESLRLFPPLPATMRNLETDLDIGGYTVAAETTVFLSIWCINWSRLNWGNDVLEFKPECHLTTDANDEQMSAK
DRAYRFMTFSGGPRNCVGMRFATLESTCMLVSILQQCVLTRPKDAPPIQPKLVGIVQKPELGVWLHVAPRPTPRYA*

>CYP5616C1 S.Parasitica

MEGSVMSAGAAILVIVVAYVYHRYGRNAPFLPGLPFPAGLHRPFVGVAVEMSTLEGMTRLFVDAADANGM
VSFRLLSQPSVAVTRADHVRQVYMATTNRGPTPIIDMHMQAFLGDRSVSLLMRDEWKLHRRLVSRAFQWQNLAAM

VPAMASIAETFTNVWRGAATPSIDVFPLLKRLALDTIGRTGFGYDMGALRDAANPMASALDFLVNEINRRCLEQPLHPS
SHLYWLPTRTNRRHAEAVSTVRTTLHSIIQARMMSHATSSLRHHDLLEAMLEAASADNSPMDAKTLADNVVTFFFAGS
DTTSSAMAYTLFLLATHRDVQAKAVAEIDKVVGQSSPITYEAMQQLPYVSAVLTEALRLYAPAPFTYRHADSDLVLGDH
VVPAGTLLALPMWFINRSPLNWGDDAAEFKPERHLAADCDRFAAKDRAYRFVSFSGGPRNCVGMRFAQLEATVLLTT
ILRQCIVTRPSDAPSVEPTAVGISITPEGGMWLSLTPRAAVSA*

>CYP5616C2 S. declina

MEGSVTSAGAAILVILVAYVCHRYGRNAPFLPGLPFPTGLHKPFVGVATEMSTLKGMTRLFVDAADANGMV
SFRLLTQRSVAVTRADHVRQVYMATTNRGPTPIIDMHMQAFLGDRSVSLLMRDEWKLHRRLISRAFQWQNLAAMVP
AMASVAETFVGVWLQATTPAIDVFPLLKRLALDTIGRTGFGFDMGALLDEANPMASALDFLVNEVNRRCLEQPLCPSS
HLYWLPTRMNRRHADAVRTVRTTLHNLIQARIATYAASSLRHHDLLEAMLDAASADNSPMDAKTLADNVLTFFFAGS
DTTSSAMAYTLFLLAMHPDVQAKAVAEIDTVVSTTSPITYEAVQRLPYVSAVLTEALRLYAPAPFTYRHADDDLVLGDHV
VPAGTLLALPMWFINRSPLNWGNDATEFKPERHLASDCDRFSAKDRAFRFASFSGGPRNCVGMRFAQLEAAVVLTTV
LRQYIVTRPRDAPSVEPNAVGISITPEGGMWLALTPRAAVSA*

>CYP5616C3 S.Parasitica

MLDVPSLVAAILIVALAGVCHRSGCNAPFLDGLPFPAALHVPFVGAAMQLSTLSGMRRLFVDAANDNGVVSY
RSMVVNACSYRAPVPVIDMHIARLIGKRSLVLLMRDEWKLHRRLVSRAFQWQNLAAMVPAMALIAETFTNVWLNAE
TPSIDVFSLLKRSALDTIGRTGFGYDLQSLHDANNPMASALAFLLSETNRRCFDEAHKPLSHLHWVPTPANRRFVKEATL
ARSILNEIVEARLRALHADTSEATYYHDLLQALVDAASADNSPMDAETFADNVFTFVLAGSDTISTSMAYGLYLLAMHP
DVKATAVADIDAVVGKTTPITYEAMQRLPYVSAVLTEAMRLFPASPIAVRNLEAPLMLDGHCIPAGTIMTIPIWFGDDA
DEFKPERHLDDTSAVTSGVAAKDRAFRFMTFSGGPCNCVGMRFAQLEATVMLVTVLRRCVVSRPDDAPCVIPNAVGV
SITPEKGMWLSLASRAAVSASLKCDPKDL*

>CYP5616C4 S. declina

MLDVASLIAASLVVALAVVCHRFGRNVPFLEGLPFPSALHVPFVGAAMQLSTLSGMRRLFRDAANANGIVSY
RIMAQPSVAVTRADHVRQVVNASSYRAPVPVIDMHIERLVGKRSIVQLMHNEWKLHRRLVRAFQWQNLANMVPA
MASIAESFTDIWLQAMTPSMDVFTLLKRSALDTIGRTGFGYDLQSLRDVHNPMATALAFLLSETNRRCFDEAHKPLSHL
HWVPTHANRRFVKEATLARSILSEIVQARLNALHTGTSGSTYPDLLQAMVDAASADNSPMDADTLADNVFTFVLAGSD
TISTSMAYALYLLATHPEVQAKAVAEIDKVVGTSAPITYEAVQQLPYVSAVLTESMRLFPASPIVIRHLEAPLTLDDHCVPA
GTIMTIPIWFVNRSPLNWGDDADEFKPERHFDDAVAHGIAAKDRAFRFMTFSGGPRNCVGMRFAQLEAMVMLVTV
LRRCVVSRPHDAPCVMPNAVGVSITPEQGMWLSLTPRTDPA*

>CYP5617A1 S. declina

MLATSELLTLGIPAVLAVCVAALLVARRNPYAHLPGPPPTSWLFGNGKEAMDFALWEQDGGFGEPHANGSIF
YYRMLHIYRVIVTDANAIKHSLRNKNPREIEAYNNLAPSFFTIIGFVFVPFYKYLPLASNRRRAEAQSVLESVIDRVLQQKLS
ALKTERTGAPNDILDLILAHASGMSMANIRALLLMFMIAGHEFVAQHPQVAEKVKDECSRMRGESTDETPRWEQLGQ
LSYLSAVIHETMRMTPTLADVSRVALEADALPMDGGSWVVIPKGANIYFDIVAIHRNPTYWSQPDETSRIYSYLAERFIE
GTDVYEADLVLRGGKPNTFCYFPFGLGDKNCIGSRFAMAEMLVVLSTLFARYHVALTPAANVNVRKLTATITPMHLEVS
LSLLPHN*

>CYP5617A2 S.Parasitica

MKRYGRVFYYRVLHMHRIVITDADAIKHVMVSKAKNYPRSPLVRTLFKVLNQAVLYIPTVEKKLGGGDTLVTT

EGSAHAALRKLFNPHFGQANVKTMLHVFQHHTDVFMEQLAIDQPLDLKNRTRQRGPHPFSCDLVFTQVALDMIGVA
AFGYDFESLRNKNPREIQAFHDNNLTPSLFGVIGFALVPFYEYLPLASNRRRAEAQSVLQSLIDRVLQQKLIALETSTAGSA
PKDILDLILAQASDMPMADIRALLLMFMFAGHETTDNTLAWVVSFVAQHPTVAAKVHDECLRMRCDGNDKVPTWE
QLGHLSYLSAVIHETLRLRPTVPETSRHAAEADTLPLGDGSSVGIPKGADIFLDIIAIHRNPTYWSQPNEFLPERFIEGTDVY
EADLALRGGKPNTFCYFPFSVGDKNCIGSRFAMAEMLVVLSTLFSTYSVALTPAANLNVRKDTVAIAPVYLEVTLSPLPTR
APFV*

>CYP5617A3 S.Parasitica

MVSKAKNYPRSPLVRTLFKKLEGGDTLVTTEGYAHAALRKLFNPHFGQANVKTMLHVFQHHTDVFMEQLAI
DQPLDLKNRTRQREPHPFSCDLVFTQVALDMIGVAAFGYDFESLRNKNPREIQAFHDNNLTPSLFGVIGFALVPFYEYLP
LESNSRRAEAQSVLQSLIDRVLQRKLIALETSTAGSAPKDILDLILAQASDMPMADIRALLLMFMFAGHETTDNTLAWVV
SFVAQHPPVAAKVHDECLRMRCDGNDKVPTWEQLGHLSYLSAVIHETLRLRPTVPETSRHAAEADTLPLGTSRLPLPLL
HRACPRALDIFFDIIAIHRNPTYWSQPNEFLPERFIEGTDVYKADLTLRGGKPNTFCYFPFGVGDKNCIGSHFAMAEMLV
VLSTLLSTYRVALTPAANLNVRKDTVAIAPVYLEVTLAPLPTRSPFV*

>CYP5617A4 S. declina

MMLFTIGRLTLPELSLLVATVVGVFLWLAARRSPYGHLFGPPPTSWLFGSGREAMDFSIWSQDGGFGEPGL
MWLKRYGSVYYYRVLHMHRIVVADPVAIKHVMVTHGRNYPRAPITRIISKKLAGGESLLSTEGRSHTALRKLFNPHFGQ
ASVKTMLGIFQHHTNVFMEQLTAKEPVDLQNLFTKMTLDVIGVAAFGFDFQSLLDKNAREIQAYNDIHLTPSFFGIIGFV
FLPFYEYLPLASNRHRAEAQAVLQGVIDRVLQQKVAALNSAKARDAPKDILDLILEQASDMSMADIRAMLLMFMIAGH
ETTDNTLGWIVSFVAQHPEVAANVHEECSRMRGDGNNTVPTWDDLSKLPYLSAVIHESMRLRPTVPELPSRLALEAD
MVPLTDGSHLAVPKGANIFLDIIAIQRNPTYWSRPNEFLPERFIEGTNTYEADLALRGGKPNTFCYFPFGLGDKRCIGNRF
SMTEMLVVLSAFFAKYRVALTSAADVNVRKHTGTIAPVHLEVHLASIATK*

>CYP5617B1 S. declina

MLVQALWTLALAGAVCYYLCIIRSRRQMLAKLPSPPASSWFLGHAREVMGDVSGTYPNPALSWLEEYGCAYY
FRLLHLGRVALADPVGLKHVLVTHASRYPRDDTTRTFLRTYMSGDSLVSVKGADHASMRKALNPHFGLAHLKLFVPIFV
AHTNAMLAHFTRAIDTNTPVNLHKVWSLLTLDIMGSSMFGYDFGALAGTNAENNQAYVDAQFPPSIIGMVGTVLVPG
WSYLPLPGYARRRRAIATLPGIVMRVIEAKMAQAIDDHPPQDLLDLMLISDRNMTSAEARVHVQVFMQAGHETTSHA
LSWAIAMLLRYPNVAAKVQAECDATVARFGSDVSYEAVADLVYVTAFLKETARCYPAITMLAPRSVTIDDVIPLADGSSV
ANPKGTSIYVDLVAVQRNSKYWTRPNDFCPERFMEGSALSNADRELRGGAGHTFHYLPFSMGEKNCIGQRFAMLEM
QILLVKTLAAVTFKATDRTNLNPSRELVSITPTCLEVTVHRRSATTA*

>CYP5617C1 S.Parasitica

MLADAAWILLTLLLAVVVPICIAYVVLCPSDHGFSYARLPGPPPSHWLLGNARDIFQARWNEGSFPEPGLSWL
KQYGSAYHVRILYIHRIALADPVALKHVLVTKAKDFPRNNLSRAFITKLVQGVGLLSAEGDDHMAMRRMMNPHFSHSN
LKSFVPLFAAHTRRLLSQWRTDKLVDTNAPVNLYAYFTKLTLDIIGISAFGYDFGAITGTDEAESNAYNDAKLPATLFSILG
TAYVPGWRYLPLPGFARRRAARDTLMAVVLRVIEAKMAAPAVDPPRDLLDRMLAAGPDMSATEARAHVFTFMQAG
HETTSNTLCWVVALLSQHPAIEAAVRAECQAALQQCPDGSFTADGLAHIPLLTAVIQETLRYYPTVINLAARGVTKDEVV
PLSDGSLLHVPKSANLWVDVAAIHRNPAYWTRPDEFVPDRFIEGTEVHGADLHLRGGKGHTFFYFPFSAGEKNCIGHRF
ALMELQTILVHVLASVRFAVTPAAVLHPKRQLATITPVLLETTIHSV*

>CYP5617C2 S. declina

MMLADVANVVLPLLLAVVVPLMIAYVMLCPVDHRFSYARLPGPEPSHWLLGNARDVFQTRWSEGSFPEPGL
SWLKKYGAAYYVRVLHIHRIALADPVGLKHVLVTNAKQFPRNNLSRAFLTKLVQGVGLLSAEGDDHAAMRRMLNPHFS
HTNLKSFVPLFAAHARHLLSQWAADKLIDTNAPVNLYTYFAKLTLDIIGISAFGYDFGAIAGTNEAESSAYNDAKLPATLFT
ILGTAYVPGWRYLPLPGFARRRAARDTLMTIALRVIEAKMAAPAVDPPRDLLDRMLVAGPDMSATDARAHVFTFMQA
GHETTSNTLCWVVALLSQHPAIEMAVRAECQAALQQCPDGFTAEGLAHIPLLTAVIQETLRIYPTVINLAARGVTKDEVV
PLSDGSQLHVPKSAMVWVDVAAIHRNPAYWTRPDEFVPDRFIEGTDIHSADLHLRGGKGHTFFYFPFSAGEKNCIGHR
FATMELQTILVHVLSSVRFAVTPAAVLHPKRQVGTITPVLLETTIHSI*

>CYP5617D1 S. declina

MELMLMGSAGALIALGLFVYTLMQPYVRGLYKLRGPPSSSLLLGLAADLGGIDWDRGNYPQPIVRYAREYGM
VYYNRLFHMHRMAMMDPVGLKHILVTRSDIYPRCNITRTLFQKLARGVGLLSAEGAVHDRMRKQLHPHFTPVQIKSFL
PIFEQHAKAFLGHLPPSTPIDLLERFTQLALDVIGVTAFGFAFNAIQGTRSDEIRACEDCKIPISILANVGAAYLPYWDSLPL
PWVARQRAAKDVLQQIVTKVIDAKLASPKTHTARDLLDLMLLSDVSAADARVHAATFLQAGHETTSNTLAWVVVTLTA
HPEMAERVHCECHAVLARHPTGLTSDAVGELVLLTACIHETLRLYPTAPTLSDRIATQDNVVPLMDGDSFFVPKGTQVG
IDVLAIHRNPRFWTDPDAFLPGWRVQTRSGREIQQSVDRFVEGHALQRADKALRGELSQSFFYLPFSTGDKNCIGQRF
ALLEMQVALVYLLGTFRVHLTPRANVCPKRDLTTMHPTLLEVTLTAL*

>CYP5617D2 S.Parasitica

MAMLDPVGLKHILVTRSDIYPRCHITRTLFQKLARGVGLLSAEGAVHDRMRKQLHPHFTPLQIKSFLPIFEQHA
KAFVSHLQPSTPIDLLERLTQLALDVIGVTAFGFVFDAIKGKRSDEIRACEDCKIPISILANVGAAYFPYWDHIPLPWIARQ
ARAKDVLQQLVTSVIDAKLASPKTRTARDLLDLMLLSEMSAADARVHAATFLQAGHETTSNTLAWVIVTLTAHPEMAE
RVHRECQDVLALHPTGLTATAICDIVFLTACIHETLRLYPTAPTLSDRIATQDDVVPLVDGHSFFVPKGTQVGIDVLAIHR
NPRFWTRPDAFLPGTTGNERLSRLKQCVDRFMEGHALQRADKALRGELSQSFVYLPFSTGAKNCIGQRFALLEMQVAL
VYLLGTYRVHLTPRANVFPKRDLTTMHPTLLEVTLTA*

>CYP5617E1 S. declina

METTMVLMALALLLVPLSVFMWLAPYQRSLAKKLPSPPLLHTLFGHASGVMGIDWTTGNYPEPGVSWLRRY
GLAYYHRAMHIQRITLADPVGLKHVLVTHSSKYPRDHISRTIFSKMFRGVGLLSAHGAGHAAMRKQLNPHFTHLQLKSF
VPIFERHATRFRKALLALPPLTPIDLPAQLTRLTLDIIGVSAFGYDFQGVLNSTSDELRAYDDARLPSSLVLTLGMTYLPFWE
YLPFADIAKRRHATDTLQRIVLRVIEAKMLAPTTVSNDLLDLLFASGPRLTPAEACVHVMTFLQAGHETTSNTLCWVLA
MLAKHPSVADRVHAECKIVLERFPGPLPSEALGELPYLTAVLQETLRCYPTVPALAARDALEDDTIPLQDGSTLFVPAGTA
IWIDIVAIHRNPAFWTNPDAFLPERFLDGNALSAADKALRVGKSSTFFYLPFGAGDKNCIGQRFAMMEMQVILLHLLAS
HTFPLTERACVHPKRDIATMTPVCLETTFECRQAVAASS*

>CYP5617E2 S.Parasitica

MGIDWTAGNYPEPGVSWLRRYGLAYYHRAMHIQRITLADPVGLKHVLVTHSSKYPRDHISRTIFSKMFRGTG
LLSAHGAAHAAMRKQLNPHFTHLQLKSFVPIFELHATRFLKVLLTLPAQTPIDLHAHFTKLTLDIIGVSAFGYDFEAVLDST
SADLRAYDDARLPSSLLLTLGMTCIPFWEYLPFADISKRRRATDTLQRIVLRVIEAKMLAPTAGSSDLLDLLFASGPRLTPA
EACVHVMTFLQAGHETTSNTLCWVLAMLAKHPAVADRVHAECKAVLERFPAPLPYEALSEVPYLTAVLQETLRCYPTV
PALAARDALDDDTIPLHDGSTLSVPAGTAIWIDIVAIHRNPAFWTHPDAFLPERFLDGDALYAADKALRDGMSSTFFYLP
FGAGDKNCIGQRFAMMEMQVILLHLLASHKFPLTKRACMHPKRDIATMTPVCLETTFEPHQAVAASS*

>CYP5617F1 S. declina

MSTRHDARMDASSSVIVGVGTSVVAMLVAWLLYRARFHPLQALRGPPSTHVIAGNGREFANFAWSATRPF
PEPFASWTKTYGTAFYYRVLFQHRVNLADPVSLQHVLVTKAANYPRDTIPRSILRTLLGGDGLLSSEDQVHAGQRKHLH
PPFSQTNLKAFVSIFAAETATLLSVLDAMPDGHIVDVPDLLTRLTLNVIGKTAFGYDFNTLDPTNNQAAIALSAFDKLNTP
PRPLYILGLAFLPHFEHWPLPYLRQIQTAKSMLFRIVDDVIAHKVATPAPAFDLLDLLLADAAMTATEARVHVMTFMFA
GHETTSSTLSWVLAMLATHPAVEAAVVDECRRALANAGGAVSWQTLSDLSLLTATIYETLRLYPTVPFITMRTCTHDD
WIPRTDDDPFFMPKGAQATFCVGAIHQHARYWSRPTEFLPARFLDGSPEHEADALLRHGNSSRFTFVPFSLGPKNCIG
KRFAIAEMQTVLLHLLPRFALTRTPASNLTPKLTGVTIKPTALPLRLLRR*

>CYP5617F2 S.Parasitica

MADVDSRVAVAALTGGLALVGFRTLRRSQVLTQALPGPRRTSIVYGNAREFGKTRWHDGQRFPEPFVTWQT
DFGGAFYYRVFWHHRVNLSDPTSLQHVFVVNAANYPRASIPRALFQNLLGGDGLLSSEDPAHAAQRKRLNPHFAQSVF
KAYIPVFAAETETLGKTLDSLTGPVSVPNLMTRLTLNIIGKTAFGYDFKTLDPTNDQAAIVLSAFEKLNTPPRFLYTLGLTLL
PGFQHWPLPYLRQIQAAKATLFRIVDDVIAHKVTSPSRDLLDLLLADAAMTAAEARVHVMTFMFAGHETTSNTLSWVL
AMLATHPTVEAAVVDECRRALADAGGTVSWQTLGDLPLLTATIYETLRLYPTAPFITTRHCVADDSIPRTDDAPFFMPA
GAHVSFSVGAIHRNPLYWTDPDAFVPQRFLDDSTQALADKALRNGKGNTFTYMPFSAGAKNCIGKRFAVAEMQTVLL
QLLPTYAFTLAPDANLHPKLTGVTIKPSNLVMHVSHRAASCAKGNTTK*

>CYP5617F3 S. declina

MADVVAATVAALTGGLALVGVRFLRRGQALRALPGPRSTSVVYGNAREFGKTRWHDGQRFPAPFVSWQN
DFGGAFHYRVFWHHRVNLSDPTSLQHVFVVNAANYPRASIPRALFQNLLGGDGLLSSEDPAHAAQRKRLNPHFAQSIF
KGYIPVFAGETETLASTLGTLTGPVSVPDLMTRLTLNIIGKTAFGYDFETLDPTNKRAAIALEAFEKLNTPPRFLYTLGLTLLP
GFQHWPLPYLRQIQTAKSTLFRIVDDVIAHKVASPAPARDLLDLLLADAAMTPTEARVHVMTFMFAGHETTSNTLSWV
LAMLATHPAVEAAVVDECRRALANAGGAVSWQTLSDLPLLTATIYETLRLYPTAPFITTRHCVADDSIPRTDDAPFFMP
AGAHVSFSVGAIHRNPRYWTNPDAFVPQRFLDDSTEALADKALRNGKGNTFTYMPFSAGAKNCIGKRFAIAEMQTVLL
QLLPKYAFTLATDANLHPKLTGVTIKPSNLVMHISRRAPSCAATLF*

>CYP5617G1 S. declina

MLSPTQLALLVPVASSAVWFLYHFFLIPRIHRLNALPGPPPKSIISGNAKDMVKFAWSEEDPYPGVYGKWLRT
YGPAYHLRLLHFHTVVLADPDALKHVFVSRADNYIRHATQRGLYWSFTGGAGVLTTEGDVHAAQRKMLNPHFGYVNL
KTFVRIFNTHAAVFASRLASLADGNTVLDLHERMTKLSFDIIGLAAFGYAFKSQENESVSILDAFEALNMTPTFASIYGHA
YIPGFEHLPLPFLRKRKAAKAVLAQVVDDVIARKLAASAESANKDLLDLVLESDENMTPHEARVHVLTFLLAGHETTSTSL
AWVLALLAQHPAVAAAVADECRRVLAKAGDDGLSWDDLGELKYLKAVINETQRLQPTAGILTLHCVADDLVPRADAE
PFFLPAGSRITLHIGATHRNPQYWSRPDEFLPERFLEGSALFAADKDLQRGRSSTYFFLPFSNGPRSCIGSRFAIAEMLVVL
THALARYDFRLDVSANTHAKIAGLTYKPTKLAMTVVPRTN*

>CYP5617H1 S. declina

MRVFATERLAIADPDALKHVLVSNAKNYPRHIISRSLLRSFTGGIGLLSSEDPTHAGQRKMLNPHFAYAKLKTF
VDIFAQHAQTFNALMAPKADSCDVVDMFDLMTKLSFDIIGLAAFGLDFNALHNDSADIMAAFDDLNTVPTLLFAFGN
AYVPGFENLPLPDLRKRKAAKKVLFQAVHDVIATKLSAPPTTSRDLLDLMLDSPEHKITADEARVHVMTFLFAGHETTSN
TLCWVFAMLATHPDVEARVVDECQRVMATGQLSWEAVGDLKYLTAVIYETLRINPTVTNLATRVCAADDYIPMVDGK
PIFCPAATLGMNEMLRACHSLVALSSSHPMT*

>CYP5617H2 S.Parasitica

MDIFASVHPVLLVGSAAVVALGAYFTYLFVVSPFLHPLNALPGPPPTSIFFGNGKELAQFAWDEATPFPGVQGQWLEQYGPAYHVRVLATERLAIADPDALKHVLVSNAKNYPRHVISRSMLRSFTGGIGLLSSEDPTHAGQRKMLNPHFAYAKLKTFVDVFSQHARTFNELMAPKADASDVLDMFDLMTKLSFDIIGLAAFGLEFNALQNDSADIMAAFDDLNMVPTLFFAFGNAYVPGFENLPLADLRKRKAAKKVLFQAVHDVIAAKLSAPPTTSRDLLDLMLDSPEHKITADEARVHVMTFLFAGHETTSNTLCWVFAMLATHPDVEARVVDECRRVMATGHLSWEAFGELKYLTAVIHETLRINPTVVNLSMRVCETDDYIPMVDGKPVFCPAGTQIAMSTAVIHRNATYWTRPNEFLPGTLTVCRSSHQHLLFTPFSTGPKNCIGNRFALAELQVVLATVVSEYQFSLHAS*

>CYP5617J1 S. declina

MDLALVCGLLALGLGLAFLYFFVLAPAWSPLRAIPGPAASHIIFGNLKEIIDTKWANGHYPEPGLKWTLQYGGAVHYRAFLSHRILLTDPEALKHVFLTHGDTYPRDPTARRFLSNLTGGDGLLSSEGDVHTGMRKLLMPHFGYAKVKTFVDVFQHHTAHLMRHLDTVVASGASVDMHDLFTKLTLDVIGVSAFGYNFESLAGSNSTTLEAYHMMNNTPSLAYFVGSLILPGFKHYPLPRLVKMKRAKAILFKVVDDVIAKKLAHPRDIATDLLDLMLDESTQTDHKVSAAEARVHVLTFLLAGHETTSTTLAWVFTLLAQHPEAEAKARAEARAVQKAYGSISYAALGDLKYISAVIHETLRIFPTITTLASRIADADDHIPLESGKPIFVPKGTTIVANTGVMHRNPLYWSRPTEFLPDRFLDDSDVYLADKGLRGGRGNTFFYMPFSAGAKNCIGMRFATAELQVVVATLLTQFSFALAPEANTNPKLSGVSLKPVQLTMHVNHA*

>CYP5618A1 S. declina

MATINASLHDAITYYQCGGVDLLKSLSPSVVRLDVLLLRTLVAVAVVALSLCFLPTTWATIAQKHRIASALRSLPGPPGLPLLGNLIQLGQHMHDLHLWKRHLATVYGSTYAIRVDLVMNGSIVTNKPCNIEHILSTNCSNYVKPQIIQDVCKEVMGQSIFAINPDSPLWACQRKLMANMFSVNSFRKYMDSVFVDAADAALGRLDAAARRNDTVNLETTLLTLTTTIAFRIGFGRHVPEAMTTDAFHGLFREAGAITANRFTRPWYKFAGAVLPSEARLRTVVGEIDAALYALIAERKAAPVTPSAVDVLSQLLDQQRRGLLELSDTVVRDTMMTVLLAGRETVASGLLWILYCVSRHPAVEAKLLAEVDGVSSVDYESMSSLVYMEAVMKETWRLFPPTPLELKAAVADDVLPDGTFVPAGVNVEFSPFVMNRDPTRWRNPDAFEPERWLEPGFSPTDFEYPVFNAGKRKCVGQRIAMLQTKFILCKLYRHLRFDMLDEKDPTFALGISLFPTNGMAVKPVLRRPSSTLSTTPAPAA*

>CYP5618A2 S.Parasitica

MATINANLHDAITYCQCDFLKTLAPSDVRLDVLLLRTLVAIAVVALSLHCLPRTWAMMAQKRRIASALRSLPGPPGLPLLGNLLQLGQHMHDLHLWKRHLAAVYGPTYAIRVDVVMNGSIVTNKPCNIEHILSTNCSNYVKPQIIQDVCKEVMGQSIFAINPDSPLWACQRKLMANMFSVNSFRKYMDSVFVDAADDALGRLDAAARRHDTVNLETTLLTLTTTIAFRIGFGRHVPEEMTTDAFHGLFREAGAITANRFTRPWYKFAGAILPSEARLRTVVGQIDAALYALIAERKAAPLTPGAVDVLSQLLDQQRRGLLELSDTVVRDTMMTVLLAGRETVASGLLWILYCVSRHPAVEAKLLAEVDAVSSVDYESMGSLVYMEAVMKETWRLFPPTPLELKAAVADDVLPDGTFVPAGVNVEFSPFVMNRDPTRWRNPDVFEPERWLEHGFSPTDFEYPVFNAGKRKCVGQRIAMLQTKFILCKLYGHLRFDILDEKDPTFALGISLFPTNGMAVKPVLRRPSKLSATPASAA*

>CYP5618B1 S. declina

MVLGFTFAQETAAAVAVGLACLLIAKAVVVYRRKARIAKGLAHLPSLPGHKLLGNLVQQGEHIHDHYYWKLELFETLGSTYAMRVDLLLDGSVCTSSAANVRHILATNHTNYVKPAMMQRALGELMGSGIFNVNPSASSSWKAQRKIIASLFSTNAIKTLMDTIFVEHTSALVSEIETLGDGALLDVEATALSLTTKFTYAMAFGLAFETENATEFRDLFREASNLSVSRFTQPWYQWLGWFMPSEYRLARVMRRINALCHDTIRTKRLSATRGDDLLSELLRRQEAGNDMISDTFIRDMMMTMMLAGRETVGSGLAWVIYTVSKHPIVEARLVSELQAGEISYAGVGCMSYLDAVVRETLRLFPPVPYELKSAITDDVWPDGTFVPA

GTNVEFSPFAMGRNETRWPQATTFSPERWLNDTPRPSAFEYPVFNAGPRSCVGQTVALLQLKVVVASLYQRFTFDLIS
PLNEPAFELGIGLFSQDSIQVRAYLRH*

>CYP5618B2 S. declina

MVFGFTFAQETAAAVAVGLACLLIAKVVVVYRRKARIAKGLAHLPSLPGHKLLGNLVQQGEHIHDHYYWKLEL
FETLGSTYAMRVDLLLDGSVCTSSAANVRHILATNHTNYVKPAMMQRALGELMGSGIFNVNPSASSSWKAQRKILASL
FSTNAIKTLMDTIFLEHTSALLSEIETLGDGALLDVEATALSLTTKFTYAMAFGLVFETENATEFRDLFREASNLSVSRFTQP
WYHWLGWFMPSEYRLARVMRRINALCRDTIRTKRLSATRGDDLLSELLRRQEAGNDMISDTFIRDMMMTMMLAGR
ETVGSGLAWVIYTVSKHPIVEARLVSELQAGEISYVGVGCMSYLDAVVRETLRLFPPVPYELKRAVTDDVWPDGTFVPA
GTNVEFSPFAMGRNETRWPQATTFSPERWLNDAPRPSAFEYPVFNAGPRSCVGQTVALLQLKVVVASLYQRFTFDLIS
PLNEPAFELGIGLFSQDSIQVRARNRDSL*

>CYP5618B3 S.Parasitica

MALGLTFAQETAIAVAVGLTYMLVTKIPLFYRRKARIAAALARLPSLPGHKLFGNLVQQHEHIHDHYYWKLELF
ETLGTTYAVRVDLLMDGSVCTSSAANVQHILASNHTNYVKPAMMQRALGELMGRGIFNVNPSASSSWKAQRKIIASLF
STNAIKTLMDAVFLEHTSALVSEIEAQGDGALLDVEAIALSLTTKFTYAMAFGLVFEAANATEFRNLFREASNLSVARFTQ
PWYRWLGWFMPSEYRLARVMRRVNALCHDTIRTKRLSATRGNDLLSELLRRQEAGDDLISDTFIRDMMMTMMLAG
RETVGSGLAWILYAVSKHPDVEARLLSELQTSEITYASVGCMSYLDAIVRETFRLFPPVPYELKSAVADDVLPDGTFVPAG
TNVEFSPFVMGRDHTRWPQATTFSPERWLTGATRPSAFEYPVFHAGPRRCVGQAVALLQLKVVVASLYQRFTFELIAP
LNDPAFELGIGLFSQDGIQVRAHNRRSL*

>CYP5618B4 S.Parasitica

MALGLTFAQETAIAVAVGLTYMLITKLPLIYRRKARIAAALARLPSLPGHKLFGNLVQQHEHIHDHYYWKLELFE
TLGSTYAVRVDLLMDGSVCTSSAANVQHILASNHTNYVKPAMMQRALSELMGRGIFNVNPSASSSWKAQRKIIASLFS
TNAIKTLMDAVFLEHTSALVSEIEAQGDGALLDVEAIALSLTTKFTYAMAFGLVFETANATEFRDLFREASNLSVARFTQP
WYRWLGWFMPSEYRLARVMRRINALCRDTIRTKRLSATRGNDLLSELLRRQEAGDNLISDTFIRDMMMTMMLAGRE
TVGSGLAWILYAVSKHSDVEARLLSELQASEITYASVGCMSYLDAIVRETFRLFPPVPYELKSAVADDVLPDGTFVPAGTN
VEFSPFVMGRDHTRWPQATTFSPERWLTGATRPSAFEYPVFHAGPRRCVGQSVALLQLKVVVASLYQRFTFELIAPLN
DPAFELGIGLFSQDGIQVRAYLRP*

>CYP5618C1 S.Parasitica

MNLVSGHLVLLALAAYVVVAIARYVYHKRTMAAALATLPGPPSTWLLGNSGQIAGHVTELYDWKLRQTIAYG
ATYCLRVDVLTNGSIFTSSAANLEHILSKRHANYIKPPVMETVLRELLGSSIFNINVSHPSWRFQRKLIASLFSVNALKAWT
TTTYTKHLASLVQALDTESSSIDLSPLVLSWTTHCIYEMAFGATLDADDMQAMHDLVHEAGELIFYRFTHPWYVLFQW
CMPSEFRLHAVMTRINALCNQTITNARATATHESTNVLAELLRRQATDASITDVLIRDMMMGMFLAGRESVGSSILWA
LYCVATHPHVEAILVDELGTAEISFESIASSVYLDAVVRETLRLFPPVPIELKCAVADDVLPDGTYVPQGAMLEYSAYVMG
RDPRRWADADQFQPDRWLSMATRPTAYELPTFNAGVRSCVGQQAALLQTKLVVATLVQRFHLDVLSPKRQDGSLYA
LGLALLPRGGLHVRARARCSLGLA*

>CYP5618C2 S. declina

MDLLLGHYILLALAVYALVAIARWLHHKRTMVSALSRLPGPPSSLFLGNLAQIAGHVHELYDWKLRQTIAYGA
TYCLRVDVLTNGSIFTSSAANLEHILSKRHANYIKPLLMETVLHELLGSSIFNINECHPSWRFQRKLIASLFSVNALKAWTST
TYTKHLGPLLQTIDAEKSVDLAPLVLSWTTHCIYEMAFGATLDADDMRVMHDLVHEAAELIFYRFTHPWYLLFQWCMP

SEFRLHAVIARINALCYKTIAHARSSTMHASTTVLAELLRRQATDASITDVIIRDMMMGMFLAGRESVGSSILWALYCVA
KHPHVEAALVTELGDIIISYESIGACGYLDAVVRETLRLFPPVPIELKCAVADDVLPDGTFVPQGAMLEYSAYVMGRDPSR
WVDADQFRPERWLHMATRPTAYELPTFNAGVRSCVGQQAALLQTKLVVATVVQRYHLDVLSPKSDDEGPMYALGLA
LLPRGGLHVRARAR*

>CYP5619A1 fusion protein S. declina

MGNLTSTGATHGDVHMDKMVMYLDGDRSAMMDGDLFVLEKALATEKVVGFCGPEALKVFDANLRDGTF
VRHGALPSGLNELLGAVLPTTDGDAHARKKKLVLAAFSGAQLAAYKPLIRTTIQNEHAKWAAHGASMSLVANAKVLVF
KLSLLLILGLEDNYDNSRELLDTYMLALRNSVRRADPAGVRSRDELIRTMINPALATSHDRVHTGKPKPCALDHLVAAGV
LSDDDLRAELFHLLCMSLGGLECWVANCITAAASSTDVLAQLTAGRDAFITKYPAEADRWSHLGDLGYVNNYIQEVKRT
YVAGPSHMYARATKDTDVRTSEGTFHVPKGCLVAAALDGTNKHPSVWANPTKFDPSRFSTAKVDMAFGFCPHAIGA
DRRCAGEELSTLILQSFMVSLFDFMWKMLPHQDYTLDTTLVNPMPKGGLMVVGFHRRTDLSASMVEVAGSEEDWKF
LSLPEAKVYRDDKEALHDMFADERLDLWTHLMLKLLAKKQSMWNKPFANQAITAPKYQKTLPKITLYGLKIQIPTEDED
WPSDPWNEVATVKFLRDSCPLGDDFEHTWLPGEDMERYVMSKVGSMWPRVNVHWNDRYSDRALELLVFNGLGQ
HLVTKLRTAHDDGSYYGICLDFMQALDVRPGYAKYGADAYFNAKGKVTKIVRLGKTVHPGDEDWEYAKLCFRGSLQTK
VTALDHLLGIHITVANGLVTSTREQLPPTHPLRRLLKPFTFRSVIINYNASYALFWPKGMLHRAFSLSVEGMQQTWELGL
ANFKYETFPEHKARQNIDTTTLPYHEDGMDFWLIVRGFVGSYIDLYYPCDESLTQDTAVQAFWSYLKTTLPPNSIRPLSK
DNIKDFVAHAIFLVSSMHNHLGTIAEYVSDPAFCPSAWVEGELAGRPGPCVRGALIMAATGFVQPSIKEDFSHIMLDDA
AKAVCRKFTADVCAYAAVVEGRNTKRQHPYQAFNPNTMEMAVSI*

>CYP5619B1 fusion protein S. declina

MGSQASTPAGAAPSLRRAASLKKMIMFMKDPRTAMMDCRDHYGDVFLMESSLVNEKIMGFCGPEALLAY
DTQVKEGKIVRASAFPTGILELLGAVVSTLDDDAHAKRKAALLVAFTPEKLDAYKPKIREIIQHDHAAWAARGGSLSLALS
CKKMVFHVFMATLLGLENVDDEYRELVEAFVSSIRKSARKPDTTGMDARTQVVEELIRPAVREAKARVAAQKPLPTVVE
VLVADGRLSDEELNLELFHALFAGLGGVTCLVINAVTACIELPAIREKVSAAREAFLAKYPNEDDRWSHFADLGYMHHFI
LEVKRFYVAGPTQLYGRATDDLEISTANGSFKVPKGCLATAGLEVTSKHPDVWSDPHTFNPDRFAPQDASTTPVDPDAF
KDGARDVTAPDMMYKFCPHSIGIARRCAGEGLTTLVLQCFVVSLFDFIWQMVPGQNYQLEEKSSTPTPIGQLMAVGF
HRRTLDDVVTFGTAGSDEDWHFLSLPQAKELVGSGTADLYDDARMDLWTRLMIKLIGKKQATWDRPFVESCLTIPKH
QKVLPKLTLIQTSIEIPTEDEDWPKQPWLEIKQSNFLRDHAPFIDDFKHTWLPGEDMERYVMSKLGHMWPRVNVHW
NDRYSDRALELLAFNGLGQHLLMKLPEAHDDGSYYGICLDFMNVLEVRPGYAKYGADAYFTAKGKVTKIIRGGVTSRPG
EDGWEYAKLCFRGSLQTKVTAVDHLLGIHATVANYMVTSIREQLPPAHPVRRLLKPFTFRSVAINFGAGRSLFWPKGML
QRAYALTDKGMKQTWEYGLANFKYETFPERKARQSIDTVTLPFHEDGIEYWQICRTFANDYVDLYYKSEDATSADADLK
RFWTFLDEKLPFTMRPLNLENLKDFLAHGIFLVSSMHNHLGTIAEYVSDPAFCPSAWVEGELAGRPGTGVRLALIMTAT
GFTQPDITEDFSHLMLDDAAKAVCKAFTAAVIAQIAVVDARNATRVQPFQSFNPKTMEMAVSI*

>CYP5619B2 fusion protein S. declina

MGNEASTVHADGAATDLPASHRAMNILKMIEFSKDPRAGMLESRDQFGDLFLLESHLVSEKIAGFCGPELLA
AFDDKLRDGSIVREGAFPPGVLALLGPIMSTIDGEEHDARKAAALEALTPARLDLYAPIIREIVEAEHASWAARGGAISLA
CLTRDMVFRIFLKVLYGVERHDGNKFRVLLDDFIVSIRRSSKHADPHGVRCRTQILDELIRPAIANAQARASNKTPVPSVI
DCLVANGKMTPDVLETEAFHFLFAGFGGVACLATNILTAVATHPSARKDLLDARAEYVTKYDGDARWAHFHDLGYVNL
FILEVKRFYVAGPTAVFGRTKTDLEIPTKNGVYKLPKGCLAAAGLEATNRHPDVWTDPNLFNPNRFRDLGHVRTTKPHA
FCPHAFGESSHRRCAGEDLTTLILQSTVVSLYDFVWQMVPNQDYKLAVGSSTPTPVGQLMAVGFHRRTDDAVEIIGTV
GSKADWKFLNLPEAKELVGTAMDLYDDARLDLWTRLMIKLIGKKQAVWDRPYANQILRIPQHQKPLPKITLIQTNIDIAT

EDEDWPNQPWLEIQQSNFLRDHAPFVDNFEHTWLPGEDMERYVMSKVGSMWPRVNVHWNDRYSDRALELLAFN
GFGQHLLTKLPEAHDDGSYYGICLNFMKSLEVRPGYAKYGADAFFTSKGKVTKIIRGDIASRPGDSGWEYAKLCFRGSLQ
TKVTAVDHLLGIHATVANIMVVANREQLPPTHPLRRLIKPFTFRSVAINYGAGRALFWPKGMLQRAYALTDKGMKQTT
QDAPAHRHNDAAVP*

>CYP5619C1 fusion protein S. declina

MGNQPSTEAGVAPLPDSKRANSIFSLLAFAKDPKAAMAESRDTLGNLFLIESAVVSEKIAGFCGPEMLSQYDA
HVAAGHIVRENALPAGIVELLGPILATLDGDVHDSRKEAIMGAFSKEMLASYAPIVFEIVQKEHAAWAAHGGEISLALSC
KKTVFKVFLAILYGITNLTPAEYDAKFDPFRDLLDSFIRAIPKSSKGADAEGLVCKQRLLDELVAPALAASQARVEAKAPVP
CFLDYMLGQTELTPDVVHLEAFHALFAGLGGTQCLVVNTITALAQYPTVAEKVHASRAKFVIKYHDDRWRHFDNLGYC
NRFLLEVKRFYSAGPAQLFGRTTQELTFTTPDGEFAIPKGVLAVAGLDATNRHPDVWTDPSVFNPDRFDNGFSEASDLY
KLCPHAIGKTTGGRKCAGRDLATLVLQASLVSLFDFKWTLVPNQDLSLEEGKSTPMPKGLLMASSFTHRHSESETECDV
ADWHLLNLPEAKALVGIAGTVSDDEDDARLDLWTRLMIKLIAKKQARWNKPVANEVLTVPQFQKELPKMTLIQTNIQV
ATEDEDWPNQPWLEIQQSNFLRDYAPFVDNFEHTWLPGEDMERYVMSKVGSMWPRVNVHWNDRYSDRALELLAF
NGFGQHLLTKLPEAHDDGSYYGICLNFLKGLEVRPGYAKYGADAFFSAEGKVTKIVRGDVTVRPGDDNWAYAKLCFRG
SLQTKITAVDHLLGVHATVANIMVIANREQLPPTHPLRRLIKPFTFRSIAINYGAGRALFWPKGMLQRAYALTDKGMKQ
TWDIGLANFKYETFPEHIARQNIDTTTLPFHEDGMDYWHICRSFVSNYVDLYYKSEDALQNDTDVHAFWTFLSTKLPVP
MRTLTLENLKDFVAHFIFLVSSMHNHLGTIAEYVSDPAFCPSAWVEGELAGRPSTGVRLALIMTATGFAQPAITEDFSHI
MLDDAAKAVCQAFTAAVTAQIAVVDARNATRVQPFQSFNPKTMEMAVSI*

>CYP5619D1 fusion protein S. declina

MVSLPLLVIVIVGQVAGAPQGLGSVLQGVINDVKHSVAGVRYVFESLVDAEPVTGFCSPEALRAFDDALASGA
LERRTAYPTGILELTGPTLSTIDGPAFLKRQDAFLNALSGAALSTYQPRIQRRIQEDHATWAARGSTFSLALYAKTSTFKVF
LDVVYGIDDPEKYTGHRAQLDEYLFYLSKTSSRAPSDAAKIREHLLAAIVRPAIASSLARVRSGAPLTCVLDTVVAQGTVSE
ADLALESFQLLAMGLPGLEGLVVHTITAMVSLDDVRGQMATARDAYTAKYPGGAFWSHLDDLDAVNQYVNEVQRVC
GASPRHTFARATKDFSVPSGSGATVAVPKNRLTVVLLDCINNDPKRWPSPEQFQPARFAAANTSAYGFAPFAIDDLVH
RAEGRREGLSRLILQSHVVSLLDFVAVMAPLQSFALGDGVNPLPIDLLTTVSFRYVPGVVQGDIDAWRRLHHPSAKLYN
GSLENPLLAASDKRLDFWTHSMIQLFNVRFETWVTPTAAASIKVPTTQKNLPKRTLYGTSIQIPTEDEDVAIPKVILESAKL
LQDTAPFVDNFDAKWAPGEDMEGCVLSKVGRMWPRVRVHWDDRYSDRALELLVFNGLGQHMVQKLATAHDDGS
YYTVATNYLASIEVRTGYAITGADAFFDKNGKVTKIVRLGKTIRPIDASWEYVKMCFRSSLVSKITAVDHLIGLHVTVGNY
MTTGSREQLPPTHPLRRLIKPFTFRAVAINYDASIALFAPKGMLHRAFPYTEKGLKDTWAMALKSLTLEPFPVHLARQQV
DTITLPYHEDGADYWEIVRTFVSEYLDLYYTSNDDVTHDVSIQALWTFLNKQLPTPLGVLSLDNLKDVVAHSIFLVTAMH
NHLGGIAEYVSDPAFCPVSWVEGELSGRPGNAVRAALIMSGTGFPQPNILEDFSHVLLDDAAKAVAHRFTASLQAFVH
VVEARNAQRIHPYQAFNPAVMDMAIGI*

>CYP5619D2 fusion protein S. declina

MVALVPLLLTAVGVVGTQENTLKGFFQGVISDIKHAVTDVRYTFESLVNAEPVTGFCSPDALRAFDDALSSGAL
SRHAAYPAGLLDLSGPTLSTLDGQAFAIRQESLLNALSGPSLAAYQPRIQQLIQDDHATWAARGGTFSLALQAKTTTFKV
FLAVVYGVTQPDEYVGYRAQLDEYLEYAKKTLSRAPSDAIKIRDRLLATLVRPAIAASHARVRAGAAPTCVLDALVAQNT
MSDSDLATEGFQLMAMGLLGLEGLVVHTITAMVSVDGVRGQLGSARDAYVSKYPNGAHWRHLDDLSVVNAYVNEV
QRVYNASPRHTFARATKDFVVTNSSSVPKHSLTAALLDCLNYNAARWPSPAQFQVARFAGANPSAYEFAPFALNDLVD
RRAGRREGLSRLILQTHVVSLLDFAAVMAPLQSYALDDGLNPLPVDLLTTVGFHYAPGVAHSSNAYDDAWRRLRQPSA
KLYNSSIESPLSSDKRLDFLTHSMIQLLNVRFATWVTPTAAASITVPKSQKPLAKQTLHGTSIQIPVDDEDVSIPKVLLDGA

KLLQDTAPFVDNFDDSWVPGEDMEGYVLSKVGRMWPRVRVHWDDRYSDRALELFVFNGLGQHMVTKLSAAHSDG
SYYTATTSFLETLDVRPGYAVTGADAYFDKNGKVTKIVRLGKTFRPADAQWEYVKMCFRSSVANKVTAVDHLIGLHVTV
GNYMTTASREQLPPTHPLRRLIKPFTFRAVAINYEASKLLFAPKGILHRAHPYSEKGLKDTWAMALQSLKLEPFPVHMAR
QNIDTLKLPFHEDGMDFWTIVRGFTGEYLNLYYESDEDVTRDASTQAFWAFLDKQLPTPLGALSLESLKDVVAHGIFLVT
AMHNHLGGIAEYVSDPAFCPVSWVEGELAGRPGAAVRTALIMSGTGYPQPSILEDFSHVLLDDAAKAVAHRFTTSLQS
FVMVVEARNAQRVLPYQGFNPAVMDMAIGI*

>CYP5620A1 P.aphanidermatum

MSSLHPPSLGDASSVQVALGAALATAMAYKLLVSPWLEPPPHERYAAMNGLKDVKVARRLKQSSLLLGDMP
ELQRNMHRFNDWIHDKTLECDEQPWILHVPTQPDTMVIASPALIEDITKTQNDIFGKGDDQREMLSDLAGYGISIADG
KIWYHQRKVASKFFSARMFRDVATRIINKHANCIFDVLAATYKTGKELNFTSLLHQFTLQTFAELGFGLDMHAIDEAESL
KLEQAFDKAFPQVLRRVQTPTWLWKLKRYLGIGSEGEYRKTTQYLRQFVSGMISKSIEKIRTEGERKEAATIVELFIHAMQ
DGEADFPPERLCDIAMVFVLGGRDTTADTLNWLFYMLSKHPDXXXXXXXXXXXXXXXXXXXXXXXXXXXXXXXXXXX
XXXXXETLRLYPVLPQNGRQASRDTVLSDGTFVPQGTVMALATYAMGRLESIWGEDAAEFKPERFIDETTGKLKVFSSY
KFFSFHAGPRVCIGLSLAMLEMKLFIASLLSRFRLDTAPNDGQYDFSFLMPLKDPLLVRVRPASSSAP*

>CYP5620A2 P.aphanidermatum

MVSVVLPSSLQEASSLQVAAGAAVATFLAYKLLFPSKAPHERYAAKHGLKNFLEMDEQPWLRVVPSLPEMLM
VASPALIEDITKTQNDIFGKGDTQRELFVDQLGNGFFAQDGESWFHQRKVASKFFSARMFRDVATKLINKHARVFFNVL
ERARVNNEAVDMTQLTKQFTLETFAELGFGLEMNAIGSKEDHALSRAMDEALPQVIFRLQVPSVIWKTQRFFNIGGER
KHKEVTDFIRKSVNDVVVKCIDKIEREGRRQEAATVVELFIHAMKDGETEFSAEVLSNVAIMFMMAGRDTTADTLSWFL
YTLSKHPEVETKIRAELWDQVPDLMEGKIDFLDMEQTHGLVYLEAAIKETLRLYPVVPLSARQASRDTVLVDGTFVPKGT
VVGLATYAMGRLKSVWGEDAAEYKPERFIDETTGKLTTISSFQFFSFHAGPRVCIGMNLAMLEMKLLLSAMMSRFHLDI
APNSGAYNVNASLTVKEPIMAHVRLTKSN*

>CYP5620B1 P.aphanidermatum

MVLVLPPTAKLDATQVALAGGALLAAYKLVQWYRSPVEKTPAAATIHTLPSTLPVVGNMLDAIQHGDQWHD
WITQHCVELEGALWRLKMPGRADVIMISTPEMMEDVTKTHFDTFIKGPFQHDWLRDYMGDGIFTSDGERWVNLRK
TAARFFSARLLREHMTECIKKNAVDVYRVLDNAVERDEVVNLHQLFKEFAMQAFAKMGFGIDINCVGASEDHPFQAA
MDTAAQVLVTRFRRPVWVWKLLRWLDVGVEAKLSRAIAITNEAVYRMARESIEITTNPATKLAQANGTYRPKGVLELFL
QAVDSEHMKTIKVEDLRDFALVFMVAGRDTTSDTFAWFFYHVLKDPVVERKLREELARKLPDFLKSPTADSLKMEDMA
SLVYLEATIKETLRLYPQPPLNIRECTQDTVLSNGALVRKGELIGLAAFAMARNPVVWGDDAAAFNPDRWIDANSHQLK
TISSFKFIAFHAGPRSCLGENLAMLELKMLIVNLFARYTFDMLPGNDGAYDVSMAMSLKHPLLARLKKASYKDC*

>CYP5620B2 P.aphanidermatum

MVRVLPPESSPDAVQLAAASFAVLVAYKLVQWWRSPATKTVDSAKIRTVSSTLPVLGNMLDAMKHGHCLH
DWISSTCNELQGQLWRLKIPSRADVVIVSTPDIMEDITKTHFESFVKGDYQRDWLRDYLGDGIFSSDGDRWVHQRKTA
ARFFSARLLREHMATCIQKNALDVYNVLDKVAQNGGALDLQQLFKEFAIQAFAEMGFGIHLNCIGASEEHPFQAAMDT
ATRVLSLRFRRPVVMWKLLRWLNVGEEAELRRSVKIIDDAVYKMARESIDATFNPAVKDAKKNGTYKAKSVMELFLHA
VESESMNTIKLEDLRDFALVFVVAGRDTTADTFTWFFYLVSQHPEVERKIREELTRKLPEFMNGTRREALRTDDIVGLVYL
EAVIKETLRLYPLPPLNIRECAQDTVLSNGALVRKGELVGLATFAMGRNPLVWGEDAASFRPERWIDPDTLKLKTVTSFK
FIAFHAGPRSCLGESLAMLELKLLIVNLFARYKFDVLPGNDGAYEMSMSMSMKEPLLARVRIHHASLMPLSYSFCSALA*

>CYP5620B3 P.aphanidermatum

MVLDTGTLILWSVGFALAWSVLVVFRKPAKAPPGTRPIRTLDTTLPVLGNLLDHVKHGHRFHDWLSDNCIKFK GDLWKVTIPGQADVVMVSTPDVVEDITKTHFDNFIKGEYQRDWMHDFLGDGIFTSDGERWVHQRKTASRFFTARVL REYMTKCIQKNAQDVYSVLDKASSSGAVVNLQQLFKEFAIQAFGEMEEHPFQAAMDTAVRVLSLRFRRPVWFWKLLR RLNVGAEGELRRSMAIVNEAVYSIARQSLETSREKRIFTDEEHQAHHAKSVLELFLSAVESDSMESIQLQDLRDFALVFVL AGRDTTADTFTWFFYMLSEHPEVEQTIRDELARMLPEFMQAPNARALKMDDVAHLTYLEAALKETLRLFPLAAFNVRE VAVDTVLSNGALVRKGQLMGLSAYAMARNPVVWGDDAASFRPERWIDATTGRLAVVSSYKFLTFHAGPRTCLGQNL ALLELKLMIVNLLSRFSFHVLPGNDGSYEMSLTMPMKHPLRARIT

>CYP5620C1 P.aphanidermatum

MADVEPLGPIVAGVAVVTVALAWWLSAPSQDKKKRRNAQGVYELDAVFPVFGSFFDRVQNAHRMYDWM ADYSRQVDEAAWVFRLPGQPDRIMFTAPEALEAISTEYFDNFRKGAYQIECITDMFGAGLVASDEARWYHQRKAAVKF FSAKTLRALMTDSMQKNMTKVCHVIDGKMAQGQQVDLKQLFMEFTMDTFAELGMGVDLKCIGAANPHPFQHALD VIAPLVSRRFRVPSWWWKAERRLNIGPEAVVAHHKTVVDQWLAETIDKILVSARSKSYRPNDSRIKSIVELFVEYGVDDA EGVQPEDLKHFLLTFVVAARDTTATTLSWLFYLLGQHPQVEERLRSELSSKVETHHSSGYLTSDHLKSTVYLDAVIRETIRL FPAASFTMRECVSDTEVCPGVMVRKGQLAGMCAYAVNRNPKVWGEDANTFRPERWIHPSTGELLHVPLTSFFSFGA GPRHCIGMNLAMLELRIVTANLLSRYQFEIDRCNDGSYVVAPTMQMKHPLRAMATPL*

>CYP5620D1 P.aphanidermatum

MLPPTSILKRLSSFLESICLGLQPQILTLATLVTLLGATAWLVLMKTSLKPLPKSSTVLDSRQLPRPPSTLPVLGNLI DVLSNQQRYHTWVHELCVKFNYRPWRLELPGQPPIIMLSSAEQFEEVMTTKADSFGKGQVQLDQVRDFVGENLVSTV GQPWYHQRKIATKFFSARSLNAYMASSAHRNLMKIKDILHTHQSNDVPVHLSNLIYDFTLQSFVETGVGAKLDWLGQT ERHPFQVAVDEAAVLVARRLRLPSWWWRTLRALNIGYEARLASHIAYIDSWLTKTVNVGLAALFTRQAGFPDSNAEPE AKSVIEFFLENSRDDREGMRSSDLVDFIRVMLLAARDTTAVAMAWFFFEIGRNPRVEGRIREELWRVYPTRAGKRTEYF TPQDARDLIYLEAAIKETLRLHTPVAENRREVAEDTIICDNILVHKGDFVGLSAYAAARNPNVWGRDAAEFRPERWIDEK TGVLRQFPNTKFFTFGAGPRICVGKSLAMLELRVVAVNLISRFHFELDESVTGEYGASLLYSLKDPVIARVYPAQLRE*

>CYP5620D2 P.aphanidermatum

MLPITDLLASLSSSPNAWTQPNLMSEPQTLALATATMIAVLSATAWFVSAASSPKLLTSDGKPLKPRELPRPPN TLPVMGDMLTILSNQERYYSWIHELCANFNYKPWRLEIPGQPSTIVLSSAEQFEEVMTKKTGRFDKGQVQIDQIRDFIG DNLVSSVGEPWYHQRKTATKFFSARSLNAYMASSARRNLTQIMDILHTHQENGTSVNLSSLIFDFTLQSFVETGVGVKL DWIGRKEPHPFQVAIDEASDLIAKRLRMPTWLWRVMRTLNVGYEAKLASHMSYIMSWLANIVNEGLEAQAAKKNGA PNANDEPEAKSVIELFIENSRDDLDGMRADDLVDFIRVMLIGARDTTAITLAWFFYEISKNPRVENKIREELHRVYPTRAG KSSEYFSPQETRPLVYLEATIKETLRLHSPSGENRREVTEDTVICGDALVRKGEFIILSGYSAARNPKVWGPDATVFKPER WIDETTGELRQFTNTTFFSFGAGPRLCVGKGLAMLELRAVTCNFLSRFHFQLDESVGGDFGISFLYNLKDPVIAKVFPAS VLSQK*

>CYP5620D3 P.aphanidermatum

MRPSDLVEFIQIMLIGARDTTAATLTWFFYELSQNPRVEAKIREELWRKYPSRAGQRTEYFSPEEARALVYMEA AIKETLRLHPPAPSNYRVVAEDTVISDDVLLRKGVVVCLSVYAEGRNPKVWGPDATEFKPERWIDEASGTLRQVPNAKF ATFGAGPRICIGKGLAMLELRVVVANLLSRFHFELDENTHDKYQLGILLGMKSPVIARVQAAASPVL*

>CYP5620E1 P.aphanidermatum

MCWSILQSSTMEASAGIQGSLTASFLATAAVGIGVALVLSAWVVSATTNAKDGASTDHMLRLDTTHPFLGDL
RTFAANASRLHYWLTEQSLRFHGEPWRIRMPLGMKNLVVVTHPEAVEDVLIKQFDNFPRGEHRIQVIEDVLGRSVVTS
DGAKWYHLRKIASRFFTANALRKCMMTSVQHGMSQVYEQIDKDVASNDAVNMLKLFQEFSLQVFSEAGLGLRPQFIG
NTTGHPVEHSMDRSTMMVMRRLRLPMWYWKLERWLNIGPERELAAITRSLRQWLRSVVDESMEVAAKKQKAKQS
DRDPDDDGIKSIVELFMEQSGEESGMQPQDLVDFILTFLVASRDTSSVALSWFFLMLAKHPDVEKRGLSAEGFLTSDHV
RQLFYLEATIKEVLRLYPSVPVIQKEALHDTLLCGKYAVKKGELVVIENYGMGRMPNVWGPDAAEFKPERWIDPQTGGL
ITVPTSKLNAFGSGPRICIGMKLAMVELRVATANLLSRYQFSLARPNDGEHVVAIELMLKDPLMMKATPVKRGSEPIQ*

>CYP5620E-fragment1 P.aphanidermatum

ALHRRVHSLPGSSVFLGDLIEFVANPHRLHDWIPETSQRFEGEPWRIRLPGLRPCVIVTKPEIIAEVTTRQYDSFP
RGEFVNELSEDLVGRSIVTSDGERWYKLRKMAAKFFTARALRMCMTTSMRRATFEMYAFIDETIAQGESIDMFKMFHE
FAVQMFAKDTVLCDTYEVKQGELVIMQQYAMARLPSVWDLDAAEFKPER*

>CYP5620F1 P.aphanidermatum

MLDEYAATIALTIVVGGALLFAATLRTREAAHVAKTLGGCRPRKIHSLGTRIPFIGDFLEITRQTHRKHDWMVE
QCLRFHGEPWQAKIPGMPAFVMLTSPEAVEEVTTTQFDHFAKGPFQISVLNDLLGNGVFASDGEQWYHQRKTAAKFF
SAKSLRECMANTMQRNMERVHSVLSRARPDDELVDLSHLFHQFTLQTFTEGGLGVEMEVIGSKQMHPFEDAVDTAM
PIISRRGRLPPFVWKLERWLSVGQEKQLADSMRVARRWLSSMIEASIRAMERQHGNQQHRNDNDNVKSVVQLFVDH
TREDMAGLQATNLVDFLLNFVIAARDTSALTLCWFFFALRQNPAVEKRIRREMAEKIALNTTYLTADDAKKLVYLEATM
KETLRLYPAGPNTVKYVVRDTVICDDIFLREGQMISLSAYVLARNPRVWGHDAAEFKPERWIGASGELTLAPASEFFTFH
AGPRSCLGMSLAMLALRVVTANLLHRYEIEVDPANDGSYVNSPALAMMHPLLAKVRPRGV*

>CYP5620G1 P.aphanidermatum

MVLLPSACDHFGLTLGIGIAGALALLALWPRSPSHIKLTEKPYRKIHNLDGTQPIVGDLPEYARNAERFHDWLN
ENCLRVNNEPWRMRIVGQNNVLVFSSPDAFEDILVTQFDNFPRGEYLNSMLEGVFGRAIITLDGEQWYRQRKAAAKFF
TAKALRVCMLHTMHKNVLQMYKALDKSMRDSKPINFTNLLHQFTLHTFAEIGLGXXXXXXXXXXXXXXXXXXXXXXXXX
XXXXXXXEFYWKLERFLNYGPERELARVMNDVRAWFGGIIDQSLRSARERVERREASNAVDDENAIKSVVELFVESSQS
DLEGLRAEDLVDFLLTFVLAAQDTSALTLAWFFYAINKYPVVEKRIRDEMEEILPGLGIKNDTYLTTEHISKLVYFEATMRE
TLRLYTTVPLMQKVAEKDTVVCGDIPVRAGESVILSMYAMARNPHVWGPDAGEFKPDRWIDQANGELLSFPATKFAA
FSAGPRICIGMKLGMLEIRVVAANLMRRYKLELQPPSDGSYLVGITLSHKQPLHMKVTHSA*

>CYP5620G2 P.aphanidermatum

MRLACPSSSPDQLDSAALALGGMWVNNAHRLTKTSMILGDLSEYLENAHRLHDWMSETCLRFNGEPWRM
RIIGQRDMIVFSTPEALEEILVTQFDNFPHGDQTMRKNVSQLNEALDRSIAEHKEFDLSNALHQFALQTFSEIGLGVELQ
WIGKDDKHPLATFHHVAPAVMRRFRVPTSYWKLERFLNIGPEGRLATVMKDVRKWFNTLIEESISKIVARQQTSGGYA
EADENRVKSVVELFVEQSSQDIDGMRAEDLTDFLLLFVLAAEDTNAITVSWLIVALQRYPHVEKRIRDEMNSVLPRLNVT
EEMYLTSDHVGKLVYFEATLRESMRLXXXXXXXXXXXXXXXXXXXXXXXXXXXXXXXXXXXXXXXXXXXXXXXXXXXXXXX
XXXXXXXXXXXXAGPRNCVGMKLGMLEVRVLAANLVHKYHLEPVDDHHGHYQTGITLDTQKPLRVR

>CYP5620G3 P.aphanidermatum

VTHTKRSSSTAVSQEETHKKVLTLPTSRAVVGDLLEYLQNADRFHDWLTENCLLLHNEPWRLRIFGQKDMIVV
SSPEAFEDIFVTQFDNFPRGEYLNSMFEDVFGKSMITFDGEQWYRQRKAAAKFFTAKALRICMLKIMHKNVLQMYDTL

DGHIASGEPADFTRLLHQFTLQTFTEIGLGIDLRWIGMKEPHPLEEIDTVGSAATMRRFRLPDFYWKLERLLNFGPEKQL
AQAMKEVRQWFGSILEQSLKSARHHQANLRHEDESDEPVVKSVVELFVESSDEEMEGLRPENLPDFLLSFVLAAQDTS
ALTLGWFFYAVNKFPEVEVRIRREMEAILPALDVTEDTYLTTDHVSRLVYFEATIRETLRLYATVPLTQKVTVNDTTICGGV
PLKAGEGIILSMYALARNPRVWGPDAAEFKPERWIDQATGDLLSFPPTKFASFGAGPRICIGMKLGMLEVRVVAANLLR
RYNLKLISPNDGGYLVGVSLVHKDPLEMRISRATASASVKKE*

>CYP5620G4 P.aphanidermatum

MTAAVKAVREWFDVVIKQSLETIAKRQASQKPSDEEPIKSIVEMFFENSSEDKSGLRSDDLVDFLLSFVFAAQD
TTVLTLTRFFLALADHPEVEKRVREEMKAVLPTLGVDRDTCLTADHAQHLTYLEATIREVLRLYPTEPATQRIAAEDTIICG
DVQIKKNDQIGLSMYAMARNPSVWGPDAAEFKPERWVDKSGSLRVEPPTRFATFGAGPRQCIGMKLAQLEIRVVAA
NLLHQYEFALAAPNNRDYLVGITLMLKEPLMMKVKRA*

>CYP5620H1 P.aphanidermatum

MLRSTLPVLGNMLDMARNSHRLLDWLVDCCREFDNNPWKRRIPGQPEMIFFSSPDAMEEITTTQFENFETG
EVQIEMIEGLFGRGLASDGERWYHQRKTAVKFFSARSLRAFMTKSMHKNLEQAQESETLVDLKKLFHEFTLQTFVEMG
LGINLDWIGSENPHPFQKAIDTASPLLVRRFRRPRWLWKLERWLNIGPEATFASSMKIVHAWLHEVLEQSLTETVSNKK
KAMDYLDDEEIKSVVELFVQSSRDDADGIRSEDLVDFLLTFVIAARDTTAVTLSWLFYELGQYPRVVKALQDEMTSRLPA
NIVDNKAAYLTTDHTRNLTYLEATLKEVLRLHPAATTTVKQAVRDTVVCGDVPVYKGQLVLLGAYAMARSPHVWGED
AAEFKPERWINATTGELLQFPAAKFFSFHVGPRTCIGMNLARRDCQLAASFPLRNRP*

>CYP5620H2 P.aphanidermatum

MPSMEAPATALVAVSCALVGAVWLLRKNVSRTPTGLRAIPVPSDTLPVLGNLLVYLMRNDTLREWGVEQCL
QFRGQPWRRYIPGQPELITFSNPDAIEEITTTQFECFEKGEFQIDIISGLFGRGLVASDGERWYHQRKAAAKFFSARSLRT
CMAHSMRKNLGQLCGVLDEAARSGTPVNLKHLFHEFTLQTFVEMGLGVELQWIGQKGERHAFPQAIDAASPLMAM
RFRRPSWFWKMQRWMDVGAERELGEKMRT*

>CYP5620J1 P.aphanidermatum

MVLAPSSLALVHEAPLALVLGVALAVVALVVSRRASPSKRHDASTPPDPPTLPSTLPVLGNLLDMLREGDVLYD
YIDRGCKQFDYEPWKFTVPGRPDFVVLSSPEAIEDVTITQFDVFPKGEFIRECVRDMFGDGMITSDGDRWYHQRKIGAK
FFSAKVMRKLMHQSVYKNLKRVHDVLDASIATGAQVELTKLFMEFTIETFAETGMGVKLNCIGATEPHPFQEGLDESAP
CLDECVKKSNNISEDDIKSVVELFITYGNKDGVEGVEPQDLLDFMLTFVIAARDTTADTLSWMMLKLRDHPEVMRKIRD
EIRAVGDTIGLCASSMGRNPKVWGPDAAEFKPERWINPDNKNELLQFPATKFFSFSAGPRMCIGMNLAMMELRALA
ANLFYRYQFDVDPSNDGSYVVAPTFNMKYPLLARVHRL*

>CYP5620J-fragment1 P.aphanidermatum

MVLTSLSLTLELGTKFFSSKVMRKLIHDSVYKNLERVCEVLDRYAENDNQIDLMKLFLEFTIETFAITGLGVELNC
IGAKEPHPFQKGLDAPAPVVMRRARMPTWYWKLERWLCVGHERSLATHMGNVRAWLNEVIHRSLEDRIKKKKARKL
HSEDEIKSVVEMFIESSSEDELSNVWGPDAAVFKPERWINPNNKRELLQFQSTKFFSFSAGPRSCIGMNVAMMQL*

>CYP5621A1 P.vexan

MGAGGVYYPGPLGGMLVWLLRPLQGSTSPPTVAIATRRPPRQSAESSSSLETLPRPATTLPLLGNTLDVLRLG
DSFAEWVCGTTRLVGGRLWLLQMLGRAPTVVVSSPELIEDVQRAQFAKFGVLSGSDDDDGLSKLFGAGLSAAXXXRW
RMQHKTLAAALGSLEMRDTMLQVVESHSMVLETVLEAAEVVDVKKLLRLFATEVLVDMAVGVELGQLKTQSPSGLQR
AMDAVVAAVANRQLLPPFMWKLLRLLNVGSEAVLASNAVAMRQEMDRLLSHSLTARHRRPTLVRMIMAHRPSTSPF

TSELLRDMVLNVVLSGRDAMADTMAAFLVCMATHSEVQARVYAEVAGLGTDITPETLDRLVYLEAAVKETLRLYPPHP
LVRRFALEDVKLADSTRLPRGTCVVTVAAALARRTDVWGMDAAHFRPERWIVDEDGGPRLRRVSNFQFNALLAGPRA
CPGASIAMPQLKLVLARVLSRLSVAVPSTGSHQIGQGANLHPHLLRFERREHKVHPEADEPHAIAA*
