# Supplementary material for: Diversity and evolution of cytochrome P450 monooxygenases in Oomycetes
Source: Sci Rep. 2015 Jul 1;5:11572. doi: 10.1038/srep11572 (PMC4486971; doi:10.1038/srep11572)
Supplement: Supplementary Figure S1 [file srep11572-s3.pdf]

Diversity and evolution of cytochrome P450 monooxygenases in Oomycetes

Mopeli Marshal Sello[1], Norventia Jafta[1], David R Nelson[2], Wanping Chen[3], Jae-Hyuk Yu[4], Mohammad Parvez[1], Ipeleng Kopano Rosinah Kgosiemang[1], Richie Monyaki[1], Seiso Caiphus Raselemane[1], Lehlohonolo Benedict Qhanya[1], Ntsane Trevor Mthakathi[1], Samson Sitheni Mashele[1*], Khajamohiddin Syed[1*]

* Corresponding authors email: khajamohiddinsyed@gmail.com & smashele@cut.ac.za

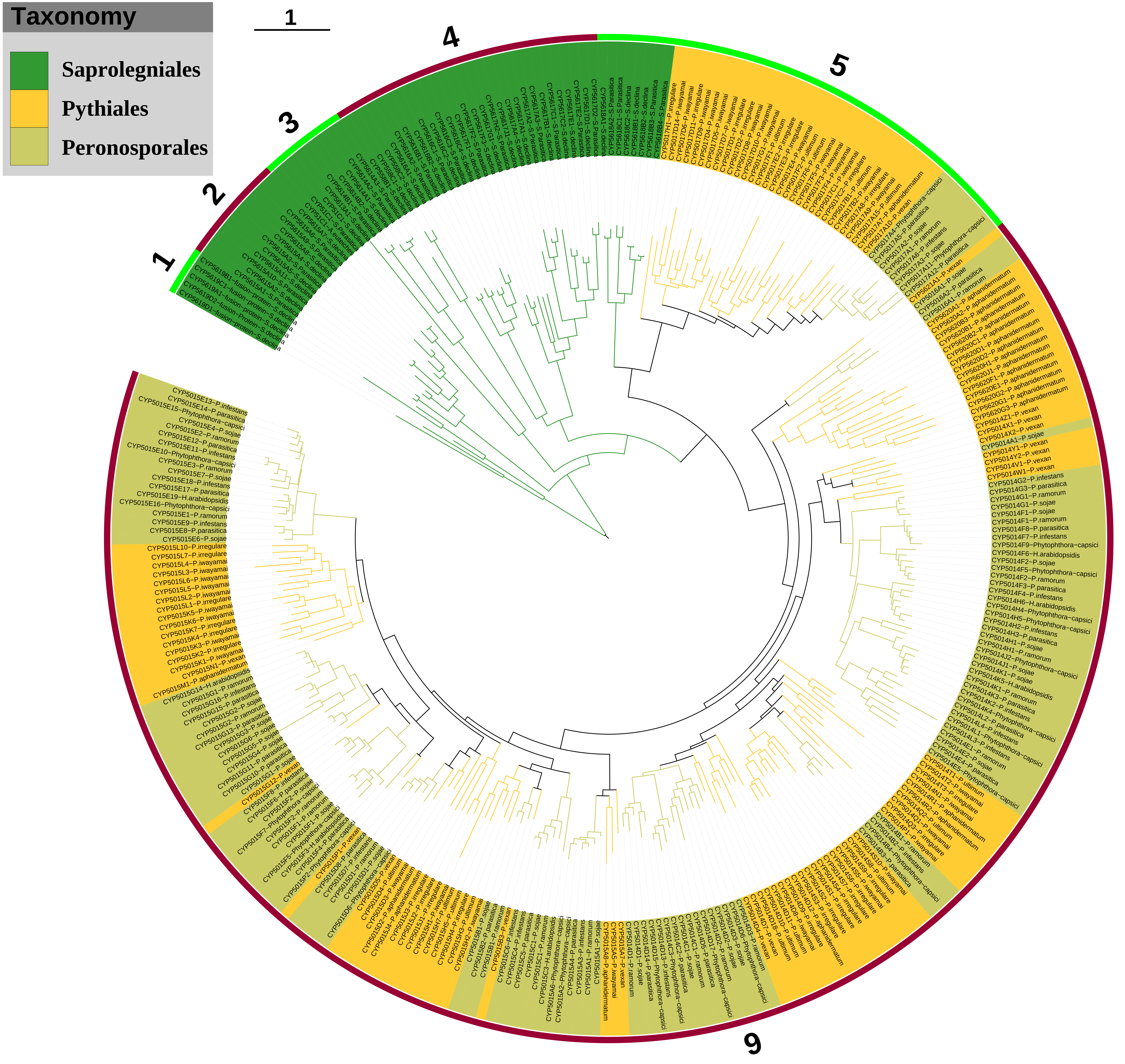
Taxonomy
Saprolegniales
Pythiales
Peronosporales
1
1
2
3
4
5
9
